# Formalin-fixed paraffin-embedded renal biopsy tissues: an underexploited biospecimen resource for gene expression profiling in IgA nephropathy

**DOI:** 10.1038/s41598-020-72026-2

**Published:** 2020-09-16

**Authors:** Sharon Natasha Cox, Samantha Chiurlia, Chiara Divella, Michele Rossini, Grazia Serino, Mario Bonomini, Vittorio Sirolli, Francesca B. Aiello, Gianluigi Zaza, Isabella Squarzoni, Concetta Gangemi, Maria Stangou, Aikaterini Papagianni, Mark Haas, Francesco Paolo Schena

**Affiliations:** 1 Schena Foundation, Research Center of Kidney Diseases, Strada Provinciale Valenzano-Casamassima Km. 3.00, 70100 Valenzano, Bari, Italy; 2 Division of Nephrology, Dialysis, and Transplantation, Department of Emergency and Organ Transplantation, University of Bari, Bari, Italy; 3 National Institute of Gastroenterology “S. de Bellis”, Research Hospital, 70013 Castellana Grotte, Bari, Italy; 4 Department of Medicine and Aging Sciences, University “G. D’Annunzio” of Chieti-Pescara, Chieti, Italy; 5 Renal Unit, Department of Medicine, University-Hospital of Verona, Verona, Italy; 6 Department of Nephrology, Hippokration General Hospital, Aristotle University of Thessaloniki, Thessaloniki, Greece; 7 Department of Pathology and Laboratory Medicine, Cedars-Sinai Medical Center, Los Angeles, CA USA

**Keywords:** Transcription, Acute kidney injury, Chronic kidney disease, Gene expression analysis

## Abstract

Primary IgA nephropathy (IgAN) diagnosis is based on IgA-dominant glomerular deposits and histological scoring is done on formalin-fixed paraffin embedded tissue (FFPE) sections using the Oxford classification. Our aim was to use this underexploited resource to extract RNA and identify genes that characterize active (endocapillary–extracapillary proliferations) and chronic (tubulo-interstitial) renal lesions in total renal cortex. RNA was extracted from archival FFPE renal biopsies of 52 IgAN patients, 22 non-IgAN and normal renal tissue of 7 kidney living donors (KLD) as controls. Genome-wide gene expression profiles were obtained and biomarker identification was carried out comparing gene expression signatures a subset of IgAN patients with active (N = 8), and chronic (N = 12) renal lesions *versus* non-IgAN and KLD. Bioinformatic analysis identified transcripts for active (*DEFA4,* *TNFAIP6,* *FAR2*) and chronic (*LTB,* *CXCL6, ITGAX*) renal lesions that were validated by RT-PCR and IHC. Finally, two of them (TNFAIP6 for active and CXCL6 for chronic) were confirmed in the urine of an independent cohort of IgAN patients compared with non-IgAN patients and controls. We have integrated transcriptomics with histomorphological scores, identified specific gene expression changes using the invaluable repository of archival renal biopsies and discovered two urinary biomarkers that may be used for specific clinical decision making.

## Introduction

Traditionally, fresh or frozen tissues are used to extract RNA for transcriptomic studies as they usually provide high quality nucleic acid. Great importance is now given to the valuable underexploited resource of formalin-fixed paraffin-embedded (FFPE) specimens from routinely collected biopsies. These specimens originate from patients with a long term clinical follow-up and are promptly available for processing^1–7^. Kokkat et al.^8^ have demonstrated that no significant differences have been found in terms of quality and quantity of genomic DNA, total RNA, and total protein extracted from blocks stored over decades compared to recent blocks. FFPE-derived microarrays have been demonstrated to be reliably applicable in transcriptome profiling showing a high level of data reproducibility, relative gene expression concordance and are able to identify similar differentially regulated biological networks as those found from fresh or frozen tissue RNA source^7,9,10^.

Immunoglobulin A Nephropathy (IgAN) is the most common form of primary glomerulonephritis that is characterized by circulating immune complexes and polymeric IgA1 mesangial deposition^11,12^. The disease shows a wide range of clinical symptoms i.e. recurrent episodes of gross hematuria in concomitance of upper respiratory tract infections or persistent microhematuria with or without mild-moderate proteinuria, but the definite diagnosis requires a kidney biopsy^13^. The histologic evaluation of the renal lesions is based on the “Oxford classification”. Four types of renal lesions are scored: mesangial hypercellularity (M 0, 1), endocapillary hypercellularity (E 0, 1), segmental glomerulosclerosis (S 0, 1) and tubular atrophy/interstitial fibrosis (T 0, 1, 2)^14^. Recently, an extension of the MEST score has been suggested adding the glomerular extracapillary lesion (Crescent; C 0, 1, 2) to the Oxford classification because E and C are predictive of active lesions^15,16^ and these lesions are able to identify patients at increased risk of poor outcome without immunosuppression^13^.

Renal transcriptomic studies on IgAN have been performed on microdissected glomeruli and tubulointerstitium, but obtained data are related to these separate compartments^17–20^. However, the histologic pattern of a kidney biopsy is characterized by the simultaneous occurrence of glomerular and tubulointerstitial lesions with varying degrees of involvement, thus we sought to perform a transcriptomic study on whole renal cortex to identify genes and secreted components contemporarily involved in glomerular and tubular damage^21^.

Aims of our study have been (1) to apply transcriptomics on whole renal cortex from FFPE renal specimens of IgAN patients to understand the molecular pathways involved in different degrees of kidney damage; (2) to identify specific gene expression changes of the active renal lesions that may be more responsive to immunosuppressive therapy^22,23^; (3) to discover differently expressed non-invasive biomarkers detectable in the urine that characterize active and chronic renal lesions to avoid a second kidney biopsy, a procedure that is associated with substantial risks i.e. bleeding^24,25^. These complications are even more relevant when co-morbidities are present and impede this invasive clinical procedure^26^. Therefore, there is an urgent need for the identification of new measurable biomarkers, specifically urinary biomarkers seem to be the best option as they are able to give an indirect screenshot of disease activity at the renal level, and valuable in assessing the efficacy of treatment^27,28^. Here, we have described MEST-C-associated gene expression changes in biopsy specimens and evaluate specific urinary protein excretion.

## Materials and methods

### FFPE tissue specimens and urinary samples

Archival FFPE kidney biopsies of patients with glomerulonephritis were collected in a multicenter study from three Renal Units (Bari, Chieti, and Verona, Italy). Clinical and demographic data of the microarray study cohort are summarized in Table 1A. This cohort included 52 IgAN patients, 22 non-IgAN patients [Membranous Nephropathy (MN) n = 11, Minimal Change Disease (MCD) n = 9 and Focal Segmental Glomerulosclerosis (FSGS) n = 2] and 7 Kidney Living Donors (KLD) that were used as controls. All biopsy specimens were collected from drug-naïve patients before immunosuppressive treatment; At the time of kidney biopsy, all patients gave their informed consent to use remaining portions of needle-core biopsy specimens for research purpose after its primary use for routine histologic staining. Therefore, no formal ethical approval was required for processing archival kidney biopsies for our study. The same processing protocol was performed in all three renal pathology units and were carried out manually. The biopsies were fixed with 10% neutral buffered formalin solution (Sigma-Aldrich, Merck KGaA, Darmstadt, Germany) at room temperature for 8 h but no longer than 24 h. Tissues were dehydrated through ascending grades of alcohol and cleared in xylene and embedded into paraffin blocks. Biopsy specimens were stored at room temperature for a mean time of 1–12 years. IgAN patients were scored using the MEST-C classification by three pathologists (MR, FBA, CG) and stratified into 4 groups with different composite renal lesions based on the E, C, and T (but not M or S) scores: minimal (E0, C0, T0); active (E1 and/or C1, C2, T0); chronic (T1 or T2, E0, C0); mixed group composed of active and chronic lesions (E1 and/or C1, C2, T1 or T2). A control group of 6 patients with biopsy-proven lupus nephritis (LN) with active proliferative lesions (Classes III/IV) was added for immunohistochemical (IHC) staining (Table 1B). Urinary samples were collected from an independent test cohort of 51 IgAN patients with active (n = 34) and chronic renal lesions (n = 17). These patients were selected with the same histological criteria used to define FFPE active (E1 and/or C1, C2, T0) and chronic group (T1 or T2, E0, C0). Furthermore, non-IgAN of which MN (n = 4), MCD (n = 4), hypertensive diseases or diabetic nephropathy (n = 15), LN (n = 11), vasculitis disease (VS, n = 6), and healthy blood donors (HBD n = 14) were also collected. Urinary samples from this independent test cohort were collected from three Renal Units (Bari, Chieti, Verona, Italy) and one from Thessaloniki, Greece (Table 1C). LN patients had a pathologic diagnosis of active proliferative LN (Classes III/IV), and the vasculitis patients had PR3 (proteinase 3) or MPO (myeloperoxidase)-ANCA (anti-neutrophil cytoplasmic antibody) associated vasculitis syndrome. Urine samples from an independent cohort of patients were collected at the time of kidney biopsy and used for the detection of soluble potential biomarkers. Urine collection was approved by the local institutional Ethics Committees (Istituto Tumori “Giovanni Paolo II”-Bari, Italy, Nephrology dept. University of Thessaloniki, Greece and Azienda Ospedaliera Universitaria Integrata Verona, Italy) and all patients agreed to participate in the study and signed written informed consent. The study was carried out according to the principles of the Declaration of Helsinki.

**Table 1:** (A) Clinical and laboratory features of 52 IgAN patients, 22 non-IgAN patients [Membranous Nephropathy (MN) n = 11, Minimal Change Disease (MCD) n = 9 and Focal Segmental Glomerulosclerosis (FSGS) n = 2] and 7 Kidney Living Donors (KLD) included in the study. (B) Clinical and laboratory features of active proliferative Lupus Nephritis (LN) patients (Classes III/IV) used for IHC analysis. (C) The test cohort is made up of 51 IgAN patients, 23 non-IgAN patients (4 minimal change disease, 4 membranous nephropathy, 15 hypertensive disease or diabetic nephropathy), 17 patients with lupus nephritis (LN) or vasculitis (VS) and 14 Healthy Blood Donors (HBD).

	A Multicenter study cohort			B IHC analysis	C Test cohort			
	IgAN	Non-IgAN (MN/MCD/FSGS)	KLD	LN	IgAN	Non-IgAN	LN-VS	HBD
Number	52	11/9/2	7	6	51	23	17	14
Male/female	38/14	10/12	1/6	1/5	36/15	15/8	5/12	8/6
Age (years)	41.19 ± 13.00	47.85 ± 18.61	54.83 ± 13.75	45.00 ± 13.65	41.47 ± 14.73	61.25 ± 15.39	43.88 ± 15.46	43.96 ± 11.68
sCr (mg/dL)	1.32 ± 0.57	0.92 ± 0.34	0.96 ± 0.14	1.42 ± 0.98	1.62 ± 0.74	1.71 ± 1.01	1.18 ± 0.39	0.86 ± 0.19
eGFR (CKD-EPI)	73.5 ± 33.36	92.40 ± 28.21	82.20 ± 5.00	81.21 ± 29.24	64.57 ± 31.57	44.73 ± 25.59	52.7 ± 23.84	102.29 ± 25.19
Proteinuria (g/24 h)	1.97 ± 2.41	9.10 ± 6.93	n.d	1.44 ± 1.12	1.73 ± 1.64	6.70 ± 2.86	1.44 ± 0.62	n.d
Hypertension (%)	25%	14%	n.d	33%	41%	45%	30%	n.d
Follow-up (mean time in yrs)	6.64 ± 3.03	5.00 ± 3.66	n.d	n.d	7.89 ± 5.55	7.94 ± 5.49	n.d	n.d

Data is expressed as mean ± standard deviation (SD); *n.d.* not determined; data refers to the time of Biopsy.

### RNA extraction, microarray gene expression profiling and statistical analysis

Total RNA was extracted from the archival FFPE renal tissue samples; beforehand, all working areas, instrument surfaces and pipets were treated with RNaseZap solution (Sigma, St. Louis, MO, USA). RNase-free tips and microtubes were used throughout the study. Paraffin was removed from eight sections (5 µm thick) of freshly cut FFPE tissue sections using a Deparaffinization Solution (Qiagen GmbH, Hilden, Germany) at 56 °C for 3 min. Then, samples were incubated in a lysis buffer containing proteinase K at 56 °C for 15 min to release RNA molecules from crosslinked protein molecules, and then a short incubation was performed (80 °C for 15 min) to reverse formalin crosslinking of the released nucleic acids and then total RNA was immediately extracted using RNeasy FFPE Kit (Qiagen, GmbH, Hilden, Germany). RNA integrity and quality were evaluated. All RNA samples had OD260/280 > 1.9 confirming the purity of RNA. The RNA Integrity Number (RIN) was evaluated with Agilent RNA Pico Chips and run on Agilent 2,100 Bioanalyzer. RNA was in the range of 2.1–5.0; the most abundant RNA fragments were in the range of 100–200 ribonucleotides for all samples (Supplementary Figure 1), values similar to those found in other articles^2,5,7^, (Supplementary Table 1). Total RNA (300 ng) was converted into cDNA using the Whole-Genome cDNA-mediated Annealing, Selection, extension and Ligation (DASL) HT assay (Illumina, San Diego, CA, USA). The DASL assay contains probe sets that span about 50 bases, which allows the profiling of partially degraded RNA samples. The cDNA was then hybridized to the HumanHT-12 BeadChip, and then scanned on the HiScanSQ (Illumina Inc., San Diego, CA, USA).

Gene expression data analysis was done using Genespring GX 14.9 (Agilent, Santa Clara, CA, USA). Raw data was uploaded on GenomeStudio software (Illumina) and checked to confirm no outliers. PCA was used for this scope. Gene expression data analysis was done using Genespring GX 14.9 (Agilent, Santa Clara, CA, USA). Raw signal values < 1 were set to a threshold of 1 and all values were log2 transformed. Each sample was then normalized using a 75th percentile shift algorithm in which the log2-transformed intensity value corresponding to the 75th percentile was subtracted from log2-transformed intensity value for each probe within a sample. Baseline transformation was then carried out to rescale intensity values to the median of all samples. For each probe, flag Information was taken into consideration, the lower cutoff for 'Present' call was 0.8 and the upper cutoff for 'Absent' call was 0.6. Microarray statistical analysis was carried out using the ANOVA with Student–Newman–Keuls (SNK) post-hoc test on four groups: IgAN with active renal lesions, IgAN with chronic renal lesions and non-IgAN, all against the KLD group. This statistical analysis generated a gene list of 4,924 FDR corrected *p* value < 0.05 (Benjamii Hochberg FDR) probes (Supplementary Figure 2, Supplementary Table 2). Gene lists for active and chronic renal lesions, and non-IgAN were obtained (Supplementary Figure 2). Specific probes for active and chronic renal lesions in kidney biopsy samples were obtained from the VENN diagram constructed with these significant probes. Microarray data and MIAME compliant are deposited in the GEO database and are accessible through GEO Series accession number GSE116626.

Ingenuity Pathway Analysis (IPA) software (Ingenuity System, Redwood City, CA, USA) was used to assess biological relationships among genes and entities with a FC > 1.5. IPA computes a score for each network according to the fit on the set of supplied focus genes (here, genes differently expressed in active and chronic renal lesions). These scores indicate the likelihood of focus genes belonging to a network versus those obtained by chance. A score 42 indicates a 99% confidence that is a focus gene network not generated by chance alone. Gene Set Enrichment Analysis (GSEA) was used to assess enriched gene sets with microarray data characterizing active and chronic lesions. Every enriched gene set was sorted according to a common biological function using the Molecular Signatures Database, Broad Institute (https://software.broadinstitute.org/gsea/)^29^. We used canonical pathways (CP) of curated gene sets c2, which contain gene sets collected from the pathway databases (BioCarta, KEGG and Reactome) in the Molecular Signature Database version 4.05. Significance of differential expression, as determined by the enrichment analysis, was recalculated 1,000 times. A corrected FDR q-value correction was applied.

### Quantitative real-time (qRT–PCR) analysis

Real-time PCR was used to validate microarray gene expression data of six differentially modulated genes: *Defensin Alpha 4 (DEFA4)*; *TNF Alpha Induced Protein 6* (*TNFAIP6/TSG-6*); *Fatty Acyl-CoA Reductase 2 *(*FAR2)*; *Lymphotoxin beta* (*LTB*); *Granulocyte chemotactic protein 2* (*GCP-2/CXCL6*); *Integrin Subunit Alpha X* (*ITGAX*). Experiments were performed on the same cohort used in microarray experiments. Reverse transcription (RT) was performed using a Whole Transcriptome Amplification Kit (Sigma Aldrich, St Louis, MO, USA), optimized to amplify RNA from FFPE samples. Each RT reaction contained 250 ng of total RNA and the obtained cDNA was purified using a MinElute Purification Kit (Qiagen GmbH, Hilden, Germany)^3^. Real-time PCRs were performed in triplicate using PrimeTime qPCR Primer assays (IDT) on 30 ng of diluted cDNA. Real-time PCR amplification reactions were performed in triplicate in 25 μl final volumes via SYBR Green (SensiMix SYBR Hi-ROX kit, Bioline). The *β-actin *gene amplification was used as a reference standard to normalize the target signal. Amplification specificity was controlled by a melting curve and the amount of mRNA target was evaluated using the comparative cycle threshold (ΔCt) method.

### Immunohistochemistry

Immunohistochemical evaluation of proteins was performed on kidney biopsy specimens from the same patients included in the microarray study (Table 1A) and another control group of LN patients (Table1B).

Thin sections (2 μm) of paraffin-embedded tissue were deparaffinized and hydrated through xylene and graded alcohol series. After antigen retrieval, the endogenous peroxidase activity was quenched by incubation in a solution of H_2_O_2_ 3% for 7 min, the sections were blocked with protein block serum-free (Dako, Glostrup, Denmark) at room temperature (RT) for 10 min. Then, the slides were incubated with rabbit polyclonal antibodies (anti-human DEFA4 1:15, overnight incubation at + 4 °C, Novusbio NBP2-13910; anti-human TNFAIP6 1:200, overnight incubation at + 4 °C, Atlas HPA050884; anti-human FAR2 1:20, overnight incubation at + 4 °C Atlas HPA015884; anti-human LTB 1:20, 1 h incubation at RT, Atlas HPA048884; anti-human CXCL6/GCP2 1:150, overnight incubation at + 4 °C, Biomatik CAU25699) or mouse monoclonal antibody (anti-human ITGAX 1:500, overnight incubation at + 4 °C, Novus NBP2-44598).

The binding of the secondary biotinylated antibody was detected by the Dako Real EnVision, Peroxidase/DAB kit (Dako), according to the manufacturer’s instructions. The peroxidase reaction was shown by a brown precipitate, counterstained with Mayers hematoxylin (blue), and mounted with Glycergel (DakoCytomation, Carpinteria, CA, USA). Negative controls were obtained incubating serial sections with the blocking solution and then omitting the primary antibody. Digital images were obtained by Aperio ScanScope CS2 device (Aperio Technologies, Vista, CA, USA) with 20 × magnification). Aperio specific ImageScope software was used to measure the staining intensity and the percentage of positive cells using the Positive pixel count v9 algorithm (Supplementary Figure 3) For each section, the intensity of the staining with absent (0) to strong (+++) was converted into a number. Only high intensity pixels (identified by the software as strong positive) were considered as a positive staining and were normalized to the selected area (total number of pixels in the section). Immunohistochemical staining analysis was performed on the whole biopsy on both glomerular ad tubular cells.

### Enzyme-linked immunosorbent assay

Enzyme-linked immunosorbent assays (ELISA) for urinary TNFAIP6, DEFA4, CXCL6 and LTB were performed on the independent test cohort (Table 1C). All urinary samples were processed immediately after collection and centrifuged at 3,000×*g* for 10 min to remove cellular debris. The supernatants were then collected, aliquoted and immediately stored at − 80 °C until use. The soluble forms of proteins were detected using a standardized ELISA (BIOMATIK) following the manufacturer’s protocol. Urine creatinine was assayed for each of these samples and values were expressed per millimole of urinary creatininuria to correct for differences in concentration.

### Statistical analysis

Data was expressed as mean ± standard error of the mean (SEM) unless otherwise stated. For normally distributed variables, the two-tailed Student’s t-test was used to assess differences between two groups, while ANOVA test with post hoc corrections (Tukey's Multiple Comparison Test) was done to evaluate statistical significant differences between groups^30^. For non-normally distributed variables Mann–Whitney test was used to assess differences between two groups and Kruskal–Wallis test (Dunn's Multiple Comparison Test) was used to evaluate statistical significant differences between groups^30^. Qualitative variables were summarized as count and percentages and comparisons between independent groups were performed by chi-square and Fisher exact Test. A *p* value < 0.05 was considered statistically significant. All statistical analyses and graphs were generated with GraphPad Prism 5.0 (GraphPad Software, San Diego, CA).

## Results

### Identification of specific gene expression signatures in active and chronic lesions

**Figure 1:**
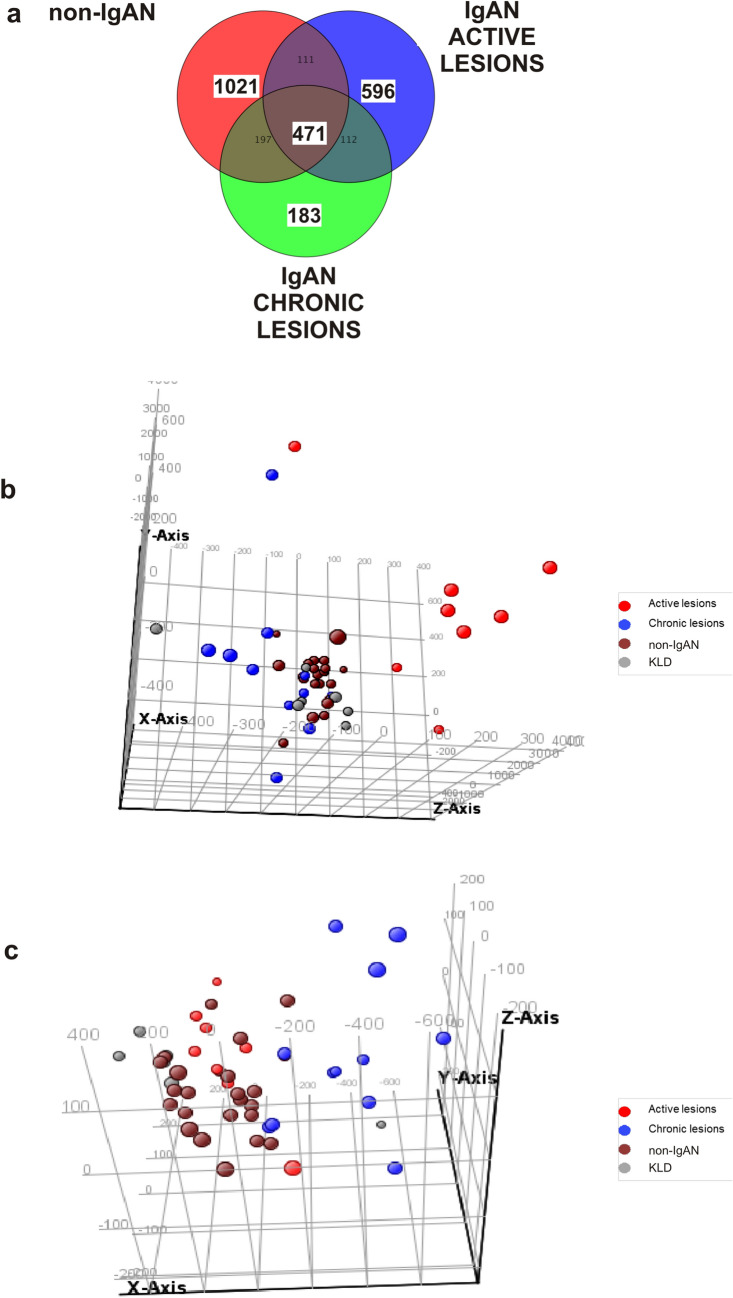
Specific gene expression signatures in active and chronic renal lesions of IgAN. (**A**) Venn diagram depicts differentially expressed genes in active IgAN (n = 596), chronic IgAN (n = 183), and non-IgAN lesions (n = 471) compared to control kidneys. ANOVA using Student–Newman–Keuls (SNK) post-hoc test was applied. Benjamini–Hochberg false discovery rate (FDR) multiple testing correction using asymptotic *p* value computation (*p* value < 0.05) was done. (**B**) Three-dimensional Principal Component Analysis shows different spatial representation of the active (red) renal lesion group compared to other groups (Chronic-blue; non-IgAN-brown; KLD-grey). (**C**) Three-dimensional Principal Component Analysis shows different spatial representation of the chronic (blue) renal lesion group compared to other groups (Active-Red; non-IgAN-brown; KLD-grey).

To identify transcripts associated with specific renal lesions we subdivided 52 biopsy-diagnosed IgAN patients according to the MEST-C classification (Table 1). At first, we performed a differential analysis using the individual M, E, S, T, C lesions, but this grouping contained very few IgAN patients and data analysis could not be performed (data not shown). For this reason we introduced a composite classifier approach, still based on the MEST-C classification system, sub-dividing the cohort into 4 lesions groups: minimal lesions (E0, C0, T0), active lesions (E1 and/or C1, C2, T0), chronic lesions (T1 or T2, E0, C0), mixed lesions (E1 and/or C1, C2, T1 or T2) (Table 2). The minimal and mixed lesion groups were not considered further for microarray data differential analysis. Thus, the first group was excluded because their gene expression profile was not statistically different from the KLD group (data not shown) and the last group was excluded because biopsies contained both active and chronic renal lesions (data not shown). Therefore, microarray data analysis for biomarker identification was carried out on the following four groups: IgAN with active renal lesions (n = 8), IgAN with chronic lesions (N = 12), non-IgAN (MN n = 11; MCD n = 9; FSGS n = 2) and 7 KLD as controls (Table 3). Then, we compared genome wide transcriptomic profile of these four groups using the One-way ANOVA test. A Venn diagram was constructed with the statistically significant gene lists showing the number of common and specific genes for each group (Fig. 1A). We identified 596 statistically significant probes in IgAN group with active renal lesions (Supplementary Table 3) among which we found *FAR2* up-regulated, a gene that is strongly associated with kidney disease^31–33^. On the other hand, we found 183 specific probes differently expressed within the IgAN group with chronic renal lesions (Supplementary Table 4). Since these probe sets have been selected from the VENN diagram containing non-IgAN specific probes this selection automatically excluded probes common to other types of glomerulonephritis. Principal Component analysis (PCA) showed that 596 and 183 statistically significant probes discriminated respectively the active and chronic renal lesion groups from all other groups (Fig. 1B,C). Then, we compared 471 genes that were common to all types of glomerulonephritis (central part of the VENN diagram, Fig. 1A) with genes identified by other gene expression studies performed on freshly isolated specimens^17,34^. We found that 16%^17^ and 35%^34^ of differently expressed genes overlapped with our dataset (Supplementary Table 5) highlighting a substantial gene expression concordance between fresh and FFPE tissues.

**Table 2:** MEST-C Stratification of 52 IgAN patients based on the type of histological lesions.

MEST-C classification	Minimal lesions	Active lesions	Chronic lesions	Mixed lesions
NUMBER	22	8	12	10
**Mesangial hypercellularity (M)**				
M0	15 (68%)	7 (87.5%)	3 (25%)	2 (20%)
M1	7 (32%)	1 (12.5%)	9 (75%)	8 (80%)
**Endocapillary hypercellularity (E)**				
E0	22 (100%)	3 (38%)	12 (100%)	4 (40%)
E1	0	5 (62%)	0	6 (60%)
**Segmental sclerosis (S)**				
S0	10 (46%)	4 (50%)	3 (25%)	0
S1	12 (54%)	4 (50%)	9 (75%)	10 (100%)
**Tubular atrophy/interstitial fibrosis (T)**				
T0	22 (100%)	8 (100%)	0	0
T1	0	0	10 (83%)	6 (60%)
T2	0	0	2 (17%)	4 (40%)
**Crescents (C)**				
C0	22 (100%)	0	12 (100%)	5 (50%)
C1	0	3 (38%)	0	3 (30%)
C2	0	0	0	2 (20%)

**Table 3:** Clinical and laboratory features of 8 IgAN patients with active lesions, 12 IgAN patients with chronic lesions 22 non-IgAN patients [Membranous Nephropathy (MN) n = 11, Minimal Change Disease (MCD) n = 9 and Focal Segmental Glomerulosclerosis (FSGS) n = 2] and 7 Kidney Living Donors (KLD) included in the microarray study biomarker identification.

	IgAN-Active Lesions	IgAN-Chronic Lesions	Non-IgAN (MN/MCD/FSGS)	KLD	*p* value	
n	8	12	22	7		
Sex, male n (%)	5 (62.5)	9 (75.0)	10 (45.5)	6 (83.3)	0.24*	
Age, years mean (SD)	36.62 (15.33)	44.00 (10.72)	47.85 (18.61)	54.83 (13.75)	0.176^§^	
Scr (mg/dL), mean (SD)	1.11 (0.55)	1.58 (0.56)	0.92 (0.34)	0.96 (0.14)	0.001^§^	^#^ *p* = 0.001
eGFR-CKD/EPI [mean (SD)]	88.58 (36.52)	53.85 (27.63)	92.40 (28.21)	82.20 (4.97)	0.004§	^#^ *p* = 0.003
proteinuria (g/24 h) [mean (SD)]	2.03 (2.49)	1.56 (1.06)	9.10 (6.93)	n.d	< 0.001§	^#^*p* = 0.001; ^&^*p* = 0.006
Hypertension, n.(%)	1 (12.5)	4 (33.3)	3 (13.6)	n.d	0.51*	
follow up years [mean (SD)]	7.40 (2.30)	7.36 (2.62)	5.00 (3.66)	n.d	0.261§	

*Fisher exact test; ^§^One-way analysis of variance; ^#^IgAN-chronic vs Non-IgAN; ^&^IgAN-Active vs Non-IgAN. Data refer to the time of Biopsy.

### Network analysis in active and chronic lesions

**Figure 2:**
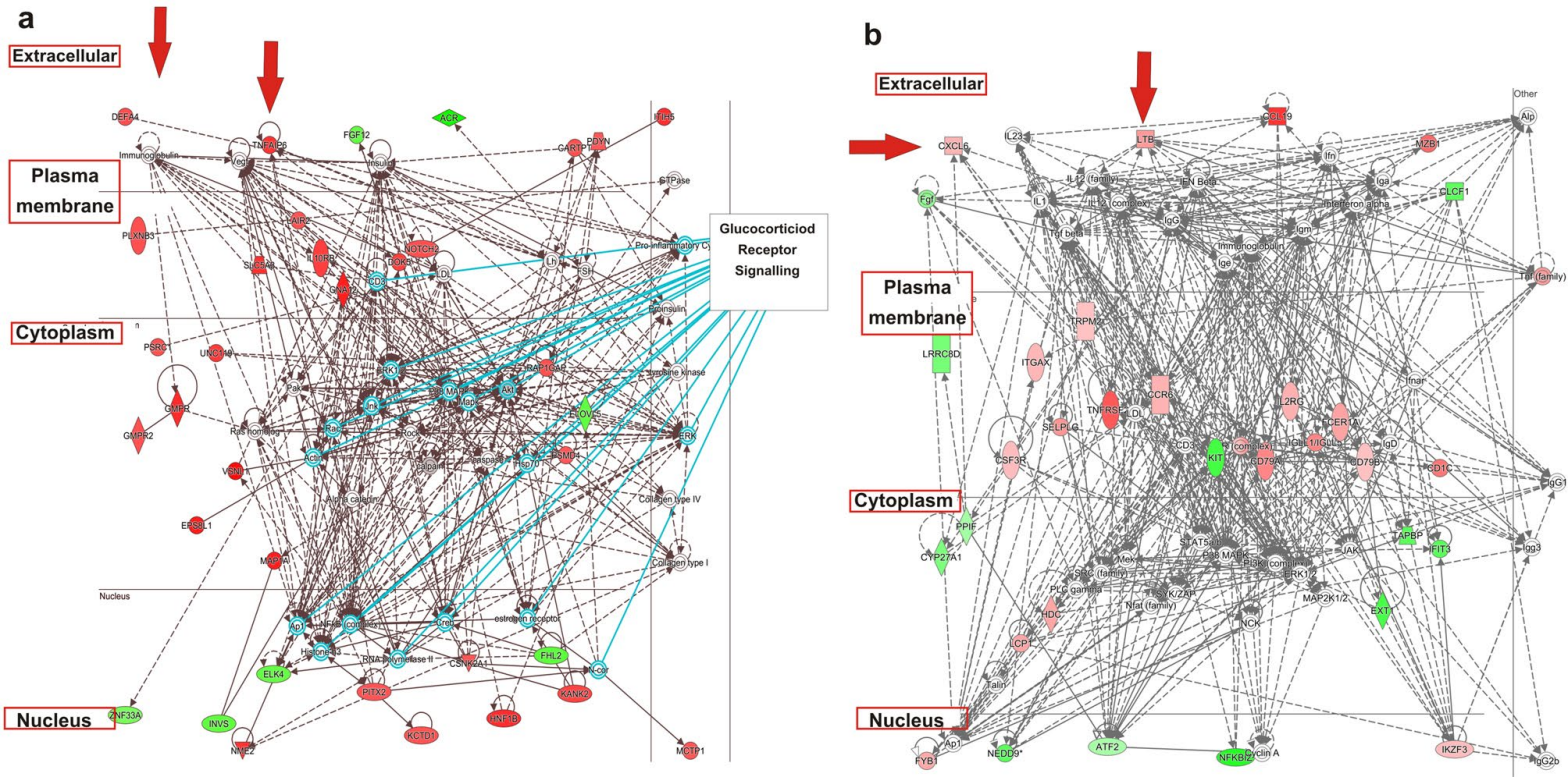
Network analysis in active and chronic lesions. Networks were algorithmically constructed by IPA software on the basis of the functional and biological connectivity of genes. The network is graphically represented as nodes (genes) and edges (the biological relationship between genes). Red- and green-shaded nodes represent up- and downregulated genes, respectively; others (empty nodes) are those that IPA automatically includes because they are biologically linked to our genes based on the evidence in the literature genes have been placed automatically by IPA in different cellular compartments from nucleus to extracellular space: (**A**) the top ranked network for active renal lesions in IgAN shows a high degree of interconnectivity between genes (score 63, n.35 associated genes); (**B**) the top ranked network for chronic renal lesions in IgAN shows a great connectivity between genes (score 56, n = 33 associated genes).

The connectivity between differently expressed genes with a Fold Change (FC) > 1.5 was studied using IPA and network analysis was done using both chronic and active specific genes. The top ranked network for active lesions showed a high degree of interconnectivity between genes (score 63, n = 35 associated genes, Fig. 2A). When we overlaid the most representative canonical pathways onto the top ranked network we found that the most representative was the glucocorticoid signalling pathway (Fig. 2A). Specifically, 18 genes from glucocorticoid signalling pathway were used to construct the network suggesting a potential influence of glucocorticoids in aberrantly expressed genes in this condition. This network highlighted two secreted components in the extracellular space *DEFA4* and *TNFAIP6*, the former gene has been extensively demonstrated to be associated with IgAN^35–38^ and the latter is involved in extracellular matrix stability and cell migration^39^.

The top ranked network for chronic renal lesions also showed a great connectivity between genes (score 56, n = 33 associated genes, Fig. 2B) and surprisingly a cluster of Immunoglobulins including IgA was activated within the network. Many extracellular chemokines involved in renal inflammation were upregulated such as *LTB*^40^ and *GCP-2/CXCL6*^41,42^, another gene (*ITGAX)* involved in cell adhesion and infiltration and extensively associated with IgAN^35,37^ was also found up-regulated.

We also performed a GSEA, a computational method that determines a priori whether a defined set of genes belongs to a specific curated molecular pathway involved in human disease. The most significant curated enriched pathway (C2 collection) generated with active lesions genes was the "KEGG_CYTOKINE_CYTOKINE_RECEPTOR_INTERACTION" pathway (FDR q-value = 2.7 e^−9^, Supplementary Table 6), while chronic lesions genes were enriched with the "REACTOME_METABOLISM_OF_PROTEINS" and "NABA_MATRISOME" pathways (FDR q-value = 2.02 e^−9^, Supplementary table 7).

### Quantitative real-time PCR for *DEFA4, TNFAIP6, FAR2, LTB, CXCL6* and *ITGAX*

**Figure 3:**
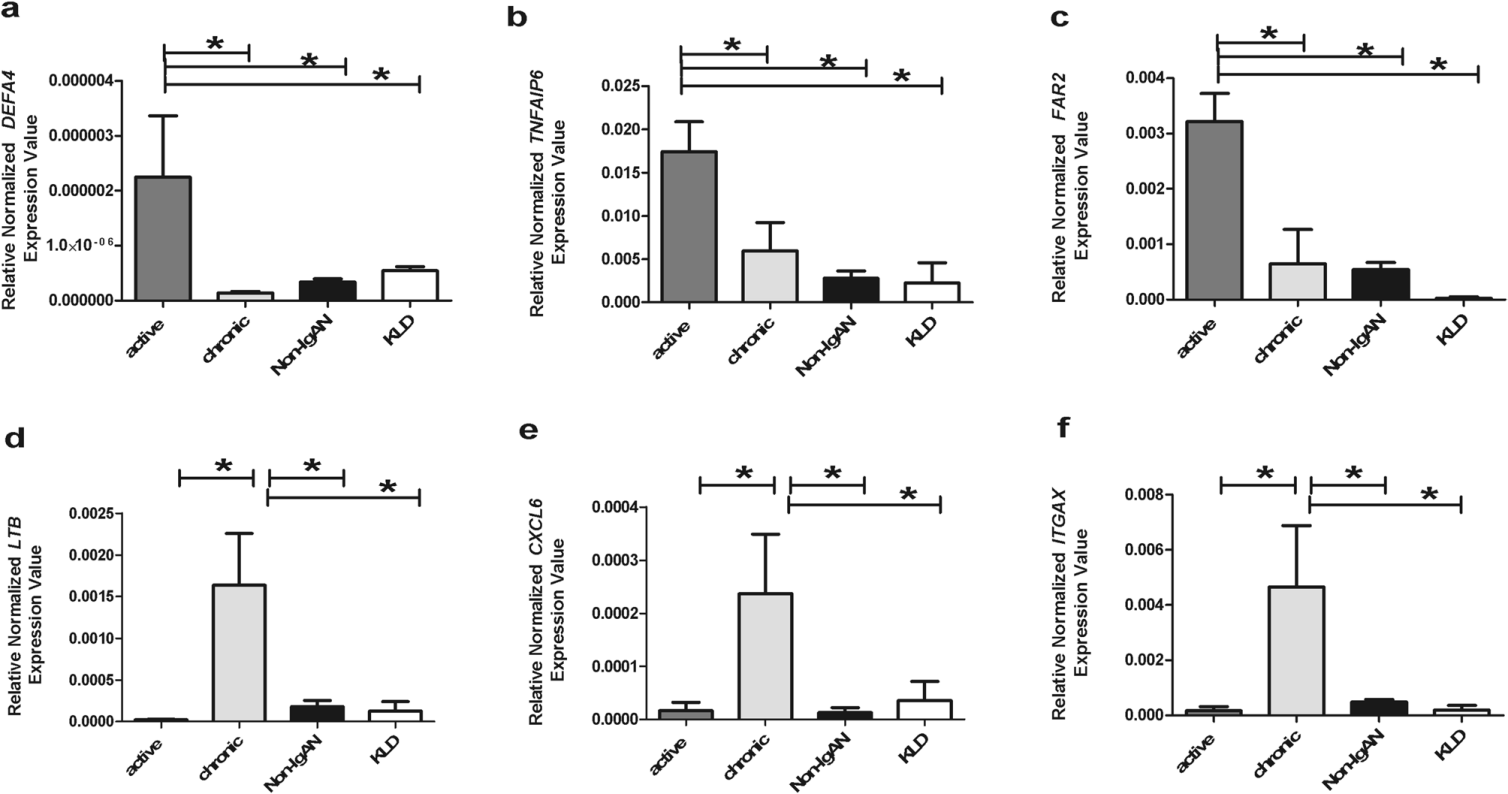
Gene expression levels evaluated by real-time (RT)PCR in kidney biopsy specimens of IgAN patients with active (*DEFA4, TNFAIP6, FAR2*) and chronic renal lesions (*LTB, CXCL6 and ITGAX*), 22 non-IgAN (non-IgAN) and 7 kidney living donors (KLD). (**A–C**) *DEFA4, TNFAIP6, FAR2* normalized gene expression levels are significantly higher in IgAN patients with active renal lesions compared to all other groups. (**A**) *DEFA4* ANOVA F(3,19) = 8.012, *p* = 0.0012; Tukey’s multiple comparison test, active versus chronic, active versus non IgAN and active versus KLD all *p* < 0.05. (**B**) *TNFAIP6* ANOVA F(3,21) = 6.761, *p* = 0.0023; Tukey’s multiple comparison test, active versus chronic, active versus non IgAN and active versus KLD all *p* < 0.05. (**C**) *FAR2* Kruskal–Wallis test *p* = 0.0013. Dunn’s multiple comparison test, active versus chronic, active versus non-IgAN and active versus KLD, all *p* < 0.05. (**D–F**) *LTB, CXCL6, ITGAX* normalized gene expression levels are significantly higher in IgAN patients with chronic renal lesions compared to all other groups. (**D**) *LTB* ANOVA F(3,16) = 5.646 *p* = 0.0079. Tukey’s multiple comparison test, chronic versus active, chronic versus non IgAN , chronic versus KLD all *p* < 0.05. (**E**) *CXCL6* Kruskal–Wallis test *p* = 0.0057. Dunn’s multiple comparison test, chronic versus active, chronic versus non-IgAN and chronic versus KLD, all *p* < 0.05. (**F**) *ITGAX* Kruskal–Wallis test *p* = 0.0009. Dunn’s multiple comparison test, chronic versus active, chronic versus non-IgAN and chronic versus KLD, all *p* < 0.05.

To further establish the validity of gene expression determined by microarray analysis, we performed quantitative real-time PCR (RT-PCR) on the patient groups and controls used for the microarray study. We chose representative genes that were found differently expressed in the active and chronic lesion groups focalizing our attention on those that were present in the top ranked networks (Fig. 2A,B) and were involved in IgAN or renal inflammation favouring secreted proteins. We found that normalized gene expression levels for *DEFA4, TNFAIP6* and *FAR2* were significantly higher in the IgAN group characterized by active renal lesions compared to the chronic group, non-IgAN and KLD [Fig. 3, (A) *DEFA4* ANOVA F(3,19) = 8.012, *p* = 0.0012; (B) *TNFAIP6* ANOVA F(3,21) = 6.761, *p* = 0.0023; (C) *FAR2* Kruskal–Wallis test *p* = 0.0013]. We chose three other representative genes (*LTB, CXCL6, ITGAX)* that were significantly higher in the IgAN group characterized by chronic renal lesions compared to the active lesions, non-IgAN and KLD (Fig. 3 (D) *LTB* ANOVA F(3,16) = 5.646 *p* = 0.0079; (E) *CXCL6* Kruskal–Wallis test *p* = 0.0057; (F) *ITGAX* Kruskal–Wallis test *p* = 0.0009).

### Renal tissue DEFA4, TNFAIP6 and FAR2 protein expression levels in the active renal lesion group

**Figure 4:**
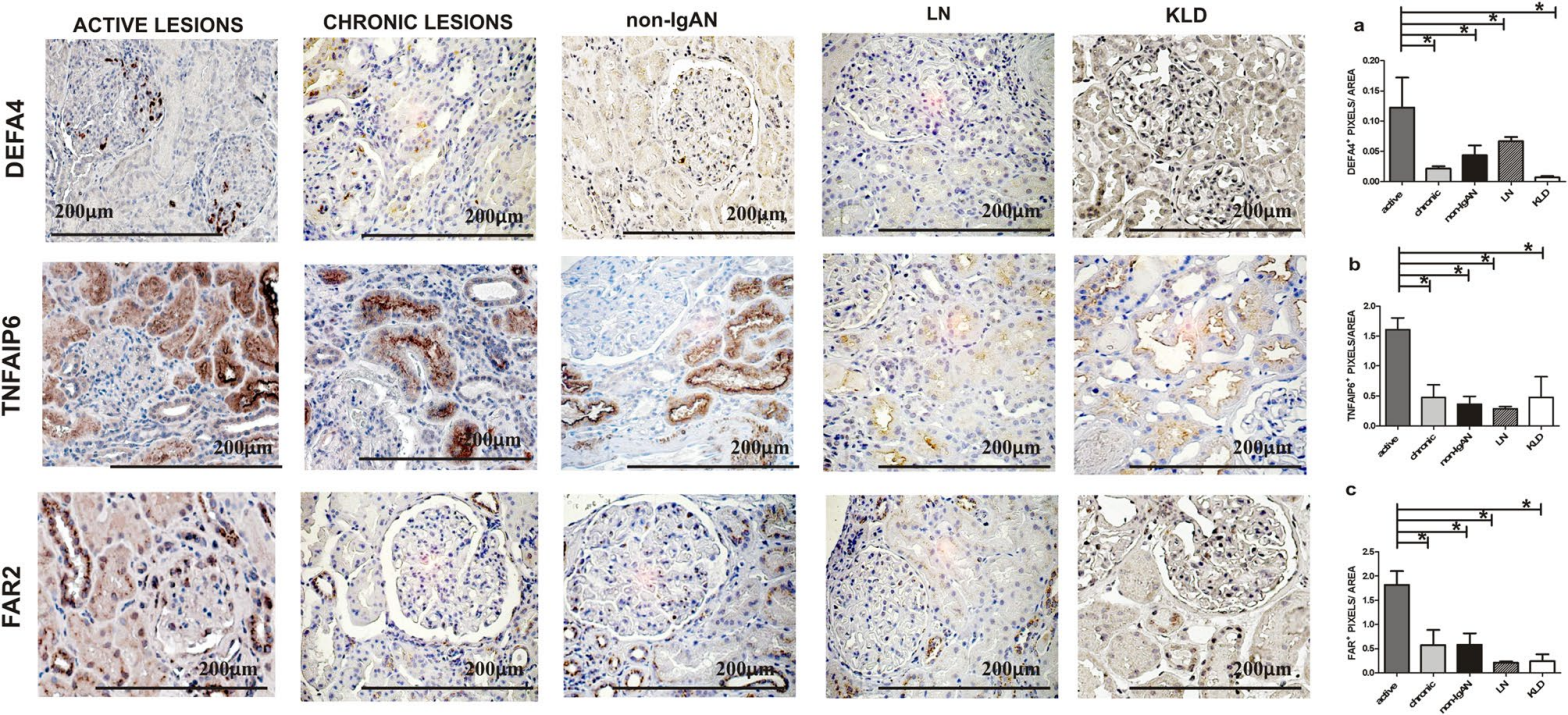
DEFA4, TNFAIP6 and FAR2 protein expression levels in IgAN with active renal lesions. (**A**) Immunohistochemical analysis for DEFA4 shows a positive glomerular expression compared to other groups (n = 4 active group; n = 4 chronic; n = 4 non IgAN; n = 6 LN; n = 4 KLD). Kruskal–Wallis test *p* = 0.0131. Dunn’s multiple comparison test, active versus chronic, active versus non-IgAN, active versus LN and active versus KLD, all *p* < 0.05. (**B**) A strong TNFAIP6 expression has been found in the active lesion group compared to the other groups (n = 6 active group; n = 4 chronic; n = 4 non IgAN; n = 6 LN; n = 4 KLD). Kruskal–Wallis test *p* = 0.0216. Dunn’s multiple comparison test, active versus chronic, active versus non-IgAN, active versus LN and active versus KLD, all *p* < 0.05. (**C**) A strong FAR2 expression has been found in the active lesion group compared to the other groups (n = 4 active group; n = 4 chronic; n = 4 non IgAN; n = 6 LN; n = 4 KLD). Kruskal–Wallis test *p* = 0.0079. Dunn’s multiple comparison test, active versus chronic, active versus non-IgAN, active versus LN and active versus KLD, all *p* < 0.05. Magnification 40× .

Microarray data and RT-PCR showed an up-regulation of DEFA4, TNFAIP6 and FAR2 transcripts in the active renal lesion group. Here, we decided to study their protein expression and localization in biopsy specimens from the active renal lesions group, the chronic renal lesions group, non-IgAN and KLD, the same used for microarray analysis (Table 1A). Furthermore, a control group with a pathologic diagnosis of active proliferative LN (Classes III/IV) was added for IHC analysis to confirm that identified proteins were specific to active IgAN (Table 1B). IHC staining was evaluated on the whole biopsy section without making differences between glomerular ad tubular staining, in line with how gene expression data was obtained. DEFA4 protein expression localized in the glomeruli was up-regulated in the active lesion group and the differences between groups were statistically significant (Kruskal–Wallis test *p* = 0.0131. Figure 4A). TNFAIP6 protein expression was up-regulated in glomerular and tubular cells (Kruskal–Wallis test *p* = 0.0216, Fig. 4B). The FAR2 perinuclear protein expression was found up-regulated in both glomerular and tubular cells (Kruskal–Wallis test *p* = 0.0079 Fig. 4C).

### Renal tissue LTB, CXCL6 and ITGAX protein expression levels in the chronic renal lesion group

**Figure 5:**
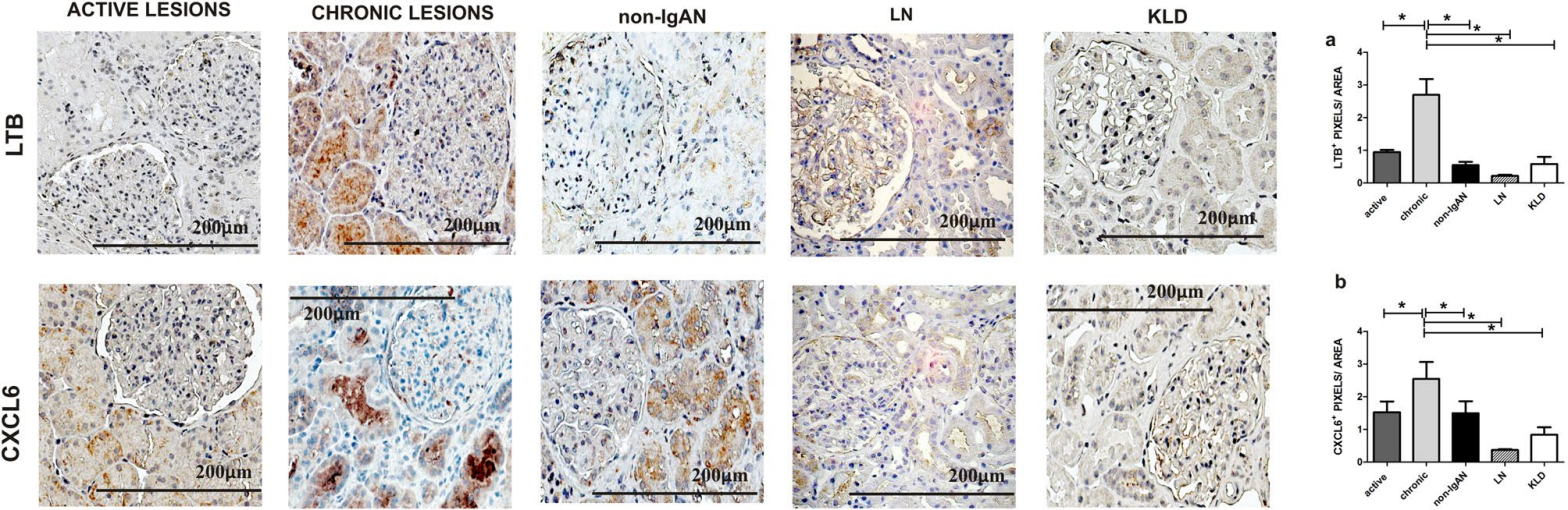
LTB and CXCL6 protein expression levels in IgAN with chronic renal lesions. (**A**) A strong LTB tubular expression has been found in the chronic lesion group compared to the other groups (n = 4 active group; n = 5 chronic; n = 4 non IgAN; n = 6 LN; n = 4 KLD). Kruskal–Wallis test *p* value = 0.0038. Dunn’s multiple comparison test, chronic versus active, chronic versus non-IgAN, chronic versus LN and chronic versus KLD, all *p* < 0.05. (**B**) A strong CXCL6 tubular expression has been found in the active lesion group compared to the other groups (n = 5 active group; n = 5 chronic; n = 5 non-IgAN; n = 6 LN; n = 4 KLD). Kruskal–Wallis test *p* = 0.0074. Dunn’s multiple comparison test, chronic versus active, chronic versus non-IgAN, chronic versus LN and chronic versus KLD, all *p* < 0.05. Magnification 40 × .

Then, we evaluated the LTB*,* CXCL6 *and* ITGAX protein expression pattern in IgAN patients with chronic renal lesions compared to the other groups. LTB and CXCL6 protein expressions were localized in both glomeruli and tubules and a statistically significant increase was found in the chronic renal lesion group (Kruskal–Wallis test *p* = 0.0038, *p* = 0.0074, Fig. 5A,B respectively). The differences in ITGAX protein expression between groups were not statistically significant thus this protein was not further investigated (data not shown).

### TNFAIP6, DEFA4, CXCL6 and LTB urinary levels in IgAN

**Figure 6:**
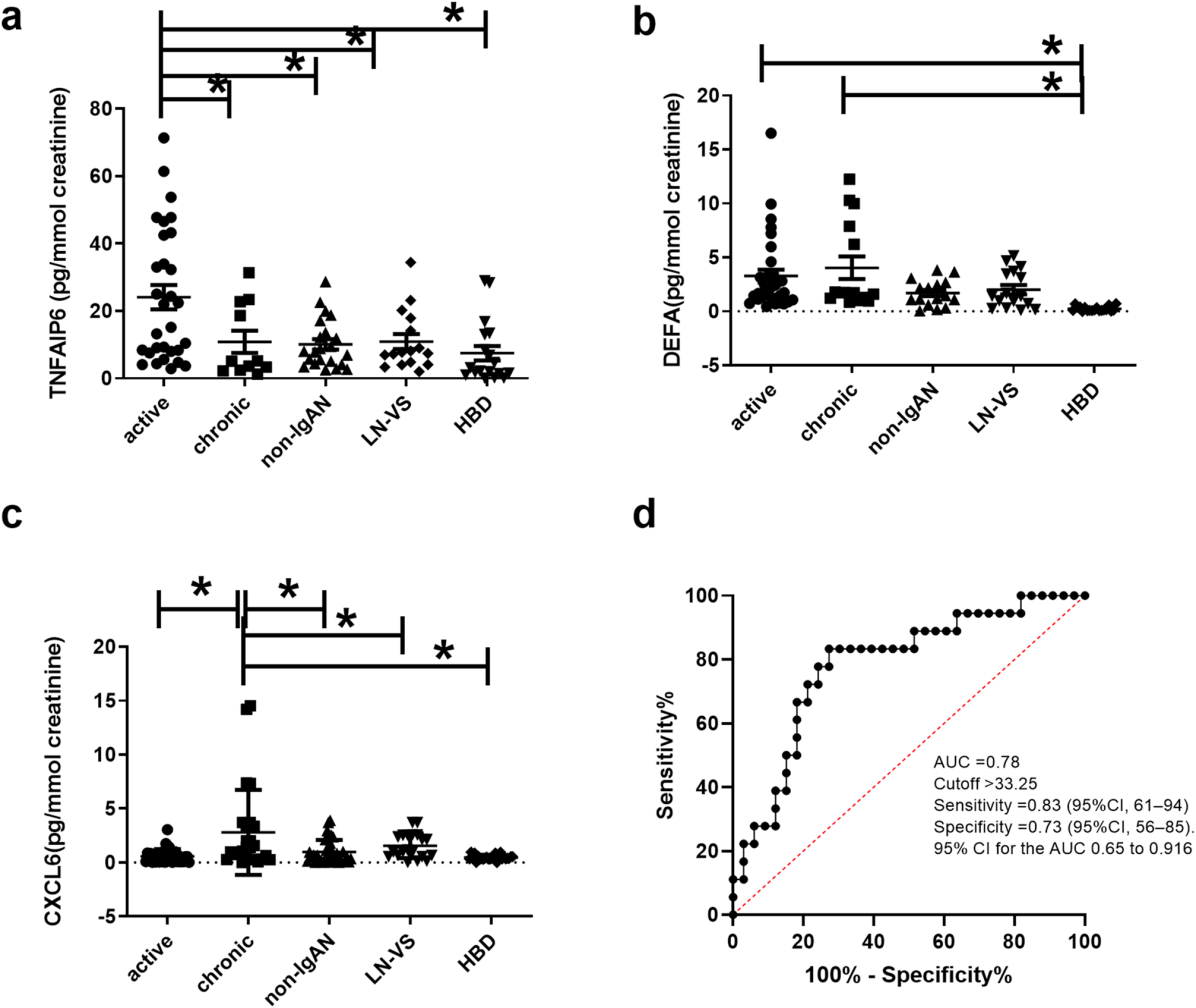
TNFAIP6, DEFA4, and CXCL6 urinary levels in IgAN. (**A**) Urinary TNFAIP6 levels are significantly higher in IgAN patients with active renal lesions compared to other groups (n = 34 active group, n = 17 chronic, n = 23 non-IgAN, n = 17 LN and VS and n = 14 HBD). ANOVA F(4,94) = 6.246 *p* = 0.0002; Tukey’s multiple comparison test, active versus all *p* < 0.05. (**B**) High urinary DEFA4 levels are present in both active and chronic renal lesion groups, and these levels are significantly higher compared to non-IgAN, LN-VS and HBD (n = 34 active group, n = 17 chronic, n = 23 non-IgAN, n = 17 LN and VS and n = 14 HBD. ANOVA F(4,94) = 5.759 *p* = 0.0003; Tukey’s multiple comparison test, active versus and chronic versus HBD *p* < 0.05. (**C**) Urinary CXCL6 levels are significantly higher in IgAN patients with chronic renal lesions compared to the other groups (n = 34 active group, n = 17 chronic, n = 23 non IgAN, n = 17 LN and VS and n = 14 HBD. Kruskal–Wallis test *p* value = 0.0002. Dunn’s multiple comparison test, chronic versus all groups *p* < 0.05, (**D**) TNFAIP6/CXCL6 ratio is able to predict patients characterized by active or chronic renal lesions using a receiver operating characteristic curve (ROC) analysis. The area under the curve (AUC) was 0.78 (n = 33 active, n = 17 chronic as controls, 95% confidence interval 0.648–0.9156, *p* = 0.0009), a cut-off value of 33.25 had the highest sensitivity 83.33% (95% CI 61–94) and specificity 73% (95% CI 56–85).

Our next aim was to evaluate if the up-regulated proteins (TNFAIP6, DEFA4, CXCL6 and LTB) located by IPA in the extracellular space (see RED ARROWS Fig. 2A,B) were also detectable in the urine and if the statistically significant expression differences were also maintained in this biological fluid. For this aim, urinary samples were collected from an independent test cohort of 51 IgAN patients with active (n = 34) and chronic renal lesions (n = 17). These patients were selected with the same histological criteria used to define FFPE active (E1 and/or C1, C2, T0) and chronic group (T1 or T2, E0, C0). Furthermore, we collected non-IgAN (MN (n = 4), MCD (n = 4), hypertensive diseases or diabetic nephropathy (n = 15), LN (n = 11), VS (n = 6), and HBD (n = 14) (Table 1C). The urinary TNFAIP6 level was found significantly higher in IgAN patients with active renal lesions compared to the other groups (ANOVA F(4,43) = 6.246 *p* = 0.0002, Fig. 6A). DEFA4 soluble protein was found in both active and chronic renal lesion group, possibly due to further epithelial cell alpha-defensin secretion in the urinary tract^43^, however, the protein levels were significantly higher in IgAN patients compared to the other groups (ANOVA F(4,94) = 5.759 *p* = 0.0003. Tukey’s multiple comparison test, active and chronic versus non-IgAN, LN-VS and HBD *p* < 0.05. Figure 6B). The urinary CXCL6 level was found significantly higher in IgAN patients with chronic renal lesions compared to the other groups, Kruskal–Wallis test *p* value = 0.0002. Dunn’s multiple comparison test, chronic versus all groups *p* < 0.05 (Fig. 6C). The soluble form of LTB was undetectable in the urine. Next, we tested the ability of the TNFAIP6/CXCL6 ratio to predict patients characterized by active or chronic renal lesions using a receiver operating characteristic curve (ROC) analysis. The area under the curve (AUC) was 0.78 (n = 33 active, n = 17 chronic, 95% confidence interval 0.648–0.9156, *p* = 0.0009), a cut-off value of 33.25 had the highest sensitivity 83.33% (95% CI 61–94) and specificity 73% (95% CI 56–85) (Fig. 6D).

## Discussion

We applied transcriptomics to identify genes that characterize active (endocapillary and extracapillary proliferations) and chronic (tubulo-interstitial) renal lesions using underexploited FFPE biopsy specimens from IgAN patients. We identified distinct molecular pathways involved in different types of kidney damage and found specific gene expression changes in active renal lesions that may be more responsive to immunosuppressive therapy. Bioinformatic analysis identified specific transcripts for active (*DEFA4, TNFAIP6, FAR2*) and chronic (*LTB, CXCL6, ITGAX*) renal lesions and validated them with RT-PCR and IHC. Finally, TNFAIP6 and CXCL6 were confirmed in the urine of an independent cohort of IgAN patients and suggest a potential predictive value of the TNFAIP6/CXCL6 ratio for disease activity in IgAN patients.

Three innovative aspects have been adopted in this study. The first one is related to the nature of the sample material used. We extracted RNA from all archival FFPE biopsy specimens collected in the multicentre study and the results were similar to those found by others in terms of quality (Supplementary Table 1)^2,5,7^. Reports show the feasibility of using FFPE renal tissue as a source for isolating RNA for global gene expression profiling (Supplementary Table 8)^1–7,9^. In particular, we used the RNeasy Kit that has been demonstrated to be the best in terms of RNA purity^2^, and we used a reliable WG-DASL assay specifically designed for genome-wide expression profiling of archived material^44^, but several other commercially available kits and platforms are available for this scope^2^. This recently validated approach could be applied in the near future as vast numbers of FFPE specimens are routinely collected and constitute an extensive repository of tissue material with a long-term clinical follow-up, providing a valuable resource for clinical research^8,45,46^.

The second aspect is related to the use of the renal cortex as a whole, methodology that has already been applied to other glomerulonephritis^47^. Previous transcriptomic studies were performed on microdissected compartments^17–20^, here we wanted to give a global view of the renal cortex as the histologic pattern is scored considering the simultaneous occurrence of glomerular and tubular lesions. We focalized our attention on mediators that are secreted in the extracellular space (Fig. 2A,B) so that they could be used as new measurable urinary biomarkers, able to give an indirect screenshot of disease activity at the renal level.

The third aspect is related to the statistical procedure used. Previous microarray gene expression studies on biopsy specimens^17,18,20,34^ simply compared the IgAN transcriptome with normal renal tissue. Here, we were able to exclude the transcripts that were common to other non-IgAN because they were automatically filtered out by selecting specific gene sets from the VENN diagram. Specifically, when we compared 471 genes that were common to other types of non-IgAN in the central part of the VENN diagram, (Fig. 1A) we found that 16% and 35% of differently expressed genes overlapped with those identified by other expression studies^17,34^ (Supplementary Table 5). This comparison with previous works on IgAN kidney biopsy specimens also highlights a high degree of gene expression concordance between fresh and FFPE tissues.

Some genes presented in this work have already been described for IgAN. Copy number variations of the DEFA locus, are strongly associated with susceptibility to and progression of the disease^48^. Furthermore, both ITGAX and DEFA4 have been associated with IgAN using a completely different approach through the Genome Wide Association Study (GWAS) recognizing several susceptibility loci^35–38^. Among these we find ITGAX and DEFA4. GWASs pinpoint several common single nucleotide polymorphisms associated with IgAN and these have been validated on a large number of IgAN patients^49^. In this context, ITGAX and DEFA4 loci could represent an expression quantitative trait loci (eQTLs), conferring a direct genetic explanation of the aberrant gene expressions found in our IgAN patients.

The most significant network constructed with differently expressed genes in active renal lesions highlighted two secreted components in the extracellular space *DEFA4*, *TNFAIP6*. Defensins are a family of antimicrobial and cytotoxic peptides thought to be involved in host defence^50^. They are abundant in neutrophil granules but can also be found in mucosal epithelial surfaces such as those of the intestine, respiratory tract and urinary tract. Specifically, we demonstrated high gene and protein expression levels of DEFA4, also known as human neutrophil peptide-4, in kidney biopsies of IgAN patients with active renal lesions; neutrophils were present in 50% of these biopsies and future studies will need to confirm their involvement in this condition^51–54^. DEFA4 urinary levels were statistically higher in both IgAN groups compared to all other groups. On the other hand, the differences found in DEFA4 renal expression levels between active and chronic lesions in IgAN patients were not maintained in the urine, this could be explained by additional defensin secretion in bladder urothelium and ureter, as seen with other defensins^55^.

*TNFAIP6,* also known as *TSG-6,* is a multifunctional protein associated with inflammation up-regulated by pro-inflammatory mediators in neutrophils, monocytes and endothelial cells. This protein may be involved in crescents formation and endocapillary proliferation as TNFAIP6 has been demonstrated to participate in pro-fibrotic phenotype and tissue remodelling. The selective silencing of this gene improves survival of kidney epithelial cells in vitro models of oxygen and glucose deprivation^56–58^; furthermore, it is involved in the interaction between hyaluronan and CD44, both targets for nephroprotection^59–62^. *FAR2* is strongly associated with kidney disease and a strong expression was found in the IgAN group characterized by active renal lesions. Low intensity expression was found in chronic lesions group and in other non-IgAN and the expression was totally absent in KLD. This gene was missing in the top network, but we decided to follow-up this gene due to its involvement in kidney disease^31–33^. Furthermore, we added another non-IgAN control group of active proliferative lesions as LN patients (Class III/IV), to confirm that these markers characterising the IgAN active lesions group were not common to other types of active glomerulonephritis.

There is an intriguing discussion on therapy benefits of corticosteroid treatment in IgAN^63–65^. Our most significant network constructed with genes characterizing active renal lesions showed that 18 genes are influenced by the glucocorticoid receptor signalling because they belong to this pathway (Fig. 2A). Specifically, we found various glucocorticoid target genes such as Nuclear factor kB (NFkB) and AKT that are directly connected with the aberrantly modulated genes^66^. These results are supported by other gene expression studies that evaluated differentially expressed transcripts in patients with endocapillary proliferation compared to IgAN without endocapillary lesions^19^. Here we support the hypothesis of the potential reversibility of the E and C lesions after corticosteroid therapy, confirmed by other authors^67^.

Dsyregulated genes in chronic renal lesions generated a statistically significant network where the secreted proteins *LTB* and *CXCL6* were up-regulated. The former gene is involved in renal inflammation, in the communication between lymphocytes and is expressed in activated lymphocytes, dendritic cells, and in tertiary lymphoid tissues^40, 68–70^. We confirm previous reports that describe the LTB staining in tubular epithelial cells^40^, but here we demonstrate that the up-regulation is specifically associated to chronic tubular lesions. *CXCL6*, on the other hand, is expressed in macrophages, epithelial and mesenchymal cells during inflammation^41^ and the expression is strongly associated with fibrosis^71^ and it increases in human renal epithelial cells stimulated with pro-inflammatory cytokines^72^. For the first time, we demonstrate that *CXCL6* gene and protein are up-regulated in the chronic renal lesions and high levels were found in the urine, thus making it a candidate biomarker for monitoring this condition.

There are some limitations in our work. Firstly, we were unable to detect protein urinary levels on the archival group used for the microarray study as urinary samples were not available for this cohort. We bypassed this inconvenience by testing protein concentration in an independent cohort of IgAN patients. Secondly, we were unable to perform correlation analysis between IHC staining levels and urinary biomarker levels because results came from different cohorts. Results obtained from this independent cohort strengthens our results, but future studies conducted on larger cohorts will need to confirm the biological relevance of these proteins and the potential predictive value of TNFAIP6/CXCL6 ratio as a possible urinary biomarker for disease activity and chronicity, as demonstrated by ROC analysis.

In conclusion, our findings demonstrate the feasibility of using an extensively available FFPE repository stored in the pathology laboratories for the identification of molecular phenotypes associated with histomorphological lesions, as currently done for cancer^1,73^. This approach may yield important insights into the molecular pathogenesis of renal lesions in IgAN and give insights into prospective therapeutic strategies. The dual approach applied here, histomorphological study on one hand and molecular mechanisms on the other, may drive towards a finer stratification of IgAN patients.

## Supplementary information

Supplementary Information.
