# Supplementary material for: Formalin-fixed paraffin-embedded renal biopsy tissues: an underexploited biospecimen resource for gene expression profiling in IgA nephropathy
Source: Sci Rep. 2020 Sep 16;10:15164. doi: 10.1038/s41598-020-72026-2 (PMC7494931; doi:10.1038/s41598-020-72026-2)
Supplement: Supplementary file 1 — Supplementary Information. [file 41598_2020_72026_MOESM1_ESM.docx]

**Supplementary Data**

**Formalin-Fixed Paraffin-Embedded Renal Biopsy Tissues: An Underexploited Biospecimen resource for gene expression profiling in IgA Nephropathy**

Sharon Natasha Cox,^1,2*^ Samantha Chiurlia,^1^ Chiara Divella,^2^ Michele Rossini,^2^ Grazia Serino,^3^ Mario Bonomini,^4^ Vittorio Sirolli,^4^ Francesca B Aiello,^4^ Gianluigi Zaza,^5^ Isabella Squarzoni,^5^ Concetta Gangemi,^5^ Maria Stangou,^6^ Aikaterini Papagiannii,^6^ Mark Haas,^7^ Francesco Paolo Schena.^1,2*^

^1^Schena Foundation, Research Center of Kidney Diseases, Strada Prov. le Valenzano-Casamassima Km 3, 70100 Valenzano (Bari), Italy

^2^Division of Nephrology, Dialysis, and Transplantation, Department of Emergency and Organ Transplantation, University of Bari, Bari, Italy.

^3^National Institute of Gastroenterology “S. de Bellis”, Research Hospital, 70013, Castellana Grotte (Bari), Italy.

^4^Department of Medicine and Aging Sciences, University "G. D'Annunzio" of Chieti-Pescara, Chieti, Italy.

^5^Renal Unit, Department of Medicine, University-Hospital of Verona, Verona, Italy

^6^Department of Nephrology, Aristotle University of Thessaloniki, Hippokration General Hospital, Thessaloniki, Greece.

^7^Department of Pathology and Laboratory Medicine, Cedars-Sinai Medical Center, Los Angeles, California. United States.

**
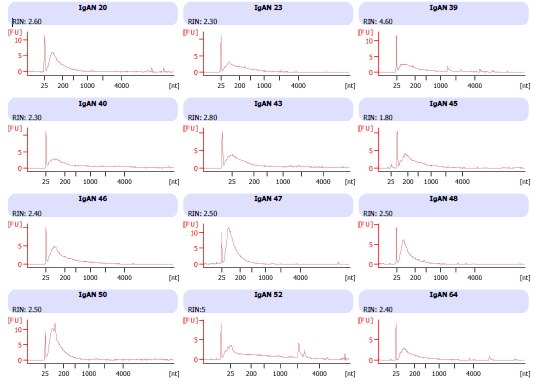
**

**Supplementary_Figure 1-** Representative RNA Integrity Numbers (RIN) that were in the range of 2.1–5, the most abundant RNA fragments were in the range of 100–200 ribonucleotides


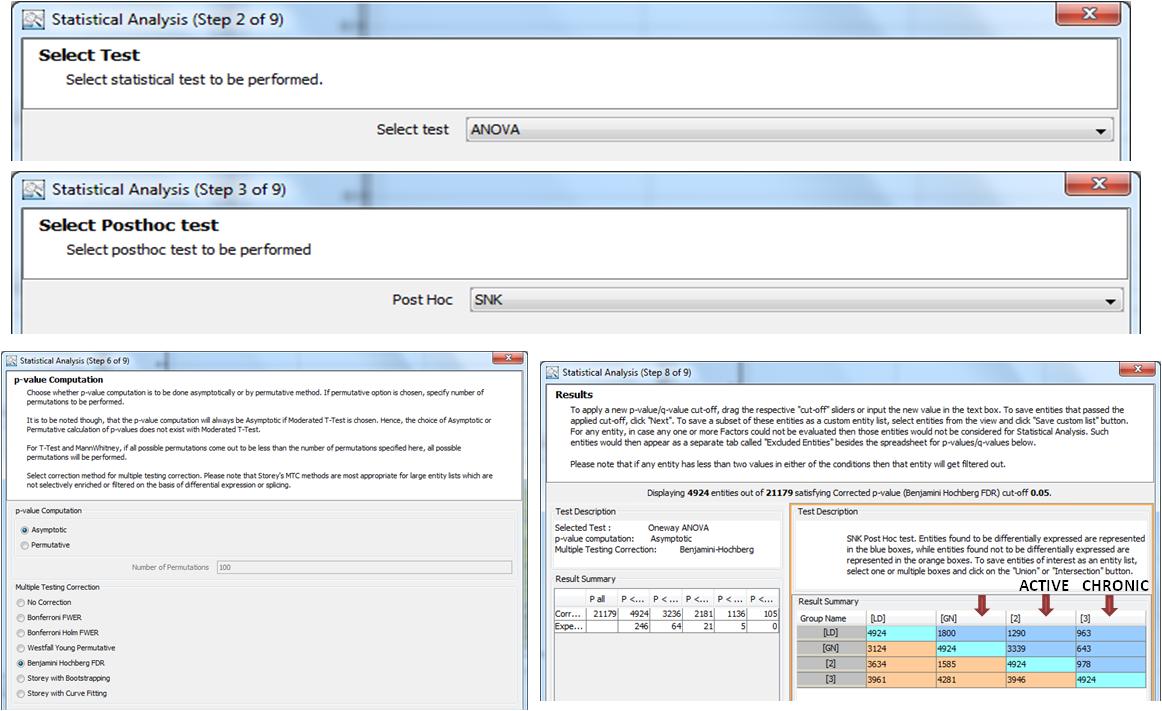


**Non-IgAN**

**Supplementary_Figure 2.** Consecutive screenshots of the statistical analysis performed with GeneSpring software. This statistical analysis generated a gene list of 4924 probes satisfying the corrected p- value(Benjamini Hochberg FDR) cut-off of 0.05. Gene lists for active (1290 probes) and chronic (963 probes) renal lesions, and non-IgAN (1800) were obtained. These probe lists have been used for constructing the VENN diagram(manuscript Figure 1A ) to find specific probes for active and chronic renal lesions


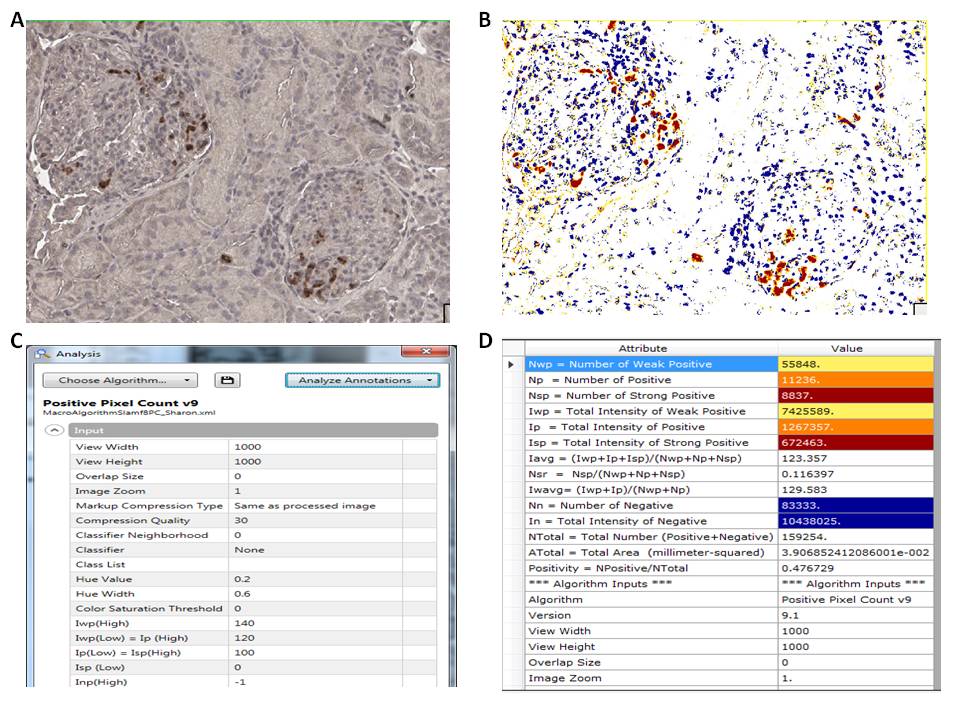


**Supplementary Figure 3.** The Aperio specific ImageScope software with Positive pixel count algorithm was used to measure the staining intensity. For each section, the intensity of the staining with absent (0) to strong (+++) was converted into a number. Only high intensity pixels (identified by the software as strong positive) were considered as a positive staining and were normalized to the selected area (total number of pixels in the section). A)Typical Image at 20x magnification, B) strong positive staining as identified by the algorithm (RED) C) Input settings for algorithm D) Signal staining converted into a numbers, the red number indicates the total intensity of strong positive.

**Supplementary Table 1**: RNA extracted from formalin-fixed paraffin-embedded from different tissues

| **Tissue Type** | **Archived Kidney Biopsy (Time storage, yrs)** | **RNA Quality**  **(RIN)** | **RNA Fragments** | **Authors** |
| --- | --- | --- | --- | --- |
| **Breast Cancer** | 10 | 1.0-6.1 | n.r | **Ribeiro-Silva, A., et al 2007 (Reference 1)** |
| **Prostate Cancer** | 7-15 |  | **150-200** | **Patel, P.G. et al. 2017 (Reference 4)** |
| **Brain** | 4 | 2.1-4.4 |  | **Wimmer, I. et al. 2018 (Reference 7)** |
| **Idiopathic Pulmonary Fibrosis** | 6 | 2.1-2.6 |  | **Vukmirovic, M et al 2017 (Reference 5)** |
| **Colon Cancer** | 4-10 | 2-15 |  | **Bedler, N. et al 2016 (Reference 2)** |
| **Breast Cancer** | 1-10 | 1.6-2.4 |  | **Choi, Y et al 2017(Reference 6)** |
| **Kidney Biopsy** | **2-9** | **2.1-5** | **100-200** | **Current Study** |

RIN:RNA Integrity Number.

**Supplementary Table 2**: 4924 probes satisfied the corrected p-value (Benjamii Hochberg FDR) cutoff of 0.05 when IgAN with active renal lesions, IgAN with chronic lesions and non-IgAN GN were compared with LD group.

| Probe_Id | p (Corr) | p | [active](normalized) | [cronic](normalized) | [GN](normalized) | [LD](normalized) | Symbol |
| --- | --- | --- | --- | --- | --- | --- | --- |
| ILMN_3166640 | 0.004921 | 2.56E-04 | 0.478673 | 0.186576 | -0.28038 | 0.278202 | ERCC-00157 |
| ILMN_3166653 | 0.004191 | 1.77E-04 | 0.506586 | 0.025158 | -0.22109 | 0.322802 | ERCC-00158 |
| ILMN_3165483 | 0.007466 | 6.28E-04 | 0.228791 | 0.035636 | -0.23394 | 0.222291 | ERCC-00062 |
| ILMN_3165648 | 0.005851 | 3.89E-04 | 0.371841 | 0.025412 | -0.19816 | 0.208789 | ERCC-00077 |
| ILMN_3166414 | 0.003538 | 1.03E-04 | 0.776979 | -0.06067 | -0.28195 | 0.253152 | ERCC-00137 |
| ILMN_3166255 | 0.00358 | 1.07E-04 | 0.488336 | 0.070846 | -0.33386 | 0.196968 | ERCC-00126 |
| ILMN_3243894 | 0.025157 | 0.004325 | 0.526685 | -0.35115 | -0.52984 | 0.500014 | SNORA60 |
| ILMN_2121316 | 0.005657 | 3.64E-04 | 0.33848 | -0.00409 | -0.21725 | 0.011024 | SCYL1BP1 |
| ILMN_1704173 | 0.010118 | 0.001055 | 0.389756 | 0.072628 | -0.23104 | 0.130695 | AIFM3 |
| ILMN_1660232 | 0.019848 | 0.003012 | 0.029262 | 0.003553 | 0.160075 | -0.44564 | PEX5 |
| ILMN_1775074 | 0.016564 | 0.00232 | 0.071059 | -1.00679 | 0.094207 | -0.12999 | TUBGCP2 |
| ILMN_1734742 | 0.020429 | 0.003175 | -0.14369 | 0.123441 | 0.344709 | -0.44709 | ARHGDIA |
| ILMN_1704019 | 0.01215 | 0.001429 | 0.288317 | 0.043928 | -0.28321 | 0.089455 | OGFOD2 |
| ILMN_3245759 | 0.016686 | 0.002343 | 0.207303 | -0.08857 | -0.44266 | 0.670332 | SNORD92 |
| ILMN_1710329 | 0.004034 | 1.51E-04 | 0.38095 | -0.03085 | -0.41083 | 0.127854 | MYEF2 |
| ILMN_3238183 | 1.07E-04 | 1.21E-07 | -0.38312 | 0.016871 | 0.514172 | -0.93449 | BMS1P5 |
| ILMN_2057836 | 0.001853 | 1.84E-05 | 0.427731 | -0.10968 | -0.35831 | -0.06451 | RNU2-1 |
| ILMN_1797975 | 0.04906 | 0.011341 | 0.097227 | 0.015628 | -0.3976 | -0.09819 | CXCR3 |
| ILMN_1765208 | 0.015209 | 0.002043 | -0.1817 | -0.03788 | -0.43505 | 0.406401 | GLUL |
| ILMN_1656837 | 0.037447 | 0.007667 | -0.12227 | 0.36352 | -0.25327 | -0.43835 | RBP1 |
| ILMN_1657627 | 0.011494 | 0.001306 | 0.058975 | 0.510559 | -0.31625 | -0.01465 | CBFA2T3 |
| ILMN_1751607 | 3.70E-07 | 1.22E-10 | 0.757653 | 1.064673 | -0.15155 | 4.38566 | FOSB |
| ILMN_1723113 | 0.011107 | 0.001236 | 0.29932 | 0.020707 | -0.30798 | 0.002365 | WFDC13 |
| ILMN_1668847 | 0.010049 | 0.001038 | 0.282087 | -0.13189 | -0.03121 | 1.080167 | WWP2 |
| ILMN_2157843 | 0.006356 | 4.59E-04 | 0.781395 | 0.078995 | -0.29042 | 0.524509 | ZNF829 |
| ILMN_2221768 | 0.014381 | 0.001871 | 0.516438 | 0.229612 | -0.24287 | 0.173694 | PRAMEF4 |
| ILMN_3238814 | 0.029555 | 0.005502 | -0.21125 | -0.30499 | 0.265725 | -0.31139 | LOC100170939 |
| ILMN_1741727 | 0.045519 | 0.010155 | -0.14883 | -0.13072 | 0.058127 | -1.28404 | QPCT |
| ILMN_1774071 | 0.004651 | 2.29E-04 | 0.453842 | 0.077223 | -0.16786 | 0.182285 | CD177 |
| ILMN_1782247 | 0.019278 | 0.002889 | 0.298202 | 0.070866 | -0.20628 | -0.18481 | KAT2A |
| ILMN_1718832 | 0.03503 | 0.007006 | 0.067303 | -0.24572 | 0.185044 | -0.64789 | SPHAR |
| ILMN_1778756 | 0.014546 | 0.001902 | 0.36446 | 0.023288 | -0.18436 | 0.080206 | SULT1A1 |
| ILMN_2409720 | 0.036023 | 0.007272 | -0.22598 | 0.419618 | 0.239819 | -1.02543 | SLA2 |
| ILMN_1742889 | 0.008833 | 8.45E-04 | 0.147521 | -0.0591 | -0.32549 | 0.033958 | WDR77 |
| ILMN_2398039 | 0.049954 | 0.011602 | -0.2276 | 0.05317 | -0.22035 | 0.461717 | TCERG1 |
| ILMN_2156250 | 0.008598 | 7.99E-04 | 0.310202 | -0.0176 | -0.26733 | 0.248073 | SOCS3 |
| ILMN_1706553 | 0.03621 | 0.00733 | 0.24191 | 0.190368 | -0.17213 | 0.178535 | SMG7 |
| ILMN_1783448 | 0.018123 | 0.002643 | 0.22913 | 0.1151 | -0.11013 | -0.1106 | DYNC1LI2 |
| ILMN_1776480 | 0.041355 | 0.008847 | -0.25844 | 0.050371 | 0.311207 | -0.82535 | HINFP |
| ILMN_1809676 | 0.005556 | 3.50E-04 | 0.589106 | 0.108582 | -0.11927 | 0.312097 | CACNG6 |
| ILMN_1807972 | 0.015254 | 0.002056 | -0.67503 | -0.06058 | 0.058079 | -1.47455 | MICAL1 |
| ILMN_1655625 | 0.003387 | 9.00E-05 | 0.299142 | -0.03475 | -0.23431 | 0.121427 | GPATCH1 |
| ILMN_1668592 | 0.013731 | 0.001731 | 0.395052 | -0.08889 | 0.785439 | -0.21872 | STON1 |
| ILMN_2359500 | 0.004544 | 2.17E-04 | 0.381404 | 0.023379 | -0.28335 | 0.092093 | NCDN |
| ILMN_1765532 | 0.035576 | 0.007149 | 0.201282 | 0.136797 | -0.12896 | 0.265016 | RDBP |
| ILMN_2388547 | 0.041861 | 0.009021 | -0.47816 | -0.62604 | 0.094521 | 0.160678 | EPSTI1 |
| ILMN_1681591 | 0.007468 | 6.29E-04 | 0.166198 | -0.17402 | -0.3343 | 0.2459 | PTPN1 |
| ILMN_2343677 | 0.004543 | 2.15E-04 | 0.406441 | -0.23523 | -0.34605 | 0.059278 | SOX5 |
| ILMN_1760051 | 0.041128 | 0.008779 | 0.893981 | 0.180497 | -0.21883 | 0.456506 | SOX5 |
| ILMN_1670707 | 0.007726 | 6.68E-04 | 0.49725 | -0.04444 | -0.30356 | 0.139024 | C17orf46 |
| ILMN_1730995 | 0.040215 | 0.008476 | -0.1231 | -0.22268 | 0.232195 | -0.53973 | AFAP1L2 |
| ILMN_2412807 | 0.002851 | 5.22E-05 | 0.338819 | 0.129589 | -0.2635 | 0.004429 | DCTN1 |
| ILMN_1761409 | 0.009588 | 9.63E-04 | -0.16521 | 0.263948 | 0.118657 | -0.85141 | ZNF167 |
| ILMN_1676413 | 0.045237 | 0.010077 | 0.836118 | -0.35947 | 0.227945 | -0.03964 | VSNL1 |
| ILMN_1774074 | 0.034085 | 0.006736 | 0.176307 | 0.147923 | -0.23194 | -0.05056 | RXRB |
| ILMN_1729294 | 0.008217 | 7.41E-04 | 0.241153 | -0.12948 | -0.12346 | -0.11121 | RNF130 |
| ILMN_1759679 | 0.004866 | 2.48E-04 | 0.392317 | 0.080155 | -0.32892 | 0.098729 | FAM81B |
| ILMN_3245599 | 0.006726 | 5.26E-04 | 0.611581 | -0.3254 | -0.11068 | 0.48339 | SNORD109A |
| ILMN_1674588 | 0.036061 | 0.007284 | 0.340865 | 0.410266 | -0.13601 | -0.0236 | HAO1 |
| ILMN_1756455 | 0.010638 | 0.001148 | 0.333587 | 0.095839 | -0.30247 | 0.066515 | IL5RA |
| ILMN_3239168 | 0.003917 | 1.39E-04 | 0.479061 | 0.13535 | -0.23112 | 0.267306 | LOC645332 |
| ILMN_1683149 | 0.037299 | 0.007612 | 0.222315 | -0.15817 | -0.11754 | 0.214254 | LOC388882 |
| ILMN_1775743 | 0.021203 | 0.003344 | 0.239528 | 0.158688 | -0.11081 | 0.134559 | BTG1 |
| ILMN_1695763 | 0.033642 | 0.006612 | 0.421056 | -0.47494 | 0.051119 | 0.355762 | PDIA5 |
| ILMN_2136133 | 0.041845 | 0.009014 | 0.146572 | 0.044676 | -0.20406 | -0.26541 | PABPC1 |
| ILMN_2358265 | 0.032937 | 0.006412 | 0.298723 | 0.152363 | -0.19359 | 0.275553 | F7 |
| ILMN_2267025 | 0.005334 | 3.21E-04 | 0.425579 | -0.009 | -0.21435 | 0.123127 | F7 |
| ILMN_1737036 | 0.004255 | 1.89E-04 | 0.327915 | 0.085406 | -0.31283 | 0.014151 | GGT7 |
| ILMN_2107068 | 0.031169 | 0.005935 | -0.01933 | -0.04488 | 0.192067 | -0.353 | HOXA2 |
| ILMN_1759252 | 0.014615 | 0.001915 | 0.27412 | 0.062491 | -0.18655 | -0.0677 | ADD1 |
| ILMN_2245627 | 0.022322 | 0.003637 | 0.278621 | 0.312798 | -0.26475 | 0.232423 | DRGX |
| ILMN_1798870 | 0.019653 | 0.002965 | 0.32541 | -0.01767 | -0.24419 | 0.084693 | RBPJL |
| ILMN_1814600 | 0.02336 | 0.00389 | -0.05634 | -0.72559 | 0.027792 | 1.193514 | DEPDC1B |
| ILMN_1698849 | 0.034354 | 0.006816 | 0.192719 | 0.222358 | -0.26451 | 0.137052 | CHRNA2 |
| ILMN_1722634 | 0.012553 | 0.00151 | 0.302236 | -0.01584 | -0.27992 | -7.82E-04 | NUCB1 |
| ILMN_2117171 | 0.021324 | 0.00337 | 0.181249 | 0.111672 | -0.22395 | -0.25497 | LMO4 |
| ILMN_1812163 | 0.006731 | 5.28E-04 | 0.192083 | 0.023222 | -0.32913 | 0.145332 | GYPE |
| ILMN_3237488 | 0.002842 | 5.10E-05 | 0.429566 | 0.064271 | -0.29615 | 0.250058 | AKR1C2 |
| ILMN_1713249 | 0.026757 | 0.004734 | 0.132306 | 0.014236 | -0.27432 | -0.08421 | PHF19 |
| ILMN_1801842 | 0.002962 | 5.78E-05 | 0.549027 | 0.059534 | -0.29723 | 0.853341 | PTX3 |
| ILMN_1697493 | 0.033999 | 0.006712 | -0.35656 | -0.36995 | 0.174389 | -0.47669 | WDFY3 |
| ILMN_2131926 | 0.006957 | 5.58E-04 | 0.330833 | -0.01196 | -0.2765 | 0.108974 | ZNF718 |
| ILMN_1809964 | 0.005595 | 3.56E-04 | 0.346522 | 0.103259 | -0.32428 | 0.175747 | OR10J1 |
| ILMN_1754009 | 0.007032 | 5.67E-04 | 0.512207 | 0.171712 | -0.22888 | 0.127536 | KRTAP10-12 |
| ILMN_2125747 | 0.031354 | 0.00599 | -0.9008 | 0.725711 | -0.31448 | -0.22388 | LOC606724 |
| ILMN_1693519 | 0.007172 | 5.89E-04 | 0.022082 | 0.157064 | -0.24455 | 0.178424 | PRKG1 |
| ILMN_1726901 | 0.017074 | 0.002422 | 0.136931 | 0.187539 | -0.23344 | 0.040706 | KLC1 |
| ILMN_1718253 | 0.032237 | 0.006226 | 0.695626 | 0.119094 | -0.27009 | 0.133387 | TAS2R3 |
| ILMN_3234751 | 0.019116 | 0.002856 | 0.585997 | -0.39795 | -0.41711 | 0.519901 | SNORD103A |
| ILMN_3301818 | 0.002985 | 5.92E-05 | -0.70593 | -0.19097 | 0.35852 | -0.83262 | ST20 |
| ILMN_2316878 | 0.004474 | 2.08E-04 | 0.181693 | -0.3165 | 0.584766 | 0.698771 | PTPRO |
| ILMN_2289473 | 0.015193 | 0.002038 | 0.265825 | 0.054083 | -0.25225 | 0.090177 | TEPP |
| ILMN_2398294 | 0.003641 | 1.15E-04 | 0.132717 | 0.013727 | -0.39567 | 0.108103 | TEPP |
| ILMN_1799848 | 0.038762 | 0.008024 | 0.466308 | 0.019209 | -0.23495 | -0.249 | ANKRD22 |
| ILMN_2319000 | 0.034999 | 0.006997 | -0.1444 | 0.381103 | -0.35007 | -0.06851 | MATK |
| ILMN_3247543 | 0.005706 | 3.70E-04 | 0.35906 | 0.027948 | -0.28387 | 0.03532 | ELP2P |
| ILMN_1665397 | 0.027058 | 0.004797 | 0.312931 | -0.04011 | -0.20609 | 0.00973 | ZNF445 |
| ILMN_2162860 | 0.033124 | 0.006467 | 0.220379 | -0.09663 | -0.26528 | 0.094575 | SLFN11 |
| ILMN_3226904 | 0.020411 | 0.003167 | 0.307562 | 0.006579 | -0.23183 | 0.306099 | NOP2 |
| ILMN_2361400 | 0.021784 | 0.003475 | 0.454587 | -0.09427 | -0.19765 | 0.109339 | ABCA12 |
| ILMN_1813775 | 0.003641 | 1.16E-04 | 0.124038 | -0.01888 | -0.30407 | 0.266104 | GAK |
| ILMN_2376050 | 0.016782 | 0.002365 | 0.430798 | 0.412864 | -0.38424 | -0.18049 | FXYD3 |
| ILMN_1657701 | 0.006999 | 5.62E-04 | 0.198184 | 0.093758 | -0.28733 | 0.07585 | TMEM137 |
| ILMN_1767747 | 0.025924 | 0.004536 | 0.196611 | 0.005014 | -0.26283 | 0.104659 | HDAC2 |
| ILMN_1679428 | 0.006946 | 5.55E-04 | -0.05602 | 0.696716 | 0.461865 | -0.22652 | CHIC2 |
| ILMN_1704765 | 0.03257 | 0.006322 | 0.273707 | -0.20665 | -0.22929 | -0.11306 | ZSCAN22 |
| ILMN_1691916 | 0.004969 | 2.63E-04 | -0.12043 | 0.138983 | 0.127569 | -0.63384 | KIAA1919 |
| ILMN_2187071 | 0.003944 | 1.42E-04 | 0.480664 | 0.05585 | -0.19411 | 0.114703 | C1orf201 |
| ILMN_3309064 | 0.013578 | 0.001703 | 0.365796 | 0.076565 | -0.21597 | -0.19096 | MIR10A |
| ILMN_1748182 | 0.033736 | 0.006637 | 0.222129 | 0.154315 | -0.2712 | 0.125421 | SRY |
| ILMN_2133799 | 0.005046 | 2.76E-04 | 0.33682 | -0.03281 | -0.31982 | 0.05438 | ACAT2 |
| ILMN_2390099 | 0.002418 | 3.37E-05 | 0.430391 | -0.02115 | -0.26658 | 0.25071 | COL9A1 |
| ILMN_1763398 | 0.005638 | 3.62E-04 | 0.357813 | 0.077707 | -0.29918 | 0.092598 | CLEC17A |
| ILMN_2414878 | 0.035315 | 0.007088 | -0.29516 | -0.4803 | 0.241149 | 0.475129 | STXBP1 |
| ILMN_1714121 | 0.037214 | 0.007586 | 0.54663 | 0.056314 | -0.08736 | -0.01933 | PRMT8 |
| ILMN_2054928 | 0.001835 | 1.77E-05 | -0.57629 | -0.03734 | 0.201503 | -0.9642 | XPNPEP3 |
| ILMN_1732860 | 0.019672 | 0.00297 | 0.419082 | -0.00224 | -0.20444 | 0.135233 | FLJ26850 |
| ILMN_1737635 | 0.040991 | 0.008729 | 0.186215 | -0.01468 | -0.2355 | -0.12142 | RAD1 |
| ILMN_1813019 | 0.010504 | 0.001123 | 0.364367 | 0.046185 | -0.20111 | -0.07793 | DNAJB4 |
| ILMN_1731326 | 0.033472 | 0.006556 | 0.201311 | -0.03119 | -0.11447 | 0.135236 | FLJ46210 |
| ILMN_1725534 | 0.008246 | 7.48E-04 | 0.317756 | 0.035739 | -0.17895 | -0.0121 | ACTN4 |
| ILMN_1789500 | 0.01381 | 0.001748 | 0.312063 | 0.157451 | -0.40892 | -0.00934 | KIAA1875 |
| ILMN_1789196 | 0.044225 | 0.009757 | 0.370415 | 0.129322 | -0.07623 | -0.0141 | TPM2 |
| ILMN_1754538 | 0.014686 | 0.001928 | -0.22171 | -0.03494 | 0.395126 | -0.40758 | C10orf58 |
| ILMN_1797731 | 0.003753 | 1.24E-04 | -0.43713 | 0.403561 | -0.02427 | -0.8188 | MS4A6A |
| ILMN_1687875 | 0.006387 | 4.64E-04 | 0.518391 | -0.03813 | -0.32432 | 0.286168 | UNC5C |
| ILMN_1725510 | 0.004927 | 2.57E-04 | 0.299843 | 0.014974 | -0.26169 | -0.03015 | DHCR24 |
| ILMN_1725145 | 0.005266 | 3.14E-04 | 0.25122 | 0.063243 | -0.38784 | -0.02559 | POLA |
| ILMN_2180239 | 0.028652 | 0.005248 | 0.050541 | 0.123763 | -0.3104 | 0.009608 | DOPEY2 |
| ILMN_1687315 | 0.017881 | 0.002598 | 0.185903 | -0.44466 | -0.05608 | -0.13755 | RXRA |
| ILMN_1688633 | 0.037299 | 0.007611 | 0.164173 | -0.07759 | -0.38993 | 0.314417 | CLCN2 |
| ILMN_1721344 | 0.013904 | 0.00177 | 0.058954 | 0.031996 | -0.35084 | 0.006 | MOBKL2A |
| ILMN_1815686 | 0.002919 | 5.64E-05 | 0.323096 | 0.104929 | -0.29729 | 0.161995 | EGLN2 |
| ILMN_1747067 | 0.00379 | 1.27E-04 | 0.454827 | 0.193405 | -0.42963 | 0.36298 | NPAS1 |
| ILMN_1699844 | 0.037854 | 0.007773 | -0.1832 | -0.26271 | 0.280387 | -0.80993 | SCRN3 |
| ILMN_1658909 | 0.029521 | 0.00549 | 0.230333 | 0.062541 | -0.28656 | 0.01025 | OSGEPL1 |
| ILMN_2361104 | 0.011611 | 0.001335 | -0.06266 | 0.175417 | 0.216773 | -0.51248 | ZMAT3 |
| ILMN_3248575 | 5.74E-04 | 2.04E-06 | 0.664196 | -0.11581 | -0.49238 | 0.282752 | SNORA42 |
| ILMN_3309699 | 0.006484 | 4.82E-04 | 0.314398 | 0.252266 | -0.23879 | 0.190763 | MIR29B1 |
| ILMN_2399896 | 0.00898 | 8.73E-04 | 0.268801 | -0.00992 | -0.15767 | 0.013925 | SEC31A |
| ILMN_2189993 | 0.029103 | 0.005374 | -0.09162 | -0.29713 | 0.277125 | -0.21116 | MRPS35 |
| ILMN_1756289 | 0.001091 | 6.03E-06 | 0.333329 | -0.06309 | -0.374 | 0.310226 | PPP1R12B |
| ILMN_1807919 | 0.037761 | 0.00774 | -0.09771 | -0.43933 | 0.130839 | -0.29643 | TNS1 |
| ILMN_1781764 | 0.00716 | 5.84E-04 | 0.214793 | -0.0237 | -0.17253 | 0.334954 | HNRNPH2 |
| ILMN_1791902 | 0.034085 | 0.006739 | -0.32247 | 0.098111 | -0.19701 | 0.824882 | RARA |
| ILMN_1696683 | 0.049005 | 0.011324 | 0.21718 | 0.045828 | -0.29674 | -0.11665 | THRB |
| ILMN_1792305 | 0.034889 | 0.00696 | 0.04298 | 0.019909 | 0.168307 | -0.60325 | ZNF318 |
| ILMN_3235046 | 0.007602 | 6.48E-04 | 0.297008 | 0.145268 | -0.26438 | 0.373518 | LOC100132354 |
| ILMN_1657769 | 0.017434 | 0.002501 | 0.364579 | 0.485497 | -0.22627 | 0.382577 | EIF5 |
| ILMN_1701596 | 0.016593 | 0.002327 | 0.181234 | -0.18696 | -0.33688 | 0.11652 | RPS20 |
| ILMN_1759184 | 0.001337 | 9.08E-06 | 0.141191 | 0.203619 | -0.31688 | 0.162472 | C19orf48 |
| ILMN_1694666 | 0.023901 | 0.004007 | 0.598881 | 0.189014 | -0.1654 | 0.00658 | BCL2L15 |
| ILMN_1703346 | 0.004474 | 2.09E-04 | 0.414338 | -0.0211 | -0.24877 | 0.210985 | CHRNE |
| ILMN_2313889 | 0.00198 | 2.12E-05 | 0.240817 | -0.03254 | -0.55329 | 0.152394 | ZNF682 |
| ILMN_2345872 | 0.004131 | 1.67E-04 | 0.33225 | 0.054073 | -0.27544 | -0.03673 | SUMF2 |
| ILMN_1685371 | 0.005252 | 3.13E-04 | -0.11511 | -0.02877 | 0.267167 | -0.31137 | SUMF2 |
| ILMN_1757152 | 0.00914 | 8.97E-04 | 0.489275 | -0.0646 | -0.26188 | 0.191206 | MAGEC2 |
| ILMN_1723087 | 0.033303 | 0.00651 | 0.364792 | 0.090102 | -0.16066 | 0.067305 | MDK |
| ILMN_2203896 | 0.045605 | 0.010189 | 0.08141 | -0.10009 | 0.385902 | -0.51637 | SMAD7 |
| ILMN_3247506 | 0.005431 | 3.35E-04 | 0.330702 | 0.033938 | -0.30235 | 0.149297 | FCGR1C |
| ILMN_1710768 | 0.008587 | 7.98E-04 | 0.387633 | 0.070743 | -0.13884 | -0.0579 | ZNF673 |
| ILMN_3238950 | 0.028661 | 0.005252 | 0.473809 | -0.10871 | -0.28285 | 0.087656 | SNORD112 |
| ILMN_1803045 | 0.033772 | 0.006653 | 0.210103 | -0.38307 | 0.149016 | -0.92791 | TUBGCP5 |
| ILMN_1713918 | 0.025443 | 0.004394 | -0.4513 | -0.15556 | 0.17524 | -0.74024 | CYTH3 |
| ILMN_1702723 | 0.016887 | 0.002384 | 0.216401 | -0.11946 | -0.44959 | 0.319975 | STK24 |
| ILMN_1683231 | 0.006811 | 5.39E-04 | 0.407803 | 0.073473 | -0.24581 | -0.02702 | FAM83F |
| ILMN_1695311 | 0.038613 | 0.007986 | 0.101026 | 0.141198 | -0.30502 | -0.9325 | HLA-DMA |
| ILMN_1789781 | 0.030245 | 0.005677 | 0.157666 | 0.119805 | -0.26324 | 1.042327 | PIM3 |
| ILMN_2366212 | 0.00541 | 3.32E-04 | 0.231291 | 0.628407 | -0.3497 | -0.15106 | CD79B |
| ILMN_1788061 | 0.00499 | 2.67E-04 | 0.387971 | 0.090257 | -0.24116 | 0.189544 | Sep-12 |
| ILMN_1708064 | 0.005325 | 3.20E-04 | 0.251047 | 0.024621 | -0.27721 | -0.04763 | MAP4 |
| ILMN_2354140 | 0.006816 | 5.40E-04 | 0.156099 | -6.56E-04 | -0.2092 | 0.087556 | NAT5 |
| ILMN_1733407 | 0.01409 | 0.00181 | 0.274749 | 0.09622 | -0.19204 | 0.119768 | QTRTD1 |
| ILMN_2409451 | 0.021185 | 0.003338 | 0.253458 | -0.15644 | -0.17599 | 0.067655 | NCKAP1 |
| ILMN_3247088 | 0.004377 | 2.01E-04 | 0.710519 | 0.021245 | -0.1843 | 0.216094 | TMEM151B |
| ILMN_1672843 | 0.034153 | 0.006754 | 0.128426 | 0.017279 | -0.18355 | 0.001914 | FBXO8 |
| ILMN_1716377 | 0.013117 | 0.001616 | 0.197811 | 0.356635 | -0.30932 | -0.01753 | HMGCLL1 |
| ILMN_1698072 | 0.015493 | 0.002104 | 0.466552 | -0.38019 | -0.27823 | 0.018728 | PITRM1 |
| ILMN_1750641 | 0.016564 | 0.002318 | 0.395127 | 0.215259 | -0.30414 | 0.038552 | SRCAP |
| ILMN_1773696 | 0.008801 | 8.39E-04 | -0.88272 | -0.87192 | 0.013697 | 0.421088 | RGMB |
| ILMN_2142815 | 0.026385 | 0.00464 | 0.170656 | 0.207425 | -0.2619 | -0.31396 | RPL35 |
| ILMN_1684758 | 0.046483 | 0.010472 | 1.235068 | 0.418236 | 0.152862 | 0.124544 | ETFB |
| ILMN_1652719 | 0.003753 | 1.25E-04 | 0.230588 | 0.036318 | -0.29639 | 0.180919 | TMCO5A |
| ILMN_1792265 | 0.026588 | 0.004686 | 0.23356 | -0.11596 | -0.18128 | 0.105544 | TRIM4 |
| ILMN_1761486 | 0.004131 | 1.68E-04 | 0.252346 | 0.035816 | -0.29262 | 0.149813 | C13orf34 |
| ILMN_1658834 | 0.005029 | 2.74E-04 | 0.468776 | 0.158021 | -0.23076 | 0.305704 | ZC3H18 |
| ILMN_1809866 | 0.005587 | 3.54E-04 | 0.050278 | 0.006966 | -0.34898 | 0.222402 | WDR74 |
| ILMN_1792314 | 0.015265 | 0.002062 | 0.192712 | -0.00367 | -0.27753 | 0.04564 | ACTR1A |
| ILMN_3242830 | 0.00256 | 4.04E-05 | 0.428034 | 0.010247 | -0.28202 | 0.189085 | MSLNL |
| ILMN_1658207 | 0.001984 | 2.17E-05 | 0.246221 | 0.056543 | -0.275 | 0.268744 | MSLNL |
| ILMN_1750101 | 0.004528 | 2.14E-04 | 0.331625 | 0.050016 | -0.28999 | -0.05466 | S100A11 |
| ILMN_2382309 | 0.011611 | 0.001334 | 0.400245 | -0.06017 | -0.45567 | 0.306453 | TCL1B |
| ILMN_2147078 | 4.84E-04 | 1.40E-06 | 0.505082 | 0.16735 | -0.33443 | 0.180739 | PIWIL4 |
| ILMN_2259966 | 0.010614 | 0.001143 | 0.35834 | 0.098833 | -0.22239 | 0.085845 | TGM5 |
| ILMN_3297392 | 0.005404 | 3.31E-04 | 0.129786 | 0.027837 | -0.22736 | 0.103109 | UBE2K |
| ILMN_2377980 | 0.021804 | 0.003482 | 0.1045 | -0.47024 | 0.095995 | -0.10402 | PPP1CA |
| ILMN_1659852 | 0.003455 | 9.66E-05 | 0.664676 | -0.02695 | -0.26183 | 0.25995 | PWWP2B |
| ILMN_1724372 | 0.003028 | 6.26E-05 | 0.31491 | 0.056591 | -0.29356 | 0.559668 | SIRT3 |
| ILMN_1721034 | 0.005389 | 3.27E-04 | 0.322638 | -0.12653 | -0.16609 | -0.43379 | ZNF227 |
| ILMN_1712774 | 0.004068 | 1.62E-04 | 0.513978 | -0.04508 | -0.25367 | 0.170492 | IRS4 |
| ILMN_1685954 | 0.037822 | 0.007763 | 0.602925 | -0.11436 | -0.15269 | 0.316299 | HMBS |
| ILMN_2341487 | 0.013632 | 0.001711 | 0.702203 | -0.13302 | -0.24835 | 0.28491 | C11orf49 |
| ILMN_2302757 | 0.049335 | 0.011423 | 0.472257 | -0.04765 | -0.13102 | -0.07113 | FCGBP |
| ILMN_1739257 | 0.016725 | 0.002352 | 0.277733 | 0.038298 | -0.25745 | -0.01839 | EIF3E |
| ILMN_1806778 | 0.030819 | 0.005838 | 0.176358 | 0.069977 | -0.20561 | 0.029173 | UBE2E1 |
| ILMN_1689336 | 3.04E-05 | 2.58E-08 | -0.15116 | -0.06218 | -0.01834 | -0.96669 | HOXA10 |
| ILMN_2403911 | 0.003724 | 1.22E-04 | 0.240858 | -0.04939 | -0.21615 | 0.184264 | ARFIP1 |
| ILMN_1746704 | 0.004371 | 1.99E-04 | 0.304387 | -0.25168 | -0.20473 | 0.149957 | TRIM8 |
| ILMN_1718124 | 0.004963 | 2.62E-04 | 0.213886 | 0.214268 | -0.25118 | 0.356608 | OTOR |
| ILMN_1768454 | 0.025457 | 0.004398 | 0.322773 | 0.05018 | -0.1528 | 0.117669 | WDR21C |
| ILMN_1799100 | 0.002863 | 5.35E-05 | 0.355363 | 0.075278 | -0.27415 | 0.292042 | C16orf38 |
| ILMN_1741464 | 0.003673 | 1.19E-04 | -0.28099 | 0.031145 | 0.081835 | -0.31867 | HOOK3 |
| ILMN_2188204 | 0.034158 | 0.006759 | 0.170472 | 0.107603 | -0.19172 | 0.103708 | ATG12 |
| ILMN_1693598 | 6.06E-04 | 2.26E-06 | 0.647346 | 0.444258 | -0.47896 | 1.148393 | RTN4 |
| ILMN_1729495 | 0.043805 | 0.009622 | -0.16155 | 0.047998 | 0.007231 | -0.82059 | TRIM41 |
| ILMN_1747204 | 0.027282 | 0.00486 | 0.473766 | -0.69729 | -0.35252 | 0.209067 | HTRA2 |
| ILMN_1703803 | 0.009812 | 0.001001 | 0.453996 | 0.131228 | -0.10527 | 0.240796 | ADAMTS3 |
| ILMN_1754846 | 0.015264 | 0.002061 | 0.519849 | -0.00995 | -0.144 | 0.274048 | OR2L3 |
| ILMN_1748751 | 0.005728 | 3.73E-04 | 0.656506 | 0.088766 | -0.42969 | 0.883454 | NLF2 |
| ILMN_1727135 | 0.004929 | 2.57E-04 | 0.358174 | -0.02906 | -0.31449 | 0.134726 | FIBCD1 |
| ILMN_1741440 | 0.028923 | 0.005323 | -0.11451 | -0.19109 | 0.229111 | -0.36231 | SLC35A1 |
| ILMN_2112128 | 0.02926 | 0.005423 | -0.11729 | -0.50684 | 0.097361 | 0.531367 | MAPK4 |
| ILMN_1740146 | 0.038068 | 0.00783 | 0.159253 | 0.344105 | -0.29156 | -0.05445 | GPR83 |
| ILMN_3247223 | 0.003279 | 8.27E-05 | -0.35114 | 0.017391 | 0.35175 | -1.3135 | TPBG |
| ILMN_1799466 | 0.02209 | 0.00357 | 0.368149 | 0.020144 | -0.21447 | 0.065137 | MTX2 |
| ILMN_1736218 | 0.006752 | 5.30E-04 | 0.506492 | 0.191064 | -0.23528 | 0.247965 | CHRNB3 |
| ILMN_2411897 | 0.001255 | 7.78E-06 | 0.033507 | -0.28884 | -0.12413 | 1.446818 | KLF10 |
| ILMN_1659122 | 0.002465 | 3.61E-05 | 0.537724 | -0.0351 | -0.23385 | 1.366763 | KLF10 |
| ILMN_1801476 | 0.005629 | 3.60E-04 | 0.324451 | 0.069851 | -0.23412 | -0.13959 | CDS1 |
| ILMN_2284810 | 0.009655 | 9.73E-04 | 0.272343 | -0.02997 | -0.33884 | -0.00194 | CIDEA |
| ILMN_1692602 | 0.004074 | 1.62E-04 | 0.235816 | -0.03001 | -0.32367 | 0.08676 | ZAN |
| ILMN_1697735 | 0.011699 | 0.001352 | 0.10851 | -0.09489 | -0.29346 | 0.081687 | EWSR1 |
| ILMN_3241644 | 0.007034 | 5.68E-04 | 0.345954 | -0.07289 | -0.2958 | 0.008939 | LOC727797 |
| ILMN_1671209 | 0.009348 | 9.26E-04 | 0.337666 | -0.05553 | -0.27807 | 0.073184 | SYTL2 |
| ILMN_3246556 | 0.019653 | 0.002966 | 0.194584 | -0.09283 | -0.31863 | 0.221766 | LOC100133308 |
| ILMN_3242412 | 0.00822 | 7.42E-04 | 0.187725 | -0.00847 | -0.295 | 0.069712 | CALHM1 |
| ILMN_2376458 | 0.00943 | 9.41E-04 | 0.008536 | 0.437202 | -0.30272 | -0.9243 | CSF2RA |
| ILMN_1802105 | 0.03042 | 0.005734 | 0.337911 | -0.03025 | -0.14621 | 0.172672 | STYK1 |
| ILMN_1772787 | 0.003279 | 7.94E-05 | 0.363768 | -0.03852 | -0.27657 | 0.245802 | KIR2DS4 |
| ILMN_2279413 | 0.001466 | 1.17E-05 | 0.460864 | 0.013029 | -0.21343 | 0.097116 | SIAH1 |
| ILMN_2278112 | 0.024548 | 0.004147 | 0.070092 | 0.045806 | -0.21529 | 0.250404 | PRKACB |
| ILMN_1655348 | 0.003185 | 7.20E-05 | 0.412607 | 0.07661 | -0.25229 | 0.19599 | GPR1 |
| ILMN_1713550 | 0.001794 | 1.63E-05 | 0.4243 | 0.018653 | -0.28345 | -0.12546 | LGR6 |
| ILMN_1731353 | 0.012599 | 0.001523 | -0.14391 | -0.37967 | 0.103647 | -1.35013 | CHPF |
| ILMN_1691526 | 0.002863 | 5.43E-05 | 0.433719 | 2.18E-04 | -0.33295 | 0.106238 | MAPKAP1 |
| ILMN_1728517 | 0.004971 | 2.64E-04 | -0.01602 | -0.35956 | -0.04155 | -1.40177 | FNTB |
| ILMN_2148298 | 0.00674 | 5.29E-04 | 0.364943 | 0.135043 | -0.21776 | -0.09128 | ZNF749 |
| ILMN_1682323 | 0.009271 | 9.15E-04 | 0.361364 | 0.239972 | -0.39207 | -0.00914 | DDX51 |
| ILMN_1773117 | 0.006193 | 4.34E-04 | 0.117197 | 0.347492 | -0.21774 | 0.129308 | BCOR |
| ILMN_1745852 | 0.024892 | 0.004253 | -0.30847 | 0.027702 | 0.359781 | -0.30539 | WDR33 |
| ILMN_1756236 | 0.011623 | 0.001339 | 0.382069 | 0.068223 | -0.29087 | 0.362343 | C1QL2 |
| ILMN_1766245 | 0.018745 | 0.002776 | -0.8452 | 0.169419 | 0.194449 | -0.87877 | SUPT4H1 |
| ILMN_1682783 | 0.042576 | 0.009209 | 0.190105 | 0.023304 | -0.25602 | -0.13983 | TUG1 |
| ILMN_1724774 | 0.031916 | 0.006153 | 0.171773 | 0.115685 | -0.26187 | 0.283818 | SYT3 |
| ILMN_2401978 | 0.019001 | 0.002832 | 0.223273 | -0.03683 | -0.22184 | -0.06299 | STAT3 |
| ILMN_1762897 | 0.008971 | 8.70E-04 | 0.557743 | 0.327811 | -0.13602 | 0.295985 | GPR113 |
| ILMN_1775062 | 0.004068 | 1.61E-04 | 0.395859 | 0.030871 | -0.28633 | 0.141342 | LOC165186 |
| ILMN_3239298 | 0.004255 | 1.89E-04 | 0.284708 | -0.00306 | -0.37539 | 0.13328 | OTUD7B |
| ILMN_1786357 | 0.028517 | 0.005207 | 0.268997 | -0.06805 | -0.36087 | -0.12358 | CENTD2 |
| ILMN_1773310 | 0.043935 | 0.009665 | 0.355822 | 0.280439 | -0.19172 | 0.463736 | FUT5 |
| ILMN_1815890 | 0.003038 | 6.35E-05 | 0.458306 | 0.16677 | -0.27294 | 0.279352 | IL12RB1 |
| ILMN_1788802 | 0.002417 | 3.29E-05 | 0.179946 | -0.03589 | -0.33332 | 0.203885 | GCH1 |
| ILMN_1666222 | 0.040224 | 0.008484 | -0.5425 | 0.048651 | 0.457043 | 0.583739 | PHACTR3 |
| ILMN_2211032 | 0.041092 | 0.008769 | 0.418408 | 0.114097 | -0.20597 | 0.020813 | SPANXB1 |
| ILMN_1736796 | 0.026654 | 0.004706 | 0.14393 | -0.32375 | 0.549805 | -0.36054 | RB1CC1 |
| ILMN_1677859 | 0.006077 | 4.19E-04 | 0.262124 | -0.03948 | -0.2799 | 0.141585 | CCDC105 |
| ILMN_1728445 | 0.011109 | 0.001237 | 0.456672 | -0.02092 | -0.24808 | 0.143852 | IGFBP1 |
| ILMN_1658027 | 0.004473 | 2.08E-04 | 0.353848 | 0.002712 | -0.1479 | -0.23459 | RAD54L |
| ILMN_1654323 | 0.011539 | 0.001319 | 0.500751 | 0.129535 | -0.25615 | 0.120741 | UBE3A |
| ILMN_2121068 | 0.003387 | 8.89E-05 | 0.184996 | 0.141421 | -0.29983 | 0.161069 | ADAM17 |
| ILMN_1798588 | 0.011306 | 0.001272 | -0.42964 | -0.11702 | 0.185654 | -0.08478 | HLTF |
| ILMN_1696488 | 0.018369 | 0.002705 | 0.573766 | 0.113448 | -0.2751 | 0.156329 | FGF23 |
| ILMN_1659541 | 0.028004 | 0.005058 | 0.597053 | 0.139217 | -0.21141 | 0.181273 | CRELD1 |
| ILMN_1789094 | 0.019102 | 0.002852 | -0.23191 | 0.027129 | 0.235877 | -0.69483 | NINL |
| ILMN_2096747 | 0.003451 | 9.60E-05 | 0.302294 | 0.089708 | -0.31917 | 0.238777 | SNORA33 |
| ILMN_1755405 | 0.028397 | 0.005174 | 0.150149 | 0.108257 | -0.08662 | -0.47987 | FRAG1 |
| ILMN_1681724 | 0.022306 | 0.00363 | 0.459789 | 0.133277 | -0.52594 | 0.217768 | FPGS |
| ILMN_1713948 | 0.021845 | 0.003495 | 0.63783 | 0.029465 | -0.18694 | 0.097153 | FPGS |
| ILMN_1688625 | 0.044235 | 0.009764 | 0.049863 | 0.032431 | -0.35616 | 0.222759 | AIM1 |
| ILMN_1798712 | 0.006231 | 4.40E-04 | 0.198061 | -0.08892 | -0.18589 | 0.118831 | USP4 |
| ILMN_1726459 | 0.017873 | 0.002593 | 1.23117 | 0.039028 | 0.13098 | 1.309143 | NPAS4 |
| ILMN_1652602 | 0.011125 | 0.00124 | 0.285306 | -0.30081 | 0.583674 | -0.61033 | C11orf35 |
| ILMN_1794266 | 0.003455 | 9.65E-05 | 0.497609 | 0.034563 | -0.22813 | 0.186124 | SPINLW1 |
| ILMN_1662747 | 0.038832 | 0.008045 | 0.44994 | 0.467827 | 0.402104 | -1.09959 | EVI2A |
| ILMN_1798886 | 0.006731 | 5.27E-04 | -0.25063 | -0.51136 | 0.307468 | -0.41017 | NUDT21 |
| ILMN_2059780 | 0.046148 | 0.010374 | 0.254492 | -0.43362 | 0.353173 | 1.345955 | CYP4A22 |
| ILMN_1759219 | 0.011494 | 0.001307 | -0.84769 | -0.39516 | 0.316022 | -0.11894 | ZMAT5 |
| ILMN_2248112 | 0.006731 | 5.27E-04 | 0.317248 | -0.00783 | -0.24552 | 0.528414 | TBX1 |
| ILMN_1778360 | 0.029544 | 0.005498 | 0.153309 | -0.43225 | 0.078151 | -0.27405 | PYGB |
| ILMN_1661137 | 0.027701 | 0.004981 | 0.089041 | 9.82E-04 | -0.19464 | -0.84914 | SH3RF2 |
| ILMN_1735453 | 0.045531 | 0.010164 | 0.15076 | 0.103248 | -0.17975 | -0.05463 | FAM98A |
| ILMN_1664691 | 0.005554 | 3.50E-04 | 0.359904 | 0.201491 | -0.32087 | 0.119296 | DAPP1 |
| ILMN_1812031 | 0.013536 | 0.001692 | 0.238638 | 6.53E-04 | -0.33068 | -0.01124 | PALM |
| ILMN_1731644 | 0.003963 | 1.44E-04 | 0.216422 | 0.020604 | -0.25975 | -0.20335 | SETDB2 |
| ILMN_1720724 | 0.036405 | 0.007385 | 0.223161 | 0.265734 | -0.30012 | -0.1256 | IFT122 |
| ILMN_1667594 | 0.001337 | 9.00E-06 | 0.451582 | 0.140619 | -0.32112 | 1.185369 | KLF10 |
| ILMN_1770676 | 0.003989 | 1.46E-04 | 0.373233 | -0.01529 | -0.27808 | 0.077621 | CAPRIN1 |
| ILMN_1797813 | 0.027132 | 0.004814 | 0.218744 | 0.129581 | -0.17925 | 0.030311 | SUZ12 |
| ILMN_1794063 | 0.031883 | 0.006138 | 0.132633 | 0.012288 | -0.23633 | 0.123508 | ANKRD27 |
| ILMN_1738099 | 0.013217 | 0.001639 | -0.32777 | 0.273813 | -0.03799 | 0.597806 | C2orf34 |
| ILMN_1782441 | 0.029119 | 0.005381 | 0.918281 | -0.26997 | -0.10406 | -0.24059 | CDKL2 |
| ILMN_1718248 | 0.007725 | 6.67E-04 | 0.422347 | 0.031823 | -0.2066 | 0.255683 | CFHR5 |
| ILMN_3310491 | 0.042688 | 0.009239 | 0.212884 | -0.03308 | -0.28426 | -0.01027 | MIR1978 |
| ILMN_1698766 | 0.030872 | 0.00586 | 0.118732 | -0.06674 | -0.29762 | 0.155407 | PYCARD |
| ILMN_1715332 | 0.020624 | 0.003221 | 0.360806 | -0.97773 | -0.17842 | 0.681115 | TTC21A |
| ILMN_1759012 | 0.001283 | 8.18E-06 | 0.702401 | 0.061612 | -0.13831 | 0.147903 | PPP1R1B |
| ILMN_1679731 | 0.004255 | 1.89E-04 | 0.313859 | -0.08094 | 0.431274 | -0.69308 | SLC15A4 |
| ILMN_1679277 | 0.002067 | 2.41E-05 | 0.412342 | 0.168687 | -0.3537 | 0.119145 | CMTM3 |
| ILMN_1662091 | 0.004999 | 2.68E-04 | 0.361888 | -0.02287 | -0.27587 | 0.130807 | FLJ43860 |
| ILMN_1787689 | 0.014613 | 0.001914 | 0.353721 | 0.015104 | -0.22969 | 0.17207 | A2BP1 |
| ILMN_1670832 | 0.005413 | 3.33E-04 | 0.465893 | 0.031312 | -0.28191 | 0.10098 | LOC441070 |
| ILMN_1659495 | 0.020015 | 0.003063 | 0.336195 | 0.072306 | -0.18112 | 0.01647 | HBS1L |
| ILMN_1718013 | 0.017419 | 0.002497 | -0.48159 | -0.19139 | 0.17686 | -0.25601 | CPSF3L |
| ILMN_2284706 | 0.031637 | 0.006068 | 0.024947 | -1.19486 | 0.081673 | -0.38323 | PHF11 |
| ILMN_1782993 | 0.004255 | 1.89E-04 | 0.350371 | 0.066309 | -0.17645 | 0.724105 | DHODH |
| ILMN_1756920 | 0.002996 | 5.96E-05 | 0.294691 | 0.070467 | -0.30473 | -0.13467 | ADAM15 |
| ILMN_1763264 | 0.031142 | 0.005926 | 0.029045 | -0.39114 | 0.26214 | -0.7599 | MRPL2 |
| ILMN_1787813 | 0.003279 | 8.31E-05 | 0.475359 | -0.08298 | -0.16542 | -0.4142 | SLC5A3 |
| ILMN_1656316 | 0.010323 | 0.001091 | 0.410883 | 0.074515 | -0.25275 | 0.109429 | ZMYM3 |
| ILMN_1753903 | 0.004464 | 2.07E-04 | 0.271729 | 0.014591 | -0.27348 | 0.180716 | C1GALT1C1 |
| ILMN_3249620 | 0.031369 | 0.005994 | 0.207611 | 0.089983 | -0.27663 | -0.3072 | LOC220930 |
| ILMN_3251506 | 0.005956 | 4.02E-04 | 0.155811 | -0.12794 | 0.088484 | -0.50632 | ZNF69 |
| ILMN_1671215 | 0.038285 | 0.007892 | 0.248624 | -0.05752 | -0.23037 | -0.0359 | FBXL19 |
| ILMN_2070300 | 0.048953 | 0.011303 | 0.26591 | 0.178125 | -0.17072 | -0.1935 | LSM2 |
| ILMN_1777845 | 0.020086 | 0.003085 | 0.637277 | 0.128738 | -0.14669 | -0.10687 | NOTCH2 |
| ILMN_1795507 | 0.006332 | 4.55E-04 | -0.29913 | 0.051426 | 0.097826 | -1.53478 | ABCA6 |
| ILMN_1761031 | 0.005237 | 3.08E-04 | 0.079716 | 0.556538 | 0.198621 | -0.42211 | PTPDC1 |
| ILMN_2096116 | 0.025468 | 0.004404 | -0.21236 | 0.033654 | 0.194504 | -0.46098 | HSP90B1 |
| ILMN_1661138 | 0.037214 | 0.00759 | -0.06444 | 0.273624 | 0.05338 | -0.34348 | GON4L |
| ILMN_2333319 | 0.002998 | 6.04E-05 | 0.243376 | 0.045764 | -0.2395 | 0.036529 | PTBP1 |
| ILMN_2070583 | 0.004191 | 1.73E-04 | 0.444709 | -0.0108 | -0.23637 | 0.269942 | DEFB128 |
| ILMN_1782953 | 0.004191 | 1.78E-04 | 0.860556 | -0.31017 | 0.353617 | 2.215211 | MMP19 |
| ILMN_3308698 | 0.016726 | 0.002353 | 0.326746 | -0.06475 | -0.10775 | 0.118734 | MIR1276 |
| ILMN_1800517 | 0.01649 | 0.002294 | -0.18555 | 0.158335 | 0.214075 | -0.14649 | ODF2L |
| ILMN_1680618 | 4.48E-05 | 4.65E-08 | 0.048565 | 0.135959 | -0.40483 | 1.449829 | MYC |
| ILMN_1796475 | 0.003571 | 1.06E-04 | 0.516819 | -0.02545 | -0.21798 | 0.219573 | GPR6 |
| ILMN_1751016 | 0.020241 | 0.003124 | -1.00675 | -0.28727 | 0.094429 | -0.01239 | LONRF2 |
| ILMN_3233229 | 0.01877 | 0.00278 | -0.3351 | -0.95169 | 0.33524 | -0.35679 | SNHG7 |
| ILMN_1794364 | 0.001698 | 1.48E-05 | -0.08522 | 0.624959 | -0.72531 | -0.08187 | CTSW |
| ILMN_3237067 | 0.044707 | 0.0099 | 0.383808 | 0.011222 | -0.18507 | 0.084906 | FAM75B |
| ILMN_1814789 | 0.004192 | 1.80E-04 | -0.18145 | -0.04428 | 0.188761 | -0.69911 | UBAP2L |
| ILMN_3230215 | 0.003109 | 6.75E-05 | 0.330113 | -0.0865 | -0.38666 | 0.180286 | TOX2 |
| ILMN_1798657 | 0.021201 | 0.003342 | 0.058398 | -0.17796 | 0.00876 | -0.46011 | TBL1XR1 |
| ILMN_2163538 | 0.013655 | 0.001717 | -0.01048 | 0.100321 | -0.27469 | 0.186349 | TTTY11 |
| ILMN_1745409 | 0.027475 | 0.004915 | 0.189551 | 0.017135 | -0.31378 | 0.084251 | LOC340529 |
| ILMN_1706304 | 0.020042 | 0.003075 | 0.305358 | 0.42154 | -0.359 | -0.11318 | EIF2C4 |
| ILMN_1779706 | 0.041413 | 0.008873 | 0.408776 | 0.144384 | -0.18364 | 0.44323 | TP53BP2 |
| ILMN_2192316 | 0.008458 | 7.79E-04 | 0.365827 | -0.00407 | -0.16212 | 0.01836 | TOP1 |
| ILMN_1707748 | 0.004191 | 1.78E-04 | 0.548276 | 0.157076 | -0.49847 | 0.986026 | PIM3 |
| ILMN_1723123 | 0.010497 | 0.001121 | 0.386231 | 0.046311 | -0.28352 | -0.10338 | FGFR3 |
| ILMN_2151817 | 0.01823 | 0.00267 | 0.373711 | 0.044915 | -0.16961 | -0.00353 | PFN1 |
| ILMN_1655906 | 0.039355 | 0.008225 | 0.309067 | 0.108873 | -0.25152 | -0.13467 | FBXW7 |
| ILMN_1680777 | 0.031916 | 0.006151 | 0.054441 | 0.01409 | -0.24775 | 0.506858 | MAP2K3 |
| ILMN_2382679 | 0.01078 | 0.001172 | 0.338695 | 0.004621 | -0.33224 | 0.013991 | REG3A |
| ILMN_1804886 | 0.008098 | 7.22E-04 | 0.323134 | 0.033553 | -0.19875 | 0.118564 | HTR1D |
| ILMN_1751589 | 0.005256 | 3.13E-04 | 0.168003 | -0.06963 | -0.23518 | 0.253722 | NUDCD2 |
| ILMN_2201668 | 0.006883 | 5.48E-04 | 0.227162 | 0.084772 | -0.27912 | 0.440014 | SLC19A2 |
| ILMN_1815949 | 0.004191 | 1.78E-04 | 0.18049 | 0.080196 | -0.27575 | 0.181024 | SLC24A4 |
| ILMN_1723822 | 0.023886 | 0.004004 | 0.059257 | -0.12962 | -0.27381 | 0.044255 | PBRM1 |
| ILMN_1686555 | 0.016318 | 0.002264 | -0.12169 | 0.104327 | -0.26836 | 0.2993 | FYN |
| ILMN_1771179 | 0.011623 | 0.00134 | 0.371694 | -0.0249 | -0.19478 | 0.139857 | CYB561 |
| ILMN_3307841 | 0.005866 | 3.91E-04 | -0.50004 | 0.395878 | 0.384643 | -1.46649 | AGR2 |
| ILMN_2398664 | 0.027238 | 0.004847 | 0.210532 | -0.04658 | -0.30953 | 0.128149 | RNF34 |
| ILMN_1686750 | 0.009163 | 9.00E-04 | 0.453556 | 0.032325 | -0.22942 | -0.16753 | MGEA5 |
| ILMN_1684205 | 0.008407 | 7.69E-04 | 0.363092 | 0.064152 | -0.26002 | 0.108421 | CIB1 |
| ILMN_3310745 | 0.022364 | 0.003646 | 0.650439 | 0.010189 | -0.30846 | 0.07316 | SNORD116-27 |
| ILMN_1775753 | 0.027172 | 0.004829 | 0.090397 | -0.20445 | -0.18791 | 0.490681 | FBXW2 |
| ILMN_1702965 | 0.008993 | 8.76E-04 | 0.272577 | 0.172893 | -0.2918 | 0.134693 | PDE6H |
| ILMN_2227968 | 0.029013 | 0.005348 | -0.11127 | -0.95893 | 0.261315 | -1.01252 | NTHL1 |
| ILMN_1685663 | 0.030256 | 0.00568 | -0.54105 | -0.11553 | 0.345054 | -0.9569 | CYP24A1 |
| ILMN_3248941 | 0.002852 | 5.24E-05 | 0.189263 | -0.06556 | 0.23084 | -0.40331 | C6orf225 |
| ILMN_1686889 | 0.005476 | 3.43E-04 | 0.156763 | 0.011783 | 0.13303 | -0.81861 | C6orf225 |
| ILMN_1749838 | 0.024086 | 0.004046 | 0.101426 | 0.212243 | -0.22248 | -0.03458 | MZF1 |
| ILMN_1670816 | 0.027168 | 0.004826 | 0.048806 | 0.139071 | -0.12862 | -0.40341 | MZF1 |
| ILMN_1718477 | 0.010355 | 0.001097 | 0.19065 | 0.034366 | -0.26308 | 0.205536 | UPK3A |
| ILMN_1662155 | 0.004065 | 1.59E-04 | 0.258394 | 0.323453 | -0.45095 | 0.234494 | PRKCZ |
| ILMN_1700382 | 0.007647 | 6.56E-04 | 0.44906 | 0.128693 | -0.25764 | 0.151804 | HOXA1 |
| ILMN_1813028 | 0.009372 | 9.31E-04 | -0.20442 | 0.384037 | 0.022957 | -0.06848 | CBX5 |
| ILMN_2396691 | 0.012749 | 0.001551 | -0.31569 | 0.185013 | 0.085168 | -0.16149 | AKAP9 |
| ILMN_1745817 | 0.007395 | 6.18E-04 | -0.29102 | -0.47858 | 0.343268 | -0.94609 | NELL1 |
| ILMN_1767662 | 0.033347 | 0.006522 | 0.077165 | -0.30548 | 0.414558 | -0.5753 | LASS6 |
| ILMN_1760493 | 0.009786 | 9.96E-04 | 0.004415 | -0.11643 | 0.146398 | -0.95837 | LIMS2 |
| ILMN_1737211 | 0.013523 | 0.00169 | 0.180353 | 0.215161 | 0.102874 | -0.40407 | ZNF585A |
| ILMN_1692080 | 0.003523 | 1.01E-04 | 0.288195 | 0.334066 | -0.34141 | 0.085187 | ANKH |
| ILMN_1797604 | 0.024704 | 0.004202 | 0.100244 | -0.4839 | 0.068455 | 0.065996 | CAP1 |
| ILMN_2106265 | 0.01263 | 0.00153 | -0.97828 | -0.61602 | 0.13599 | -0.92641 | GDPD1 |
| ILMN_3241661 | 0.004627 | 2.25E-04 | 0.109121 | 0.057749 | -0.26164 | 0.07777 | LRRIQ3 |
| ILMN_1674985 | 0.048016 | 0.011003 | 0.205381 | -0.2218 | -0.06815 | -0.21602 | TMEM51 |
| ILMN_2350301 | 0.015838 | 0.00217 | 0.374551 | 0.083179 | -0.16547 | 0.142701 | PRB1 |
| ILMN_2097421 | 0.003631 | 1.14E-04 | 0.362465 | 0.211084 | -0.12744 | -0.13146 | MRPL51 |
| ILMN_1723874 | 0.016893 | 0.002386 | 0.203335 | -0.07006 | -0.24501 | -0.09417 | MRPS6 |
| ILMN_1808457 | 0.007643 | 6.53E-04 | 0.335748 | 0.088865 | -0.30915 | 0.117228 | TBX18 |
| ILMN_1687023 | 0.007468 | 6.29E-04 | 0.156794 | 0.102974 | -0.37342 | 0.119216 | GJC1 |
| ILMN_1700766 | 0.011023 | 0.001224 | 0.349893 | -0.10774 | -0.30493 | -0.13715 | ZNF324B |
| ILMN_2325056 | 0.042758 | 0.009261 | 0.372168 | -0.33305 | -0.2694 | 0.720205 | SLC29A4 |
| ILMN_1725726 | 0.017422 | 0.002498 | 0.114954 | -0.06071 | -0.30496 | 0.125348 | DHRS2 |
| ILMN_1698019 | 0.01551 | 0.002108 | 0.118374 | -0.1641 | -0.2191 | 0.439423 | LGMN |
| ILMN_1697220 | 0.04782 | 0.010934 | 0.482674 | -0.37905 | 0.157071 | 0.152426 | NT5E |
| ILMN_1689852 | 0.004063 | 1.58E-04 | 0.413673 | 0.100406 | -0.34741 | 0.131988 | PAQR6 |
| ILMN_2407464 | 0.040086 | 0.008436 | 0.093628 | 0.241821 | -0.1057 | -0.52258 | FASTK |
| ILMN_1796497 | 0.024704 | 0.004203 | 0.169399 | 0.116154 | -0.19558 | -0.0794 | PIP3-E |
| ILMN_1737640 | 0.00789 | 6.91E-04 | 0.693901 | -0.06668 | -0.24922 | 0.168408 | CSAG1 |
| ILMN_1794085 | 0.006371 | 4.62E-04 | -1.44955 | -0.34769 | 0.22932 | -0.11816 | SAPS1 |
| ILMN_1732053 | 0.007543 | 6.41E-04 | 0.322914 | 0.101532 | -0.31575 | -0.05582 | SNRNP70 |
| ILMN_1713285 | 0.017042 | 0.002413 | 0.344076 | -0.10669 | -0.09263 | -0.14785 | NAPA |
| ILMN_3248270 | 0.024892 | 0.004249 | 0.326795 | -0.07127 | -0.40902 | 0.046756 | SNORD62B |
| ILMN_2383300 | 0.004063 | 1.58E-04 | 0.210118 | 0.103897 | -0.21997 | -0.00445 | PTPRU |
| ILMN_1656992 | 0.003058 | 6.41E-05 | 0.364658 | -0.00279 | -0.26291 | 0.176873 | NLRP10 |
| ILMN_1804663 | 0.003834 | 1.31E-04 | 0.257168 | 0.080309 | -0.34338 | -0.20557 | THBS3 |
| ILMN_2347805 | 0.037434 | 0.007659 | 0.288346 | -0.16074 | 0.015888 | -0.37698 | EXOC1 |
| ILMN_2222443 | 0.00499 | 2.67E-04 | 0.394839 | 0.097632 | -0.2474 | 0.126127 | KLRK1 |
| ILMN_1716384 | 0.03734 | 0.007627 | 0.091018 | -0.34564 | 0.063087 | 0.08101 | ATL2 |
| ILMN_3248223 | 2.60E-04 | 5.53E-07 | 0.466743 | 0.014235 | -0.2218 | 0.261429 | SCARNA12 |
| ILMN_1653821 | 0.046483 | 0.010473 | 1.065783 | 0.323816 | -0.10195 | 0.179006 | ADRA1A |
| ILMN_1697301 | 0.011494 | 0.001309 | 0.218528 | 0.058488 | -0.27388 | 0.101498 | ST8SIA3 |
| ILMN_1703314 | 0.001853 | 1.85E-05 | 0.049354 | -0.02712 | -0.33381 | 0.259683 | KLHL36 |
| ILMN_1765833 | 0.029114 | 0.005378 | 0.401189 | -0.00996 | -0.21827 | 0.012004 | SLC9A3R2 |
| ILMN_3246304 | 0.003185 | 7.20E-05 | 0.46913 | 0.056128 | -0.23138 | 0.137422 | CPSF4L |
| ILMN_1732296 | 0.011385 | 0.001284 | 0.506113 | -0.16152 | -0.25791 | 0.341608 | ID3 |
| ILMN_1730984 | 0.003087 | 6.66E-05 | 0.600264 | -0.0741 | -0.35426 | 0.005836 | OR4C13 |
| ILMN_1697200 | 0.04697 | 0.010645 | -0.08906 | 0.111043 | 0.189254 | -0.54441 | MON2 |
| ILMN_2081682 | 0.014292 | 0.001851 | 0.030796 | 0.112089 | -0.29641 | 0.154055 | SMAP2 |
| ILMN_1782851 | 0.045284 | 0.010092 | 0.224361 | -0.2497 | -0.05105 | 0.552477 | TAPBP |
| ILMN_1811648 | 0.048768 | 0.011244 | 0.036918 | 0.101075 | -0.17506 | 0.03361 | DCAKD |
| ILMN_3240838 | 0.021584 | 0.00343 | 0.155731 | 0.098659 | -0.2991 | -0.07411 | SLC25A6 |
| ILMN_2347145 | 0.013925 | 0.001775 | 0.143649 | -0.00783 | -0.24658 | 0.007707 | DCN |
| ILMN_1675523 | 0.009895 | 0.001014 | 0.317947 | -7.03E-04 | -0.2772 | 0.061382 | PPAP2C |
| ILMN_1687863 | 0.005075 | 2.79E-04 | 0.710474 | 0.08497 | -0.14827 | 0.229862 | LSS |
| ILMN_2102752 | 0.00885 | 8.47E-04 | 0.202442 | 0.056303 | -0.30951 | 0.177986 | LOC285453 |
| ILMN_1715972 | 0.013978 | 0.001786 | 0.59568 | 0.094584 | -0.2726 | 0.194975 | SLC22A2 |
| ILMN_1718990 | 0.013023 | 0.001603 | 0.858001 | -0.60776 | -0.30028 | 1.113538 | NFRKB |
| ILMN_1769092 | 0.046137 | 0.010367 | -0.10067 | -1.08301 | 0.096106 | -0.62473 | FAM176B |
| ILMN_2363843 | 0.005197 | 3.03E-04 | 0.470113 | 0.081045 | -0.24167 | 0.227092 | RFX5 |
| ILMN_3238264 | 0.012108 | 0.001421 | 0.451483 | 0.053984 | -0.29738 | 0.223854 | PSAPL1 |
| ILMN_1789492 | 0.015541 | 0.002115 | 0.270582 | 0.046582 | -0.24377 | -0.01908 | ZDHHC8 |
| ILMN_1661554 | 0.004344 | 1.96E-04 | 0.341793 | 0.018852 | -0.18187 | -0.02798 | DIAPH1 |
| ILMN_3242818 | 0.022775 | 0.003741 | 0.175508 | 0.012679 | -0.22042 | 0.298286 | RNU105C |
| ILMN_2318430 | 0.003346 | 8.69E-05 | 0.07771 | -0.02743 | -0.3418 | 0.366011 | EIF5 |
| ILMN_3307752 | 0.006207 | 4.35E-04 | 0.350186 | 0.106323 | -0.28011 | 0.168787 | DLEU7 |
| ILMN_1736340 | 0.034407 | 0.006831 | 0.11955 | 0.135924 | -0.13891 | 0.101627 | ANGEL2 |
| ILMN_1724280 | 1.28E-04 | 1.65E-07 | -0.01536 | 0.260553 | -0.12075 | 2.201164 | EGR3 |
| ILMN_2397294 | 0.001297 | 8.39E-06 | 0.472342 | 0.136208 | -0.31664 | 0.072675 | PLCB4 |
| ILMN_1723116 | 8.86E-04 | 4.18E-06 | -0.01758 | -0.23769 | 0.63454 | -0.21586 | AMFR |
| ILMN_1767342 | 0.006616 | 5.03E-04 | 0.641066 | -0.06446 | -0.29395 | 0.114516 | EPHA5 |
| ILMN_1746243 | 0.003411 | 9.40E-05 | 0.23662 | -0.04637 | -0.34073 | -0.24877 | TES |
| ILMN_1795836 | 0.043333 | 0.009449 | 0.106709 | -0.01069 | -0.23081 | 0.042979 | C21orf57 |
| ILMN_2411127 | 0.035216 | 0.00705 | -0.17241 | -0.26629 | 0.19484 | -0.51018 | C21orf57 |
| ILMN_1666624 | 0.004255 | 1.89E-04 | 0.334676 | -0.0255 | -0.30559 | 0.165543 | RABL2A |
| ILMN_3245006 | 0.048324 | 0.011105 | 0.14778 | -0.05679 | -0.32028 | 0.177609 | C12orf68 |
| ILMN_3237632 | 0.046345 | 0.010427 | 0.053046 | 0.248072 | -0.17882 | 0.111971 | NCLN |
| ILMN_1728353 | 0.005042 | 2.75E-04 | -0.26046 | 0.0059 | 0.419031 | -0.24793 | EYA3 |
| ILMN_2360028 | 0.007668 | 6.61E-04 | 0.324116 | 0.043107 | -0.20297 | 0.012714 | NFATC3 |
| ILMN_2110829 | 9.19E-06 | 6.51E-09 | -0.15514 | -0.12943 | 0.346261 | -0.72244 | LOC441743 |
| ILMN_1713875 | 0.002167 | 2.76E-05 | 0.484477 | -0.02726 | -0.37531 | 3.15E-04 | NME1 |
| ILMN_1668236 | 0.008174 | 7.32E-04 | 0.732421 | -0.08823 | -0.20893 | 0.23883 | KCNB2 |
| ILMN_1757467 | 0.006786 | 5.35E-04 | 0.365574 | -0.38756 | -0.11366 | 0.008818 | H1F0 |
| ILMN_1771841 | 5.01E-04 | 1.51E-06 | 0.613539 | -0.39295 | -0.49944 | 1.779359 | FOSL1 |
| ILMN_1772686 | 0.021466 | 0.003404 | 0.258511 | 0.21506 | -0.23915 | -0.07498 | FGD3 |
| ILMN_1796235 | 0.031826 | 0.006124 | 0.166878 | 0.025093 | -0.30631 | -0.01486 | CIRH1A |
| ILMN_3188110 | 0.004034 | 1.51E-04 | -0.63224 | -0.03876 | 0.148674 | -0.85885 | C19orf60 |
| ILMN_1792922 | 0.047218 | 0.010741 | 0.198037 | -0.01237 | -0.21729 | 0.067793 | SOD2 |
| ILMN_1800958 | 0.041578 | 0.008921 | -0.13509 | -0.29885 | 0.387506 | -0.72006 | ALS2CR4 |
| ILMN_1748027 | 0.005627 | 3.59E-04 | 0.749666 | 0.59918 | -0.26528 | 1.569278 | PGAM5 |
| ILMN_1670149 | 0.005879 | 3.93E-04 | 0.299968 | 0.003651 | -0.26797 | 0.126887 | OR4S1 |
| ILMN_1682232 | 0.001466 | 1.17E-05 | -0.96856 | 0.195929 | 0.107808 | -1.25003 | MIER1 |
| ILMN_2366587 | 0.008458 | 7.78E-04 | 0.348504 | -0.08602 | -0.27038 | 0.212682 | SPDYA |
| ILMN_1698233 | 0.043995 | 0.009684 | 0.309268 | -0.11699 | -0.21353 | 0.113434 | RWDD2B |
| ILMN_1768795 | 0.012409 | 0.00148 | -0.32045 | -0.46092 | 0.168101 | -0.8 | RNF113B |
| ILMN_1789830 | 0.018552 | 0.002738 | 0.22947 | 0.102117 | -0.26668 | -0.29929 | CFLAR |
| ILMN_1756443 | 0.003917 | 1.40E-04 | 0.511703 | 0.166441 | -0.31444 | 0.023083 | INHA |
| ILMN_1803590 | 0.001984 | 2.17E-05 | -0.27955 | 0.093007 | 0.409295 | -0.85311 | ZFC3H1 |
| ILMN_2308582 | 0.018199 | 0.002663 | 0.361505 | 0.016363 | -0.15371 | 0.060615 | CYB5R3 |
| ILMN_1696189 | 0.005057 | 2.77E-04 | 0.491588 | 0.068749 | -0.27396 | 0.155079 | KCNAB1 |
| ILMN_3247945 | 0.012752 | 0.001552 | 0.203834 | 0.08959 | -0.20249 | 0.269233 | GOLGA7B |
| ILMN_3235143 | 0.017135 | 0.002437 | 0.49393 | -0.11418 | -0.18991 | 0.136991 | LOC731779 |
| ILMN_3248703 | 0.040154 | 0.008458 | 0.299892 | 0.517465 | -0.17375 | -0.05079 | SNORD99 |
| ILMN_1659099 | 0.040127 | 0.00845 | 0.047972 | -0.32326 | 0.303473 | -0.13388 | ROCK2 |
| ILMN_2058337 | 0.021923 | 0.003521 | 0.231037 | 0.039343 | -0.30577 | 0.082151 | ROCK2 |
| ILMN_2371911 | 0.030819 | 0.005841 | 0.040864 | -0.26764 | -0.09304 | -1.04561 | MUC1 |
| ILMN_1749372 | 0.028652 | 0.005245 | -0.4008 | -0.1807 | 0.032539 | -1.18411 | GGT5 |
| ILMN_1778897 | 0.009015 | 8.78E-04 | 0.477306 | 0.023383 | -0.25337 | 0.16057 | TBC1D21 |
| ILMN_1726805 | 0.005029 | 2.74E-04 | 0.332645 | 0.165193 | -0.2161 | 0.15352 | STX2 |
| ILMN_1756102 | 0.049954 | 0.0116 | 0.254325 | -0.30986 | -0.10093 | -0.66506 | RFX3 |
| ILMN_3248263 | 0.032566 | 0.006314 | 0.186959 | -0.01595 | -0.07396 | -0.57537 | CCDC93 |
| ILMN_1777725 | 0.019161 | 0.002867 | 0.228958 | 0.045786 | -0.20347 | 0.138792 | LSM14B |
| ILMN_2076658 | 0.040796 | 0.008669 | -0.40031 | -0.18229 | 0.204459 | -0.53391 | MRPL1 |
| ILMN_1755727 | 0.038996 | 0.008116 | -0.01126 | -0.19562 | 0.244297 | -0.66868 | KDM5B |
| ILMN_1812249 | 0.024892 | 0.004249 | 0.372537 | 0.096045 | -0.12487 | 0.151488 | C15orf54 |
| ILMN_2336393 | 0.021575 | 0.003428 | 0.042016 | 0.169144 | -0.27729 | -0.06568 | SMN2 |
| ILMN_1664511 | 0.006011 | 4.10E-04 | 0.421673 | -0.03343 | -0.31777 | -0.14009 | NDC80 |
| ILMN_2206592 | 0.005129 | 2.91E-04 | 0.197175 | -0.02288 | -0.29423 | 0.166635 | PRAMEF9 |
| ILMN_2317751 | 0.041594 | 0.008937 | -0.55463 | -0.72615 | 0.035929 | 0.452474 | REC8 |
| ILMN_2206041 | 0.025859 | 0.004518 | 0.081113 | 0.142662 | 0.002497 | 0.572768 | OR8U1 |
| ILMN_2400926 | 0.004191 | 1.75E-04 | 0.254421 | 0.287294 | -0.35149 | 0.39197 | OPRL1 |
| ILMN_2400922 | 5.81E-04 | 2.11E-06 | 0.434881 | 0.210761 | -0.46029 | 0.141875 | OPRL1 |
| ILMN_1662950 | 0.00898 | 8.73E-04 | 0.887838 | 0.350528 | -0.35222 | 0.727867 | EPS8L1 |
| ILMN_1700268 | 0.0491 | 0.011356 | 0.286795 | -0.01478 | -0.19791 | 0.169706 | QPRT |
| ILMN_2193175 | 0.035272 | 0.007073 | 0.074729 | 0.251419 | 0.132418 | -0.80439 | C3orf1 |
| ILMN_1769720 | 0.028843 | 0.005303 | 0.148646 | -0.59213 | -0.14057 | 0.479405 | STAU2 |
| ILMN_2318459 | 0.019169 | 0.002869 | 0.202321 | -0.10333 | -0.29043 | 0.1945 | TMEM8B |
| ILMN_1775520 | 0.008651 | 8.06E-04 | 0.204829 | 0.111793 | -0.27175 | 0.222489 | CCDC113 |
| ILMN_3310910 | 0.005228 | 3.05E-04 | 0.373629 | -0.0806 | -0.29836 | 0.083767 | MIR29A |
| ILMN_1795063 | 0.007901 | 6.94E-04 | -0.67761 | 0.122761 | 0.3643 | -1.61125 | ZADH2 |
| ILMN_2407605 | 0.027914 | 0.005032 | 0.07071 | 0.163303 | -0.38937 | 0.194005 | GIYD2 |
| ILMN_1668408 | 0.046137 | 0.010366 | 0.24461 | -0.15101 | -0.21415 | 0.100529 | AIFM1 |
| ILMN_2400546 | 0.012268 | 0.00145 | 0.572737 | 0.105665 | -0.18843 | 0.252705 | C19orf36 |
| ILMN_1699473 | 0.0471 | 0.0107 | 0.312518 | 0.215483 | -0.46661 | -0.00717 | KIAA1967 |
| ILMN_1812545 | 0.023416 | 0.003901 | 0.481241 | -0.12474 | -0.18945 | 0.173799 | DMC1 |
| ILMN_1660426 | 0.014599 | 0.001911 | 0.136571 | 0.085812 | -0.26892 | 0.059989 | CPSF4 |
| ILMN_1762582 | 0.005773 | 3.80E-04 | 0.303111 | 0.073338 | -0.28037 | 0.073079 | ARNT |
| ILMN_1733538 | 0.033378 | 0.006529 | 0.423816 | 0.088362 | -0.25526 | 0.111348 | RGS10 |
| ILMN_1754757 | 0.003122 | 6.80E-05 | 0.198799 | 0.033774 | -0.45044 | 0.054986 | SCNN1D |
| ILMN_1704043 | 0.004014 | 1.48E-04 | 0.247353 | 0.013846 | -0.26622 | 0.303435 | LOC643905 |
| ILMN_2352245 | 0.044154 | 0.009734 | -0.26589 | -0.14516 | 0.338707 | -0.75798 | RASSF6 |
| ILMN_2296803 | 0.007543 | 6.41E-04 | 0.555731 | 0.08538 | -0.22945 | 1.041811 | CAPN3 |
| ILMN_1779632 | 0.008458 | 7.79E-04 | 0.395511 | -0.47221 | -0.14067 | 0.442421 | TMEM1 |
| ILMN_1742187 | 0.036355 | 0.007369 | -0.22249 | -0.06824 | 0.287045 | -0.12375 | MAN1A1 |
| ILMN_1752086 | 0.031869 | 0.006133 | -0.06381 | -0.16049 | -0.37246 | 0.262273 | C4orf41 |
| ILMN_1787627 | 0.024064 | 0.00404 | 0.003771 | 0.03467 | -0.23479 | 0.013341 | GPRASP1 |
| ILMN_1814856 | 0.004068 | 1.61E-04 | 0.296983 | 0.005941 | -0.26192 | 0.116202 | C9orf7 |
| ILMN_1809291 | 0.024553 | 0.004152 | 0.01619 | -0.55901 | -0.19209 | 0.473509 | TSPAN7 |
| ILMN_1709367 | 0.040786 | 0.008664 | 0.833546 | -0.23011 | -0.23925 | 0.104794 | FAM186B |
| ILMN_1734010 | 0.019864 | 0.003019 | 0.140985 | 0.02946 | -0.25126 | 0.03892 | C10orf118 |
| ILMN_3289895 | 0.023336 | 0.003884 | 0.131452 | -0.06015 | -0.21733 | 0.170357 | LOC151300 |
| ILMN_1755536 | 0.020692 | 0.003236 | 0.220563 | -0.11291 | -0.0676 | -0.05393 | PFDN5 |
| ILMN_2325763 | 0.008403 | 7.68E-04 | -0.13863 | 0.50245 | 0.33947 | -1.27816 | VCAM1 |
| ILMN_2156115 | 0.013958 | 0.001781 | 0.181191 | 0.168266 | -0.1794 | 0.005806 | IFT80 |
| ILMN_1760617 | 0.020411 | 0.003169 | 0.292438 | 0.129241 | -0.1588 | -0.07196 | IFT80 |
| ILMN_1736741 | 0.014514 | 0.001894 | 0.34513 | -0.10019 | -0.23748 | 0.168834 | RASSF8 |
| ILMN_2072391 | 7.15E-04 | 3.01E-06 | 0.575448 | -0.15288 | -0.41905 | 0.365663 | SNORD31 |
| ILMN_2316740 | 0.02562 | 0.004444 | 0.417013 | 0.006698 | -0.24034 | 0.080888 | FOXP4 |
| ILMN_1723381 | 0.041029 | 0.008749 | 0.806209 | 1.161922 | -0.0901 | 0.063986 | TSSK4 |
| ILMN_2120340 | 0.033784 | 0.006657 | 0.118954 | 0.104103 | -0.42855 | 0.164105 | RUVBL2 |
| ILMN_3304405 | 0.032397 | 0.006273 | 0.338766 | -0.18641 | 0.050851 | -0.21049 | NCRNA00081 |
| ILMN_1706783 | 0.008659 | 8.08E-04 | 0.415352 | -0.04386 | -0.49986 | 0.75103 | TAGLN |
| ILMN_2092118 | 0.040838 | 0.008685 | 0.410125 | -0.02764 | -0.25192 | 0.634018 | FPR1 |
| ILMN_1665280 | 0.002536 | 3.86E-05 | 0.322493 | -0.14092 | -0.25902 | 0.142825 | SPCS1 |
| ILMN_1807300 | 0.038832 | 0.008044 | -0.05705 | -0.02327 | 0.357739 | -0.17767 | PKD2 |
| ILMN_1726603 | 0.010306 | 0.001086 | 0.362125 | 0.044276 | -0.24076 | 0.011977 | ATP5I |
| ILMN_1759818 | 0.022957 | 0.003787 | 0.209088 | 0.054534 | -0.23082 | -0.174 | SORL1 |
| ILMN_1655039 | 0.008431 | 7.74E-04 | 0.347589 | 0.096294 | -0.23167 | 0.044892 | SLC27A4 |
| ILMN_1806099 | 0.003537 | 1.03E-04 | 0.179321 | 0.034708 | -0.293 | 0.170445 | RXFP4 |
| ILMN_2127298 | 0.002851 | 5.19E-05 | 0.457557 | -0.22143 | -0.17728 | 1.116442 | F2RL3 |
| ILMN_1813581 | 0.009735 | 9.85E-04 | 0.584941 | 0.0267 | -0.19769 | 0.116104 | CNR1 |
| ILMN_1792495 | 0.01721 | 0.002453 | 0.284086 | 0.07948 | -0.2939 | 0.095029 | AHNAK |
| ILMN_1714567 | 0.008449 | 7.76E-04 | 0.221681 | 0.085351 | -0.0458 | -0.36368 | AHNAK |
| ILMN_2125346 | 0.0152 | 0.00204 | 0.302461 | -0.04727 | -0.29098 | 0.078675 | MUC16 |
| ILMN_2396956 | 0.043381 | 0.009486 | -0.01444 | 0.228875 | -0.09743 | 0.24061 | AKAP13 |
| ILMN_1744235 | 0.025457 | 0.004399 | 0.788937 | 0.358362 | -0.3637 | 0.203518 | C20orf106 |
| ILMN_1679158 | 0.003258 | 7.64E-05 | 0.394912 | 0.043148 | -0.25267 | 0.142957 | FAAH2 |
| ILMN_1804854 | 0.013256 | 0.001647 | 0.068463 | 0.080908 | -0.00934 | -0.4032 | CTNNA1 |
| ILMN_1680130 | 0.027011 | 0.004784 | -0.09856 | -0.63091 | 0.092773 | -0.09889 | DYM |
| ILMN_2189222 | 0.006471 | 4.78E-04 | 0.127959 | 0.078692 | -0.31654 | 0.184207 | KLHL8 |
| ILMN_1732374 | 0.002073 | 2.47E-05 | 0.315107 | -0.06554 | -0.35564 | 0.120819 | C17orf41 |
| ILMN_1714896 | 0.002761 | 4.84E-05 | 0.301707 | 0.016789 | -0.2677 | 0.140124 | SART3 |
| ILMN_1741843 | 0.007024 | 5.65E-04 | 0.354977 | 0.042895 | -0.2445 | 0.22627 | RD3 |
| ILMN_1684391 | 0.00985 | 0.001007 | 0.168378 | -0.02591 | -0.32688 | -0.07838 | PLOD1 |
| ILMN_2250445 | 0.007806 | 6.79E-04 | 0.217971 | -0.00106 | -0.29091 | 0.064059 | SMTN |
| ILMN_2128048 | 0.017739 | 0.002569 | 0.224884 | 0.141774 | -0.27761 | -0.22989 | PCDHB3 |
| ILMN_1711765 | 0.002965 | 5.81E-05 | 0.766469 | -0.23359 | -0.24505 | 0.253516 | PSMD4 |
| ILMN_1712291 | 0.002491 | 3.67E-05 | -0.20527 | -0.15289 | 0.264747 | -1.21474 | MICALL2 |
| ILMN_1810584 | 0.002863 | 5.42E-05 | 0.290347 | 0.019219 | -0.26991 | -0.00959 | IL1R1 |
| ILMN_2134453 | 0.008082 | 7.20E-04 | 0.178507 | 0.007346 | -0.25856 | 0.172185 | FCGR3B |
| ILMN_1751666 | 0.029269 | 0.005426 | 0.420167 | 0.143447 | 0.060418 | 0.815231 | GPSM1 |
| ILMN_1707055 | 0.045614 | 0.010198 | 0.43614 | -0.03579 | -0.23079 | 0.431117 | GRRP1 |
| ILMN_2332440 | 0.009582 | 9.62E-04 | 0.209121 | 0.004463 | -0.24774 | 0.086728 | KCNMB2 |
| ILMN_2167805 | 0.026385 | 0.004641 | -0.16908 | 0.328969 | 0.264192 | -0.56747 | LUM |
| ILMN_1695446 | 0.005675 | 3.66E-04 | 0.446445 | 0.097418 | -0.25226 | 0.202666 | AMBP |
| ILMN_1709294 | 0.046095 | 0.010343 | 0.30403 | 0.145564 | -0.19507 | 0.155869 | CDCA8 |
| ILMN_2215119 | 0.01833 | 0.002695 | 0.406233 | -0.11186 | -0.1067 | -0.18185 | SYNJ2 |
| ILMN_1688417 | 0.002717 | 4.62E-05 | 0.456563 | 0.070049 | -0.30316 | 0.176788 | MYL2 |
| ILMN_1662086 | 0.0051 | 2.83E-04 | 0.46406 | 0.009824 | -0.19556 | 0.146853 | GUCA1A |
| ILMN_1777663 | 0.002073 | 2.46E-05 | 0.299024 | -0.07695 | -0.15436 | 0.43744 | TOP2B |
| ILMN_1690818 | 0.013217 | 0.00164 | 0.261928 | 0.071007 | -0.20153 | -0.02239 | AKAP5 |
| ILMN_2394750 | 0.049045 | 0.011336 | 0.102347 | 0.198932 | -0.08842 | -0.12916 | EXOC4 |
| ILMN_1735788 | 0.019525 | 0.002938 | -0.26211 | -0.48385 | 0.076567 | -1.38285 | TRIOBP |
| ILMN_2356745 | 0.029501 | 0.005485 | 0.202472 | 0.026696 | -0.24587 | 0.037342 | BTBD7 |
| ILMN_2246105 | 0.040463 | 0.00855 | 0.404439 | 0.0582 | -0.22407 | 0.219513 | C3orf23 |
| ILMN_1718971 | 0.016551 | 0.002312 | 0.054283 | -0.11491 | -0.21942 | 0.156304 | TTC7B |
| ILMN_2106449 | 0.04493 | 0.009976 | -0.5928 | 0.075501 | -0.20301 | 0.860189 | PLEKHF2 |
| ILMN_1687392 | 0.018231 | 0.002671 | 0.228146 | 0.015535 | -0.34947 | -0.15173 | NRK |
| ILMN_1652163 | 0.043118 | 0.009383 | -0.29042 | 0.018434 | 0.199788 | -0.34114 | DVL2 |
| ILMN_1740915 | 0.008208 | 7.39E-04 | 0.685674 | 0.090618 | -0.29585 | 0.14459 | SPATA3 |
| ILMN_3245954 | 0.010504 | 0.001125 | 0.373092 | -0.09959 | -0.29921 | 0.10708 | LOC285501 |
| ILMN_1755290 | 0.034223 | 0.00678 | 0.150291 | 0.364202 | -0.38046 | 0.018759 | CEP170 |
| ILMN_2294684 | 0.002586 | 4.11E-05 | 0.390537 | 0.043804 | -0.26733 | 0.082428 | CEP170 |
| ILMN_1693045 | 0.004255 | 1.88E-04 | 0.354409 | -0.03295 | -0.283 | 0.038627 | TMED1 |
| ILMN_1808765 | 0.013809 | 0.001747 | -0.06484 | 0.131973 | 0.205702 | 0.060704 | ZNF25 |
| ILMN_1656420 | 0.019776 | 0.002997 | -0.18223 | -0.39039 | 0.117882 | -0.47997 | MGC3020 |
| ILMN_2048607 | 0.006677 | 5.17E-04 | 0.140875 | -0.02561 | -0.31364 | 0.095841 | ANKRD9 |
| ILMN_2358801 | 0.006682 | 5.18E-04 | 0.434147 | 0.184163 | -0.26294 | 0.200313 | PLEKHG5 |
| ILMN_1815039 | 0.043844 | 0.009635 | 0.214215 | 0.043884 | -0.18947 | -0.07201 | C6orf153 |
| ILMN_1806500 | 0.013421 | 0.001675 | 0.134886 | 0.430708 | -0.21253 | 1.153584 | CPNE5 |
| ILMN_3242993 | 0.036198 | 0.007322 | 0.436762 | -0.08243 | -0.26032 | 0.474204 | ZFR2 |
| ILMN_3310451 | 0.022312 | 0.003633 | 0.514747 | -0.39646 | -0.32424 | -0.21662 | SNORD114-31 |
| ILMN_2155452 | 0.002621 | 4.29E-05 | 0.398361 | 0.092768 | -0.27597 | 0.151941 | HPR |
| ILMN_1760479 | 0.00733 | 6.11E-04 | 0.414932 | 0.130772 | -0.19953 | 0.175297 | MOS |
| ILMN_1686405 | 0.011187 | 0.001251 | 0.15613 | -0.54707 | -0.03885 | 0.102581 | KDR |
| ILMN_1747099 | 0.020466 | 0.003184 | 0.201931 | 0.007428 | -0.17123 | 0.106771 | LUC7L2 |
| ILMN_1709440 | 0.011604 | 0.001332 | 0.215787 | 0.052823 | -0.31522 | 0.145704 | CPM |
| ILMN_1706497 | 0.021806 | 0.003483 | 0.815777 | -0.06675 | 0.043045 | 0.9039 | P2RX2 |
| ILMN_2135944 | 0.004882 | 2.51E-04 | 0.350838 | -0.03609 | -0.29885 | 0.154154 | CT45A4 |
| ILMN_1757546 | 0.004063 | 1.58E-04 | 0.382072 | -0.03785 | -0.19747 | 0.130596 | OR1D2 |
| ILMN_1771118 | 0.004944 | 2.61E-04 | 0.337521 | -0.14576 | -0.16826 | 0.231371 | SLCO1B1 |
| ILMN_2129910 | 0.014283 | 0.001845 | 0.243436 | 0.009442 | -0.37539 | 0.074028 | SLC12A5 |
| ILMN_1741599 | 0.006404 | 4.70E-04 | 0.436616 | 0.03498 | -0.11622 | 0.005525 | MEMO1 |
| ILMN_1652777 | 0.020112 | 0.003094 | 0.464588 | -0.30134 | -0.28476 | 0.682503 | CDC42EP2 |
| ILMN_1789457 | 0.011797 | 0.001369 | 0.40385 | -0.13594 | -0.21017 | 0.100987 | GNL1 |
| ILMN_1782635 | 0.002067 | 2.40E-05 | -0.20894 | 0.342121 | 0.290856 | -0.42102 | YARS2 |
| ILMN_2105177 | 0.025317 | 0.004368 | 0.052036 | -0.48602 | 0.298109 | -0.74311 | SAMD8 |
| ILMN_1762899 | 0.001255 | 7.80E-06 | -0.21733 | 0.147743 | -0.15299 | 2.049959 | EGR1 |
| ILMN_1726239 | 0.005688 | 3.68E-04 | 0.351671 | -0.03861 | -0.17579 | 0.113226 | TBCA |
| ILMN_1659444 | 0.04782 | 0.010933 | 0.311457 | -0.25044 | -0.1017 | -0.0095 | KIF1B |
| ILMN_1735552 | 0.0114 | 0.001287 | 0.246579 | 0.014428 | -0.28606 | -0.02224 | KIF1B |
| ILMN_1661646 | 0.028966 | 0.005334 | 0.351331 | 0.044557 | -0.15869 | 0.242907 | BANK1 |
| ILMN_1723007 | 0.01365 | 0.001715 | 0.0648 | -0.02127 | 0.008352 | -0.50995 | ZCCHC9 |
| ILMN_1682544 | 0.015955 | 0.002195 | 0.138819 | -0.08931 | -0.33846 | 0.11361 | CLUL1 |
| ILMN_1763852 | 0.002178 | 2.80E-05 | 0.319585 | 0.006896 | -0.29478 | 0.103825 | ACACB |
| ILMN_1715113 | 0.024892 | 0.004254 | 0.350422 | 0.162397 | -0.11341 | -0.09908 | HARS2 |
| ILMN_2212590 | 0.012993 | 0.001598 | 0.040275 | -0.14895 | 0.273152 | -0.53146 | TMEM170A |
| ILMN_2293067 | 0.003279 | 8.08E-05 | 0.308651 | -0.28882 | -0.38093 | 0.167488 | PM20D1 |
| ILMN_1715583 | 0.039513 | 0.008273 | 0.295349 | 0.121176 | -0.33895 | 0.028536 | BOP1 |
| ILMN_1794949 | 0.02279 | 0.003747 | 0.22932 | -0.07559 | -0.34771 | 0.006133 | DSCR1L1 |
| ILMN_2091920 | 0.011424 | 0.00129 | 0.40979 | 0.377837 | -0.39587 | 0.605769 | PTCRA |
| ILMN_1730118 | 0.042387 | 0.009157 | -0.44962 | -0.07944 | 0.319989 | -0.26089 | ZNF644 |
| ILMN_1729801 | 0.003387 | 9.14E-05 | 0.519914 | 0.017879 | -0.23647 | 0.259198 | S100A8 |
| ILMN_1735463 | 0.03042 | 0.005734 | -0.30267 | -0.46764 | -0.05925 | -1.11565 | EXD3 |
| ILMN_1787885 | 0.024746 | 0.004212 | -0.14272 | -0.89983 | 0.124707 | -0.91698 | NUDT18 |
| ILMN_1706140 | 0.008858 | 8.51E-04 | 0.302869 | -0.06757 | -0.2497 | 0.072662 | PAGE2B |
| ILMN_1779095 | 0.003258 | 7.61E-05 | 0.4008 | 0.0659 | -0.31287 | 0.552463 | CEBPE |
| ILMN_1779897 | 0.011708 | 0.001355 | 0.246389 | -0.05963 | -0.33075 | 0.070963 | NPR1 |
| ILMN_1761733 | 0.012698 | 0.00154 | 0.196748 | 0.184402 | -0.19952 | -0.16296 | HLA-DMB |
| ILMN_1659148 | 0.008931 | 8.64E-04 | 0.329074 | -0.06749 | -0.2136 | 0.080744 | ARL8A |
| ILMN_1731446 | 0.043935 | 0.009667 | 0.35462 | 0.162379 | -0.30794 | 0.02268 | COL11A1 |
| ILMN_1747205 | 0.004646 | 2.28E-04 | 0.309254 | -0.0261 | -0.29037 | -0.07964 | JDP2 |
| ILMN_1681984 | 0.022715 | 0.003726 | -0.62796 | 0.083145 | 0.324482 | -0.60266 | GALNT10 |
| ILMN_1782538 | 0.025737 | 0.004474 | 0.150979 | 0.050562 | -0.29025 | -0.05084 | VIM |
| ILMN_2058251 | 0.029034 | 0.005353 | 0.141271 | 0.176478 | -0.13671 | -0.19575 | VIM |
| ILMN_1693430 | 0.003944 | 1.42E-04 | 0.442757 | -0.00109 | -0.22207 | 0.140191 | NME1-NME2 |
| ILMN_2319994 | 0.006112 | 4.22E-04 | 0.289822 | -0.02538 | -0.3425 | 0.07373 | RPL3 |
| ILMN_1787877 | 0.006222 | 4.38E-04 | 0.243111 | 0.15392 | -0.32211 | 0.180759 | CCNT2 |
| ILMN_1705568 | 0.003963 | 1.44E-04 | 0.342035 | -0.07129 | -0.24845 | 0.183574 | BCAP29 |
| ILMN_1704063 | 0.005635 | 3.61E-04 | -0.08539 | 0.231698 | -0.30648 | 0.132616 | KCNH3 |
| ILMN_2366490 | 0.033562 | 0.006586 | 0.410752 | 0.067419 | -0.10975 | 0.017871 | ZNF706 |
| ILMN_1772674 | 0.003753 | 1.24E-04 | 0.538226 | 0.056905 | -0.24159 | 0.121896 | IL21 |
| ILMN_2382471 | 0.006718 | 5.24E-04 | 0.214911 | 0.112368 | -0.22986 | 0.100073 | DUSP15 |
| ILMN_1781870 | 0.026352 | 0.00463 | 0.326426 | 0.031863 | -0.25705 | -0.03064 | RBM8A |
| ILMN_2138005 | 0.006718 | 5.24E-04 | 0.22981 | 0.11648 | -0.18843 | 0.255501 | CHL1 |
| ILMN_3251322 | 0.006311 | 4.51E-04 | 0.439298 | -0.08942 | -0.28015 | 0.205552 | ADAMTS7 |
| ILMN_1776188 | 0.02038 | 0.003156 | 0.221099 | 0.029041 | -0.24906 | 0.146172 | MAP1LC3A |
| ILMN_3224926 | 0.008801 | 8.40E-04 | -0.06217 | -0.09746 | 0.506198 | -0.614 | RBM47 |
| ILMN_3310176 | 0.011467 | 0.001297 | 0.238369 | -0.08592 | -0.53325 | -0.14237 | MIR593 |
| ILMN_3235096 | 0.015869 | 0.002177 | 0.513087 | -0.17203 | -0.37847 | 0.462833 | SNORA28 |
| ILMN_1703695 | 0.04882 | 0.01126 | 0.361687 | -0.44397 | -0.15155 | 0.302237 | C19orf12 |
| ILMN_1754600 | 0.005131 | 2.91E-04 | 0.031291 | 0.02822 | 0.541359 | -0.67226 | FNBP1L |
| ILMN_1812461 | 0.008395 | 7.67E-04 | 0.482558 | 0.317409 | -0.48681 | 1.281582 | WISP2 |
| ILMN_1664028 | 0.012098 | 0.001419 | 0.361291 | 0.108925 | -0.18267 | 0.075948 | CENPB |
| ILMN_1810977 | 0.003279 | 8.16E-05 | 0.196447 | 0.054021 | -0.1975 | 0.349917 | CDV3 |
| ILMN_2339202 | 0.009309 | 9.21E-04 | 0.08873 | -0.13904 | 0.428728 | -0.6479 | KTN1 |
| ILMN_3308956 | 0.011318 | 0.001274 | 0.505396 | 0.06395 | -0.22764 | 0.090071 | MIR371 |
| ILMN_1672417 | 0.033052 | 0.006445 | -0.3036 | 0.30573 | -0.43927 | 0.193538 | PTPRCAP |
| ILMN_2275560 | 0.006979 | 5.60E-04 | 0.234981 | -0.0432 | -0.27737 | 0.119651 | NDUFAF3 |
| ILMN_3230337 | 0.047218 | 0.010742 | 0.173998 | -0.04879 | -0.04905 | -0.61309 | LOC100132707 |
| ILMN_2226519 | 0.006173 | 4.31E-04 | 0.41413 | 0.049596 | -0.3223 | 0.079641 | CYP2D7P1 |
| ILMN_1718265 | 0.026802 | 0.004743 | 0.280989 | 0.078735 | -0.1819 | -0.05022 | ATG5 |
| ILMN_1755811 | 0.008761 | 8.30E-04 | 0.367457 | -0.25347 | -0.10253 | -0.00623 | FBXO4 |
| ILMN_1783287 | 0.005922 | 3.97E-04 | 0.232397 | 0.032809 | -0.35011 | -0.16388 | S100A14 |
| ILMN_3237165 | 0.009225 | 9.07E-04 | 0.323476 | -0.05851 | -0.31232 | 0.083445 | LOC100128164 |
| ILMN_1804357 | 0.008858 | 8.54E-04 | 0.372246 | -0.0696 | -0.29349 | 0.12112 | GNG4 |
| ILMN_2371964 | 0.018156 | 0.002651 | 0.252749 | 0.039148 | -0.29648 | -0.15079 | MRPS12 |
| ILMN_2248655 | 0.003949 | 1.42E-04 | 0.307992 | -0.0139 | -0.29969 | 0.163972 | RGS3 |
| ILMN_1702384 | 0.002754 | 4.76E-05 | 0.443043 | -0.0319 | -0.18645 | 0.0661 | ZNF706 |
| ILMN_1674282 | 0.0081 | 7.23E-04 | 0.262527 | -0.05205 | -0.24466 | 0.146845 | PPARD |
| ILMN_1721349 | 0.04695 | 0.010631 | -0.25065 | 0.101952 | 0.30461 | -0.76488 | MAGT1 |
| ILMN_3247080 | 0.008367 | 7.62E-04 | 0.280381 | 0.250031 | -0.24727 | 0.063578 | LOC100133612 |
| ILMN_3250659 | 0.006944 | 5.54E-04 | -0.51161 | 0.144038 | 0.199423 | -0.84233 | SKA2 |
| ILMN_1807807 | 0.020429 | 0.003176 | 0.265028 | 0.056613 | -0.20564 | 0.087531 | SKA2 |
| ILMN_1717294 | 0.031791 | 0.006112 | 0.281622 | -0.48267 | -0.0271 | -0.46016 | PTPN3 |
| ILMN_2254123 | 0.029679 | 0.005531 | 0.140769 | 0.144119 | 0.392857 | -0.23806 | IFT81 |
| ILMN_1798433 | 0.026138 | 0.004584 | 0.041404 | 0.115211 | -0.49633 | 0.967799 | LNPEP |
| ILMN_3234093 | 0.0051 | 2.82E-04 | 0.091785 | -0.02465 | -0.28455 | 0.105724 | SNX18 |
| ILMN_3240538 | 0.013233 | 0.001643 | 0.170902 | 0.018799 | -0.27845 | 0.092627 | C19orf38 |
| ILMN_1813572 | 0.005411 | 3.32E-04 | 0.282291 | 0.130255 | -0.24835 | 0.079376 | IL16 |
| ILMN_2320336 | 0.006583 | 4.99E-04 | 0.296846 | 0.073175 | -0.15733 | 0.189267 | CLK3 |
| ILMN_1733807 | 0.004639 | 2.27E-04 | 0.435127 | 0.041219 | -0.15316 | 0.231252 | IGF2BP1 |
| ILMN_2335109 | 0.03208 | 0.006189 | 0.27768 | -0.1178 | -0.19111 | 0.074402 | RNF12 |
| ILMN_1743402 | 0.002424 | 3.51E-05 | -0.01311 | 0.746735 | 0.647148 | -1.66962 | SIX4 |
| ILMN_1729318 | 0.010049 | 0.001037 | 0.100524 | -0.34964 | -0.20867 | 0.443611 | TOR1AIP1 |
| ILMN_1694177 | 0.00256 | 4.03E-05 | 0.45275 | -0.03935 | -0.21864 | 0.025456 | PCNA |
| ILMN_1760513 | 0.0392 | 0.008172 | -0.91868 | -0.01008 | 0.493564 | -0.08897 | DYDC2 |
| ILMN_1771224 | 0.012416 | 0.001482 | 0.074354 | 0.018955 | -0.06466 | 0.2627 | SKA1 |
| ILMN_1779370 | 0.008719 | 8.20E-04 | 0.565795 | -0.04961 | -0.15767 | 0.220351 | ARHGEF9 |
| ILMN_1764778 | 0.004063 | 1.58E-04 | 0.202791 | 0.056779 | -0.20792 | 0.203263 | NASP |
| ILMN_1780551 | 0.039506 | 0.008268 | 0.418463 | 0.196035 | -0.30881 | 0.074348 | ADRA1A |
| ILMN_1815773 | 0.041002 | 0.008736 | 0.40916 | 0.029321 | -0.18917 | 0.087018 | TMEM190 |
| ILMN_2348503 | 0.010323 | 0.001092 | 0.26761 | -0.52554 | -0.20111 | 0.624087 | PPIE |
| ILMN_2251766 | 0.028324 | 0.005154 | 0.159528 | -0.16547 | -0.25252 | 0.106369 | IL1R2 |
| ILMN_2360710 | 0.048011 | 0.010997 | 0.131232 | 0.007989 | -0.18787 | 0.024968 | TPM1 |
| ILMN_2170209 | 0.048433 | 0.011137 | 0.275146 | 0.406473 | -0.185 | -0.48194 | RASD2 |
| ILMN_1810147 | 0.003631 | 1.14E-04 | -0.08269 | -0.31272 | 0.181819 | -0.91911 | ZNF524 |
| ILMN_1709645 | 0.007413 | 6.21E-04 | 0.29938 | -0.04301 | -0.27537 | 0.093285 | CBX2 |
| ILMN_1738632 | 0.003122 | 6.91E-05 | -0.29189 | -0.03338 | 0.195981 | -0.01452 | PRKAR1A |
| ILMN_1682180 | 0.002536 | 3.83E-05 | 0.45293 | 0.038908 | -0.14353 | -0.08641 | VCPIP1 |
| ILMN_1701614 | 0.008007 | 7.09E-04 | 0.355437 | 0.085416 | -0.23074 | -0.02596 | CDC73 |
| ILMN_2249677 | 0.012644 | 0.001533 | 0.360414 | 0.195452 | -0.26517 | 0.143272 | LSR |
| ILMN_1685313 | 0.008009 | 7.10E-04 | 0.601798 | 0.135348 | -0.70478 | 0.53207 | PLEC1 |
| ILMN_1669323 | 0.015236 | 0.002051 | 0.262348 | -0.19922 | -0.27559 | 0.353263 | BACE2 |
| ILMN_1675413 | 0.030619 | 0.005787 | 1.061944 | -0.59762 | 0.123319 | 1.16653 | ENPP7 |
| ILMN_2105539 | 0.00154 | 1.30E-05 | 0.398534 | 0.010188 | -0.32532 | 0.194944 | FZD10 |
| ILMN_1701293 | 0.003585 | 1.09E-04 | 0.408852 | -0.22486 | -0.07565 | 0.177819 | COX7A2 |
| ILMN_1706959 | 0.033378 | 0.006531 | 0.300701 | -0.00949 | -0.16584 | -0.20492 | TIMM22 |
| ILMN_1798636 | 0.027133 | 0.004817 | 0.542499 | -0.05377 | -0.26051 | 0.941376 | RPL32 |
| ILMN_2372040 | 0.010341 | 0.001095 | 0.481154 | 0.205067 | -0.23065 | -0.1005 | MTP18 |
| ILMN_1666361 | 0.046435 | 0.010456 | 1.454937 | 0.243624 | -0.0225 | 1.224882 | SLC23A2 |
| ILMN_1786771 | 0.004058 | 1.55E-04 | 0.4268 | -0.07889 | -0.30661 | 4.37E-04 | LOC387646 |
| ILMN_1728440 | 0.005147 | 2.94E-04 | 0.443583 | 0.007123 | -0.21783 | 0.195475 | KRTAP6-3 |
| ILMN_1660698 | 0.011023 | 0.001224 | 0.315689 | 0.006815 | -0.23722 | -0.01382 | GTPBP8 |
| ILMN_2396338 | 0.015318 | 0.002074 | 0.944106 | 0.288758 | -0.22242 | 0.041251 | BANP |
| ILMN_1679185 | 0.00806 | 7.16E-04 | -0.24137 | -0.10403 | 0.331082 | -0.21397 | LEF1 |
| ILMN_1749109 | 0.024552 | 0.00415 | 0.347258 | 0.001978 | -0.14765 | -0.05643 | PSAP |
| ILMN_1789419 | 0.03429 | 0.006798 | -0.37698 | -0.67096 | 0.288546 | -0.12722 | EXOC3 |
| ILMN_1663699 | 0.003706 | 1.21E-04 | 0.330873 | -0.06444 | -0.24882 | 0.196633 | SLCO3A1 |
| ILMN_1760741 | 0.03257 | 0.006318 | -0.16032 | -0.03842 | 0.209692 | -0.46005 | NDUFA9 |
| ILMN_1798700 | 0.008431 | 7.73E-04 | 0.36791 | 0.063986 | -0.25377 | 0.014089 | CHRNA1 |
| ILMN_1812328 | 0.003446 | 9.56E-05 | 0.238774 | -0.03442 | -0.33042 | 0.160328 | TBL1Y |
| ILMN_2287253 | 0.005956 | 4.02E-04 | 0.352711 | 0.070246 | -0.31776 | 0.068827 | PFKL |
| ILMN_1735275 | 0.004882 | 2.50E-04 | 0.118327 | -0.21658 | 0.28159 | -0.87052 | WDSUB1 |
| ILMN_1760575 | 0.038504 | 0.007954 | -0.01458 | -0.16219 | 0.009557 | 0.513265 | PTP4A1 |
| ILMN_2258383 | 0.007922 | 6.97E-04 | -0.6697 | 0.123457 | 0.25175 | -0.59649 | BAX |
| ILMN_3246140 | 0.006875 | 5.46E-04 | 0.134711 | 0.0561 | -0.26816 | 0.134169 | ZC3H13 |
| ILMN_2118472 | 0.039978 | 0.008398 | 0.250552 | 0.036725 | -0.28936 | -0.03758 | C10orf58 |
| ILMN_1718988 | 0.011667 | 0.001346 | 0.22734 | -0.09011 | -0.12778 | 0.072089 | DAZAP2 |
| ILMN_2189037 | 0.013397 | 0.001671 | 0.244639 | -0.01157 | -0.18907 | -0.32228 | WDR52 |
| ILMN_1747622 | 0.012745 | 0.001549 | 0.636751 | 0.747994 | 0.258099 | 0.379574 | CD33 |
| ILMN_2206953 | 0.004192 | 1.80E-04 | 0.358003 | -0.04069 | -0.17794 | 0.132482 | PLCL1 |
| ILMN_2191003 | 0.02267 | 0.003713 | 1.067958 | 0.195422 | -0.12788 | 0.156496 | KRTAP5-1 |
| ILMN_3187680 | 0.010796 | 0.001175 | 0.263155 | 0.082664 | -0.26659 | -0.1518 | ACCS |
| ILMN_1731113 | 0.01785 | 0.002588 | 7.65E-04 | 0.157886 | -0.14525 | 0.217519 | ZBTB43 |
| ILMN_1775111 | 0.019906 | 0.003036 | 0.521988 | 0.052244 | -0.30082 | -0.10379 | SND1 |
| ILMN_2273103 | 0.042217 | 0.009114 | -0.5579 | -0.19548 | -0.01016 | -0.02138 | ELK4 |
| ILMN_1771805 | 0.004785 | 2.40E-04 | 0.028211 | 0.122407 | -0.20831 | 0.326092 | ELK4 |
| ILMN_1660886 | 0.00141 | 1.09E-05 | 0.308927 | -0.10061 | -0.43464 | 0.328693 | ELK4 |
| ILMN_3309428 | 0.005042 | 2.75E-04 | 0.778128 | -0.17124 | -0.32265 | 1.12451 | SNORD114-17 |
| ILMN_1657373 | 0.024822 | 0.004227 | 0.338236 | -0.10945 | -0.17379 | -0.18301 | LEPREL1 |
| ILMN_1735762 | 0.043075 | 0.009372 | 0.443542 | 0.03811 | -0.12395 | -0.12802 | NPNT |
| ILMN_3310266 | 0.005298 | 3.17E-04 | 0.383712 | -0.04352 | -0.24735 | 0.059545 | MIR548O |
| ILMN_1664499 | 0.011187 | 0.00125 | 0.608957 | 0.070614 | -0.2941 | 0.050012 | PLEKHG7 |
| ILMN_1731610 | 0.002785 | 4.92E-05 | -0.78613 | -0.9891 | -0.00865 | 0.904061 | ABLIM1 |
| ILMN_2320330 | 0.014638 | 0.00192 | 0.367892 | -0.07634 | -0.2852 | -0.18792 | MAL |
| ILMN_1719695 | 0.021809 | 0.003485 | 0.152984 | 0.101652 | -0.28077 | 0.554243 | NFKBIZ |
| ILMN_1772697 | 0.02463 | 0.004178 | 0.22675 | -0.18046 | -0.27652 | -0.03027 | KREMEN1 |
| ILMN_2320853 | 0.008872 | 8.57E-04 | 0.242648 | -0.08722 | -0.21389 | 0.122089 | UBE2D3 |
| ILMN_2233334 | 0.007447 | 6.26E-04 | 0.400759 | 0.004873 | -0.11569 | 0.180131 | MYH16 |
| ILMN_1762843 | 0.003641 | 1.16E-04 | 0.381225 | 0.193129 | -0.26191 | 0.167886 | FAM163B |
| ILMN_1748563 | 0.006666 | 5.15E-04 | 0.663402 | -0.06377 | -0.3147 | 0.18675 | LOC85391 |
| ILMN_1809477 | 0.012978 | 0.001595 | 0.146954 | -0.11195 | -0.27458 | 0.092062 | CARHSP1 |
| ILMN_1677511 | 0.018941 | 0.002815 | 0.407395 | 0.046704 | -0.2762 | 0.063643 | PTGS2 |
| ILMN_1772208 | 0.032566 | 0.006313 | 0.630616 | 0.701973 | -0.48661 | -0.0246 | CCDC88B |
| ILMN_2300636 | 0.01149 | 0.001301 | 0.188869 | -0.06377 | -0.31116 | 0.340639 | HMGXB4 |
| ILMN_1761722 | 0.012953 | 0.001591 | -0.00214 | -0.24114 | -0.05164 | -1.06438 | ZNF579 |
| ILMN_1688458 | 0.007413 | 6.22E-04 | 0.255391 | -0.03646 | -0.29824 | 0.178023 | CELA2B |
| ILMN_1680314 | 0.029485 | 0.005481 | 0.233969 | -0.21348 | -0.22491 | 0.273635 | TXN |
| ILMN_1748271 | 0.031826 | 0.006123 | 0.244095 | -0.2797 | -0.15291 | 0.271132 | ATF2 |
| ILMN_1756308 | 0.011491 | 0.001302 | 0.293933 | 0.087665 | 0.087447 | -0.64923 | NAE1 |
| ILMN_1737953 | 0.008458 | 7.79E-04 | 0.256277 | -0.07532 | -0.38368 | 0.207444 | MKS1 |
| ILMN_1787657 | 0.001337 | 8.83E-06 | -0.29675 | -0.05544 | 0.450195 | -0.85013 | CLDN12 |
| ILMN_2138662 | 0.022643 | 0.003707 | 0.384816 | 0.196471 | -0.11736 | 0.136393 | SORCS3 |
| ILMN_1661197 | 0.024147 | 0.004061 | 0.22052 | -0.71412 | 0.122226 | 0.605642 | CLCF1 |
| ILMN_2134224 | 0.004063 | 1.57E-04 | 0.116624 | 0.008781 | -0.44581 | -0.09488 | ATP13A1 |
| ILMN_1734660 | 0.01132 | 0.001275 | 0.371821 | 0.030746 | -0.20265 | 0.180194 | ICAM4 |
| ILMN_2304512 | 0.007896 | 6.92E-04 | 0.654273 | 0.029328 | -0.29723 | 0.252764 | SAA1 |
| ILMN_2059452 | 0.049388 | 0.01144 | 0.099262 | 0.156033 | -0.1831 | 0.23514 | SLC12A2 |
| ILMN_1653438 | 0.042758 | 0.009263 | 0.266605 | 0.085978 | -0.14124 | 0.034492 | PHF14 |
| ILMN_2401770 | 0.012217 | 0.001441 | -0.36328 | -0.15248 | 0.157422 | -0.58362 | PHF14 |
| ILMN_1736936 | 0.005131 | 2.93E-04 | 0.371793 | -0.2972 | -0.34621 | 0.318549 | FOXI1 |
| ILMN_1687431 | 0.011491 | 0.001302 | 0.421292 | 0.009212 | -0.24739 | 0.067798 | RNF6 |
| ILMN_1674665 | 0.019931 | 0.003044 | 0.117794 | -0.04093 | -0.25525 | -0.01541 | CPNE2 |
| ILMN_2404182 | 8.85E-04 | 4.13E-06 | 0.439236 | -0.02274 | -0.27768 | 0.161721 | DUOX1 |
| ILMN_1676864 | 0.005578 | 3.53E-04 | 0.655837 | -0.00862 | -0.31247 | 0.50514 | DDX31 |
| ILMN_1797668 | 0.016511 | 0.002304 | 0.061287 | -0.08044 | -0.23926 | 0.104944 | INSM1 |
| ILMN_1788955 | 0.011765 | 0.001363 | 0.336048 | 0.028825 | -0.17373 | -0.03273 | PDLIM1 |
| ILMN_1687751 | 0.030341 | 0.005702 | 0.11825 | 0.033976 | -0.29443 | 0.09412 | BAALC |
| ILMN_2310589 | 0.016782 | 0.002365 | 0.218186 | 0.049211 | -0.25813 | -0.04235 | DIABLO |
| ILMN_1791099 | 0.015136 | 0.002024 | 0.415193 | -0.28861 | -0.43595 | -0.2031 | TRIM6-TRIM34 |
| ILMN_1805366 | 0.039187 | 0.008167 | 0.122687 | 0.162539 | -0.28161 | 0.123592 | TBC1D8B |
| ILMN_1740010 | 0.01078 | 0.001172 | 0.222183 | -0.01844 | -0.24459 | 0.042667 | PCNX |
| ILMN_1715259 | 0.029286 | 0.005432 | 0.543151 | 0.240739 | -0.17511 | 0.281892 | OR6V1 |
| ILMN_1758214 | 0.027584 | 0.004943 | 0.103402 | -0.02049 | -0.22088 | 0.045349 | RARS2 |
| ILMN_1704672 | 0.022034 | 0.003546 | -0.25056 | -0.3826 | 0.245 | -0.44053 | OBFC2B |
| ILMN_1699651 | 7.13E-09 | 6.74E-13 | 0.929199 | 0.125955 | -0.45465 | 2.38954 | IL6 |
| ILMN_2266309 | 0.040473 | 0.008561 | 0.243923 | 0.006278 | -0.15863 | -0.00382 | CEPT1 |
| ILMN_1683263 | 0.02762 | 0.004952 | 0.25265 | 0.057508 | 0.235595 | -0.69193 | TSPAN8 |
| ILMN_1665515 | 0.002298 | 3.07E-05 | 0.310747 | -0.31113 | -0.32447 | 0.374439 | MGC4677 |
| ILMN_1655497 | 0.023194 | 0.003846 | 0.287088 | -0.04306 | -0.19075 | 0.103266 | EIF4B |
| ILMN_1785635 | 0.022977 | 0.003798 | -0.23003 | -0.29458 | 0.495192 | -1.04915 | BRD3 |
| ILMN_1700628 | 0.015078 | 0.002011 | 0.221231 | -0.05959 | -0.19715 | 0.202509 | DDX24 |
| ILMN_2144064 | 0.044225 | 0.009758 | 0.184996 | -0.04137 | 0.246539 | -0.18061 | RNF138P1 |
| ILMN_1763523 | 0.002373 | 3.21E-05 | 0.208847 | -0.02241 | -0.38014 | 0.074046 | HARS |
| ILMN_2185984 | 0.046104 | 0.010349 | 0.23006 | -0.21353 | -0.1213 | 0.154993 | SASH1 |
| ILMN_2219556 | 0.048324 | 0.011101 | -0.15858 | -0.27994 | 0.273487 | 0.417498 | ISCA1 |
| ILMN_1741021 | 0.004377 | 2.01E-04 | 0.156986 | 0.151311 | -0.45223 | 0.740901 | CH25H |
| ILMN_1753347 | 0.006957 | 5.58E-04 | 1.009721 | -0.0433 | -0.19435 | 0.151021 | DEFA4 |
| ILMN_1812759 | 0.00326 | 7.68E-05 | 0.392547 | -0.02915 | -0.27895 | 0.1471 | GCH1 |
| ILMN_2342579 | 0.027893 | 0.005025 | -0.28225 | 0.898687 | -0.53207 | 0.455213 | IL7R |
| ILMN_2334210 | 0.022288 | 0.003626 | -0.18316 | 0.170357 | -0.31004 | 0.370423 | ITGB4 |
| ILMN_1654246 | 0.044047 | 0.009704 | -0.10538 | -0.41029 | 0.324948 | -0.36613 | SIRT6 |
| ILMN_1666022 | 0.010118 | 0.001056 | 0.255458 | 0.015679 | -0.20618 | 0.764366 | TNFRSF10D |
| ILMN_2178186 | 0.008524 | 7.87E-04 | -0.11953 | 0.515225 | 0.439937 | -0.54342 | PIGW |
| ILMN_1750294 | 0.022977 | 0.003798 | 0.370512 | -0.07026 | -0.20206 | 0.150471 | ADAM7 |
| ILMN_1777541 | 0.006356 | 4.60E-04 | 0.520438 | 0.043609 | -0.27165 | 0.185663 | KLK6 |
| ILMN_1803813 | 0.014828 | 0.00196 | 0.499182 | -0.05046 | -0.17169 | -0.35578 | ASTE1 |
| ILMN_1727252 | 0.002502 | 3.71E-05 | 0.341389 | 0.043472 | -0.30836 | 0.10233 | WNT8A |
| ILMN_1789233 | 0.010399 | 0.001104 | 0.266949 | 0.069098 | -0.15513 | 0.086139 | VPS37C |
| ILMN_2409754 | 0.005846 | 3.88E-04 | -0.68866 | -0.62977 | 0.274255 | -0.44436 | PRR5 |
| ILMN_1777322 | 0.029103 | 0.005374 | -0.03326 | -0.1847 | 0.326492 | -0.04945 | FAM91A1 |
| ILMN_1760332 | 0.018941 | 0.002818 | 0.052721 | 0.155694 | -0.31585 | 0.089979 | PIGL |
| ILMN_1659762 | 0.001061 | 5.36E-06 | 0.265254 | 0.052004 | -0.24589 | 0.203965 | BTF3 |
| ILMN_1675057 | 0.025828 | 0.004506 | 0.40203 | -0.22247 | -0.31341 | 0.135134 | DRD2 |
| ILMN_1809141 | 0.025988 | 0.004551 | 0.173828 | 0.087877 | -0.2112 | -0.28512 | ING4 |
| ILMN_1815548 | 0.003122 | 6.89E-05 | 0.344259 | -0.03284 | -0.27138 | 0.10154 | OR52D1 |
| ILMN_1723536 | 0.00943 | 9.42E-04 | 0.078163 | 0.1388 | 0.470918 | -0.54667 | USP33 |
| ILMN_2212823 | 0.015428 | 0.002092 | -0.28907 | -0.13637 | 0.087282 | -1.17947 | ZNF577 |
| ILMN_1677197 | 0.00581 | 3.85E-04 | 0.488747 | 0.027357 | -0.21613 | 0.211881 | RARA |
| ILMN_1659206 | 0.001086 | 5.67E-06 | 0.104658 | 0.159746 | -0.21966 | 0.499101 | RARA |
| ILMN_2279635 | 0.02423 | 0.004084 | 0.300595 | -0.0544 | -0.25492 | 0.048252 | EIF4G2 |
| ILMN_2380946 | 0.004708 | 2.33E-04 | -0.17448 | 0.157619 | 0.348395 | -0.7284 | EIF4G2 |
| ILMN_1799710 | 0.011943 | 0.001393 | 0.458273 | -0.04215 | -0.20604 | 0.047254 | CCDC96 |
| ILMN_1763500 | 0.031887 | 0.006141 | 0.15193 | 0.035574 | -0.19471 | 0.190992 | GPR82 |
| ILMN_1656017 | 0.02452 | 0.00414 | 0.550475 | 0.261145 | -0.37606 | 0.035665 | NRF1 |
| ILMN_1749515 | 0.025764 | 0.004483 | 0.427926 | 0.062071 | -0.18897 | 0.123463 | CELA3B |
| ILMN_1693664 | 0.048087 | 0.011023 | 0.228326 | -0.10536 | -0.16746 | 0.110078 | POMGNT1 |
| ILMN_1663160 | 0.014354 | 0.001866 | -0.17675 | 0.106938 | 0.207968 | -0.83813 | ZNF337 |
| ILMN_1718331 | 0.014513 | 0.001893 | 0.366371 | -0.14282 | -0.25131 | 0.145288 | MMP16 |
| ILMN_1757732 | 0.017293 | 0.00247 | -0.24721 | -0.0191 | 0.439543 | 0.892336 | OSGIN2 |
| ILMN_1670000 | 0.028601 | 0.005229 | 0.158462 | 0.040394 | -0.19937 | 0.024349 | DCAF6 |
| ILMN_1660125 | 0.004882 | 2.51E-04 | 0.246571 | 0.114965 | -0.31617 | 0.182418 | SFMBT2 |
| ILMN_2335304 | 0.014971 | 0.001984 | 0.281045 | 0.079434 | -0.24269 | -0.02911 | WDR35 |
| ILMN_1689718 | 0.005404 | 3.30E-04 | 0.714273 | 0.025115 | -0.19888 | 0.271188 | IL17RE |
| ILMN_2276952 | 0.004651 | 2.29E-04 | 0.191937 | -0.11774 | -0.2898 | 0.824667 | TSC22D3 |
| ILMN_2144401 | 0.034214 | 0.006775 | 0.666956 | -0.24539 | -0.15353 | 0.687345 | GLB1L2 |
| ILMN_1726250 | 0.005877 | 3.92E-04 | 0.490203 | 0.09122 | -0.21458 | 0.089009 | CPAMD8 |
| ILMN_1658310 | 0.003312 | 8.42E-05 | 0.408365 | 0.172288 | -0.19319 | 0.246235 | PCYT1A |
| ILMN_3250032 | 0.047728 | 0.010894 | 0.447442 | 0.059795 | -0.10495 | -0.18723 | XPR1 |
| ILMN_3249963 | 0.003087 | 6.64E-05 | 0.387734 | 0.079235 | -0.27123 | 0.145648 | PCBP2 |
| ILMN_3249059 | 0.048211 | 0.011057 | 0.248671 | 0.001222 | -0.27594 | 0.020813 | SNHG12 |
| ILMN_1658636 | 0.002024 | 2.31E-05 | 0.338813 | -0.11109 | 0.047367 | -0.95708 | MRE11A |
| ILMN_2271379 | 0.009786 | 9.95E-04 | 0.314464 | 0.21291 | -0.31257 | -0.09841 | SDCCAG3 |
| ILMN_1680054 | 0.036204 | 0.007325 | 0.554289 | 0.48084 | -0.47843 | 0.411776 | LAMB3 |
| ILMN_1745788 | 0.006345 | 4.58E-04 | 0.198954 | 0.23891 | -0.23067 | -0.28146 | CX3CR1 |
| ILMN_2073202 | 0.00358 | 1.08E-04 | 0.109103 | 0.013023 | -0.32316 | 0.129183 | MEI1 |
| ILMN_1687140 | 0.020112 | 0.003092 | 0.426388 | 0.205787 | -0.26061 | 0.407952 | STARD7 |
| ILMN_3245973 | 0.029817 | 0.005571 | 0.20469 | 0.130659 | -0.189 | 0.018647 | MSL1 |
| ILMN_1667980 | 0.010939 | 0.001205 | 0.648953 | 0.103458 | -0.21123 | 0.123425 | CACNA1S |
| ILMN_1757437 | 0.022322 | 0.003637 | -0.34162 | 0.238948 | 0.443998 | 0.424598 | UMPS |
| ILMN_1676288 | 0.012403 | 0.001479 | -0.30868 | 0.230323 | 0.099058 | -0.77495 | ACBD4 |
| ILMN_1801869 | 0.009106 | 8.90E-04 | -0.31401 | 0.163293 | 0.096909 | -0.82224 | WDR75 |
| ILMN_1726842 | 0.033438 | 0.006547 | 0.161748 | -0.10714 | 0.219525 | -0.55719 | TYW3 |
| ILMN_1703511 | 0.016511 | 0.002304 | 0.504152 | -0.00522 | -0.41004 | 0.040371 | PDZRN3 |
| ILMN_1657729 | 0.025303 | 0.004364 | 0.404885 | 0.107555 | -0.10478 | 0.108857 | ZNF254 |
| ILMN_2045351 | 0.013547 | 0.001694 | 0.245099 | -0.09622 | -0.33429 | 0.251063 | SPINK9 |
| ILMN_1684038 | 0.028093 | 0.00508 | 0.246887 | 0.048175 | -0.1485 | 0.118927 | CLIP1 |
| ILMN_2153029 | 0.0051 | 2.84E-04 | 0.587086 | 0.077737 | -0.19299 | 0.148684 | CGB |
| ILMN_1778536 | 0.021439 | 0.003398 | 0.262189 | -0.06955 | -0.2652 | 0.099956 | BTLA |
| ILMN_1759628 | 0.008524 | 7.88E-04 | 0.217973 | 0.020969 | -0.18228 | 0.070889 | ATP1B3 |
| ILMN_1783304 | 0.025472 | 0.004405 | 0.189602 | 0.004434 | -0.20133 | -0.02677 | ATP1B3 |
| ILMN_1772957 | 0.036023 | 0.007273 | 0.018106 | 0.001828 | 5.86E-04 | 0.687326 | FOXRED2 |
| ILMN_1776263 | 0.046436 | 0.010459 | 0.278227 | 0.044806 | -0.26622 | -0.05481 | TMEM174 |
| ILMN_1651712 | 0.006582 | 4.98E-04 | 0.267508 | -0.0948 | -0.20384 | 0.26251 | RBM11 |
| ILMN_1660223 | 0.014757 | 0.001945 | 0.341911 | 0.002407 | -0.17731 | -0.09175 | CREBL2 |
| ILMN_1802642 | 0.020993 | 0.003295 | 0.13986 | -0.49045 | 0.098016 | -0.32994 | TOM1L1 |
| ILMN_1706967 | 0.00365 | 1.17E-04 | 0.403084 | 0.037084 | -0.27683 | 0.19496 | ATF7 |
| ILMN_1757660 | 0.001226 | 7.26E-06 | 0.077414 | 0.20724 | -0.31934 | -0.3008 | CAPS |
| ILMN_1689212 | 0.00943 | 9.40E-04 | 0.444046 | 0.384367 | -0.29077 | 0.632609 | RSHL3 |
| ILMN_1737015 | 0.033052 | 0.006446 | 0.269883 | -0.0187 | -0.14423 | 0.101486 | RPL39 |
| ILMN_1706262 | 0.005191 | 3.00E-04 | 0.183044 | -0.18056 | -0.22069 | 0.147098 | HAGHL |
| ILMN_1765714 | 0.002717 | 4.61E-05 | 0.298552 | -0.0317 | -0.31557 | 0.250994 | UBE2E3 |
| ILMN_1693882 | 0.017392 | 0.002491 | -0.06688 | -0.62169 | 0.091405 | -1.05318 | TAPT1 |
| ILMN_1811775 | 0.016952 | 0.002397 | 0.086883 | -0.28843 | 0.276765 | -0.63476 | CCDC124 |
| ILMN_2148459 | 0.005009 | 2.69E-04 | -0.34566 | -5.39E-04 | 0.219234 | -0.66467 | B2M |
| ILMN_1749118 | 0.008689 | 8.13E-04 | 0.354815 | 0.025729 | -0.27783 | 0.02471 | CALML5 |
| ILMN_3307714 | 0.010198 | 0.00107 | 0.843403 | 0.182608 | -0.12538 | 0.476799 | GRIN2B |
| ILMN_2097943 | 0.028196 | 0.005115 | 0.559615 | -0.02447 | -0.2092 | 0.193881 | C1orf87 |
| ILMN_1665682 | 0.011531 | 0.001317 | 0.448135 | 1.118886 | -0.04376 | 0.505388 | IL15RA |
| ILMN_1730728 | 0.014003 | 0.001795 | 0.36348 | -0.00564 | -0.26233 | 0.119472 | OR2B2 |
| ILMN_1761996 | 2.54E-04 | 5.27E-07 | 0.405009 | -0.02039 | -0.32956 | 0.168605 | SFRS5 |
| ILMN_2378868 | 0.0051 | 2.84E-04 | 0.209235 | 0.09205 | -0.24779 | -0.13914 | SFRS5 |
| ILMN_1692168 | 0.029843 | 0.005581 | 0.228849 | -0.26586 | -0.31562 | -0.17574 | UBE2Z |
| ILMN_2226403 | 0.022908 | 0.003774 | 0.609081 | 0.069883 | -0.16577 | 0.525036 | OR4Q3 |
| ILMN_1706886 | 0.007265 | 6.01E-04 | 0.232262 | -0.11684 | -0.36482 | 0.027603 | BCL7A |
| ILMN_3241104 | 0.028666 | 0.005254 | 0.3534 | 0.018122 | -0.27505 | 0.074327 | LOC286135 |
| ILMN_1699358 | 0.037413 | 0.007653 | -0.07475 | 0.108965 | -0.45435 | 0.301065 | GNA12 |
| ILMN_1692191 | 0.048996 | 0.011316 | 1.079417 | 0.01384 | -0.15219 | 0.079778 | GNA12 |
| ILMN_1751086 | 0.046505 | 0.010489 | 0.209637 | 0.015938 | -0.20884 | -0.01109 | ATL3 |
| ILMN_1663754 | 0.004191 | 1.78E-04 | -0.10938 | 0.093098 | 0.348924 | -0.50807 | ZNF442 |
| ILMN_1657064 | 0.007944 | 7.00E-04 | 0.484667 | -0.30307 | -0.31851 | 0.529753 | C6orf70 |
| ILMN_3237236 | 0.001508 | 1.25E-05 | 0.500109 | -0.03715 | -0.3092 | 0.04837 | SNORD5 |
| ILMN_1758250 | 0.04695 | 0.010637 | -0.12537 | 0.592958 | -0.25209 | -0.19745 | TRAFD1 |
| ILMN_1792317 | 0.003061 | 6.44E-05 | 0.092647 | 0.084972 | -0.31493 | 0.189564 | SLCO1C1 |
| ILMN_1772132 | 0.015993 | 0.002201 | 0.370478 | -0.0234 | -0.10242 | -0.05786 | ATP5B |
| ILMN_1756806 | 0.015209 | 0.002042 | 0.09101 | 0.030484 | -0.30554 | 0.166604 | MCL1 |
| ILMN_2049343 | 0.028106 | 0.005084 | 0.263636 | 0.035198 | -0.31579 | -0.13216 | LOC642947 |
| ILMN_2294762 | 0.003258 | 7.58E-05 | -0.71666 | -0.10914 | 0.324318 | -1.39428 | AMY1A |
| ILMN_1773154 | 0.011174 | 0.001248 | 0.262309 | 0.017525 | -0.24827 | 0.045052 | NFKBIA |
| ILMN_1698745 | 0.018941 | 0.002819 | 0.306674 | 0.052365 | -0.1887 | 0.191124 | CLRN1 |
| ILMN_1730516 | 0.0058 | 3.83E-04 | 0.24051 | 0.010506 | -0.2434 | -0.02799 | TMEM133 |
| ILMN_1784977 | 0.0051 | 2.83E-04 | 0.249717 | 0.030007 | -0.23504 | -0.01176 | DOHH |
| ILMN_1775566 | 0.037443 | 0.007664 | 0.297756 | -0.05006 | -0.28007 | -0.11345 | ATP1A1 |
| ILMN_1739256 | 0.013777 | 0.001741 | 0.376224 | 0.008268 | -0.26367 | 0.074964 | BAHD1 |
| ILMN_1797116 | 3.70E-04 | 8.98E-07 | 0.422201 | -0.06209 | -0.36619 | 0.101633 | FAM45B |
| ILMN_2395969 | 0.014427 | 0.001877 | 0.375956 | -0.16412 | -0.1703 | 0.134054 | PRDX3 |
| ILMN_2153787 | 0.020241 | 0.003126 | -0.41873 | 0.9556 | -0.32916 | 0.704116 | ARRDC5 |
| ILMN_1743966 | 0.016412 | 0.002281 | 0.070708 | -0.40936 | -0.00259 | -0.90002 | BCL9L |
| ILMN_1755704 | 0.03923 | 0.008182 | 0.444459 | -0.26227 | -0.11525 | 0.666908 | PRB4 |
| ILMN_1657632 | 0.005129 | 2.90E-04 | -0.19556 | -0.37531 | 0.07915 | -1.03764 | ZMYM6 |
| ILMN_2166457 | 0.005264 | 3.14E-04 | -0.2887 | -0.46838 | 0.467507 | 0.15514 | HPGD |
| ILMN_1735006 | 0.041851 | 0.009017 | 0.151675 | -0.25273 | -0.01149 | -0.43492 | ATG2B |
| ILMN_3239383 | 0.033999 | 0.006713 | -0.06771 | -0.71957 | 0.358459 | -0.00579 | CA5BP |
| ILMN_1654118 | 0.014438 | 0.00188 | -0.56492 | -0.51103 | 0.440812 | -0.55393 | BCL2L1 |
| ILMN_1662065 | 0.008906 | 8.61E-04 | 0.269977 | 0.078543 | -0.33594 | 0.241406 | WDR92 |
| ILMN_3236452 | 0.007968 | 7.04E-04 | 0.36574 | 0.128671 | -0.21669 | 0.220542 | FAM187B |
| ILMN_3244646 | 0.028843 | 0.005303 | 0.304037 | 0.02221 | -0.37031 | -0.22705 | RNU1G2 |
| ILMN_2374244 | 0.021589 | 0.003433 | 0.208746 | 0.217238 | -0.1105 | 0.067448 | DYRK2 |
| ILMN_1661484 | 0.00314 | 7.00E-05 | 0.319647 | 0.149998 | -0.19366 | -0.10544 | ZBTB45 |
| ILMN_1711120 | 3.86E-04 | 1.04E-06 | -0.00855 | -0.21929 | 0.055418 | 2.115742 | ARC |
| ILMN_3308380 | 0.045032 | 0.010013 | 0.204582 | -0.06978 | -0.27024 | 0.090395 | MIR200B |
| ILMN_2371470 | 0.006875 | 5.46E-04 | 0.386751 | 0.099631 | -0.30461 | 0.122572 | C1orf124 |
| ILMN_2405078 | 0.014287 | 0.001846 | 0.276657 | -0.08193 | -0.12205 | 0.068605 | OSBPL8 |
| ILMN_1780268 | 0.028239 | 0.005131 | 0.162989 | -0.02005 | -0.29416 | 0.025808 | C1orf56 |
| ILMN_1709032 | 0.022558 | 0.00369 | 0.352507 | -0.31032 | -0.32551 | -0.21044 | FYCO1 |
| ILMN_3248343 | 0.017582 | 0.002538 | -0.1792 | 0.146643 | -0.29291 | 0.319233 | INO80 |
| ILMN_1678362 | 0.019873 | 0.003028 | -0.19347 | 0.171474 | -0.27264 | 0.260096 | INO80 |
| ILMN_2406654 | 0.035873 | 0.00723 | 0.268837 | 0.227704 | -0.15735 | 1.199894 | ABCC6 |
| ILMN_1733562 | 0.004131 | 1.68E-04 | -0.09165 | -0.42843 | 0.026601 | -0.73379 | TFB1M |
| ILMN_1747078 | 0.006396 | 4.66E-04 | -0.29627 | -0.50383 | 0.143378 | -0.2099 | HYLS1 |
| ILMN_1725193 | 0.009225 | 9.08E-04 | 0.358271 | -0.18247 | 0.174062 | -0.6625 | IGFBP2 |
| ILMN_1799887 | 0.02218 | 0.003597 | 0.262535 | 0.14931 | -0.2784 | -0.06024 | CTSE |
| ILMN_1760363 | 0.005447 | 3.38E-04 | 0.305245 | -0.02696 | -0.17968 | 0.153453 | GDF2 |
| ILMN_2130411 | 0.014354 | 0.001867 | -0.01629 | 0.124923 | 0.065034 | -0.41328 | KDELR1 |
| ILMN_3307868 | 0.012217 | 0.001443 | 0.274939 | -0.10059 | -0.34416 | -4.57E-04 | CHI3L1 |
| ILMN_2054938 | 0.007576 | 6.45E-04 | 0.428605 | 0.095557 | -0.25757 | 0.336579 | LOC389458 |
| ILMN_1665964 | 0.019064 | 0.002844 | 0.248707 | 0.171798 | -0.25826 | 0.007209 | GAB2 |
| ILMN_1762139 | 0.014528 | 0.001897 | 0.221295 | -0.00708 | -0.26383 | 0.112003 | CRB1 |
| ILMN_3237085 | 0.013578 | 0.001704 | 0.451749 | 0.191085 | -0.30115 | 0.29773 | NTF4 |
| ILMN_1782798 | 0.006731 | 5.28E-04 | 0.733671 | 0.039463 | -0.17659 | 0.161335 | MDFI |
| ILMN_2045994 | 0.003706 | 1.20E-04 | -0.2021 | -0.10379 | 0.135426 | -0.77812 | SEPW1 |
| ILMN_1694077 | 0.015996 | 0.002204 | 0.358366 | 0.233262 | -0.28723 | 0.069888 | TAAR6 |
| ILMN_1651850 | 0.013846 | 0.001756 | 0.297405 | 0.020881 | -0.2693 | -0.05709 | RPS16 |
| ILMN_1708906 | 0.021414 | 0.00339 | 0.251869 | 0.031732 | -0.19577 | -0.04091 | C2orf29 |
| ILMN_1701213 | 0.003277 | 7.90E-05 | 0.130396 | 0.008456 | -0.42697 | 0.056057 | PIP4K2B |
| ILMN_2380698 | 0.040732 | 0.008647 | 0.251423 | 0.107402 | -0.13482 | -0.15929 | DSTN |
| ILMN_1700604 | 0.003977 | 1.46E-04 | 0.399596 | 0.006746 | -0.14873 | -0.23352 | RBM14 |
| ILMN_3248035 | 0.00943 | 9.41E-04 | 0.442978 | 0.0279 | -0.27695 | 0.146241 | NTN5 |
| ILMN_1741143 | 0.009588 | 9.64E-04 | 0.397295 | 0.054058 | -0.23662 | 0.326724 | TXK |
| ILMN_2082109 | 0.002978 | 5.89E-05 | 0.376876 | 0.014141 | -0.27724 | 0.139244 | ZNF214 |
| ILMN_1678422 | 0.043454 | 0.009508 | 0.412627 | -0.05739 | -0.25802 | 0.058405 | DHX58 |
| ILMN_2109197 | 0.035259 | 0.007069 | 0.094601 | -0.45032 | -0.09149 | 0.392948 | EPB41L3 |
| ILMN_1756862 | 0.01021 | 0.001073 | 0.290569 | 0.084202 | -0.16018 | -0.07091 | APOL3 |
| ILMN_2369144 | 0.005332 | 3.21E-04 | 0.3815 | 0.050366 | -0.22237 | 0.062693 | APOL3 |
| ILMN_1663648 | 0.022668 | 0.003712 | 0.56292 | -0.49555 | 0.195758 | -0.3831 | MTUS1 |
| ILMN_1694111 | 0.048784 | 0.01125 | -0.11742 | 0.085988 | -0.01763 | -0.85142 | PNKP |
| ILMN_1742604 | 0.043003 | 0.009343 | 0.112312 | -0.10938 | -0.36042 | 0.001431 | C10orf4 |
| ILMN_1664607 | 0.006946 | 5.55E-04 | 0.323537 | 0.126002 | -0.29899 | -0.10003 | NRIP2 |
| ILMN_3244065 | 0.003086 | 6.60E-05 | -0.1367 | -0.20594 | 0.192236 | -0.31674 | C9orf69 |
| ILMN_1727831 | 0.004851 | 2.46E-04 | 0.270409 | 0.177558 | -0.26583 | 0.177022 | RAD51L1 |
| ILMN_1759984 | 0.013904 | 0.001771 | 0.292544 | 0.061338 | -0.27288 | -0.32423 | KCTD1 |
| ILMN_1784534 | 0.006221 | 4.37E-04 | 0.369309 | 0.001953 | -0.38143 | -0.05638 | SELS |
| ILMN_1681415 | 0.003455 | 9.65E-05 | 0.297549 | 0.105744 | -0.30108 | 0.133837 | SIGLEC7 |
| ILMN_1730611 | 0.003977 | 1.46E-04 | 0.255474 | -0.09721 | -0.13613 | 0.039447 | RTN4 |
| ILMN_1766103 | 0.002424 | 3.46E-05 | 0.357298 | 0.123487 | -0.23825 | 0.255737 | KCNK18 |
| ILMN_1727526 | 0.027311 | 0.004878 | 0.190732 | 0.180812 | -0.48422 | 0.580405 | KIAA1407 |
| ILMN_1760503 | 8.08E-04 | 3.55E-06 | 0.490395 | 0.077464 | -0.29437 | 0.081756 | ZBBX |
| ILMN_1690703 | 0.005404 | 3.31E-04 | 0.285431 | 0.100003 | -0.20654 | -0.03369 | C21orf34 |
| ILMN_2174369 | 0.013927 | 0.001776 | -0.58811 | 0.022695 | 0.121789 | 0.330124 | ELOVL5 |
| ILMN_1775408 | 0.005016 | 2.72E-04 | 0.351717 | 0.045165 | -0.16762 | 0.113985 | SPACA3 |
| ILMN_2116639 | 0.014003 | 0.001795 | 0.291074 | -0.11295 | -0.27608 | 0.048985 | TFDP2 |
| ILMN_1711439 | 2.51E-04 | 5.09E-07 | -0.43348 | -0.00566 | 0.12328 | -1.92204 | EMILIN1 |
| ILMN_2315964 | 0.009429 | 9.38E-04 | 0.400282 | 0.102273 | -0.24191 | -0.14904 | PSRC1 |
| ILMN_1782748 | 0.022373 | 0.003656 | 0.346621 | 0.055301 | -0.26632 | -0.01505 | FMNL1 |
| ILMN_3240365 | 0.002424 | 3.49E-05 | 0.368192 | -0.02663 | -0.2402 | 0.140578 | MNX1 |
| ILMN_1787529 | 0.034986 | 0.006993 | 0.339438 | 0.072008 | -0.17808 | 0.065642 | C3AR1 |
| ILMN_2198897 | 0.007201 | 5.93E-04 | 0.450619 | -0.05718 | -0.39995 | 0.116544 | ABCD2 |
| ILMN_1805526 | 0.003568 | 1.05E-04 | 0.229576 | -0.062 | -0.23558 | 0.124718 | MYL1 |
| ILMN_3191746 | 0.004034 | 1.52E-04 | 0.371701 | 0.035324 | -0.28994 | 0.040454 | BCAR4 |
| ILMN_1726815 | 0.020029 | 0.003072 | 0.565603 | -0.08705 | -0.36506 | 0.053405 | HIST1H3G |
| ILMN_1761910 | 0.004508 | 2.12E-04 | 0.748431 | 0.002978 | -0.14824 | 0.197179 | BDNF |
| ILMN_1691539 | 0.041594 | 0.008931 | 0.096395 | 0.292785 | -0.2485 | -0.01873 | LAT |
| ILMN_2404625 | 0.032871 | 0.006396 | 0.074949 | 0.151609 | -0.33421 | -0.14394 | LAT |
| ILMN_1735764 | 0.024682 | 0.004194 | -0.17474 | 0.266312 | 0.287381 | -1.07008 | HTR2B |
| ILMN_1772242 | 0.003917 | 1.38E-04 | 0.234567 | 0.012621 | -0.22776 | 0.195987 | TRPC5 |
| ILMN_1740155 | 0.021887 | 0.003507 | 0.321122 | 0.155901 | -0.29945 | 0.048442 | COL9A3 |
| ILMN_1779228 | 0.006337 | 4.56E-04 | -0.29116 | 9.02E-04 | 0.048705 | -0.94566 | CDH2 |
| ILMN_1697925 | 0.004191 | 1.78E-04 | 0.338729 | 0.395853 | -0.34378 | -0.00564 | APOB48R |
| ILMN_2100285 | 0.002205 | 2.88E-05 | 0.379378 | 0.043183 | -0.2989 | 0.171822 | CCDC147 |
| ILMN_2100287 | 0.017881 | 0.002598 | 0.264181 | -0.19563 | -0.17309 | 0.110384 | CCDC147 |
| ILMN_2325610 | 0.040914 | 0.008709 | 0.214542 | -0.31045 | -0.01171 | -0.15337 | AKT3 |
| ILMN_1675695 | 0.003258 | 7.56E-05 | -0.22362 | -0.3825 | 0.364964 | -0.57188 | PDS5B |
| ILMN_2099301 | 0.006645 | 5.10E-04 | 0.304415 | -0.02477 | -0.2215 | 0.23379 | UNC84B |
| ILMN_1782977 | 0.007649 | 6.57E-04 | 0.256004 | 0.00923 | -0.16942 | 0.189149 | UBA52 |
| ILMN_2147306 | 0.031739 | 0.006098 | 0.174927 | -0.24641 | 0.094824 | -0.36844 | PNRC2 |
| ILMN_1699423 | 0.006075 | 4.17E-04 | 0.425234 | 0.062602 | -0.12122 | 0.051324 | ZNF146 |
| ILMN_2284077 | 0.034438 | 0.006841 | 0.408003 | -0.04051 | -0.24591 | -0.01548 | SLC38A8 |
| ILMN_1808979 | 0.007314 | 6.09E-04 | 0.590423 | 0.01158 | -0.26463 | 0.158307 | CLEC4D |
| ILMN_2192072 | 0.00943 | 9.41E-04 | -0.26781 | 0.19885 | 0.041197 | -1.07099 | MMP7 |
| ILMN_1656410 | 0.015699 | 0.002144 | 0.45299 | -0.06376 | 0.229867 | 0.005151 | TRPC6 |
| ILMN_3244090 | 0.003763 | 1.25E-04 | 0.355619 | -0.0543 | -0.30927 | 0.133207 | GAGE12H |
| ILMN_2367157 | 0.010157 | 0.001062 | 0.337822 | -0.04667 | -0.25423 | 0.20855 | CHRFAM7A |
| ILMN_1764851 | 0.026525 | 0.004672 | 0.400998 | -0.26475 | -0.13776 | -0.28467 | TP53RK |
| ILMN_2363439 | 0.017539 | 0.002528 | 0.241076 | 0.243311 | -0.27213 | 0.237449 | RYK |
| ILMN_1770209 | 0.022111 | 0.00358 | 0.865107 | 0.266156 | -0.14011 | 0.253478 | LY6G5C |
| ILMN_1704441 | 0.00383 | 1.31E-04 | 0.366853 | 0.163697 | -0.19964 | 0.009834 | ZNF556 |
| ILMN_3237564 | 0.025563 | 0.004431 | 0.210013 | 0.021382 | -0.22098 | 0.078676 | PP14571 |
| ILMN_2383097 | 0.02205 | 0.00355 | 0.262698 | 0.031299 | -0.22453 | 0.059308 | RPL17 |
| ILMN_3309664 | 0.004183 | 1.72E-04 | 0.367929 | -0.03618 | -0.25609 | 0.163583 | MIR300 |
| ILMN_1675453 | 0.001086 | 5.68E-06 | -0.56178 | 0.054658 | 0.468081 | -0.74169 | HHIP |
| ILMN_2400206 | 0.01007 | 0.001042 | 0.314004 | 0.035865 | -0.26097 | 0.045151 | UBE2V1 |
| ILMN_1652826 | 0.026662 | 0.004709 | -0.12904 | 0.123606 | 0.090661 | -0.85043 | LRRC17 |
| ILMN_1674793 | 0.023696 | 0.00396 | 0.012599 | -0.04713 | -0.21763 | 0.002924 | LRRC17 |
| ILMN_1656372 | 0.010965 | 0.00121 | 0.190784 | 0.087587 | -0.31719 | -0.12322 | PES1 |
| ILMN_2119555 | 0.023038 | 0.003812 | -0.19204 | -0.2462 | 0.561476 | -0.3179 | TMTC3 |
| ILMN_1673941 | 0.007273 | 6.04E-04 | 0.278474 | 0.014273 | -0.22219 | 0.443205 | RBM24 |
| ILMN_1672662 | 0.004885 | 2.52E-04 | 0.172682 | -0.35294 | -0.30984 | 0.576821 | SLC20A1 |
| ILMN_1797181 | 0.015046 | 0.002003 | 0.585428 | 0.07546 | -0.22675 | -0.01675 | LOC93622 |
| ILMN_2388166 | 0.018941 | 0.002815 | 0.186915 | -0.0298 | -0.34273 | 0.174464 | HDAC5 |
| ILMN_1682375 | 0.005465 | 3.40E-04 | 0.133909 | 0.059309 | -0.33037 | 0.139034 | ATPBD3 |
| ILMN_1757626 | 5.52E-04 | 1.88E-06 | 0.740861 | -0.05343 | -0.29413 | 0.119291 | ARID3C |
| ILMN_1725896 | 0.002621 | 4.30E-05 | 0.364193 | -0.09228 | -0.33586 | 0.041798 | AMIGO2 |
| ILMN_1651943 | 0.001337 | 9.06E-06 | 0.367685 | 0.008772 | -0.38175 | 0.169212 | ANK3 |
| ILMN_1760705 | 0.002717 | 4.60E-05 | 0.330349 | -0.05094 | -0.31565 | 0.090743 | POF1B |
| ILMN_1810214 | 0.005191 | 3.01E-04 | 0.31101 | 0.021571 | -0.27529 | 0.104978 | JUND |
| ILMN_3242059 | 0.002811 | 5.01E-05 | 0.356466 | 0.013298 | -0.27439 | 0.083959 | C6orf221 |
| ILMN_1756734 | 0.020015 | 0.003065 | 0.35452 | 0.202039 | -0.16198 | 0.217556 | MYO15A |
| ILMN_2203147 | 0.004191 | 1.77E-04 | 0.333661 | 6.16E-04 | -0.31315 | 0.096352 | TMPRSS12 |
| ILMN_3238228 | 0.00499 | 2.67E-04 | 0.34276 | -0.06396 | -0.30891 | 0.152886 | VCX3B |
| ILMN_1677868 | 0.009777 | 9.92E-04 | 0.253819 | 0.077053 | -0.18422 | 0.017344 | ADRA2B |
| ILMN_1697694 | 0.009049 | 8.83E-04 | 0.234919 | 0.092502 | -0.24665 | -0.00457 | ATP6AP1 |
| ILMN_3245103 | 1.87E-04 | 3.35E-07 | 0.471963 | 0.053471 | -0.4457 | 0.402974 | RNU11 |
| ILMN_1723412 | 0.010986 | 0.001215 | 0.170978 | 0.383512 | -0.30511 | -0.44753 | ASCL2 |
| ILMN_2317739 | 0.005129 | 2.91E-04 | 0.354174 | -0.12537 | -0.28933 | 0.039695 | ITSN2 |
| ILMN_2413644 | 0.005087 | 2.81E-04 | 0.669006 | 0.325365 | -0.20748 | 0.168509 | TM4SF19 |
| ILMN_1726466 | 0.02762 | 0.004951 | 0.330445 | 0.092526 | -0.20624 | -0.02614 | HDHD3 |
| ILMN_2142185 | 0.047554 | 0.010841 | 0.132529 | 0.199396 | -0.02284 | -0.29291 | CLEC14A |
| ILMN_3249676 | 0.006571 | 4.95E-04 | 0.356114 | -0.20618 | -0.27985 | 0.16674 | BOLA2B |
| ILMN_3307858 | 0.006624 | 5.04E-04 | 0.385402 | 0.067329 | -0.18954 | 0.068885 | STIM2 |
| ILMN_2105966 | 0.026278 | 0.004614 | 0.120335 | -0.45056 | -0.06955 | 0.172808 | SLC35A4 |
| ILMN_1693303 | 0.022199 | 0.003603 | 0.656478 | 0.274545 | -0.18336 | 0.193578 | TAS2R41 |
| ILMN_1755869 | 0.001907 | 1.99E-05 | 0.461189 | 0.022039 | -0.35345 | 0.021712 | OR51T1 |
| ILMN_1652487 | 0.004785 | 2.40E-04 | 0.383606 | 0.057078 | -0.20865 | 0.179261 | RIOK1 |
| ILMN_1796018 | 0.002842 | 5.12E-05 | 0.468537 | -0.30377 | -0.40896 | 0.75386 | NFATC4 |
| ILMN_2401618 | 0.040542 | 0.00858 | 0.286413 | -0.42787 | 0.451213 | 0.291234 | MLX |
| ILMN_3239279 | 0.034683 | 0.006904 | 0.48602 | 0.023888 | -0.2797 | 1.66E-04 | RSPH10B2 |
| ILMN_1736981 | 0.031188 | 0.005945 | 0.514791 | 0.050041 | -0.27459 | 0.310514 | BRD4 |
| ILMN_1736005 | 0.033562 | 0.006586 | 1.182941 | -0.30916 | 0.083514 | 0.579407 | NCAPH2 |
| ILMN_2290453 | 0.011906 | 0.001387 | 0.500352 | -0.20734 | -0.12 | 0.09581 | PTGER3 |
| ILMN_3245554 | 0.00256 | 3.93E-05 | 0.815599 | -0.13497 | -0.40564 | 0.428108 | SNORD1B |
| ILMN_3251531 | 0.002761 | 4.85E-05 | 0.339045 | -0.00939 | -0.32502 | 0.017514 | ZNF793 |
| ILMN_1743783 | 0.005447 | 3.37E-04 | 0.541557 | 0.001797 | -0.2518 | 0.276175 | CCDC43 |
| ILMN_2408885 | 0.003279 | 8.20E-05 | 0.190417 | -0.00553 | -0.30596 | 0.067261 | HDAC9 |
| ILMN_1664815 | 0.012725 | 0.001546 | 0.258598 | 0.240098 | -0.29547 | 0.00671 | ELK4 |
| ILMN_1792150 | 0.002717 | 4.62E-05 | 0.394587 | 0.121851 | -0.28274 | 0.145916 | ALOX5 |
| ILMN_3242067 | 0.042236 | 0.00912 | 0.102168 | 0.023307 | -0.33142 | 0.022723 | SNORA58 |
| ILMN_1658821 | 0.044869 | 0.009955 | -0.62416 | -0.36053 | 0.340169 | -0.1224 | SAMD1 |
| ILMN_2408543 | 0.017183 | 0.002449 | 0.33561 | -0.18954 | -0.27001 | 0.643131 | PLAUR |
| ILMN_2051563 | 0.020608 | 0.003215 | 0.284728 | 0.018167 | -0.32749 | 0.070493 | HIGD2B |
| ILMN_1736575 | 0.033305 | 0.006512 | 0.170216 | -0.15097 | -0.18207 | 0.308926 | TRIM28 |
| ILMN_1711810 | 0.019755 | 0.002991 | 0.388143 | -0.37952 | -0.30739 | 0.309078 | PNKD |
| ILMN_1766573 | 0.032425 | 0.006282 | 0.134314 | -0.53874 | -0.63497 | 0.482779 | TERC |
| ILMN_2409062 | 0.006328 | 4.54E-04 | 0.277552 | -0.0375 | -0.26286 | 0.15614 | ISCU |
| ILMN_2346460 | 0.015209 | 0.002043 | -0.50674 | -0.20347 | 0.434983 | -0.61145 | NARG2 |
| ILMN_1792910 | 0.015078 | 0.002011 | 0.172677 | 0.028184 | -0.17094 | 0.307184 | MNT |
| ILMN_1695280 | 0.027133 | 0.004816 | 0.358019 | -0.02191 | -0.36398 | 0.154836 | SMG6 |
| ILMN_1698952 | 0.011385 | 0.001284 | 0.498714 | 0.10004 | -0.29034 | 0.315139 | TNFRSF11A |
| ILMN_1753190 | 0.029103 | 0.005372 | 0.300315 | -0.12997 | -0.15683 | -0.04095 | C9orf102 |
| ILMN_3249088 | 0.002998 | 6.07E-05 | 0.590381 | 0.083722 | -0.2086 | 0.194286 | LOC93432 |
| ILMN_2385220 | 0.005688 | 3.67E-04 | 0.287322 | 0.038921 | -0.28094 | -0.01417 | DFFA |
| ILMN_2068435 | 0.044869 | 0.009953 | -0.08253 | 0.154435 | 0.090971 | -0.67423 | ZNF700 |
| ILMN_1804328 | 0.006328 | 4.55E-04 | 0.285122 | 4.73E-04 | -0.22345 | 0.076104 | WWP1 |
| ILMN_1745447 | 0.009869 | 0.001009 | 0.393905 | 0.097256 | -0.14446 | 0.048882 | HIVEP2 |
| ILMN_1687636 | 0.047051 | 0.010672 | 0.169746 | -0.13364 | -0.29459 | 0.643007 | ABCA2 |
| ILMN_1747627 | 0.027191 | 0.004835 | -0.37906 | 0.156533 | 0.102544 | -0.92824 | ABCA2 |
| ILMN_1813379 | 0.005476 | 3.42E-04 | 0.492821 | -0.08223 | -0.37009 | 1.040744 | TNFRSF9 |
| ILMN_1815120 | 0.045702 | 0.01022 | 0.561794 | 0.812931 | -0.13563 | 1.446512 | PRDM14 |
| ILMN_1792681 | 0.032198 | 0.006215 | -0.03294 | -0.21408 | -0.15883 | 0.367328 | CCDC86 |
| ILMN_2371724 | 0.04512 | 0.010043 | -0.20524 | -0.10832 | 0.15205 | -0.7748 | CEACAM1 |
| ILMN_1776848 | 0.014904 | 0.001973 | 0.606792 | -0.28522 | -0.08068 | 0.540089 | ZFYVE9 |
| ILMN_3181406 | 0.038657 | 0.007998 | 0.361563 | 0.054956 | -0.20082 | 0.259465 | DHX40P |
| ILMN_1658144 | 0.031241 | 0.005958 | -0.07675 | -0.24033 | 0.142038 | -0.39115 | TRIP11 |
| ILMN_2390435 | 0.003604 | 1.10E-04 | 0.356168 | -0.12829 | -0.25808 | 0.226756 | LHX3 |
| ILMN_2284893 | 0.002863 | 5.37E-05 | 0.268699 | -0.02281 | -0.31676 | 0.053643 | LHX3 |
| ILMN_1771333 | 0.02132 | 0.003368 | -0.41035 | 0.003651 | 0.33264 | -0.95219 | CD47 |
| ILMN_1811468 | 0.018941 | 0.002819 | 0.446983 | 0.144179 | -0.17295 | 0.022791 | IRX3 |
| ILMN_3233838 | 0.006582 | 4.97E-04 | 0.089984 | -0.00964 | -0.33486 | 0.130348 | LOC399940 |
| ILMN_1783394 | 0.002417 | 3.33E-05 | 0.106997 | 0.295946 | -0.44718 | 0.571001 | ATF4 |
| ILMN_1787879 | 0.011297 | 0.001269 | 0.214722 | -0.03397 | -0.35386 | -0.12632 | ARL2 |
| ILMN_2350421 | 0.024892 | 0.00425 | 0.389663 | 0.062307 | -0.30533 | -0.01937 | C19orf29 |
| ILMN_1742779 | 0.015254 | 0.002056 | 0.255158 | 0.054356 | -0.22253 | 0.07689 | CENPL |
| ILMN_1687090 | 0.010017 | 0.001033 | -0.49586 | -0.12169 | 0.539977 | 1.076979 | GABRB3 |
| ILMN_2359159 | 0.017474 | 0.002511 | 0.311549 | -0.05832 | -0.30297 | 0.07557 | DSC3 |
| ILMN_1652306 | 0.03963 | 0.008308 | 0.16192 | -0.2864 | -0.24469 | 0.083862 | MEGF10 |
| ILMN_1690114 | 0.009812 | 0.001001 | -0.11929 | 0.313878 | 0.078024 | -0.94909 | PTPLAD2 |
| ILMN_1776649 | 0.009202 | 9.05E-04 | 0.089021 | 0.202012 | -0.06239 | -0.57633 | LRRK2 |
| ILMN_1672536 | 0.01523 | 0.002047 | 0.358838 | 0.152616 | -0.12685 | -0.07771 | FBLN1 |
| ILMN_3265895 | 0.004377 | 2.00E-04 | 0.165657 | 0.084453 | -0.37037 | -0.04704 | HNRNPR |
| ILMN_1756541 | 0.007482 | 6.31E-04 | 0.271364 | 0.018634 | -0.29788 | -0.002 | MXD4 |
| ILMN_2051519 | 0.014914 | 0.001975 | 0.299248 | 0.11282 | -0.19143 | 0.069287 | RPL37A |
| ILMN_1686313 | 0.048211 | 0.011059 | 0.481359 | -0.22762 | 0.097817 | -0.03373 | PLXNB3 |
| ILMN_1699724 | 0.008758 | 8.28E-04 | 0.438319 | 0.060411 | -0.26392 | 0.060628 | C2orf3 |
| ILMN_1693683 | 0.03771 | 0.007727 | 0.282212 | -0.06884 | -0.27806 | 0.10407 | PDAP1 |
| ILMN_2323338 | 0.009082 | 8.88E-04 | 0.219695 | -0.17259 | -0.39064 | 0.106471 | NR1I2 |
| ILMN_1658802 | 0.019906 | 0.003036 | 0.309149 | 0.037865 | -0.15681 | -0.05371 | KRTCAP2 |
| ILMN_1656134 | 0.016593 | 0.002326 | 0.191375 | -0.06243 | -0.22013 | 0.059363 | CNOT7 |
| ILMN_1668778 | 0.0066 | 5.01E-04 | -0.21708 | -0.33167 | 0.229253 | 0.010622 | PRKAA2 |
| ILMN_1771957 | 0.029296 | 0.005438 | -0.02869 | -0.2878 | 0.135108 | -1.12111 | MAN1B1 |
| ILMN_1661458 | 0.002842 | 5.12E-05 | 0.278072 | -0.06857 | -0.35174 | 0.142051 | ARF1 |
| ILMN_3242330 | 0.038114 | 0.007846 | -0.01312 | 0.053033 | -0.41415 | 0.025514 | FAM65C |
| ILMN_1795464 | 0.022692 | 0.003721 | 0.195427 | 0.880044 | -0.37585 | -0.05074 | LTA |
| ILMN_1768556 | 0.028337 | 0.005158 | 0.317911 | 0.080098 | -0.22906 | 0.076527 | CASKIN2 |
| ILMN_3308743 | 0.003916 | 1.37E-04 | 0.448219 | 0.048561 | -0.25287 | 0.132813 | MIR125B2 |
| ILMN_2151114 | 0.022957 | 0.003786 | 0.462366 | 0.011717 | -0.2243 | 0.05092 | VSNL1 |
| ILMN_1692779 | 0.017294 | 0.002472 | 0.207488 | 0.087993 | -0.10094 | -0.37512 | PRPF39 |
| ILMN_1739573 | 0.002631 | 4.36E-05 | 0.140555 | 0.061222 | -0.34701 | 0.081602 | TNRC6A |
| ILMN_1689037 | 0.002761 | 4.84E-05 | 0.425228 | -0.03411 | -0.37973 | 0.086169 | LIPG |
| ILMN_1779584 | 0.00561 | 3.58E-04 | 0.432029 | 0.068496 | -0.25393 | 0.11884 | UTP18 |
| ILMN_1799389 | 0.014874 | 0.001968 | 0.134457 | 0.038078 | -0.35266 | -0.00321 | FBXL6 |
| ILMN_1741692 | 0.005447 | 3.38E-04 | 0.391348 | 0.010603 | -0.25836 | 0.117087 | FOXL1 |
| ILMN_1749317 | 0.003277 | 7.87E-05 | 0.524672 | 0.116155 | -0.18464 | 0.331923 | C1orf222 |
| ILMN_1737738 | 8.85E-04 | 4.10E-06 | 0.292019 | -0.05432 | -0.26352 | 0.140733 | NDUFA12 |
| ILMN_1800638 | 0.006266 | 4.44E-04 | -1.45333 | 0.601668 | -0.52122 | 0.138 | CUGBP2 |
| ILMN_1773906 | 0.041002 | 0.008737 | 0.210084 | -0.05215 | -0.2718 | -0.06317 | NCOA4 |
| ILMN_1670554 | 0.010072 | 0.001044 | -0.15257 | 0.078511 | 0.041884 | -0.60781 | WDR78 |
| ILMN_2158336 | 0.041156 | 0.008789 | 0.279275 | 0.067613 | -0.17839 | 0.068838 | SH3GLB2 |
| ILMN_3243457 | 0.006365 | 4.61E-04 | 0.283256 | -0.18305 | -0.36925 | 0.107279 | ANKLE1 |
| ILMN_1695807 | 0.002205 | 2.86E-05 | 0.226011 | -0.01003 | -0.36419 | 0.087585 | DYRK3 |
| ILMN_2234970 | 0.028863 | 0.00531 | 0.18464 | -0.0386 | -0.25878 | 0.118594 | SLC39A3 |
| ILMN_1758333 | 0.008435 | 7.75E-04 | 0.368024 | 0.080354 | -0.16979 | 0.260244 | HMGXB4 |
| ILMN_2392818 | 0.017132 | 0.002433 | 0.026549 | -0.07685 | -0.3192 | -0.05757 | RTKN |
| ILMN_1764052 | 0.016236 | 0.00225 | 0.36068 | -0.08688 | -0.25832 | 0.320714 | EGR4 |
| ILMN_2058070 | 0.043487 | 0.009521 | 0.311287 | -0.05328 | -0.20946 | 0.101915 | NEDD8 |
| ILMN_1662675 | 0.00689 | 5.48E-04 | 0.460305 | 0.027576 | -0.19234 | 0.103517 | ADAM2 |
| ILMN_3245476 | 0.005688 | 3.68E-04 | 0.260934 | 0.028929 | -0.19663 | 0.092731 | PHRF1 |
| ILMN_1803194 | 0.012636 | 0.001531 | 0.272168 | -0.02302 | -0.36644 | -0.00332 | GALK1 |
| ILMN_1652754 | 0.048424 | 0.011133 | 0.115499 | 0.14362 | -0.19873 | -0.00372 | ZNF428 |
| ILMN_1716983 | 0.017467 | 0.002509 | 0.111047 | 0.546625 | -0.20859 | -1.00366 | LILRA2 |
| ILMN_1660907 | 0.02486 | 0.004236 | 0.414563 | -0.14711 | -0.17846 | 0.407849 | PTPRE |
| ILMN_1661750 | 0.036147 | 0.007307 | 0.199077 | -0.51311 | -0.1346 | 0.120254 | VIL1 |
| ILMN_1680617 | 0.013878 | 0.001761 | 0.391333 | -0.07909 | -0.20478 | 0.084588 | PLXNA2 |
| ILMN_1773645 | 0.002338 | 3.15E-05 | 0.38807 | 0.11855 | -0.24626 | 0.137861 | GMPPB |
| ILMN_3237396 | 0.004068 | 1.62E-04 | 0.294585 | 0.079069 | -0.25716 | 0.230822 | AAGAB |
| ILMN_2290628 | 0.037382 | 0.007641 | 0.305948 | 0.551093 | -0.29476 | 1.009364 | IL16 |
| ILMN_1727142 | 0.010479 | 0.001118 | 0.221879 | 0.117661 | -0.28616 | 0.097088 | IKBKB |
| ILMN_1665748 | 0.011611 | 0.001336 | 0.368079 | -0.06955 | -0.1944 | 0.124408 | DCC |
| ILMN_3238435 | 0.046853 | 0.01059 | -0.12714 | 0.205078 | -0.09705 | 0.44765 | SNORA12 |
| ILMN_1764945 | 0.020608 | 0.003216 | -0.75556 | -0.08539 | 0.040673 | -0.78969 | AP3D1 |
| ILMN_1777881 | 0.018369 | 0.002705 | 0.279451 | 0.120048 | -0.24223 | -0.00121 | TSPAN17 |
| ILMN_3241941 | 1.22E-04 | 1.44E-07 | 0.357858 | 0.225473 | -0.50674 | 0.914133 | SCARNA2 |
| ILMN_1670664 | 0.01157 | 0.001325 | -0.06717 | 0.016625 | -0.12585 | 0.483475 | PPP1R15B |
| ILMN_2296011 | 0.015143 | 0.002026 | 0.254929 | 0.146707 | -0.25695 | 0.062138 | BRWD1 |
| ILMN_2355462 | 0.002421 | 3.44E-05 | 0.157591 | -0.05106 | -0.40344 | 0.359581 | CYFIP1 |
| ILMN_1796126 | 0.0051 | 2.84E-04 | 0.234027 | 0.005418 | -0.30071 | 0.041804 | MXRA7 |
| ILMN_2264981 | 0.020411 | 0.00317 | 0.261173 | -0.47059 | 0.294042 | 0.124532 | CYFIP2 |
| ILMN_1655154 | 0.008363 | 7.62E-04 | 0.36144 | 0.03632 | -0.24458 | 0.033015 | PTBP1 |
| ILMN_2112301 | 0.012374 | 0.001471 | 0.131698 | 0.047059 | -0.21589 | 0.027794 | DRAP1 |
| ILMN_1765072 | 0.047879 | 0.010955 | 0.540799 | -0.02171 | -0.14745 | 0.172152 | KRT85 |
| ILMN_1766079 | 0.00795 | 7.02E-04 | 0.148016 | -0.15481 | -0.27656 | -2.37E-04 | SULT1C2 |
| ILMN_1704529 | 0.002614 | 4.25E-05 | 0.448284 | -1.01588 | 0.116242 | 1.037392 | PPIA |
| ILMN_3245815 | 0.035585 | 0.007153 | -0.17583 | -0.04433 | 0.212672 | -0.57748 | PAR1 |
| ILMN_1787324 | 0.003279 | 8.18E-05 | 0.275026 | 0.089203 | -0.3659 | -0.03742 | C16orf48 |
| ILMN_1741640 | 0.049595 | 0.011495 | 0.124588 | 0.064951 | -0.09481 | -0.27204 | ZNF675 |
| ILMN_2387696 | 0.01366 | 0.001718 | 0.251853 | 0.055715 | -0.37359 | -0.11736 | CCR6 |
| ILMN_1704497 | 0.002851 | 5.22E-05 | 0.339792 | 0.120443 | -0.38558 | 0.10416 | KRT25 |
| ILMN_3238972 | 0.018131 | 0.002646 | 0.643561 | -0.22159 | -0.28326 | 0.102583 | SNORD123 |
| ILMN_1679919 | 0.033874 | 0.006678 | 0.206661 | 0.01688 | -0.17733 | 0.070778 | ASCC2 |
| ILMN_1779616 | 0.013493 | 0.001684 | 0.326368 | -0.05547 | -0.2676 | -0.04223 | SUCLG1 |
| ILMN_2351298 | 0.039815 | 0.008353 | 0.111177 | 0.142619 | -0.18976 | 0.07535 | WIPF1 |
| ILMN_2086077 | 2.21E-05 | 1.67E-08 | 0.188184 | 0.159517 | -0.29606 | 1.800087 | JUNB |
| ILMN_1669296 | 0.022364 | 0.003645 | 0.415742 | 0.12638 | -0.34346 | 0.466354 | UNQ9438 |
| ILMN_1747217 | 0.027172 | 0.004828 | 0.286698 | -0.1189 | 0.151332 | -0.50866 | C15orf41 |
| ILMN_1730492 | 0.004543 | 2.15E-04 | 0.286507 | 0.053949 | -0.28557 | 0.154255 | PROP1 |
| ILMN_1748614 | 0.003641 | 1.15E-04 | 0.312507 | 0.10512 | -0.25365 | 0.192864 | RELT |
| ILMN_1796926 | 0.025821 | 0.004502 | 0.467797 | -0.38973 | 0.006131 | -0.61818 | PABPC5 |
| ILMN_1686637 | 0.005158 | 2.96E-04 | 0.703828 | 0.174558 | -0.31954 | 0.259231 | CXorf55 |
| ILMN_1727815 | 0.049955 | 0.011612 | 0.161733 | 0.209531 | -0.252 | -0.10842 | CFI |
| ILMN_1655987 | 0.043711 | 0.009589 | -0.20328 | 0.198686 | -0.06563 | -1.10588 | STAB1 |
| ILMN_1702696 | 0.004255 | 1.85E-04 | -0.40398 | 0.002057 | 0.11504 | -0.90753 | AFAR3 |
| ILMN_1784577 | 0.003802 | 1.29E-04 | 0.744847 | 0.040367 | -0.29409 | 0.157155 | ZNF12 |
| ILMN_1676868 | 0.015264 | 0.00206 | 0.06869 | 0.26974 | -0.31366 | 0.329468 | RAB37 |
| ILMN_1748675 | 0.013904 | 0.00177 | 0.35437 | 0.241131 | -0.24621 | 0.030704 | TMPRSS11B |
| ILMN_1657248 | 0.006683 | 5.19E-04 | 0.319881 | 6.77E-04 | -0.25883 | 0.139113 | EREG |
| ILMN_1789446 | 0.020776 | 0.003255 | 0.751166 | -0.03757 | -0.1556 | 0.243411 | IRGC |
| ILMN_1685289 | 0.018863 | 0.002799 | 0.314295 | 0.001586 | -0.31753 | -0.03564 | C16orf58 |
| ILMN_1654629 | 0.002005 | 2.24E-05 | 0.141292 | 0.388927 | -0.1965 | 0.547161 | TMEM175 |
| ILMN_2227338 | 0.012153 | 0.00143 | 0.690619 | 0.018534 | -0.21095 | 0.208277 | ANKRD23 |
| ILMN_2319825 | 0.002962 | 5.75E-05 | 0.358228 | -0.02859 | -0.28021 | -0.02113 | ACYP1 |
| ILMN_1791890 | 0.023626 | 0.00394 | -0.749 | -0.39513 | 1.69E-04 | -1.86859 | SPON1 |
| ILMN_1691760 | 0.004344 | 1.96E-04 | -0.11934 | 0.124894 | 0.157781 | -0.5001 | FAM45A |
| ILMN_1658175 | 0.025817 | 0.004499 | 0.580899 | 0.234645 | -0.39566 | 1.049053 | GGA1 |
| ILMN_1775171 | 0.006793 | 5.36E-04 | 0.331386 | -0.07333 | -0.2663 | 0.104384 | WIPF2 |
| ILMN_2078547 | 0.001381 | 1.01E-05 | -0.32161 | 0.087433 | 0.320234 | -0.82246 | HSPC268 |
| ILMN_2381168 | 0.00875 | 8.26E-04 | 0.826378 | 0.285269 | -0.24102 | 0.893031 | CTNNBIP1 |
| ILMN_1731621 | 3.24E-04 | 7.50E-07 | 0.887395 | 0.186973 | -0.31753 | 0.109941 | RXFP3 |
| ILMN_3246935 | 0.043695 | 0.009583 | 0.190822 | -0.21758 | -0.44836 | 0.382462 | SNORA74B |
| ILMN_1798233 | 0.034896 | 0.006965 | 0.252019 | 0.069526 | -0.19544 | -0.16135 | PSMB9 |
| ILMN_2374865 | 1.30E-04 | 1.97E-07 | 0.726935 | -0.0024 | -0.13697 | 2.761352 | ATF3 |
| ILMN_2328463 | 0.003753 | 1.25E-04 | 0.348443 | 0.112206 | -0.38897 | 0.081373 | INS-IGF2 |
| ILMN_1803303 | 0.00314 | 6.99E-05 | 0.4367 | 0.269627 | -0.28986 | 0.345609 | RAPSN |
| ILMN_3246356 | 0.010323 | 0.001092 | 0.362941 | -0.0591 | -0.23684 | 0.294176 | C5orf49 |
| ILMN_1658926 | 0.00998 | 0.001026 | 0.262017 | 0.054385 | -0.20853 | 0.018853 | NOTCH3 |
| ILMN_2069912 | 0.022221 | 0.003609 | 0.293614 | 0.032171 | -0.20946 | 0.116613 | MAGEA8 |
| ILMN_2298261 | 0.020024 | 0.00307 | 0.269029 | 0.246688 | -0.23723 | -0.10181 | RBM3 |
| ILMN_1667260 | 0.001985 | 2.19E-05 | 0.136239 | -0.03231 | -0.3234 | 0.306246 | MAPK3 |
| ILMN_2064926 | 0.014354 | 0.001866 | -0.14985 | -0.08954 | 0.234812 | -0.25312 | ITFG1 |
| ILMN_2214594 | 0.006957 | 5.57E-04 | 0.316582 | -0.03883 | -0.26356 | 0.148086 | DEFB107A |
| ILMN_1744299 | 0.016372 | 0.002274 | 0.750067 | -0.13325 | -0.37246 | 0.449471 | GPR110 |
| ILMN_2075436 | 0.003086 | 6.59E-05 | -0.30512 | 0.08494 | 0.068161 | -0.81157 | PDIA3P |
| ILMN_1755173 | 0.037486 | 0.00768 | -0.09856 | -0.6688 | 0.246584 | -0.61875 | PLEKHA4 |
| ILMN_1810891 | 0.028239 | 0.005129 | 0.293887 | 0.344365 | -0.04983 | -0.57387 | ZNF629 |
| ILMN_1707720 | 0.047095 | 0.010689 | 0.301507 | -0.22975 | -0.1635 | -0.04729 | SLC1A5 |
| ILMN_2087080 | 0.01007 | 0.001043 | 0.260236 | 0.032324 | -0.26466 | -0.1443 | RPL5 |
| ILMN_3308220 | 0.004944 | 2.60E-04 | 0.295015 | -0.01239 | -0.25598 | 0.164362 | MIR1293 |
| ILMN_1765460 | 0.004255 | 1.87E-04 | 0.345508 | 0.033652 | -0.23106 | -0.08915 | AMIGO1 |
| ILMN_2050434 | 0.0124 | 0.001477 | -0.00306 | 0.371484 | 0.217436 | -0.67878 | MGC16703 |
| ILMN_2097259 | 0.036061 | 0.007283 | 0.338135 | -0.18415 | -0.2534 | -0.2543 | CYP2U1 |
| ILMN_1670143 | 0.004065 | 1.59E-04 | 0.513194 | 0.041451 | -0.24307 | 0.269815 | C14orf70 |
| ILMN_1746232 | 0.044235 | 0.009764 | 0.119175 | 0.04811 | -0.2003 | 0.040853 | KITLG |
| ILMN_1748831 | 0.00554 | 3.48E-04 | 0.184282 | 0.047552 | -0.30765 | 0.100312 | PPP1R13B |
| ILMN_3236503 | 0.003444 | 9.53E-05 | 0.382316 | 0.013939 | -0.25735 | 0.164582 | PDCD11 |
| ILMN_1699440 | 0.012753 | 0.001555 | 0.164614 | -0.01978 | 0.336206 | -0.64048 | ZBTB47 |
| ILMN_1810785 | 0.039451 | 0.00825 | 0.195033 | -0.07164 | -0.03015 | 0.154339 | RNF11 |
| ILMN_1769155 | 0.013241 | 0.001644 | 0.262949 | 0.220404 | -0.29652 | 0.094139 | CYP2C8 |
| ILMN_1773485 | 0.043557 | 0.009547 | 0.269279 | 0.163656 | -0.09974 | 0.013103 | QKI |
| ILMN_1784216 | 0.003641 | 1.16E-04 | 0.366075 | 0.024634 | -0.29935 | 0.121018 | KRTAP19-6 |
| ILMN_1784436 | 0.005675 | 3.66E-04 | 0.101097 | 0.004702 | -0.34413 | 0.054282 | KIAA1688 |
| ILMN_2195015 | 0.029699 | 0.005538 | 0.353618 | 0.343921 | -0.21028 | 0.293769 | MRGPRE |
| ILMN_1801230 | 0.006808 | 5.38E-04 | 0.475239 | 0.355775 | -0.14318 | 0.146906 | DNASE1L1 |
| ILMN_3310720 | 0.010927 | 0.001201 | 0.28043 | -0.03638 | -0.19523 | 0.181674 | MIR1229 |
| ILMN_2276002 | 0.043237 | 0.009417 | 0.301248 | 0.026415 | -0.06721 | 0.039599 | CPNE1 |
| ILMN_1749930 | 0.011858 | 0.001378 | 0.043385 | -0.57616 | 0.335766 | 0.109679 | TMEM48 |
| ILMN_2307883 | 0.033512 | 0.006567 | 0.211774 | 0.011484 | -0.23055 | -0.01712 | ATP5J2 |
| ILMN_1767446 | 0.031354 | 0.00599 | 0.295477 | -0.00851 | -0.19581 | 0.005559 | RNF150 |
| ILMN_1754137 | 0.004034 | 1.53E-04 | 0.303953 | 0.056726 | -0.20228 | 0.26442 | CFHR1 |
| ILMN_1712095 | 0.008601 | 8.00E-04 | 0.302491 | 0.048524 | -0.22056 | 0.02344 | FOXO4 |
| ILMN_1727173 | 0.028226 | 0.005126 | 0.670191 | -0.33568 | -0.36097 | 0.401251 | SMARCD1 |
| ILMN_2158003 | 1.65E-04 | 2.72E-07 | 0.393184 | 0.323844 | -0.26713 | 0.924559 | KIAA1683 |
| ILMN_1685483 | 0.009225 | 9.08E-04 | 0.565805 | 0.035659 | -0.29331 | 0.203523 | FETUB |
| ILMN_1651574 | 0.005046 | 2.76E-04 | 0.376168 | 0.134821 | -0.20466 | -0.00528 | AQP3 |
| ILMN_3309854 | 6.45E-04 | 2.54E-06 | 0.645647 | -0.11203 | -0.35684 | 0.137259 | SNORD113-9 |
| ILMN_1803357 | 0.007602 | 6.48E-04 | 0.712089 | 0.042714 | -0.25843 | 0.116092 | FLJ40125 |
| ILMN_1686432 | 0.017972 | 0.002615 | -0.4717 | -0.43565 | 0.115942 | -0.87058 | ACYP1 |
| ILMN_1702797 | 0.004034 | 1.53E-04 | 0.629279 | -0.14784 | -0.12384 | 0.282287 | LOC541469 |
| ILMN_1741695 | 0.008853 | 8.49E-04 | 0.386093 | 0.025224 | -0.16735 | 0.11725 | COL12A1 |
| ILMN_1730937 | 0.008524 | 7.88E-04 | 0.067674 | 0.098816 | -0.16995 | 0.17943 | SLC39A13 |
| ILMN_1768470 | 0.003614 | 1.11E-04 | 0.139306 | 0.079731 | -0.26014 | 0.208876 | EIF4G1 |
| ILMN_1775508 | 0.008689 | 8.15E-04 | -0.02039 | 0.083771 | -0.22426 | 0.147617 | CYLD |
| ILMN_1699365 | 0.010072 | 0.001044 | -0.44269 | -0.5477 | 0.265908 | -0.24912 | SLC30A6 |
| ILMN_1723803 | 0.00272 | 4.65E-05 | 0.386562 | 0.186381 | -0.34185 | 0.019202 | SLC2A9 |
| ILMN_1683494 | 0.015236 | 0.00205 | 0.169568 | 0.138367 | -0.28577 | 0.017313 | TMEM154 |
| ILMN_1715668 | 0.003259 | 7.66E-05 | 0.362119 | 0.015563 | -0.36331 | 0.104604 | SLC39A4 |
| ILMN_1711617 | 0.020241 | 0.003126 | 0.155241 | 0.272077 | -0.21287 | -0.14313 | GMFG |
| ILMN_3223471 | 0.004626 | 2.24E-04 | 0.71905 | -0.07217 | -0.24395 | 0.437749 | PLXNA4 |
| ILMN_1701094 | 0.041403 | 0.008869 | 0.410752 | -0.46815 | 0.0012 | 0.658331 | CLN8 |
| ILMN_2233099 | 0.003485 | 9.87E-05 | 0.396024 | -0.03761 | -0.22291 | 0.083068 | SSRP1 |
| ILMN_1777487 | 0.036256 | 0.007346 | 0.130927 | -0.19255 | -0.36588 | -0.02631 | ZNF839 |
| ILMN_2051408 | 0.038935 | 0.008102 | 0.017126 | 0.096764 | -0.34564 | 0.068523 | PNPT1 |
| ILMN_1764723 | 0.009388 | 9.33E-04 | 0.371511 | 0.459706 | -0.27899 | -0.00148 | SH3PXD2B |
| ILMN_1712582 | 0.019873 | 0.003024 | 0.288755 | -0.12023 | -0.43371 | 0.034515 | FOLR1 |
| ILMN_1787897 | 0.005009 | 2.70E-04 | 0.158856 | 0.318327 | -0.38379 | -0.14786 | CXCL1 |
| ILMN_1700888 | 0.049955 | 0.011609 | 0.097176 | -0.17698 | 0.04913 | -0.36417 | ENPP1 |
| ILMN_1703477 | 0.003387 | 9.08E-05 | 0.249847 | 0.306736 | -0.22136 | -0.33994 | ARHGEF2 |
| ILMN_2115669 | 0.018982 | 0.002829 | 0.300011 | 0.049601 | -0.22958 | 0.10183 | SEMA4C |
| ILMN_2169983 | 0.022287 | 0.003625 | -0.02934 | 0.362813 | 0.187849 | -0.8913 | ATAD1 |
| ILMN_1709626 | 0.016505 | 0.002298 | 0.347584 | 0.017006 | -0.1497 | 0.17534 | FAM14B |
| ILMN_2070052 | 0.007054 | 5.71E-04 | 0.233785 | 0.121775 | -0.2758 | -0.10464 | LOC613037 |
| ILMN_2200836 | 0.012935 | 0.001587 | 1.542345 | -0.68067 | 0.15501 | -0.21028 | HSPB7 |
| ILMN_2188264 | 0.015692 | 0.002142 | 0.20961 | -0.14915 | -0.27049 | 1.190574 | CYR61 |
| ILMN_1712348 | 0.005158 | 2.96E-04 | 0.536428 | 0.206241 | -0.20237 | 0.28263 | GNB5 |
| ILMN_1717902 | 0.005885 | 3.94E-04 | 0.395471 | -0.03188 | -0.37962 | 0.10937 | ERBB2 |
| ILMN_1719455 | 0.026654 | 0.004703 | 0.30235 | 0.015582 | -0.23775 | 0.1099 | CRIP3 |
| ILMN_1791702 | 0.008076 | 7.19E-04 | 0.338989 | 0.07476 | -0.22322 | -0.024 | SMARCA2 |
| ILMN_1777917 | 0.002696 | 4.51E-05 | 0.48496 | -0.01134 | -0.3433 | 0.341482 | DSCR10 |
| ILMN_1676280 | 0.002761 | 4.85E-05 | 0.503151 | 0.013829 | -0.25864 | 0.214032 | NSUN3 |
| ILMN_2156982 | 0.041439 | 0.008881 | 0.079804 | 0.119605 | -0.25848 | 0.00974 | IMP4 |
| ILMN_1706326 | 0.027635 | 0.004957 | 0.245705 | -0.32114 | -0.06247 | 0.040534 | MRPL33 |
| ILMN_2047354 | 0.010177 | 0.001066 | -0.25519 | -0.68577 | 0.271223 | -0.60727 | C1orf97 |
| ILMN_1704665 | 0.019421 | 0.002913 | 0.140241 | -0.10526 | 0.284842 | -0.78673 | GPM6B |
| ILMN_1726565 | 0.003802 | 1.29E-04 | -0.13597 | -0.11144 | 0.193674 | -0.62939 | PIK3R2 |
| ILMN_1674135 | 0.005182 | 2.98E-04 | -0.08886 | -0.22141 | 0.064616 | -0.98277 | RALGPS1 |
| ILMN_3245346 | 0.0051 | 2.82E-04 | 0.297732 | 0.027992 | -0.26133 | 0.154052 | EFR3B |
| ILMN_1746533 | 0.003919 | 1.41E-04 | 0.282677 | 0.059822 | -0.32719 | 0.138291 | CDC27 |
| ILMN_1666733 | 3.50E-04 | 8.25E-07 | 0.617091 | -0.02605 | -0.34366 | 0.145451 | IL8 |
| ILMN_2184373 | 0.034739 | 0.006924 | 1.183376 | -0.08053 | -0.07682 | 0.477938 | IL8 |
| ILMN_1664919 | 0.004191 | 1.78E-04 | 0.310002 | 0.032196 | -0.2436 | 0.284671 | KCNK9 |
| ILMN_1793621 | 0.01094 | 0.001206 | 0.324139 | 0.015766 | -0.21545 | 0.171556 | ZFYVE27 |
| ILMN_3246065 | 0.007647 | 6.56E-04 | 0.148483 | 0.024448 | -0.3384 | 0.186783 | CCDC151 |
| ILMN_1688372 | 0.014694 | 0.001932 | 0.287634 | 0.045825 | -0.26284 | -0.00292 | C3orf17 |
| ILMN_3261111 | 0.005639 | 3.62E-04 | 0.317622 | 0.065692 | -0.27934 | 0.16888 | IP6K3 |
| ILMN_1802753 | 0.014675 | 0.001926 | 0.113968 | 0.149007 | -0.30815 | -0.0157 | TSSC4 |
| ILMN_1686562 | 0.044705 | 0.009898 | 0.272401 | 0.081658 | -0.30899 | 0.243137 | KIF13B |
| ILMN_2234873 | 0.011531 | 0.001318 | 0.039614 | -0.70892 | 0.234487 | 0.549493 | NME2 |
| ILMN_1743911 | 0.017467 | 0.00251 | 0.198799 | -0.03406 | -0.23305 | 0.034481 | SLC25A39 |
| ILMN_3244457 | 0.010089 | 0.001048 | -0.53352 | -0.15165 | 0.110669 | -0.56158 | EHBP1L1 |
| ILMN_1688423 | 0.007828 | 6.82E-04 | -0.10173 | 0.860844 | -0.25639 | -0.49185 | FCER1A |
| ILMN_1785198 | 0.023507 | 0.003919 | -0.62358 | -0.29061 | 0.325909 | -0.62609 | POLE3 |
| ILMN_1737513 | 0.007543 | 6.40E-04 | 0.255839 | 0.009655 | -0.2793 | 0.143431 | MYADML |
| ILMN_1759487 | 0.006808 | 5.38E-04 | 0.344149 | 0.335065 | -0.06155 | 1.952562 | EGFLAM |
| ILMN_2143822 | 0.009442 | 9.44E-04 | 0.231677 | 0.078761 | -0.25073 | 0.006072 | ZNF148 |
| ILMN_2374036 | 0.005237 | 3.08E-04 | -0.09463 | -0.19622 | 0.304947 | -0.4534 | CTSL1 |
| ILMN_1740217 | 0.011001 | 0.001218 | 0.196082 | 0.137688 | -0.26207 | -0.05566 | HACE1 |
| ILMN_1776577 | 0.032706 | 0.006358 | 0.238752 | -0.10015 | -0.22165 | -0.11958 | DSCC1 |
| ILMN_2209551 | 0.023851 | 0.003997 | 0.550142 | 0.877644 | -0.21812 | 0.130781 | SIGLECP3 |
| ILMN_1775093 | 0.004546 | 2.17E-04 | 0.005816 | 0.058632 | -0.35802 | 0.109885 | SHOX |
| ILMN_1773243 | 0.002998 | 6.04E-05 | 0.358554 | 0.058745 | -0.23583 | 0.168332 | ACER1 |
| ILMN_2114185 | 0.004255 | 1.88E-04 | 0.279199 | 0.082634 | -0.25736 | 0.181647 | C1orf104 |
| ILMN_2278433 | 0.021512 | 0.003416 | 0.283119 | 0.184668 | -0.16862 | 0.043643 | LOC285074 |
| ILMN_1781517 | 0.008221 | 7.43E-04 | 0.418712 | -0.04852 | -0.23142 | 0.141586 | LIPF |
| ILMN_1774982 | 0.003144 | 7.04E-05 | 0.093053 | 0.59039 | -0.29938 | 0.069609 | CDC42EP5 |
| ILMN_3234735 | 0.006386 | 4.63E-04 | 0.463679 | -0.10776 | -0.00894 | -0.34276 | ERI2 |
| ILMN_2255133 | 0.042767 | 0.009267 | -0.25612 | 0.642835 | -0.60817 | -0.46122 | BCL11A |
| ILMN_1790014 | 0.022854 | 0.003762 | 0.278632 | -0.07962 | -0.27442 | 0.407364 | METRNL |
| ILMN_2320574 | 0.031678 | 0.00608 | 0.178607 | 0.034528 | -0.19492 | 0.060473 | RGPD5 |
| ILMN_1806906 | 0.002609 | 4.19E-05 | 0.29423 | -0.00502 | -0.19639 | -0.01306 | SSR3 |
| ILMN_1706521 | 0.003258 | 7.55E-05 | 0.20577 | 0.037874 | -0.40866 | -0.0991 | CSNK1G2 |
| ILMN_1658259 | 0.019201 | 0.002875 | 0.277002 | -0.4547 | -0.09924 | 0.280544 | DRG1 |
| ILMN_1806464 | 0.004963 | 2.62E-04 | 0.206218 | 0.02104 | -0.24903 | 0.105697 | T-SP1 |
| ILMN_2280816 | 0.020131 | 0.003098 | 0.303909 | 0.002524 | -0.23032 | 0.107709 | CEMP1 |
| ILMN_1703427 | 0.006643 | 5.08E-04 | 0.298347 | 0.049125 | -0.15673 | 0.15247 | SON |
| ILMN_1685057 | 0.021262 | 0.003355 | -0.36804 | -0.20144 | 0.36826 | 0.655905 | SLC22A4 |
| ILMN_1789089 | 0.011248 | 0.00126 | 0.855158 | 0.016971 | -0.18393 | 0.148546 | DCTN1 |
| ILMN_3310301 | 0.008417 | 7.71E-04 | 0.361877 | 0.056982 | -0.21021 | 0.034149 | MIR342 |
| ILMN_1785891 | 0.004848 | 2.45E-04 | -0.30321 | -0.47595 | 0.286084 | -1.02457 | PRKD1 |
| ILMN_1740813 | 0.00348 | 9.80E-05 | 0.319804 | -0.09014 | -0.29995 | 0.020234 | KRT37 |
| ILMN_1771201 | 0.003568 | 1.05E-04 | 0.199899 | -0.02557 | -0.41897 | -0.07607 | TNRC6B |
| ILMN_1718336 | 0.006725 | 5.25E-04 | 0.34304 | -0.04843 | -0.2287 | 0.178715 | C7orf50 |
| ILMN_2052135 | 0.04695 | 0.010634 | 0.06772 | -0.34666 | -0.33619 | 0.639155 | LOH3CR2A |
| ILMN_1812432 | 0.012485 | 0.001493 | 0.600319 | 0.166027 | -0.2301 | 0.057258 | TIRAP |
| ILMN_1745299 | 0.002182 | 2.81E-05 | 0.330193 | 0.006625 | -0.3043 | 0.129167 | FABP7 |
| ILMN_2335893 | 0.036156 | 0.00731 | 0.025027 | -0.34295 | -0.44502 | 0.582625 | FRS2 |
| ILMN_1815578 | 0.034828 | 0.006946 | -0.05238 | 0.489305 | 0.132211 | -0.69042 | ZNF223 |
| ILMN_1706502 | 0.015275 | 0.002066 | 0.239428 | 0.06882 | -0.23144 | -0.06593 | EIF2AK2 |
| ILMN_1775269 | 0.047333 | 0.010774 | 0.046481 | -0.02991 | 0.056695 | -0.75457 | EID1 |
| ILMN_2402805 | 0.017551 | 0.002531 | 0.271886 | -0.13615 | -0.0311 | -0.70476 | TRPC4AP |
| ILMN_1677843 | 0.017467 | 0.002509 | 0.324621 | 0.082108 | -0.11892 | -0.45134 | RAB24 |
| ILMN_3243664 | 0.034376 | 0.006824 | 0.19187 | 0.19729 | -0.28028 | 0.152146 | LOC440353 |
| ILMN_1751708 | 0.045003 | 0.009998 | 0.268601 | -0.15266 | -0.13814 | -0.06451 | ITM2B |
| ILMN_2207720 | 0.028807 | 0.005287 | -0.09313 | -0.07976 | 0.276796 | -0.62954 | ITM2B |
| ILMN_1734176 | 0.009992 | 0.001028 | 0.418562 | 0.174161 | -0.17331 | 0.177559 | CGA |
| ILMN_1693905 | 0.002863 | 5.32E-05 | 0.366996 | 0.017197 | -0.27525 | 0.118671 | HAT1 |
| ILMN_1778488 | 0.004309 | 1.93E-04 | -0.23616 | 0.145705 | 0.263757 | -0.33481 | WDR41 |
| ILMN_1774589 | 0.040606 | 0.008601 | 0.304473 | 0.241333 | -0.1452 | -0.1287 | IQCC |
| ILMN_1798172 | 0.028478 | 0.005196 | 0.105973 | -0.59292 | 0.086866 | 0.199055 | IPO4 |
| ILMN_1712859 | 0.0065 | 4.88E-04 | 0.37605 | -0.044 | -0.24413 | 0.249952 | FAM89A |
| ILMN_1696975 | 0.028118 | 0.005089 | -0.58513 | -0.28744 | 0.097919 | -0.73902 | USP1 |
| ILMN_1801143 | 0.005392 | 3.27E-04 | 0.279103 | 0.033859 | -0.24454 | 0.255119 | LOXHD1 |
| ILMN_1747019 | 0.003312 | 8.43E-05 | 0.785188 | 0.010838 | -0.20129 | 0.124761 | PDYN |
| ILMN_1774708 | 0.014334 | 0.001861 | 0.317114 | 0.016822 | -0.16713 | 0.047556 | ORMDL2 |
| ILMN_2404657 | 0.006677 | 5.16E-04 | 0.341737 | 0.028854 | -0.3118 | 0.105488 | ALAD |
| ILMN_1692623 | 0.013995 | 0.001791 | 0.499999 | 0.132048 | -0.24646 | -0.05157 | LPHN3 |
| ILMN_1752658 | 0.010977 | 0.001213 | 0.280611 | 0.14657 | -0.1668 | 0.215636 | NGFR |
| ILMN_1722022 | 0.004179 | 1.72E-04 | 0.855357 | 0.382186 | -0.1741 | 0.19035 | MAGEE1 |
| ILMN_1703072 | 0.004626 | 2.24E-04 | 0.344162 | 0.03168 | -0.32059 | 0.235954 | CREB3 |
| ILMN_2347298 | 0.023994 | 0.004025 | 0.190624 | -0.00616 | -0.3817 | 0.60042 | TSPYL2 |
| ILMN_1657554 | 0.005246 | 3.10E-04 | 0.067464 | -0.16104 | -0.14566 | 0.64632 | TSPYL2 |
| ILMN_1670037 | 0.037008 | 0.00754 | 0.042103 | -0.52918 | 0.278026 | -0.75916 | POLR2L |
| ILMN_1733904 | 0.020743 | 0.003245 | 0.560623 | 0.009632 | -0.07828 | 0.481166 | C20orf114 |
| ILMN_1799224 | 0.031122 | 0.005919 | 0.068347 | 0.239293 | -0.3845 | -0.03256 | FANCA |
| ILMN_1768598 | 0.011506 | 0.001313 | 0.486363 | 0.226685 | -0.30883 | 0.195027 | LAIR1 |
| ILMN_2276758 | 0.001086 | 5.78E-06 | -0.37299 | 0.214378 | 0.372628 | -0.70038 | POFUT1 |
| ILMN_1679460 | 0.019116 | 0.002855 | -0.3609 | 0.104929 | 0.008827 | -0.42564 | PPFIBP1 |
| ILMN_2193315 | 0.04006 | 0.008418 | 0.319272 | -0.40616 | 0.056523 | -0.03069 | C14orf143 |
| ILMN_1770682 | 0.010943 | 0.001206 | -0.00874 | 0.277369 | -0.24063 | -0.14067 | TEP1 |
| ILMN_1744212 | 0.009708 | 9.81E-04 | 0.401147 | 0.474051 | -0.26935 | -0.20164 | INPP5D |
| ILMN_2222695 | 0.046275 | 0.010407 | 0.367801 | -0.11606 | -0.11777 | -0.32012 | ZNF491 |
| ILMN_1717078 | 0.040481 | 0.008565 | -0.00451 | -0.55475 | 0.007753 | 0.277373 | DNAJC16 |
| ILMN_1778308 | 0.012379 | 0.001473 | -0.14284 | -0.04983 | -0.04874 | -0.62863 | COL4A4 |
| ILMN_1691433 | 0.007633 | 6.52E-04 | 0.322898 | -0.03231 | -0.28901 | 0.080286 | PIK3R1 |
| ILMN_1726786 | 0.028383 | 0.005168 | -0.15236 | 0.140233 | 0.33495 | -0.41309 | TNRC6B |
| ILMN_2194448 | 0.027303 | 0.004874 | -0.30094 | -0.21077 | 0.222333 | -0.29682 | STT3B |
| ILMN_2081645 | 0.004191 | 1.78E-04 | 0.490454 | 0.036517 | -0.24416 | 0.181575 | RSPO2 |
| ILMN_3237685 | 0.044806 | 0.009931 | 0.009274 | 0.036862 | -0.33028 | -0.04179 | SNORD70 |
| ILMN_1797030 | 0.00716 | 5.85E-04 | 0.32372 | 0.001698 | -0.26956 | 0.131364 | FGF18 |
| ILMN_1731048 | 0.014291 | 0.001848 | 0.224593 | 0.099355 | -0.17492 | 0.261798 | TLR1 |
| ILMN_2331062 | 0.015801 | 0.002164 | 0.727 | 0.308942 | -0.2388 | 0.210897 | CBFA2T2 |
| ILMN_1676088 | 0.020112 | 0.003093 | -0.10026 | -0.48888 | 0.422284 | -0.38237 | MSRB3 |
| ILMN_1658920 | 0.014583 | 0.001908 | 0.411053 | 0.11748 | -0.31594 | -0.12984 | MCTP1 |
| ILMN_3244526 | 0.020317 | 0.003143 | 0.287818 | 0.022882 | 0.054214 | -1.23767 | TCTN3 |
| ILMN_2363658 | 0.004191 | 1.74E-04 | 0.428856 | 0.14965 | -0.35708 | -0.25453 | PXDN |
| ILMN_1662364 | 0.007015 | 5.64E-04 | 0.237962 | -0.03035 | -0.22065 | 0.165463 | AARS |
| ILMN_1651405 | 0.005131 | 2.92E-04 | 0.231232 | 0.112253 | -0.2228 | 0.05712 | BRD9 |
| ILMN_3249211 | 0.007647 | 6.56E-04 | 0.608918 | -0.13185 | -0.27863 | 0.11095 | SNORD78 |
| ILMN_1758626 | 0.0123 | 0.001459 | 0.211639 | 0.096111 | -0.17459 | 0.112089 | IDS |
| ILMN_1745329 | 0.024822 | 0.004226 | 0.3578 | 0.145044 | -0.19733 | 0.054567 | PRR14 |
| ILMN_1732885 | 0.024892 | 0.004249 | 0.032429 | 0.32342 | -0.04456 | -1.41565 | BTBD12 |
| ILMN_1813325 | 0.01466 | 0.001924 | 0.040351 | -0.00497 | -0.44511 | 0.075685 | PDE11A |
| ILMN_1795564 | 0.021887 | 0.003507 | 0.405534 | 0.176431 | -0.15088 | 0.155705 | C11orf84 |
| ILMN_1678966 | 0.003917 | 1.40E-04 | 0.452829 | -0.16673 | -0.15434 | -0.06852 | SNRPF |
| ILMN_1754752 | 0.0143 | 0.001853 | 0.392607 | -0.00791 | -0.28235 | 0.143501 | CNNM2 |
| ILMN_1765258 | 0.021499 | 0.003412 | 0.219111 | 0.060281 | -0.14411 | 0.138521 | HLA-E |
| ILMN_1794782 | 0.001226 | 7.29E-06 | -0.30475 | -0.43135 | 0.207599 | -1.13629 | ABCG1 |
| ILMN_1804738 | 0.005573 | 3.53E-04 | 0.261957 | -0.00756 | -0.3672 | 0.072856 | MEFV |
| ILMN_1688765 | 0.011546 | 0.00132 | 0.336881 | 0.065087 | -0.23661 | -0.21616 | ZNF221 |
| ILMN_1701832 | 0.00719 | 5.91E-04 | 0.209776 | 0.00499 | -0.26123 | 0.243908 | RPL19 |
| ILMN_1692754 | 0.046568 | 0.01051 | -0.37128 | 0.087986 | 0.001374 | 0.540027 | TMEM49 |
| ILMN_1727258 | 0.009709 | 9.82E-04 | 0.363481 | 0.034609 | -0.25212 | 0.106691 | AMELY |
| ILMN_1721136 | 0.015338 | 0.002078 | 0.048798 | 0.110364 | -0.37476 | 0.169098 | H6PD |
| ILMN_1719033 | 0.007088 | 5.75E-04 | 0.459001 | 0.043202 | -0.2927 | 0.106991 | PATE1 |
| ILMN_3241756 | 0.017338 | 0.00248 | 0.244272 | -0.09265 | -0.27962 | -0.00557 | FAM136B |
| ILMN_2071641 | 0.01481 | 0.001955 | -0.16964 | -0.35528 | 0.343879 | -0.64328 | KCNK1 |
| ILMN_2309848 | 0.004976 | 2.64E-04 | 0.212294 | 0.260153 | -0.31445 | -0.23471 | FXYD5 |
| ILMN_3247636 | 0.002631 | 4.35E-05 | 0.392503 | -0.10478 | -0.28684 | 0.230827 | SCARNA14 |
| ILMN_1798827 | 0.023639 | 0.003944 | 0.079837 | 0.153567 | 0.283723 | -0.4058 | SRBD1 |
| ILMN_1679382 | 0.04166 | 0.008956 | 0.113806 | 0.015958 | -0.22437 | 0.093436 | CCT2 |
| ILMN_1807913 | 0.008781 | 8.32E-04 | 0.311178 | -0.02962 | -0.27611 | 0.165854 | ART1 |
| ILMN_1774062 | 0.045313 | 0.010101 | 0.146749 | -0.30155 | 0.024281 | -0.33616 | SLC25A5 |
| ILMN_1770102 | 0.024937 | 0.004267 | -0.09397 | 0.294832 | -0.01465 | -0.91747 | FLJ21687 |
| ILMN_2132458 | 0.005595 | 3.55E-04 | 0.268794 | 0.069433 | -0.27695 | 0.068099 | CLDN4 |
| ILMN_1762545 | 0.002717 | 4.63E-05 | 0.471432 | 0.048136 | -0.36122 | 0.041799 | LOC158572 |
| ILMN_1688178 | 0.001409 | 1.07E-05 | 0.338058 | 0.010828 | -0.2977 | 0.275635 | RRP7A |
| ILMN_1663171 | 2.74E-04 | 6.09E-07 | 0.631319 | 0.013542 | -0.30975 | 0.19817 | MATN3 |
| ILMN_1711886 | 0.029296 | 0.005436 | 0.116907 | -0.28877 | -0.26006 | 0.291465 | ALG3 |
| ILMN_1789351 | 0.006645 | 5.09E-04 | 0.143394 | 0.025351 | -0.21951 | 0.17382 | SUV39H2 |
| ILMN_1813657 | 0.009408 | 9.35E-04 | 0.335116 | -0.13991 | -0.12855 | 0.102146 | PHF20 |
| ILMN_3307719 | 0.02651 | 0.004668 | 0.28187 | 0.096096 | -0.11118 | -0.54868 | ZNF490 |
| ILMN_1767322 | 0.041032 | 0.008751 | 0.457647 | 0.120484 | -0.11555 | 0.750579 | EDAR |
| ILMN_3308275 | 0.031775 | 0.006107 | 0.123392 | -0.02705 | -0.4921 | 0.088089 | SNORD113-8 |
| ILMN_1798496 | 0.006645 | 5.10E-04 | 0.132446 | 0.428038 | 0.104236 | -0.65355 | HOXB8 |
| ILMN_1705546 | 0.040081 | 0.008433 | 0.64495 | 0.578034 | -0.39312 | 1.161926 | POU5F1 |
| ILMN_1740048 | 0.014757 | 0.001943 | 0.440988 | 0.217843 | -0.20543 | 0.118257 | MYOD1 |
| ILMN_1810729 | 0.02039 | 0.003163 | -0.68787 | -0.34139 | 0.295786 | 0.149545 | UBL3 |
| ILMN_2347323 | 0.01259 | 0.001518 | 0.218947 | -0.08693 | -0.11064 | -0.40799 | DNAJC21 |
| ILMN_2235975 | 0.031571 | 0.006048 | 0.356821 | -0.01243 | -0.27356 | 0.275869 | FXYD5 |
| ILMN_1704286 | 0.025162 | 0.004328 | -0.36893 | 0.049335 | -0.05198 | -1.29171 | FXYD5 |
| ILMN_1772981 | 0.010187 | 0.001067 | 0.287863 | -0.0025 | -0.22559 | 0.007891 | EPN1 |
| ILMN_3235567 | 0.036474 | 0.007405 | 0.338055 | -0.10395 | -0.24399 | 0.071126 | LOC150568 |
| ILMN_1742330 | 0.001835 | 1.76E-05 | 0.006465 | -0.0668 | -0.02729 | -0.85622 | PLXNB1 |
| ILMN_1771051 | 0.030629 | 0.005792 | 0.256353 | -0.4389 | 0.308511 | -0.4966 | RPL29 |
| ILMN_1737517 | 0.03696 | 0.007528 | 0.074029 | -0.29965 | 0.286677 | -0.47424 | RPL29 |
| ILMN_1701269 | 0.00256 | 4.00E-05 | 0.332233 | -0.09543 | -0.11545 | 0.224305 | ATP5C1 |
| ILMN_1736353 | 0.010688 | 0.001157 | 0.371318 | 0.100346 | -0.21077 | 0.041132 | PSMC1 |
| ILMN_1690695 | 0.001091 | 6.08E-06 | 0.036635 | -0.05746 | 0.16013 | -0.69669 | PEX11A |
| ILMN_1680343 | 0.036711 | 0.007466 | -0.54218 | 0.254605 | 0.307325 | -1.29606 | AGL |
| ILMN_1780913 | 0.008134 | 7.27E-04 | 0.156509 | 0.202609 | -0.32346 | 0.400583 | PPP2R5C |
| ILMN_1670829 | 0.023671 | 0.003954 | 0.3844 | 0.040264 | -0.2332 | 0.113362 | ASB17 |
| ILMN_2210101 | 0.003791 | 1.27E-04 | 0.740859 | -0.09159 | -0.25598 | 0.157209 | ASB17 |
| ILMN_1757180 | 0.008149 | 7.29E-04 | 0.54909 | -0.08222 | -0.16567 | 0.194286 | WDR49 |
| ILMN_3311045 | 0.006251 | 4.42E-04 | 0.387932 | 0.256567 | -0.25008 | 0.067303 | MIR216A |
| ILMN_1727778 | 0.030013 | 0.005622 | 0.240027 | -0.61714 | 0.356106 | 0.111014 | NTNG1 |
| ILMN_1726108 | 0.003279 | 8.18E-05 | 0.409101 | -0.0332 | -0.18071 | 0.066797 | LASS2 |
| ILMN_1765711 | 0.003277 | 7.80E-05 | 0.36036 | 0.005469 | -0.27627 | -0.01295 | FBXO11 |
| ILMN_1652348 | 0.027191 | 0.004836 | 0.358784 | 0.01172 | -0.27086 | 0.085506 | C4orf50 |
| ILMN_1786872 | 0.031193 | 0.005947 | 0.305856 | -0.374 | -0.47332 | 0.48264 | C11orf31 |
| ILMN_1692188 | 0.007403 | 6.20E-04 | 0.426245 | 0.132624 | -0.2158 | 0.157656 | GGTLC1 |
| ILMN_1694983 | 0.042075 | 0.009075 | 0.116598 | 0.247184 | -0.25825 | 0.338656 | DDX20 |
| ILMN_1660321 | 0.015066 | 0.002007 | 0.196898 | -0.00965 | -0.31059 | -0.04298 | CDH24 |
| ILMN_3238680 | 0.033736 | 0.006635 | 0.446364 | -0.21867 | -0.11338 | 0.411353 | C7orf55 |
| ILMN_1804316 | 0.041372 | 0.008854 | 0.405522 | 0.344374 | -0.02347 | -0.90726 | TCAP |
| ILMN_3310588 | 0.028863 | 0.005311 | 0.471357 | 0.438874 | -0.17483 | 0.574049 | MIR623 |
| ILMN_1739587 | 0.005884 | 3.93E-04 | 0.476262 | -0.07031 | -0.37286 | -0.11978 | UTY |
| ILMN_1680081 | 0.006645 | 5.10E-04 | 0.058604 | -0.06745 | -0.27083 | 0.081782 | B3Gn-T6 |
| ILMN_1720124 | 0.001764 | 1.60E-05 | 0.192426 | 0.138161 | -0.27906 | 0.074988 | RCC2 |
| ILMN_1738657 | 0.008208 | 7.39E-04 | -0.86981 | -0.18244 | 0.286126 | -0.2484 | SATB2 |
| ILMN_3237462 | 0.040301 | 0.008506 | 0.113858 | -0.18588 | -0.19412 | 0.17494 | IDO2 |
| ILMN_1762629 | 0.045793 | 0.010248 | 0.160134 | 0.125331 | -0.33179 | -0.15615 | ANO9 |
| ILMN_2395474 | 0.008061 | 7.17E-04 | -0.30744 | 0.067297 | 0.041382 | -0.44053 | REV1 |
| ILMN_1689176 | 0.031176 | 0.005938 | -0.24253 | -0.42481 | 0.421938 | 0.649884 | C4orf31 |
| ILMN_1659770 | 0.041715 | 0.00898 | -0.09662 | -0.50073 | 0.132971 | -0.60143 | KCNJ15 |
| ILMN_1720708 | 0.017009 | 0.002406 | 0.194993 | 0.038064 | -0.17693 | 0.141308 | CSNK1D |
| ILMN_1764158 | 0.017539 | 0.002527 | 0.169656 | 0.240134 | -0.24882 | -0.00478 | NLGN2 |
| ILMN_2152711 | 0.003919 | 1.41E-04 | -0.04495 | -0.31017 | 0.478058 | -0.93458 | ACVR2A |
| ILMN_1810055 | 0.005868 | 3.91E-04 | -0.24699 | -0.48393 | 0.344319 | -0.67661 | ITFG3 |
| ILMN_1753547 | 0.035338 | 0.007096 | 0.3432 | -0.04587 | -0.2242 | -0.03612 | STAT5A |
| ILMN_1735333 | 0.03893 | 0.008099 | -0.13994 | 0.007199 | 0.379756 | -0.82188 | NBR1 |
| ILMN_1651254 | 0.031682 | 0.006082 | 0.282279 | 0.006184 | -0.09458 | -0.00414 | LPP |
| ILMN_2250923 | 0.029246 | 0.005414 | 0.362836 | 0.045542 | -0.17567 | -0.03121 | FOXP1 |
| ILMN_1724162 | 0.004473 | 2.08E-04 | 0.316157 | -0.12009 | -0.18758 | 0.414024 | ARX |
| ILMN_2363058 | 0.008208 | 7.38E-04 | 0.147152 | -0.05959 | -0.28672 | 0.235339 | PAOX |
| ILMN_3237966 | 0.017254 | 0.002461 | 0.262641 | -0.00757 | -0.22573 | -0.01102 | FBXL11 |
| ILMN_2073307 | 0.043333 | 0.009463 | 0.056646 | 0.670903 | 0.31009 | -0.85748 | IL10 |
| ILMN_1726081 | 0.006643 | 5.07E-04 | 0.324241 | 0.116228 | -0.47247 | 0.118264 | SLC26A9 |
| ILMN_1748884 | 0.021414 | 0.003391 | 0.254137 | 0.034381 | -0.20737 | 0.024721 | TOB2 |
| ILMN_1799969 | 0.027311 | 0.004879 | 0.228142 | -0.04597 | -0.08251 | -0.50854 | SNAPIN |
| ILMN_1663893 | 0.028666 | 0.005257 | 0.33135 | 0.018234 | -0.22622 | 0.156912 | C21orf128 |
| ILMN_2225511 | 0.020583 | 0.003205 | 0.216487 | 0.04694 | -0.33006 | 0.02381 | BRMS1L |
| ILMN_1685645 | 0.006493 | 4.85E-04 | 0.495528 | -0.09079 | -0.33546 | 0.056222 | MYOHD1 |
| ILMN_1704078 | 0.005077 | 2.80E-04 | 0.714812 | 0.035902 | -0.24941 | 0.142075 | ACTL8 |
| ILMN_1676846 | 2.50E-07 | 7.09E-11 | 0.101755 | 0.24578 | -0.18775 | 0.485473 | ABCE1 |
| ILMN_3239272 | 0.004882 | 2.51E-04 | 0.436271 | -0.09955 | -0.29616 | 0.244964 | SNORD71 |
| ILMN_1724306 | 0.014292 | 0.00185 | 0.293188 | -0.05606 | -0.26818 | 0.020714 | CACNG1 |
| ILMN_1674265 | 7.25E-04 | 3.11E-06 | 0.482816 | -0.16418 | -0.2361 | 0.055375 | AVIL |
| ILMN_2098705 | 0.004528 | 2.13E-04 | 0.566358 | 0.012204 | -0.25815 | 0.211581 | FAM9C |
| ILMN_2307721 | 0.033979 | 0.006706 | 0.627102 | 0.027454 | -0.19607 | 0.240625 | IL22RA2 |
| ILMN_1811927 | 0.02463 | 0.004174 | 0.263447 | 0.28539 | -0.28194 | 0.099206 | OR2W3 |
| ILMN_1755658 | 0.03263 | 0.006338 | 0.589663 | 0.366543 | -0.07461 | -0.20345 | ABI3 |
| ILMN_1793578 | 0.033516 | 0.006569 | -0.33075 | 0.15257 | 0.171675 | -0.68673 | ZFP37 |
| ILMN_1766154 | 0.006231 | 4.39E-04 | 0.300308 | 0.151816 | -0.21914 | 0.137005 | MRPL30 |
| ILMN_1697546 | 0.019873 | 0.003028 | 0.035016 | -0.14779 | 0.309569 | -0.30212 | BRCC3 |
| ILMN_1746516 | 0.006471 | 4.79E-04 | 0.287446 | -0.00376 | -0.25515 | 0.092129 | RPS25 |
| ILMN_3306742 | 0.045016 | 0.010003 | 0.19767 | 0.004053 | -0.28284 | -0.04596 | SIGMAR1 |
| ILMN_2398489 | 0.022111 | 0.003577 | 0.297479 | -0.01713 | -0.08369 | -0.14673 | SIGMAR1 |
| ILMN_1815261 | 0.001419 | 1.10E-05 | -0.08651 | -0.31548 | 0.897938 | -0.62989 | PDIA4 |
| ILMN_1654606 | 0.005158 | 2.96E-04 | 0.428772 | 0.020512 | -0.24129 | 0.203156 | ZDHHC22 |
| ILMN_1750400 | 0.005773 | 3.81E-04 | 0.280624 | 0.077289 | -0.19213 | -0.17173 | C19orf66 |
| ILMN_1795463 | 0.008026 | 7.12E-04 | 0.366857 | 0.013159 | -0.24441 | 0.085491 | MORC4 |
| ILMN_1700847 | 0.034625 | 0.006888 | 0.222431 | 0.406774 | 0.185009 | -0.24803 | PMS1 |
| ILMN_2113738 | 0.03257 | 0.00632 | -0.11353 | 0.30962 | 0.088241 | -0.73956 | C8orf45 |
| ILMN_1666206 | 0.043324 | 0.009442 | -0.11144 | 0.026618 | -0.33443 | 0.20979 | GSDMB |
| ILMN_1712046 | 0.002418 | 3.39E-05 | 0.317023 | 0.135178 | -0.18817 | -0.27767 | CPXM1 |
| ILMN_1764383 | 0.006454 | 4.75E-04 | -0.64198 | -0.09055 | 0.191378 | -0.05587 | MCOLN1 |
| ILMN_1810107 | 0.009318 | 9.22E-04 | 0.377336 | -0.00748 | -0.26009 | 0.156747 | C17orf74 |
| ILMN_2295844 | 0.018425 | 0.002715 | 0.098213 | 0.083808 | -0.42678 | 0.059491 | VAMP1 |
| ILMN_1737611 | 0.024979 | 0.004275 | -8.86E-04 | -0.33734 | 0.080994 | -1.23067 | VAMP1 |
| ILMN_3238745 | 0.007944 | 7.00E-04 | 0.266438 | 0.098243 | -0.35606 | -0.05634 | SNORA55 |
| ILMN_1694798 | 0.044504 | 0.009836 | 0.248076 | 0.108702 | -0.19395 | 0.012918 | C5orf28 |
| ILMN_1702866 | 0.008866 | 8.55E-04 | -0.41958 | 0.064461 | 0.330441 | -0.26482 | FLJ22639 |
| ILMN_1695991 | 0.022957 | 0.003787 | 0.257569 | 0.26302 | -0.3292 | -0.07158 | COLQ |
| ILMN_1757882 | 0.033052 | 0.006447 | 0.192466 | 0.068261 | -0.23681 | -0.24494 | PPP1R16A |
| ILMN_1776435 | 0.015279 | 0.002068 | -0.16913 | 0.215199 | -0.33678 | 0.518432 | GPRIN3 |
| ILMN_1720829 | 0.001465 | 1.16E-05 | 0.400928 | 0.147135 | -0.30908 | 1.032379 | ZFP36 |
| ILMN_1727438 | 0.003503 | 1.00E-04 | 0.390353 | -0.06394 | -0.25381 | 0.185778 | KCND3 |
| ILMN_2337856 | 0.015236 | 0.002052 | 0.159597 | 0.582783 | -0.44035 | 0.813905 | KCND3 |
| ILMN_1794490 | 0.038266 | 0.007887 | 0.161175 | -0.31317 | 0.120718 | -0.6623 | FLJ10781 |
| ILMN_1751559 | 0.029257 | 0.005418 | 1.350822 | -0.17076 | 0.066118 | 1.492781 | C16orf30 |
| ILMN_1688639 | 0.039317 | 0.008209 | -0.18435 | -0.10263 | -0.10445 | -1.0438 | FBXL2 |
| ILMN_1696355 | 0.004976 | 2.64E-04 | 0.309212 | -0.05117 | -0.2562 | 0.175125 | OR5M3 |
| ILMN_1723674 | 0.014712 | 0.001935 | 0.312253 | -0.14595 | -0.26756 | 0.255254 | NKAIN3 |
| ILMN_2263054 | 0.016236 | 0.002251 | 0.29377 | 0.066247 | -0.28017 | 0.073188 | FEZ1 |
| ILMN_1789733 | 0.004944 | 2.60E-04 | 0.213182 | 0.06838 | -0.20498 | 0.148712 | CLIP3 |
| ILMN_1707258 | 0.002102 | 2.55E-05 | 0.44067 | -0.04599 | -0.26617 | 0.196116 | UCN |
| ILMN_1745904 | 0.015033 | 0.001998 | -0.34929 | -0.23854 | 0.076594 | -1.24582 | CCDC6 |
| ILMN_2300186 | 0.0051 | 2.84E-04 | 0.296026 | -0.07271 | -0.09007 | -0.24503 | DYNLL1 |
| ILMN_2377150 | 0.038372 | 0.007915 | 0.335724 | -0.06198 | -0.16053 | -0.04432 | WDHD1 |
| ILMN_2385566 | 0.038919 | 0.008088 | 0.352375 | -0.31828 | -0.0298 | 0.428555 | PHYH |
| ILMN_1744003 | 0.003688 | 1.20E-04 | 0.533313 | 0.238609 | -0.17309 | 0.740342 | SLC44A5 |
| ILMN_1698404 | 0.047756 | 0.010907 | -0.5521 | 0.203887 | -0.27518 | 0.580341 | ERN1 |
| ILMN_1661196 | 0.008858 | 8.52E-04 | 0.273849 | 0.246775 | -0.42655 | 0.181496 | CSF2RA |
| ILMN_1688031 | 0.022373 | 0.003652 | 0.385519 | -0.13834 | 0.292347 | -0.51812 | FAM161B |
| ILMN_1803291 | 0.012753 | 0.001555 | 0.164636 | 0.107851 | -0.24752 | 0.52839 | OR4C45 |
| ILMN_1788251 | 0.037074 | 0.007555 | 0.243289 | 0.018998 | -0.19363 | -0.05311 | SNN |
| ILMN_1666635 | 0.005919 | 3.97E-04 | 0.275849 | -0.09422 | -0.17153 | 0.079084 | RPS14 |
| ILMN_1791575 | 0.031916 | 0.00615 | -0.54847 | -0.56199 | 0.147249 | -0.9746 | HNRPLL |
| ILMN_3244319 | 0.004191 | 1.79E-04 | -0.33548 | 0.191539 | 0.23401 | -0.63697 | CCDC125 |
| ILMN_1776519 | 0.028601 | 0.005229 | 0.35292 | 0.036098 | -0.23061 | -0.28397 | RAP1GAP |
| ILMN_1667686 | 0.004377 | 2.00E-04 | 0.378038 | -0.05098 | -0.23773 | 0.216351 | EXOC6 |
| ILMN_1686156 | 0.009447 | 9.46E-04 | 0.378703 | 0.098871 | -0.24055 | 0.159842 | CUL4A |
| ILMN_3251370 | 0.004255 | 1.86E-04 | 0.672093 | 0.044815 | -0.34905 | 0.065187 | HOXA4 |
| ILMN_3238910 | 0.006266 | 4.45E-04 | 0.584178 | 0.09851 | -0.31201 | 0.032512 | LOC653545 |
| ILMN_1812893 | 0.006471 | 4.79E-04 | 0.537817 | 0.114723 | -0.31434 | 0.122199 | FOXK1 |
| ILMN_3247826 | 0.010939 | 0.001205 | -0.23137 | -0.405 | 0.145831 | 0.274747 | PYROXD1 |
| ILMN_1785060 | 0.033562 | 0.006584 | 0.380482 | -0.00458 | -0.15684 | -0.26934 | TSPAN14 |
| ILMN_1786882 | 0.016782 | 0.002365 | 0.210978 | 0.068275 | -0.53615 | 0.370348 | ERCC6 |
| ILMN_1727314 | 0.005246 | 3.11E-04 | 0.363857 | 0.044767 | -0.27093 | 0.152815 | MUC4 |
| ILMN_1792039 | 0.006268 | 4.46E-04 | 0.225944 | 0.070701 | -0.2456 | 0.191873 | MUC4 |
| ILMN_2366391 | 0.020608 | 0.003216 | 0.318305 | -0.10573 | -0.19611 | 0.047123 | PRDX1 |
| ILMN_1721672 | 0.031448 | 0.006015 | 0.133041 | 0.171524 | -0.21643 | 0.166156 | GPR171 |
| ILMN_1759312 | 0.017259 | 0.002463 | 0.423776 | 1.176321 | -0.32069 | 0.580921 | AMPD1 |
| ILMN_1670019 | 0.046549 | 0.010501 | 0.500745 | -0.25695 | 0.094635 | 0.117936 | PEG10 |
| ILMN_2387090 | 0.028836 | 0.005299 | 0.182142 | 7.53E-04 | -0.16477 | 0.133826 | CGGBP1 |
| ILMN_1668863 | 0.005465 | 3.40E-04 | 0.246183 | 0.132982 | -0.24428 | -0.06196 | LYPD1 |
| ILMN_1677633 | 0.009935 | 0.00102 | 0.295984 | 0.097743 | -0.21056 | 0.184603 | NFATC3 |
| ILMN_3239704 | 0.025498 | 0.004411 | 0.513099 | -0.16732 | -0.27361 | 0.140514 | SNORA11B |
| ILMN_3248915 | 0.005532 | 3.47E-04 | 0.072389 | 0.10292 | -0.2011 | 0.250677 | SUGT1L1 |
| ILMN_1730529 | 0.004447 | 2.06E-04 | 0.37548 | 0.013543 | -0.22892 | 0.212088 | CAB39L |
| ILMN_1761990 | 0.013565 | 0.001699 | 0.097351 | 0.07749 | -0.28348 | 0.127061 | RASGEF1A |
| ILMN_2363250 | 0.01947 | 0.002924 | -0.04573 | 0.374566 | -0.27983 | 0.280059 | BCL2 |
| ILMN_1701120 | 0.003279 | 8.25E-05 | 0.550248 | -0.00598 | -0.25849 | 0.167432 | BCL2 |
| ILMN_1815313 | 0.013712 | 0.001727 | 0.29839 | -0.18202 | -0.15663 | 0.143386 | EPGN |
| ILMN_3242362 | 0.00256 | 4.02E-05 | 0.286505 | -0.11535 | -0.362 | 0.093756 | LOC92973 |
| ILMN_1701613 | 0.022062 | 0.003555 | 0.473756 | -0.29397 | -0.14573 | 0.033205 | RARRES3 |
| ILMN_1770987 | 0.00935 | 9.27E-04 | 0.760138 | -0.12283 | -0.24834 | 0.274353 | SLC8A3 |
| ILMN_2094313 | 0.001835 | 1.78E-05 | 0.22009 | 0.129392 | -0.34893 | 0.102726 | ZDHHC1 |
| ILMN_1726164 | 0.004014 | 1.49E-04 | 0.309543 | -0.51024 | 0.05196 | 0.415761 | NEDD9 |
| ILMN_1756355 | 0.025829 | 0.004509 | 0.151478 | -0.09038 | -0.18054 | 0.147187 | NDUFS3 |
| ILMN_1745501 | 0.015798 | 0.002162 | 0.135004 | -0.09592 | -0.0501 | -0.51869 | DNALI1 |
| ILMN_1785141 | 0.003279 | 8.26E-05 | 0.517399 | -0.00984 | -0.26058 | 0.11205 | MICAL2 |
| ILMN_1676905 | 0.024661 | 0.004187 | -0.17416 | -0.03886 | 0.48632 | -0.79213 | TIGD7 |
| ILMN_2415911 | 0.007747 | 6.71E-04 | 0.316097 | 0.076174 | -0.27675 | 0.164369 | ENOX2 |
| ILMN_1651538 | 0.003834 | 1.32E-04 | 0.353808 | 0.1058 | -0.28456 | 0.1324 | NUMBL |
| ILMN_3241462 | 0.013904 | 0.00177 | 0.237975 | 0.016599 | -0.27693 | 0.101905 | LOC730754 |
| ILMN_2173919 | 0.040689 | 0.00863 | 0.552494 | -0.31384 | -0.0755 | -0.14763 | MYO9A |
| ILMN_1690209 | 0.036244 | 0.007342 | 0.07222 | 0.388071 | -0.29498 | -0.60843 | C1orf186 |
| ILMN_1733554 | 0.026654 | 0.004707 | -0.08452 | 0.867512 | -0.25071 | 0.346526 | UCP3 |
| ILMN_1755992 | 0.022845 | 0.003758 | 0.272887 | 0.176922 | -0.1146 | -0.62483 | UCP3 |
| ILMN_1803300 | 0.00554 | 3.48E-04 | 0.387957 | 0.022816 | -0.30942 | 0.535941 | C14orf68 |
| ILMN_1713542 | 0.042807 | 0.009279 | -0.86272 | -0.22605 | -0.09191 | 0.772841 | TRIM14 |
| ILMN_1805725 | 0.014189 | 0.001826 | 0.377212 | 0.06362 | -0.24216 | 0.132773 | B4GALNT1 |
| ILMN_1719985 | 0.020241 | 0.003125 | 0.239679 | -0.05752 | -0.18939 | 0.070704 | FEM1A |
| ILMN_1754541 | 0.004543 | 2.16E-04 | 0.427786 | 0.098969 | -0.32823 | 0.126733 | MYCL1 |
| ILMN_1811692 | 0.004079 | 1.63E-04 | 0.311892 | -0.04939 | -0.25768 | 0.049399 | FTSJ3 |
| ILMN_3249281 | 0.002163 | 2.72E-05 | 0.403386 | 0.273299 | -0.26817 | -0.25985 | HOXA11AS |
| ILMN_1785960 | 0.026029 | 0.004561 | 0.157089 | 0.338764 | -0.21816 | 0.106804 | ZBTB37 |
| ILMN_1678423 | 0.020207 | 0.003116 | 0.100469 | -0.12325 | 0.003054 | -0.69957 | SPA17 |
| ILMN_3236804 | 0.006493 | 4.85E-04 | -0.05607 | -0.73721 | -0.17151 | 0.586896 | SNORD126 |
| ILMN_1720088 | 0.015338 | 0.002079 | 0.146374 | 0.074837 | 0.060901 | -0.50016 | SFRS12 |
| ILMN_1744923 | 0.007806 | 6.79E-04 | 0.568146 | 0.052121 | -0.18363 | 0.221267 | WFDC5 |
| ILMN_1768035 | 0.004944 | 2.60E-04 | 0.265208 | 0.004928 | -0.25866 | 0.109556 | MMP12 |
| ILMN_1743034 | 0.004191 | 1.79E-04 | 0.41416 | 0.273023 | -0.2311 | 0.011532 | KIF1B |
| ILMN_2157219 | 0.022596 | 0.003698 | 0.215199 | -0.01053 | -0.32181 | 0.010988 | AASS |
| ILMN_1801851 | 0.00673 | 5.26E-04 | 0.999219 | 0.010072 | -0.23486 | 0.184085 | ALPPL2 |
| ILMN_1767176 | 0.046483 | 0.010476 | 0.586823 | 0.112163 | -0.17953 | 0.158078 | PDE9A |
| ILMN_2191436 | 0.001835 | 1.74E-05 | 0.373072 | -0.00165 | -0.22884 | 0.026971 | POLA1 |
| ILMN_2134381 | 0.005248 | 3.11E-04 | -0.35276 | -0.07814 | 0.399054 | -0.44788 | C14orf85 |
| ILMN_1738578 | 0.001835 | 1.72E-05 | 0.221751 | -0.36932 | -0.14471 | 0.965133 | FILIP1L |
| ILMN_2335072 | 0.004626 | 2.25E-04 | -0.41145 | -0.00289 | -0.0076 | -1.02724 | TAF1C |
| ILMN_2257833 | 0.002908 | 5.55E-05 | 0.35714 | 0.138645 | -0.22399 | 0.123214 | BBS7 |
| ILMN_1786377 | 0.032317 | 0.006247 | 0.31566 | 0.061619 | -0.16017 | 0.023369 | NIPBL |
| ILMN_2388605 | 0.006077 | 4.18E-04 | 0.241198 | 0.093422 | -0.30057 | -0.08553 | ACTR2 |
| ILMN_1681543 | 0.044694 | 0.009893 | 0.163486 | -0.6507 | -1.41E-05 | 0.228097 | RHBDD1 |
| ILMN_2309449 | 0.014248 | 0.001837 | -0.21158 | -0.31912 | 0.114217 | 0.193824 | RBBP6 |
| ILMN_1665135 | 0.024321 | 0.004103 | 0.327305 | 0.121358 | -0.12296 | 0.030921 | RBBP6 |
| ILMN_1724709 | 0.003631 | 1.14E-04 | 0.448605 | 0.042391 | -0.28665 | 0.104781 | SIGLEC12 |
| ILMN_2098433 | 0.011467 | 0.001297 | 0.630906 | 0.180854 | -0.21667 | 0.027859 | FAM10A4 |
| ILMN_1653719 | 0.003853 | 1.34E-04 | 0.582233 | 0.058301 | -0.28772 | -0.14135 | ITGBL1 |
| ILMN_1746968 | 0.001283 | 8.12E-06 | 0.20116 | -0.02388 | -0.29979 | 0.433825 | PHF1 |
| ILMN_1705617 | 0.019447 | 0.002919 | 0.128248 | -0.02619 | -0.20794 | 0.02896 | CFL1 |
| ILMN_1736178 | 0.043487 | 0.009519 | 0.033292 | 0.053685 | 0.032992 | -1.02864 | AEBP1 |
| ILMN_3234762 | 0.010118 | 0.001056 | 0.394536 | 0.118884 | -0.25058 | 0.102469 | RN5S9 |
| ILMN_1792986 | 0.004851 | 2.46E-04 | 0.370345 | 0.031531 | -0.20223 | 0.182364 | RFC1 |
| ILMN_1792671 | 0.012505 | 0.001498 | 0.002808 | -0.29724 | 0.229983 | -0.66473 | C12orf29 |
| ILMN_3249235 | 0.049955 | 0.011605 | -0.03215 | 0.110647 | 0.110966 | -0.45636 | GIN1 |
| ILMN_1769390 | 0.02483 | 0.00423 | -0.198 | 0.086782 | 0.053914 | 0.457276 | ABHD15 |
| ILMN_2383693 | 0.007806 | 6.78E-04 | 0.357614 | 0.111572 | -0.12918 | 0.082344 | UPF2 |
| ILMN_1716247 | 0.032356 | 0.006261 | 0.271401 | 0.176159 | -0.26444 | 0.056703 | FIBIN |
| ILMN_2133100 | 0.008721 | 8.21E-04 | 0.385975 | 0.062416 | -0.2848 | 0.084535 | LPIN2 |
| ILMN_1725969 | 0.004255 | 1.87E-04 | 0.546545 | 0.053284 | -0.29838 | 0.156832 | RPP14 |
| ILMN_1717855 | 0.034693 | 0.006909 | 0.478029 | -0.15048 | -0.10276 | 0.25158 | PFDN1 |
| ILMN_1691119 | 0.006016 | 4.11E-04 | 0.355907 | 0.097087 | -0.18391 | 0.109033 | RNF122 |
| ILMN_1680300 | 0.0123 | 0.001458 | 0.090093 | 5.56E-04 | -0.23743 | 0.154543 | BCAN |
| ILMN_1719864 | 0.038499 | 0.007951 | -0.89615 | -0.0124 | -0.03691 | 0.175046 | PACS2 |
| ILMN_2057399 | 0.001612 | 1.38E-05 | 0.368254 | 0.010921 | -0.14905 | -0.01962 | ZBTB8OS |
| ILMN_1706426 | 0.022977 | 0.003797 | 0.057274 | -0.21038 | 0.329309 | -0.48481 | DSTN |
| ILMN_1664316 | 0.0016 | 1.36E-05 | 0.48481 | 0.174122 | -0.27573 | 0.235145 | CCR9 |
| ILMN_2113606 | 0.005129 | 2.89E-04 | 0.363454 | -0.01502 | -0.20281 | 0.144604 | CCL27 |
| ILMN_1680757 | 0.019855 | 0.003015 | 0.355863 | 0.750003 | -0.37018 | 1.07333 | LRRC26 |
| ILMN_1713605 | 0.045714 | 0.010225 | 0.165651 | -0.04568 | -0.23408 | -0.00942 | RPAP1 |
| ILMN_1680320 | 0.041182 | 0.008801 | 0.339055 | 0.420681 | -0.28313 | 0.16412 | GBX2 |
| ILMN_1808356 | 0.017814 | 0.002581 | 0.24967 | -0.02255 | -0.25759 | 0.027483 | FAM3A |
| ILMN_1806415 | 0.021502 | 0.003413 | -0.02065 | -0.43978 | 0.155363 | -0.92065 | TTLL1 |
| ILMN_1719543 | 0.008704 | 8.17E-04 | 0.13261 | -0.29142 | -0.38434 | 0.460602 | MAF |
| ILMN_1674967 | 0.046684 | 0.010543 | -0.30741 | -0.16585 | 0.006924 | -1.16902 | TSGA10 |
| ILMN_2047240 | 0.038897 | 0.008075 | 0.180703 | -0.02205 | -0.25679 | -0.06178 | ARSA |
| ILMN_1660749 | 0.015119 | 0.002019 | 0.173452 | 0.021751 | -0.35754 | -0.01735 | ASPSCR1 |
| ILMN_1739155 | 0.00616 | 4.26E-04 | 0.256931 | 0.095707 | -0.25172 | 0.225725 | OR9A4 |
| ILMN_1682699 | 0.010306 | 0.001086 | 0.190352 | 0.061227 | -0.27628 | -0.24746 | PBX2 |
| ILMN_1729058 | 0.034357 | 0.006818 | -0.19373 | -0.40769 | 0.411397 | 0.0278 | SCAMP1 |
| ILMN_1770719 | 0.005404 | 3.30E-04 | -0.14268 | -0.94927 | 0.337462 | -0.77753 | KIAA0664 |
| ILMN_1757742 | 0.002863 | 5.42E-05 | 0.39136 | -0.08541 | -0.1213 | -0.01133 | ZRANB1 |
| ILMN_2312296 | 0.022762 | 0.003737 | 0.184301 | 0.026777 | -0.18919 | 0.029105 | PCBP2 |
| ILMN_1692617 | 0.020876 | 0.003273 | 0.334549 | -0.01628 | -0.13766 | 0.136727 | C4orf35 |
| ILMN_1665033 | 0.019614 | 0.002954 | -0.12105 | -0.47945 | 0.200243 | -0.3115 | NPR3 |
| ILMN_2386053 | 0.024628 | 0.004172 | 0.164861 | 0.313745 | 0.506664 | -0.5037 | DACH1 |
| ILMN_1705035 | 0.003763 | 1.25E-04 | 0.455314 | 0.126916 | -0.19264 | 0.13631 | FBXL7 |
| ILMN_2070043 | 1.28E-04 | 1.70E-07 | -0.16577 | 0.048039 | 0.364431 | -0.97849 | PPM1K |
| ILMN_1745533 | 0.002502 | 3.71E-05 | 0.246169 | -0.02115 | -0.37848 | 0.494385 | FAM117A |
| ILMN_1782890 | 0.027303 | 0.004872 | 0.334898 | -0.1049 | -0.01942 | 0.044314 | SLC25A3 |
| ILMN_1710863 | 0.028695 | 0.005264 | 0.173221 | 0.102954 | -0.13354 | -0.27737 | GATAD1 |
| ILMN_1768339 | 0.002761 | 4.79E-05 | 0.284597 | 0.220801 | -0.33618 | 0.076269 | C10orf31 |
| ILMN_1689217 | 0.005856 | 3.90E-04 | 0.399327 | -0.08891 | -0.2512 | 0.113512 | ACVR1C |
| ILMN_2172221 | 0.028652 | 0.005247 | 0.121132 | -0.01219 | -0.24581 | 0.07464 | SUMO1P3 |
| ILMN_1731503 | 0.004919 | 2.55E-04 | 1.116437 | 0.093155 | 0.037471 | 1.002857 | MARCO |
| ILMN_1657619 | 0.003027 | 6.20E-05 | -0.58019 | 0.205259 | 0.095217 | -0.33948 | DNAJB14 |
| ILMN_1767103 | 0.031041 | 0.005901 | 0.46633 | 0.141229 | -0.19987 | -0.16063 | C11orf63 |
| ILMN_2243516 | 1.69E-04 | 2.92E-07 | -0.49421 | 0.044246 | 0.261068 | -1.02673 | C11orf63 |
| ILMN_2120103 | 0.042387 | 0.009158 | 0.106918 | 0.082617 | -0.31186 | -0.10346 | AGAP7 |
| ILMN_2386354 | 0.002161 | 2.70E-05 | 0.49248 | 0.01554 | -0.12825 | -0.11001 | CSNK2A1 |
| ILMN_1804494 | 0.025257 | 0.004353 | -0.01597 | -0.21515 | -0.36981 | 0.582956 | KLHL25 |
| ILMN_1652790 | 0.013017 | 0.001602 | 0.151182 | 0.298085 | 0.00114 | -0.33847 | CLK1 |
| ILMN_2358069 | 0.021293 | 0.003361 | 0.997417 | 0.504943 | 0.110071 | 1.068651 | MAD1L1 |
| ILMN_2372136 | 0.004255 | 1.87E-04 | 0.251579 | -0.03191 | -0.25115 | 0.056929 | P4HTM |
| ILMN_1739813 | 0.048724 | 0.011229 | 0.219022 | 0.004539 | -0.25937 | 0.00693 | HYAL1 |
| ILMN_1704598 | 0.016511 | 0.0023 | 0.503934 | 0.001363 | -0.23288 | 0.25227 | HYAL4 |
| ILMN_1717852 | 0.008689 | 8.13E-04 | 0.241882 | 0.334409 | -0.39885 | 0.084032 | USH1G |
| ILMN_1666552 | 0.022855 | 0.003764 | 0.405871 | 0.137032 | -0.1249 | 0.105314 | ZNF75A |
| ILMN_1806488 | 0.005182 | 2.99E-04 | 0.148458 | -0.1118 | -0.32811 | 0.062401 | CCDC108 |
| ILMN_2100209 | 4.69E-04 | 1.33E-06 | 0.690098 | 0.240112 | -0.48081 | 1.110741 | CCL4L1 |
| ILMN_1696692 | 0.006077 | 4.18E-04 | 0.307534 | 0.155703 | -0.27022 | 0.172395 | BAZ1B |
| ILMN_1680831 | 0.002586 | 4.13E-05 | -0.6733 | 0.222081 | 0.400573 | -0.0252 | BAZ1B |
| ILMN_1776516 | 0.039042 | 0.00813 | 0.259086 | 0.202809 | -0.3791 | 0.460452 | ITPKA |
| ILMN_1651769 | 0.030019 | 0.005626 | -0.29144 | -0.20681 | 0.105695 | -0.35163 | ZFYVE16 |
| ILMN_1702541 | 0.006404 | 4.69E-04 | 0.309243 | -0.04796 | -0.20541 | -0.08147 | CCDC55 |
| ILMN_1672302 | 0.003777 | 1.26E-04 | 0.635468 | 0.047305 | -0.13577 | 0.22193 | STK32C |
| ILMN_2151304 | 0.001527 | 1.28E-05 | 0.418336 | 0.038809 | -0.28489 | 0.195427 | KERA |
| ILMN_1814465 | 0.015236 | 0.002052 | 0.282638 | 0.01022 | -0.29611 | 0.006365 | UBE2G1 |
| ILMN_2129927 | 0.011023 | 0.001224 | 0.056655 | -0.90967 | -0.0645 | 0.464633 | EXT1 |
| ILMN_1709439 | 2.71E-04 | 5.88E-07 | -0.08938 | 0.093901 | 0.112881 | -1.04929 | CHMP1A |
| ILMN_2266214 | 0.031188 | 0.005944 | -0.04769 | 0.114556 | -0.21694 | 0.162982 | LGALS8 |
| ILMN_1678729 | 0.00898 | 8.73E-04 | 0.242553 | 0.009863 | -0.23096 | 0.118481 | SIL1 |
| ILMN_1672415 | 0.006266 | 4.45E-04 | 0.513237 | 0.026731 | -0.19929 | 0.125555 | FBXO3 |
| ILMN_3244803 | 0.018682 | 0.002764 | 0.256484 | 0.134319 | -0.2046 | -0.09486 | RAD54L2 |
| ILMN_1778134 | 0.040659 | 0.008618 | 0.230306 | 0.033489 | -0.2207 | -0.1438 | TBRG1 |
| ILMN_1732182 | 0.004386 | 2.02E-04 | -0.66595 | 0.04825 | 0.610913 | -0.90273 | FBXO44 |
| ILMN_1769013 | 0.026726 | 0.004723 | 0.632178 | 0.143519 | -0.1973 | 0.002071 | ASGR1 |
| ILMN_1679301 | 0.048251 | 0.01107 | 0.180703 | -0.32718 | -0.1274 | 0.835448 | ZFAT |
| ILMN_1800002 | 0.00699 | 5.61E-04 | 0.016539 | 0.067687 | -0.22437 | 0.11884 | SRCRB4D |
| ILMN_3240740 | 0.006677 | 5.17E-04 | 0.354339 | 0.010623 | -0.1731 | -0.0165 | EIF3L |
| ILMN_1762725 | 0.012241 | 0.001447 | 0.296848 | 0.007557 | -0.13788 | -0.14973 | EIF3L |
| ILMN_1739144 | 0.004194 | 1.81E-04 | 0.239554 | 0.133742 | -0.15974 | 0.055453 | SI |
| ILMN_2364062 | 0.020358 | 0.003151 | 0.249422 | -0.15845 | -0.23078 | 0.276368 | THOC4 |
| ILMN_1784578 | 0.032051 | 0.006182 | -0.47024 | -0.04373 | 0.19901 | -0.28631 | LRRC37A3 |
| ILMN_3240226 | 0.0081 | 7.24E-04 | 0.2448 | 0.124706 | -0.28328 | -0.0734 | LOC791120 |
| ILMN_1794767 | 0.00382 | 1.30E-04 | 0.412413 | -0.06229 | -0.26554 | 0.196783 | OR52N5 |
| ILMN_1724266 | 9.67E-04 | 4.67E-06 | 0.620218 | -0.02274 | -0.24644 | 0.139976 | LYPD2 |
| ILMN_1768279 | 0.046505 | 0.010485 | 0.382908 | -0.11152 | -0.37642 | 0.155252 | NME6 |
| ILMN_2206413 | 0.016564 | 0.002318 | 0.346467 | -0.13426 | -0.12425 | 0.197402 | CTAG1A |
| ILMN_1699472 | 0.03621 | 0.007331 | 0.349284 | -0.32672 | -0.22833 | 0.381544 | ST3GAL3 |
| ILMN_1754815 | 0.004034 | 1.53E-04 | 0.320631 | -0.01931 | -0.2995 | 0.167731 | LHX5 |
| ILMN_1693669 | 0.010965 | 0.001211 | 0.096381 | 0.156949 | -0.2796 | -0.25582 | WDR79 |
| ILMN_1741180 | 0.004851 | 2.45E-04 | 0.217812 | 0.139036 | -0.26226 | -0.32008 | HEXDC |
| ILMN_1766405 | 0.004034 | 1.52E-04 | -0.0891 | -0.25594 | 0.389067 | -0.49342 | GOLM1 |
| ILMN_1769191 | 0.004357 | 1.98E-04 | 0.310259 | -0.01908 | -0.1987 | 0.100196 | GNAS |
| ILMN_1800951 | 0.009932 | 0.001019 | 0.269487 | -0.01666 | -0.21612 | 0.078225 | ATXN1 |
| ILMN_1778358 | 0.041372 | 0.008855 | 0.227873 | 0.315093 | -0.58884 | 0.500979 | ATP8A1 |
| ILMN_2322806 | 0.01259 | 0.001519 | 0.266917 | 0.096078 | -0.09274 | 0.175412 | CAST |
| ILMN_1775501 | 0.038372 | 0.007916 | 0.160237 | 0.060926 | -0.25829 | 1.443387 | IL1B |
| ILMN_1668333 | 0.016564 | 0.002316 | 0.400085 | 0.290316 | -0.27885 | 0.294264 | TMEM148 |
| ILMN_1726281 | 0.035873 | 0.007228 | 0.932343 | 1.143695 | -0.12266 | 1.365368 | ANAPC11 |
| ILMN_2216918 | 0.020608 | 0.003213 | 0.251093 | 0.046208 | -0.20441 | 0.20743 | SHPK |
| ILMN_1797577 | 0.003399 | 9.24E-05 | 0.542604 | -0.05119 | -0.24129 | 0.244418 | C2orf73 |
| ILMN_3307926 | 0.005122 | 2.88E-04 | 0.368232 | 0.16666 | -0.21884 | -0.02171 | ADRBK1 |
| ILMN_1660547 | 0.008417 | 7.71E-04 | 0.223097 | -0.05238 | -0.3784 | -0.06544 | ERC1 |
| ILMN_2331890 | 0.015146 | 0.002028 | 0.278674 | 0.069765 | -0.2512 | 0.066163 | RPL41 |
| ILMN_3235597 | 0.003277 | 7.77E-05 | 0.45319 | 0.131451 | -0.26174 | 0.002081 | LOC147727 |
| ILMN_1809857 | 0.008822 | 8.42E-04 | 0.508487 | 0.049164 | -0.24783 | 0.119862 | ZRANB3 |
| ILMN_1796085 | 0.03683 | 0.007495 | 0.109799 | -0.10329 | -0.09875 | 0.167104 | PPM1B |
| ILMN_1768077 | 0.006404 | 4.67E-04 | 0.15131 | 0.203262 | 0.171393 | -1.06123 | C10orf61 |
| ILMN_1744239 | 0.007878 | 6.88E-04 | 0.24244 | -0.16804 | -0.19524 | 0.227613 | FEM1B |
| ILMN_1665691 | 0.032234 | 0.006223 | 0.708211 | 0.287489 | -0.12234 | 0.280718 | ACTN3 |
| ILMN_1677851 | 0.027035 | 0.00479 | 0.47952 | -0.0065 | -0.37911 | -0.13901 | RARRES1 |
| ILMN_1683576 | 0.029913 | 0.005599 | 0.48178 | -0.56892 | -0.28511 | 0.12947 | MAGED2 |
| ILMN_1661417 | 0.006173 | 4.30E-04 | 0.248592 | -0.01108 | -0.33687 | 0.068382 | MAGED2 |
| ILMN_1790784 | 0.004646 | 2.28E-04 | 0.421237 | 0.008515 | -0.20924 | 0.05504 | ZNF197 |
| ILMN_1744217 | 0.004255 | 1.88E-04 | -0.03818 | 0.010137 | -0.16003 | 0.062412 | HTR7 |
| ILMN_2358541 | 0.018941 | 0.002816 | -0.3908 | -0.03902 | 0.358558 | -0.01633 | RBMS1 |
| ILMN_2382271 | 0.010581 | 0.001138 | 0.211862 | 0.022058 | -0.3105 | 0.06789 | NCKIPSD |
| ILMN_2287941 | 0.001199 | 7.02E-06 | 0.329073 | -0.03786 | -0.32769 | 0.15282 | CYLD |
| ILMN_1811347 | 0.006697 | 5.20E-04 | 0.325041 | 0.036374 | -0.18958 | 0.090415 | LDB1 |
| ILMN_1805039 | 0.023336 | 0.003884 | 0.575992 | 0.044451 | -0.16151 | 0.265611 | DPP10 |
| ILMN_3240957 | 0.036626 | 0.007445 | 0.011349 | -0.05459 | -0.27736 | 0.019872 | C2orf80 |
| ILMN_1705637 | 0.02762 | 0.004953 | -0.85597 | 0.555493 | -0.17807 | 0.846846 | ACR |
| ILMN_1732071 | 0.024892 | 0.004251 | 0.22574 | -0.00854 | -0.20325 | 0.096789 | HIST2H2BE |
| ILMN_2050654 | 0.013839 | 0.001753 | 0.378052 | -0.06039 | -0.1236 | -0.3585 | SAV1 |
| ILMN_1664448 | 0.007122 | 5.78E-04 | 0.196316 | -0.15458 | -0.38905 | 0.020891 | PEX12 |
| ILMN_1672178 | 0.005237 | 3.08E-04 | 0.366554 | 0.107685 | -0.29288 | 0.028503 | RNF8 |
| ILMN_1797332 | 0.04829 | 0.011084 | 0.369007 | -0.14563 | -0.0018 | -0.31335 | NARS2 |
| ILMN_1717714 | 0.003279 | 8.29E-05 | 0.185431 | 0.027681 | -0.30655 | 0.237253 | CDKN2A |
| ILMN_2367020 | 0.013904 | 0.001768 | 0.326792 | -0.31217 | -0.07522 | -0.01636 | SEC61G |
| ILMN_3238941 | 0.007077 | 5.73E-04 | 0.374424 | 0.042773 | -0.24609 | 0.189091 | LOC728613 |
| ILMN_2171783 | 0.044066 | 0.00971 | 0.292612 | 0.012325 | -0.25263 | 0.046541 | CPEB3 |
| ILMN_1776146 | 0.045793 | 0.010249 | 0.742539 | 0.131899 | -0.14072 | 0.378088 | RTP2 |
| ILMN_1673175 | 0.008853 | 8.48E-04 | 0.199616 | 0.129057 | -0.20817 | 0.260547 | TNFSF11 |
| ILMN_2276598 | 0.005266 | 3.15E-04 | 0.523861 | 0.278904 | -0.32099 | 0.150955 | AP1G2 |
| ILMN_2295879 | 0.013172 | 0.001626 | -0.05614 | 0.408878 | -0.39356 | 0.391989 | NGDN |
| ILMN_1788961 | 0.031433 | 0.006011 | 0.201195 | -0.00316 | -0.08578 | -0.06472 | PPP2R2A |
| ILMN_1729832 | 0.03912 | 0.008149 | 0.105427 | -0.1905 | -0.20679 | 0.166167 | LOC653994 |
| ILMN_1702858 | 0.021886 | 0.003505 | 0.153442 | 0.04647 | -0.23293 | 0.107956 | ADHFE1 |
| ILMN_1723871 | 0.031585 | 0.006055 | -0.80943 | -1.09762 | 0.076126 | -0.22141 | OTUB1 |
| ILMN_2126423 | 0.018364 | 0.002703 | 0.18543 | -0.06318 | 0.140446 | -0.62485 | ZNF480 |
| ILMN_1806432 | 0.003584 | 1.08E-04 | 0.193522 | 0.08967 | -0.30966 | 0.096917 | NT5C |
| ILMN_2081087 | 0.005629 | 3.61E-04 | 0.267447 | -0.00916 | -0.24577 | 0.043351 | HSPA12A |
| ILMN_1743770 | 0.005354 | 3.23E-04 | 0.14676 | 0.099229 | -0.21315 | 0.043683 | SLC25A14 |
| ILMN_1800420 | 0.013166 | 0.001625 | 0.319798 | 0.223183 | -0.22307 | 0.099866 | RNF214 |
| ILMN_1737426 | 0.004063 | 1.56E-04 | 0.347118 | -0.01291 | -0.12907 | 0.040571 | PCMTD1 |
| ILMN_2046751 | 0.035386 | 0.007108 | 1.146665 | 0.090276 | -0.08959 | 0.190311 | PGLYRP3 |
| ILMN_2046750 | 0.044728 | 0.009909 | 0.45628 | -0.08274 | -0.22609 | 0.053058 | PGLYRP3 |
| ILMN_2322986 | 0.003279 | 8.31E-05 | 0.388797 | 0.036309 | -0.27881 | 0.482772 | MINA |
| ILMN_2177090 | 0.001337 | 8.93E-06 | 0.046872 | 0.257135 | -0.10494 | -0.84272 | LOC200030 |
| ILMN_1701562 | 0.010739 | 0.001164 | -0.60059 | 0.06727 | 0.197416 | 9.64E-04 | PKHD1 |
| ILMN_2407879 | 0.007348 | 6.13E-04 | -0.25936 | -0.28986 | 0.158385 | -0.76917 | SORBS2 |
| ILMN_1693183 | 0.015264 | 0.00206 | -0.25796 | 0.601241 | 0.086688 | 0.27765 | SORBS2 |
| ILMN_1704424 | 0.004789 | 2.41E-04 | 0.13179 | -0.64818 | 0.283498 | 0.395483 | PLA2R1 |
| ILMN_3240316 | 0.006782 | 5.34E-04 | 0.019972 | 0.260236 | -0.09453 | -0.42078 | TMSL3 |
| ILMN_1702715 | 0.0038 | 1.28E-04 | 0.218803 | -0.02943 | -0.38105 | 0.176509 | MECP2 |
| ILMN_1686485 | 0.004971 | 2.63E-04 | 0.55017 | -0.00831 | -0.22837 | 0.14679 | DDX59 |
| ILMN_2327812 | 0.009843 | 0.001006 | 0.371283 | 0.110748 | -0.09889 | 0.773956 | IL5RA |
| ILMN_2324909 | 0.036626 | 0.007444 | 0.400927 | -0.21112 | -0.3144 | 0.42755 | PARD3B |
| ILMN_1673892 | 0.005128 | 2.89E-04 | 0.094162 | 0.217426 | -0.26288 | 0.08456 | GK5 |
| ILMN_1731984 | 0.03884 | 0.008049 | -0.18994 | 0.13572 | 0.202298 | -0.25505 | HMGN3 |
| ILMN_1704477 | 0.031188 | 0.005942 | -0.33619 | -0.18096 | 0.20072 | -0.70593 | COX5A |
| ILMN_1768293 | 0.017881 | 0.002597 | -0.40463 | -0.06324 | 0.388391 | -1.01607 | NUP155 |
| ILMN_1731546 | 0.001465 | 1.15E-05 | 0.239599 | -0.13782 | -0.15163 | 0.276121 | RPL26 |
| ILMN_1724424 | 0.00523 | 3.07E-04 | 0.223783 | 0.092795 | -0.36805 | -0.03122 | PRRT2 |
| ILMN_3189715 | 0.00749 | 6.33E-04 | 0.27713 | 0.060654 | -0.28398 | 0.16162 | HDAC7 |
| ILMN_1760400 | 0.012753 | 0.001555 | 0.357189 | -0.29628 | 0.118152 | -0.21897 | C8orf41 |
| ILMN_2227248 | 0.006643 | 5.06E-04 | 0.388755 | 0.082742 | -0.2049 | 0.159595 | SLAMF9 |
| ILMN_2224946 | 0.017076 | 0.002423 | 0.171117 | 0.157306 | -0.32037 | 0.256581 | ZNF354C |
| ILMN_2145781 | 0.006404 | 4.70E-04 | 0.37381 | 0.067334 | -0.19292 | 0.063052 | RBMY2FP |
| ILMN_1730765 | 0.01768 | 0.002554 | 0.363115 | 0.03067 | -0.25665 | 0.008944 | DUSP22 |
| ILMN_3307950 | 0.00353 | 1.02E-04 | 0.144587 | -0.13346 | 0.045088 | -0.81721 | CDK5RAP3 |
| ILMN_2168228 | 0.004034 | 1.52E-04 | 0.279986 | -0.05422 | -0.3209 | 0.081914 | ATOH7 |
| ILMN_1784217 | 0.047595 | 0.010854 | -0.33108 | -0.41316 | 0.199546 | -0.58597 | SOX15 |
| ILMN_1702127 | 0.00749 | 6.33E-04 | 0.503454 | 0.020434 | -0.2654 | 0.289382 | SPRR2G |
| ILMN_1808305 | 0.042146 | 0.009094 | -0.36626 | -0.20209 | 0.109391 | -0.18586 | RTCD1 |
| ILMN_2216582 | 0.009372 | 9.30E-04 | 0.0897 | 0.311465 | -0.34186 | -0.31559 | LYL1 |
| ILMN_3307854 | 0.009812 | 0.001001 | 0.367849 | 0.048558 | -0.23323 | 0.129685 | PART1 |
| ILMN_1802905 | 0.012378 | 0.001472 | 0.256924 | 0.051185 | -0.25451 | 0.014221 | PIAS4 |
| ILMN_1788196 | 0.007543 | 6.40E-04 | 0.132362 | -0.08125 | -0.30106 | 0.059939 | MSL3 |
| ILMN_1787705 | 0.005158 | 2.96E-04 | 0.255652 | -0.22867 | -0.16005 | 0.110462 | ATP6V1B2 |
| ILMN_2277523 | 0.012518 | 0.001501 | 0.236974 | 0.056573 | -0.22961 | -0.3724 | DIP2A |
| ILMN_2148360 | 0.016949 | 0.002396 | -0.157 | -0.26754 | 0.295066 | -0.52385 | ADAM10 |
| ILMN_2339955 | 0.001283 | 8.24E-06 | 0.413324 | 0.770426 | -0.35525 | 1.203106 | NR4A2 |
| ILMN_1782305 | 0.02455 | 0.004149 | 0.456445 | 0.317048 | -0.06156 | 1.408584 | NR4A2 |
| ILMN_1726198 | 0.008661 | 8.08E-04 | 0.346204 | -0.15854 | -0.33294 | 0.198304 | CTSL3 |
| ILMN_1781457 | 0.021845 | 0.003496 | -0.45744 | -0.26422 | 0.098316 | -0.43063 | TIPRL |
| ILMN_2319414 | 0.001907 | 1.98E-05 | 0.277598 | 0.124824 | -0.26375 | 0.132237 | BTF3 |
| ILMN_2415748 | 0.049141 | 0.011372 | 0.186178 | 0.063148 | -0.21135 | -0.18292 | WSB1 |
| ILMN_2382964 | 0.004851 | 2.47E-04 | 0.365242 | 0.034772 | -0.29021 | 0.249762 | PRSS21 |
| ILMN_1700047 | 0.002908 | 5.57E-05 | 0.37138 | -0.12521 | -0.26198 | 0.141141 | ALAS1 |
| ILMN_1703913 | 0.009271 | 9.15E-04 | 0.300756 | -0.09375 | -0.25476 | 0.038158 | DST |
| ILMN_1685680 | 0.003279 | 8.08E-05 | 0.102321 | -0.0101 | 0.68215 | -0.5895 | SPATA1 |
| ILMN_1772882 | 0.00333 | 8.61E-05 | 0.29321 | 0.019937 | -0.33608 | 0.123149 | OR2T1 |
| ILMN_2388177 | 0.027898 | 0.005028 | 0.23625 | -0.30522 | -0.03663 | -0.79436 | SPATA7 |
| ILMN_2344956 | 0.048006 | 0.010991 | 0.120214 | -0.0752 | -0.23462 | 0.303483 | ACP1 |
| ILMN_3238931 | 0.008087 | 7.21E-04 | 0.351178 | 0.056337 | -0.21526 | 0.113943 | DEFB130 |
| ILMN_2232177 | 0.007413 | 6.22E-04 | 0.158806 | 0.053657 | -0.31029 | 0.040887 | ACTN1 |
| ILMN_1688953 | 0.010927 | 0.001201 | 1.088886 | 0.431575 | -0.09721 | 1.032451 | ARHGAP19 |
| ILMN_2278636 | 0.011494 | 0.001309 | 0.2129 | -0.0084 | -0.09078 | -0.04229 | CUTL1 |
| ILMN_1773546 | 0.011629 | 0.001341 | 0.224511 | -0.03197 | -0.25246 | 0.208841 | MRGPRX3 |
| ILMN_2408576 | 0.034625 | 0.006889 | 0.184194 | -0.45086 | -0.06602 | -1.24719 | FAM129B |
| ILMN_1735168 | 0.025154 | 0.004322 | 0.038284 | 0.103405 | -0.19117 | 0.105298 | SUV420H2 |
| ILMN_2378316 | 0.044047 | 0.009704 | -0.34305 | -0.01336 | -0.32311 | 1.155748 | NUPL1 |
| ILMN_2122014 | 0.004851 | 2.47E-04 | 0.136478 | 0.008079 | -0.18788 | 0.327598 | OR2B3P |
| ILMN_3307786 | 0.045141 | 0.010052 | 0.257911 | 0.09408 | -0.24864 | -0.12598 | L3MBTL2 |
| ILMN_1771058 | 0.01202 | 0.001409 | 0.322543 | 0.019913 | -0.26823 | 0.11299 | VAX1 |
| ILMN_3240354 | 0.019906 | 0.003035 | 0.315354 | 0.031594 | -0.23682 | 0.275812 | CYCSP52 |
| ILMN_2411282 | 0.010796 | 0.001177 | 0.331159 | 0.116098 | -0.15025 | -0.30446 | QSOX1 |
| ILMN_1772627 | 0.03054 | 0.005762 | 0.129332 | 0.81987 | -0.39611 | 0.383825 | D4S234E |
| ILMN_1660913 | 0.028811 | 0.005292 | 0.363395 | 0.161684 | -0.25935 | 0.154374 | LOC284296 |
| ILMN_3247592 | 0.006365 | 4.61E-04 | 0.319187 | 0.077965 | -0.23892 | 0.068418 | ZNF830 |
| ILMN_1738821 | 0.010118 | 0.001056 | 0.075849 | 0.107561 | -0.23871 | 0.4129 | GOLGA2 |
| ILMN_1680037 | 0.02423 | 0.004082 | 0.24549 | -0.00155 | -0.208 | 0.14262 | FAM65A |
| ILMN_1775348 | 0.004671 | 2.31E-04 | 0.659134 | 0.06868 | -0.21501 | 0.149341 | KCNH8 |
| ILMN_3307616 | 0.005688 | 3.68E-04 | 0.684016 | -0.03442 | -0.25012 | 0.060251 | EPHA10 |
| ILMN_1663263 | 0.005012 | 2.72E-04 | 0.407764 | -0.12506 | -0.2336 | 0.143764 | EPHA10 |
| ILMN_1715788 | 0.021845 | 0.003497 | 0.341039 | 0.118903 | -0.14113 | 0.160072 | MMRN2 |
| ILMN_1748823 | 0.018304 | 0.00269 | 0.484773 | -0.02711 | -0.29466 | 0.016035 | LOC374920 |
| ILMN_3242883 | 0.025859 | 0.004517 | 0.173226 | 0.09485 | -0.2237 | -0.30849 | AGAP8 |
| ILMN_1726693 | 0.043333 | 0.009462 | -0.40947 | 0.006312 | 0.244656 | -0.43925 | GTF2H1 |
| ILMN_1743677 | 0.023336 | 0.003879 | -0.02511 | 0.07199 | -0.11345 | 0.338329 | HNRNPU |
| ILMN_1771462 | 0.027311 | 0.00488 | 0.723672 | 0.622973 | -0.01434 | -0.00401 | RPL36 |
| ILMN_2225974 | 0.009812 | 0.001001 | -0.18145 | -0.03355 | 0.066366 | -0.67366 | GCLM |
| ILMN_2381397 | 0.022775 | 0.00374 | 0.337984 | -0.02901 | -0.1827 | -0.0879 | HSPD1 |
| ILMN_1667201 | 0.004922 | 2.56E-04 | 0.445484 | 0.046786 | -0.15292 | -0.01732 | WDR51B |
| ILMN_2331501 | 0.008795 | 8.36E-04 | 0.234115 | 0.03977 | -0.26033 | -0.06367 | UBC |
| ILMN_2186319 | 0.006662 | 5.13E-04 | 0.283277 | 0.147027 | -0.24223 | 0.033062 | TSPAN2 |
| ILMN_1680403 | 0.005774 | 3.81E-04 | -0.49079 | -0.09135 | 0.133507 | -0.45235 | SSR4 |
| ILMN_1703459 | 0.02585 | 0.004514 | 0.288734 | 0.075278 | -0.42592 | -0.19379 | WNT9B |
| ILMN_1664592 | 0.039414 | 0.008241 | 0.718349 | -0.34383 | -0.53548 | 0.947975 | C1orf95 |
| ILMN_1809957 | 0.030121 | 0.005649 | 0.217109 | 0.06285 | -0.31264 | -0.16009 | AP2S1 |
| ILMN_1740419 | 0.00256 | 3.95E-05 | 0.295632 | 0.064796 | -0.32404 | 0.168016 | CATSPER4 |
| ILMN_2396039 | 0.019764 | 0.002994 | -0.97169 | 0.223826 | -0.00722 | -0.15245 | HOXC6 |
| ILMN_1673886 | 0.02576 | 0.004481 | 0.353448 | -0.90365 | -0.05353 | -1.21137 | SIM2 |
| ILMN_1722858 | 0.003614 | 1.12E-04 | 0.353847 | -0.05183 | -0.24292 | 0.042156 | PPP2CA |
| ILMN_1680754 | 0.003571 | 1.06E-04 | 0.331816 | 0.083125 | -0.29589 | 0.129975 | A4GNT |
| ILMN_2325625 | 0.003279 | 8.24E-05 | 0.473074 | -0.07991 | -0.27925 | 0.245782 | FBXO44 |
| ILMN_1700203 | 0.00262 | 4.27E-05 | 0.285387 | 0.12377 | -0.52024 | 0.436641 | KIAA1984 |
| ILMN_2278550 | 0.01259 | 0.001519 | -0.05262 | -0.01522 | -0.42692 | 0.237216 | MBOAT1 |
| ILMN_3249095 | 0.00716 | 5.87E-04 | 0.216233 | 0.055547 | -0.20866 | 0.216611 | C22orf24 |
| ILMN_1736655 | 0.018041 | 0.002627 | 0.472292 | 0.138678 | -0.27107 | 0.250071 | LCE1B |
| ILMN_1815102 | 0.002449 | 3.57E-05 | 0.14652 | 0.153063 | -0.47078 | -0.17271 | LCAT |
| ILMN_1785268 | 0.014733 | 0.001939 | 0.043306 | -0.30714 | 0.136804 | -0.48573 | CD58 |
| ILMN_1734312 | 0.019931 | 0.003044 | 0.210227 | 0.108527 | -0.19317 | 0.018294 | GCN1L1 |
| ILMN_3251747 | 0.026207 | 0.004601 | 0.362807 | -0.07992 | -0.31619 | 0.036372 | UNCX |
| ILMN_2301624 | 0.043003 | 0.009344 | -0.16466 | -0.08909 | 0.087489 | -0.36517 | MACF1 |
| ILMN_1681116 | 0.02715 | 0.004821 | 0.354167 | -0.43552 | 0.196889 | 1.019433 | NPHS1 |
| ILMN_1773080 | 0.004709 | 2.34E-04 | 0.327555 | 0.01309 | -0.30569 | -0.02035 | OAZ1 |
| ILMN_2335813 | 0.003969 | 1.45E-04 | 0.544939 | -0.2829 | -0.27293 | 1.238764 | GCH1 |
| ILMN_2312275 | 0.011031 | 0.001226 | -0.0813 | 0.106917 | -0.13806 | 0.25947 | SRP54 |
| ILMN_2328666 | 0.008747 | 8.25E-04 | 0.599387 | -0.44662 | -0.15683 | 1.521714 | CD83 |
| ILMN_2382724 | 0.041025 | 0.008744 | 0.17312 | -0.06442 | -0.24088 | 0.039498 | C17orf95 |
| ILMN_1763137 | 0.006311 | 4.51E-04 | -0.80636 | 0.120888 | 0.144198 | 0.134604 | INVS |
| ILMN_1727218 | 0.00876 | 8.29E-04 | 0.273223 | 0.117468 | -0.24549 | 0.052013 | ZBTB6 |
| ILMN_1810836 | 0.017483 | 0.002514 | 0.233134 | 0.193461 | -0.15828 | -0.11815 | PDE5A |
| ILMN_2342695 | 0.044504 | 0.009835 | 0.352112 | 0.03087 | 0.497008 | -0.35222 | PDGFA |
| ILMN_3243190 | 0.022854 | 0.003761 | 0.730213 | 0.723753 | -0.21054 | 1.26598 | EMR4P |
| ILMN_3236373 | 0.00365 | 1.18E-04 | -0.2099 | 0.049782 | 0.128963 | -0.89128 | MSL2 |
| ILMN_3236594 | 0.0159 | 0.002183 | 0.347451 | -0.01145 | -0.20656 | -0.00566 | SNORA29 |
| ILMN_2075067 | 0.005009 | 2.71E-04 | 0.36748 | 0.151244 | -0.23157 | 0.255306 | DIO3 |
| ILMN_1754233 | 0.015146 | 0.002029 | 0.091064 | -0.21529 | -0.29609 | 0.058818 | ITGB1 |
| ILMN_1673936 | 0.048158 | 0.011042 | 0.210492 | 0.063181 | -0.2574 | -0.11074 | KHSRP |
| ILMN_1657347 | 0.048016 | 0.011001 | -0.71023 | -0.39647 | 0.170069 | -0.83353 | PODXL2 |
| ILMN_1709075 | 0.005042 | 2.75E-04 | 0.22356 | 0.016459 | -0.27543 | 0.087984 | OLA1 |
| ILMN_2178088 | 0.004255 | 1.89E-04 | 0.164905 | 0.340338 | -0.29876 | 0.132114 | ZNF131 |
| ILMN_2132515 | 0.007968 | 7.04E-04 | 0.369065 | 0.089944 | -0.16139 | 0.176892 | DEFB103A |
| ILMN_1702946 | 0.003853 | 1.34E-04 | -0.17369 | -0.05042 | 0.210641 | -0.27771 | THUMPD1 |
| ILMN_2338785 | 0.013217 | 0.001638 | 0.345016 | 0.081478 | -0.21523 | -0.01099 | RPS14 |
| ILMN_2165251 | 0.004882 | 2.51E-04 | 0.396627 | -0.21472 | -0.46051 | 0.693822 | RGPD6 |
| ILMN_1766200 | 0.024862 | 0.004238 | 0.313791 | 2.66E-04 | -0.13076 | -0.0479 | CALHM2 |
| ILMN_1704045 | 0.022921 | 0.003778 | 0.657152 | 0.195734 | -0.32126 | 0.621305 | DEAF1 |
| ILMN_1687888 | 0.001113 | 6.31E-06 | 0.406062 | -0.11408 | -0.31173 | 0.138035 | BTN3A1 |
| ILMN_1740294 | 0.015041 | 0.002001 | 0.148199 | -0.07393 | -0.42967 | -0.1527 | MAP1D |
| ILMN_1733932 | 0.046345 | 0.010431 | 0.286896 | -0.08768 | -0.37797 | -0.07661 | SNUPN |
| ILMN_2071429 | 0.007254 | 6.00E-04 | 0.376173 | 0.056677 | -0.25098 | 0.247718 | TTC39B |
| ILMN_1775073 | 0.033124 | 0.006464 | 0.204666 | 0.110254 | -0.21158 | 0.083881 | ZNF273 |
| ILMN_2346562 | 0.046874 | 0.010597 | -0.35932 | 0.257316 | 0.349289 | -0.2894 | ZNF273 |
| ILMN_1812567 | 0.008866 | 8.56E-04 | 0.342803 | -0.00307 | -0.22883 | 0.032809 | ICA1 |
| ILMN_1779917 | 0.004885 | 2.52E-04 | 0.446458 | -0.08947 | -0.25071 | 0.093817 | INPP5F |
| ILMN_1656040 | 0.004579 | 2.20E-04 | 0.32862 | -0.01643 | -0.31654 | 0.150289 | NTN3 |
| ILMN_1755115 | 0.00319 | 7.23E-05 | 0.201053 | -0.00445 | -0.28585 | -0.00393 | RPL23 |
| ILMN_1704675 | 0.013217 | 0.001639 | 0.331721 | 0.08503 | -0.31476 | 0.065611 | IGSF1 |
| ILMN_2414762 | 0.028397 | 0.005175 | -0.08156 | 0.332572 | -0.34229 | -0.04996 | TLR10 |
| ILMN_1673676 | 0.022671 | 0.003716 | 0.440478 | -0.0255 | -0.22676 | 0.206758 | SNX5 |
| ILMN_3246732 | 0.002424 | 3.49E-05 | 0.471102 | 0.05887 | -0.32571 | 0.120698 | C16orf88 |
| ILMN_2097793 | 0.016372 | 0.002273 | 0.23734 | 0.096894 | -0.21787 | -0.02327 | ZBTB4 |
| ILMN_1795905 | 0.003258 | 7.63E-05 | 0.348104 | 0.083572 | -0.17069 | -0.11792 | ZBTB4 |
| ILMN_1766222 | 0.019271 | 0.002887 | 0.33849 | -0.16519 | 0.179967 | -0.99659 | LARP4B |
| ILMN_1651336 | 0.007647 | 6.55E-04 | 0.352332 | -0.09028 | -0.3628 | 0.417124 | MLYCD |
| ILMN_1777024 | 0.015023 | 0.001994 | 0.729166 | 0.071542 | -0.18507 | 0.246282 | PCDHAC2 |
| ILMN_1709112 | 0.018587 | 0.002748 | 0.771084 | -0.8977 | -0.1178 | 0.413144 | CHM |
| ILMN_1702149 | 0.005447 | 3.38E-04 | 0.567721 | -0.06595 | -0.21399 | 0.210701 | CACNG4 |
| ILMN_1672114 | 0.006645 | 5.08E-04 | 0.116221 | 0.230255 | -0.35545 | 0.447953 | TNFRSF10C |
| ILMN_2368617 | 0.044665 | 0.009883 | -0.11313 | -0.53142 | 0.166073 | -0.86759 | FKRP |
| ILMN_1698968 | 0.036932 | 0.00752 | -0.29911 | -0.01352 | 0.149432 | -0.6453 | ASXL2 |
| ILMN_1769229 | 0.001031 | 5.16E-06 | 0.400083 | 0.036808 | -0.30858 | 0.165131 | BCL2A1 |
| ILMN_1681193 | 0.043064 | 0.009365 | 0.871527 | 0.053351 | -0.44572 | -0.48567 | EPS8L1 |
| ILMN_1718405 | 0.040293 | 0.008502 | 0.067946 | -0.03386 | 0.014128 | -0.57005 | RAPH1 |
| ILMN_1680732 | 0.018587 | 0.002748 | 0.391294 | 0.005978 | -0.13932 | 0.518033 | B4GALT2 |
| ILMN_2371379 | 0.013347 | 0.001659 | 0.303446 | 0.067156 | -0.19853 | 0.179766 | ACLY |
| ILMN_1761364 | 0.004063 | 1.57E-04 | 0.325954 | 5.86E-04 | -0.31825 | 0.111627 | NCR2 |
| ILMN_2322747 | 0.005305 | 3.18E-04 | 0.288605 | 0.212332 | -0.20662 | 0.063308 | ARHGAP5 |
| ILMN_1778691 | 0.040695 | 0.008635 | 0.030382 | 0.13254 | 0.104252 | -0.60247 | TIA1 |
| ILMN_2153837 | 0.030434 | 0.005741 | -0.254 | -0.04733 | 0.199168 | -0.47249 | SCNN1G |
| ILMN_2272251 | 0.005412 | 3.33E-04 | 0.500992 | 0.06603 | -0.27626 | 0.154082 | ASB11 |
| ILMN_2370624 | 0.038754 | 0.00802 | 0.318754 | 0.419421 | 0.204709 | -1.1248 | COL13A1 |
| ILMN_2364357 | 0.004627 | 2.25E-04 | 0.235546 | 0.023321 | -0.41359 | 0.075609 | RPS6KB2 |
| ILMN_2211122 | 0.039694 | 0.008325 | -0.12723 | 0.397786 | -0.35694 | 0.827908 | TRAPPC2P1 |
| ILMN_1773210 | 0.004789 | 2.41E-04 | 0.555234 | 0.185362 | -0.40408 | 0.088642 | DRD1 |
| ILMN_3247375 | 0.043837 | 0.009631 | 0.510521 | -0.27046 | -0.04428 | 0.176082 | KIAA0087 |
| ILMN_1705653 | 0.003485 | 9.89E-05 | 0.212716 | 0.116444 | -0.33588 | 0.001824 | BCAM |
| ILMN_1707548 | 0.028999 | 0.005343 | 0.403952 | 0.598962 | -0.22266 | 0.894761 | RAD18 |
| ILMN_1793829 | 0.007888 | 6.90E-04 | 0.240663 | -0.05956 | -0.20166 | 0.005361 | TMCO1 |
| ILMN_1813604 | 0.031525 | 0.006036 | 0.285928 | -0.09198 | -0.15176 | 0.119386 | NDUFB7 |
| ILMN_1680692 | 0.014291 | 0.001848 | 0.223974 | 0.259199 | -0.1134 | -0.60404 | NUCKS1 |
| ILMN_1699447 | 0.035851 | 0.007218 | -0.08816 | 0.824268 | -0.39416 | 0.178453 | TRAF5 |
| ILMN_1701331 | 0.003032 | 6.33E-05 | 0.301695 | -0.03942 | -0.40214 | 0.054858 | UBE2M |
| ILMN_1654357 | 0.018247 | 0.002677 | 0.245538 | -0.61684 | -0.12062 | 0.275514 | ZNF24 |
| ILMN_1769316 | 0.014546 | 0.0019 | 0.641761 | -0.01031 | -0.43491 | 0.158323 | UMODL1 |
| ILMN_1683302 | 0.005447 | 3.39E-04 | 0.395637 | 0.032839 | -0.22572 | 0.099451 | SMC4 |
| ILMN_1686948 | 0.020015 | 0.003067 | -0.00513 | 0.110864 | -0.19891 | 0.189153 | CASC4 |
| ILMN_1765550 | 0.007273 | 6.03E-04 | 0.302537 | -0.02416 | -0.24923 | 0.072732 | HOMEZ |
| ILMN_3244105 | 0.007136 | 5.80E-04 | 0.332999 | 0.122896 | -0.20883 | 0.291773 | C1orf204 |
| ILMN_1675788 | 0.028001 | 0.005056 | 0.148542 | 0.225669 | -0.07863 | -0.37015 | ZNF175 |
| ILMN_3238662 | 0.03416 | 0.006761 | 0.305095 | -0.04905 | -0.30244 | 0.020028 | SNORD89 |
| ILMN_1697761 | 0.004437 | 2.04E-04 | 0.405693 | -0.00639 | -0.32243 | 0.043391 | C3orf67 |
| ILMN_2160819 | 0.004543 | 2.17E-04 | 0.393953 | 0.081989 | -0.18139 | 0.034026 | RPS27 |
| ILMN_1677824 | 0.023126 | 0.003832 | -0.32895 | -0.32003 | 0.085966 | 0.679592 | RAB4A |
| ILMN_2200331 | 0.007543 | 6.41E-04 | 0.452729 | 0.113444 | -0.26015 | 0.461888 | H2AFX |
| ILMN_2095660 | 0.022373 | 0.003653 | -0.25125 | 1.117102 | -0.04743 | -0.35246 | TMEM156 |
| ILMN_2387865 | 0.037392 | 0.007645 | -0.38052 | -0.53774 | 0.214925 | -0.90104 | KCNIP4 |
| ILMN_2142935 | 0.040177 | 0.008464 | 0.264176 | 0.068009 | 7.67E-04 | -0.4424 | BLNK |
| ILMN_2069821 | 0.011496 | 0.001311 | 0.130561 | 0.025879 | -0.14954 | 0.23551 | C4orf32 |
| ILMN_2411116 | 0.005988 | 4.06E-04 | 0.341799 | -0.24525 | -0.31513 | 0.104465 | FGFR1OP |
| ILMN_1705433 | 0.010399 | 0.001104 | 0.300938 | 0.060818 | -0.21959 | 0.155383 | CBLL1 |
| ILMN_1763267 | 0.015146 | 0.002029 | 0.149555 | -0.44174 | 0.441822 | -0.1218 | ZFYVE28 |
| ILMN_1804851 | 0.02058 | 0.003204 | 0.35242 | -0.01431 | -0.07209 | -0.1691 | MRPS17 |
| ILMN_1714718 | 0.010269 | 0.00108 | 0.347495 | 0.124102 | -0.24092 | 0.249138 | CXorf59 |
| ILMN_1761833 | 0.017043 | 0.002414 | -0.42203 | -0.20313 | 0.245796 | -0.48391 | SLC40A1 |
| ILMN_1755822 | 0.012184 | 0.001435 | 0.177406 | -0.26182 | 0.212617 | -0.56232 | SYDE1 |
| ILMN_3246273 | 0.010504 | 0.001125 | 0.407176 | 0.102844 | -0.29776 | -0.05633 | RNU1-3 |
| ILMN_2194467 | 0.010847 | 0.001187 | 0.358353 | -0.0886 | -0.17627 | 0.068459 | SGCB |
| ILMN_1810324 | 0.006957 | 5.58E-04 | 0.29855 | -0.02901 | -0.24872 | -0.05976 | SGCB |
| ILMN_2403247 | 0.007279 | 6.06E-04 | 0.423733 | 0.064141 | -0.19443 | 0.126439 | CMTM7 |
| ILMN_1696810 | 0.005057 | 2.77E-04 | 0.41887 | 0.170822 | -0.25713 | 0.202347 | GPR182 |
| ILMN_1798189 | 0.006173 | 4.31E-04 | 0.315875 | -0.17431 | -0.09345 | 0.191498 | COX7C |
| ILMN_2093810 | 0.005773 | 3.81E-04 | 0.470279 | 0.153725 | -0.25724 | 0.275076 | POMP |
| ILMN_2267084 | 0.003571 | 1.05E-04 | 0.361459 | -0.07592 | -0.3317 | 0.061852 | ZFYVE1 |
| ILMN_1794392 | 0.003277 | 7.91E-05 | 0.054371 | -0.06667 | -0.15195 | 0.572347 | DDX3X |
| ILMN_1764297 | 0.033642 | 0.006613 | 0.309022 | 0.073702 | -0.18413 | 0.006914 | ARF6 |
| ILMN_2262044 | 0.005131 | 2.93E-04 | 0.219738 | 0.025105 | -0.37448 | 0.038541 | PARP10 |
| ILMN_1657749 | 0.017683 | 0.002556 | 0.369572 | 0.059197 | -0.18164 | -0.02551 | CYP27C1 |
| ILMN_1700518 | 0.003519 | 1.01E-04 | -0.2828 | 0.049636 | 0.270385 | -0.6678 | HMGN4 |
| ILMN_2272519 | 0.007649 | 6.58E-04 | 0.3859 | 0.044251 | -0.25747 | -0.00266 | FGR |
| ILMN_2384188 | 0.004626 | 2.23E-04 | 0.892439 | -0.40604 | 0.017103 | 0.636016 | NFATC1 |
| ILMN_2115534 | 0.004191 | 1.76E-04 | 0.329857 | -0.03756 | -0.27483 | 0.155433 | GPR112 |
| ILMN_1772876 | 0.048724 | 0.011228 | 0.12167 | 0.147845 | -0.1262 | -0.18833 | ZNF395 |
| ILMN_2251505 | 0.00353 | 1.02E-04 | 0.41082 | 0.009002 | -0.24207 | 0.421247 | ARPP-21 |
| ILMN_2088410 | 0.005246 | 3.10E-04 | 0.272773 | -0.15593 | -0.22468 | 0.342263 | PSMG2 |
| ILMN_1768506 | 0.036021 | 0.007269 | -0.1069 | 0.040416 | -0.33092 | -1.24014 | MAPK15 |
| ILMN_1801605 | 0.015127 | 0.002021 | 0.044962 | -0.0603 | 0.01638 | -0.71593 | BIRC6 |
| ILMN_1722834 | 0.036008 | 0.007263 | 0.235593 | 0.113895 | -0.20624 | 0.0946 | RGS12 |
| ILMN_1795715 | 0.043795 | 0.009614 | 0.206117 | 0.558754 | -0.08658 | -0.0489 | DPYD |
| ILMN_3243890 | 0.008761 | 8.30E-04 | 0.383611 | -0.13782 | -0.1259 | 0.282275 | NDUFA2 |
| ILMN_2230624 | 0.006004 | 4.08E-04 | 0.251905 | 0.019701 | -0.36618 | -0.06512 | RPL18 |
| ILMN_1718079 | 0.020241 | 0.003128 | 0.476655 | -0.02297 | -0.12553 | 0.231454 | HOXB1 |
| ILMN_1652024 | 0.002962 | 5.79E-05 | -0.85955 | -0.66771 | 0.487491 | -0.35473 | CSNK1G3 |
| ILMN_1757119 | 0.003604 | 1.10E-04 | 0.3129 | 0.024183 | -0.25283 | 0.251652 | KCNQ1DN |
| ILMN_1736847 | 8.85E-04 | 4.11E-06 | 0.579328 | -0.14113 | -0.28443 | 0.345604 | MED8 |
| ILMN_2150284 | 0.020225 | 0.00312 | -0.12557 | -0.04412 | 0.290769 | -1.33556 | RNPC3 |
| ILMN_1807169 | 0.021595 | 0.003435 | 0.178256 | -0.03222 | -0.32155 | 0.033764 | TINAGL1 |
| ILMN_1792679 | 0.026556 | 0.004678 | 0.343515 | 0.167031 | -0.1201 | 0.285544 | ITGA5 |
| ILMN_1712035 | 0.003279 | 8.31E-05 | 0.146958 | 0.003274 | -0.2966 | 0.190124 | TMEM115 |
| ILMN_2340908 | 0.007786 | 6.76E-04 | 0.180237 | -0.31314 | -0.34051 | 0.83616 | SCML1 |
| ILMN_1676058 | 0.014248 | 0.001837 | 0.138811 | 0.153621 | -0.345 | -0.0175 | MAGOHB |
| ILMN_2232494 | 0.003258 | 7.63E-05 | 0.079593 | 0.35969 | 0.19046 | -0.65692 | C5orf42 |
| ILMN_3250614 | 0.03641 | 0.007389 | -0.41991 | -0.0434 | 0.184268 | 0.07058 | EIF2AK1 |
| ILMN_3236211 | 0.028602 | 0.00523 | 0.141031 | 0.005762 | -0.3772 | -0.14674 | DGCR11 |
| ILMN_1702301 | 0.014546 | 0.001901 | -0.35704 | 0.236434 | 0.066727 | -0.66467 | DOCK10 |
| ILMN_2205622 | 0.012709 | 0.001543 | 0.304685 | 0.096098 | -0.19664 | 0.20354 | MUC2 |
| ILMN_1750901 | 0.0491 | 0.011357 | 0.161814 | -0.03647 | -0.25714 | 0.0915 | GNAT1 |
| ILMN_1766363 | 0.009709 | 9.82E-04 | 0.052265 | 0.688554 | -0.37241 | 0.854105 | FLT3 |
| ILMN_1799614 | 0.005988 | 4.06E-04 | 0.300584 | 0.08355 | -0.31401 | -0.14568 | PNPLA6 |
| ILMN_1805788 | 0.032935 | 0.00641 | -0.05211 | 0.484144 | -0.0384 | -0.16845 | PPHLN1 |
| ILMN_1726222 | 0.006662 | 5.12E-04 | 0.319609 | 0.093524 | -0.23802 | 0.14607 | FLOT2 |
| ILMN_2101278 | 0.012996 | 0.001598 | 0.203607 | 0.077953 | -0.32912 | -0.04855 | RGS18 |
| ILMN_2160906 | 0.038897 | 0.008077 | 0.102705 | -0.25512 | 0.319186 | -0.28632 | MYCT1 |
| ILMN_2052891 | 0.011023 | 0.001224 | -0.05375 | -0.20862 | 0.437275 | -0.80652 | PKD2 |
| ILMN_1797656 | 0.025779 | 0.004488 | 0.272585 | 0.077194 | -0.29969 | 0.030028 | ASCC3 |
| ILMN_1727996 | 0.007361 | 6.14E-04 | 0.233759 | 0.067487 | -0.21809 | -0.19033 | BAG4 |
| ILMN_1656287 | 0.0498 | 0.011557 | 0.313549 | -0.46985 | -0.11888 | 0.161656 | SPOCK2 |
| ILMN_1782621 | 0.008963 | 8.68E-04 | 0.150416 | -0.03586 | -0.30137 | 0.026353 | RPS12 |
| ILMN_1653871 | 0.005855 | 3.90E-04 | 0.189238 | -0.01699 | -0.00227 | 0.55173 | NAMPT |
| ILMN_1682098 | 0.04885 | 0.011274 | 0.138668 | 0.042784 | -0.13969 | -0.23218 | PSMA4 |
| ILMN_1777366 | 0.025819 | 0.004501 | 0.339691 | 0.072219 | -0.15605 | 0.078782 | TTC35 |
| ILMN_1692678 | 0.019012 | 0.002836 | -0.10814 | -0.63836 | 0.300923 | -0.67623 | FMNL3 |
| ILMN_2345512 | 0.014003 | 0.001795 | 0.312173 | 0.096894 | -0.24987 | 0.001992 | PPP4R1 |
| ILMN_3238525 | 0.002067 | 2.43E-05 | 0.663897 | -0.04828 | -0.20792 | 0.259307 | CARD17 |
| ILMN_3309269 | 0.030434 | 0.00574 | 0.327228 | -0.04499 | -0.25588 | 0.092686 | MIR557 |
| ILMN_1799819 | 0.004325 | 1.94E-04 | 0.297654 | -0.11933 | 0.042528 | -0.6174 | MARS |
| ILMN_1810725 | 0.019531 | 0.00294 | -0.03942 | -0.45091 | 0.224553 | -1.53839 | FAM129A |
| ILMN_1677466 | 0.008557 | 7.94E-04 | -0.15862 | -0.32312 | 0.10049 | 0.861616 | DUSP6 |
| ILMN_1744432 | 0.005131 | 2.93E-04 | 0.532395 | 0.122955 | -0.12961 | 0.151768 | COX8C |
| ILMN_1760374 | 0.040996 | 0.008732 | 0.234639 | 0.144777 | -0.35415 | 0.328283 | CD8A |
| ILMN_1722492 | 0.001486 | 1.21E-05 | -0.1635 | 0.156848 | 0.329382 | -0.62539 | DUSP19 |
| ILMN_1676406 | 0.00382 | 1.30E-04 | 0.40887 | -0.0333 | -0.26084 | 0.411487 | CTU2 |
| ILMN_1710052 | 0.018941 | 0.002816 | 0.700892 | -0.06749 | -0.10997 | 0.115326 | CPT1A |
| ILMN_3308951 | 0.018105 | 0.002638 | 0.585352 | 0.176163 | -0.23613 | 0.612113 | MIR200C |
| ILMN_1778836 | 0.019848 | 0.003013 | 0.055409 | -0.02703 | 0.338192 | -0.41976 | SFRS7 |
| ILMN_2053490 | 0.030668 | 0.005801 | 0.279305 | -0.0272 | -0.19248 | 0.017619 | FAM53B |
| ILMN_1754440 | 0.007413 | 6.22E-04 | 0.410121 | -0.10356 | -0.233 | 0.546184 | ADCY8 |
| ILMN_1728225 | 0.0143 | 0.001854 | 0.384175 | -0.10854 | -0.21661 | -0.01122 | KIAA1524 |
| ILMN_2099045 | 0.022221 | 0.003609 | 0.299864 | 0.121307 | -0.21311 | 0.005755 | KIAA1524 |
| ILMN_3196019 | 0.040967 | 0.008722 | 0.304176 | 0.086073 | -0.22641 | -0.02471 | FAM60A |
| ILMN_3235282 | 0.005396 | 3.28E-04 | 0.290369 | -0.02088 | -0.29045 | -0.01504 | ATAD2B |
| ILMN_1672295 | 0.001198 | 6.96E-06 | -0.15522 | 0.62935 | -0.28123 | 1.248144 | ZC3H12A |
| ILMN_1675898 | 0.002102 | 2.54E-05 | -0.46294 | 0.14053 | -0.30013 | 0.85781 | SH3BP5 |
| ILMN_1794147 | 0.047097 | 0.010694 | 0.282866 | -0.26982 | -0.23557 | 0.262478 | SMCR8 |
| ILMN_3251605 | 0.040081 | 0.008427 | -0.37984 | -0.21498 | 0.262067 | -0.3249 | KLHL28 |
| ILMN_1758090 | 0.011496 | 0.001311 | 0.349203 | -0.01515 | -0.24625 | 0.119987 | BCCIP |
| ILMN_1706031 | 0.0471 | 0.010701 | 0.419759 | -0.16254 | -0.08519 | 0.328319 | KANK1 |
| ILMN_1802096 | 0.005075 | 2.79E-04 | 0.238365 | 0.274688 | -0.32024 | -0.14111 | ABTB1 |
| ILMN_1756874 | 0.008538 | 7.91E-04 | 0.203437 | 0.610189 | -0.16844 | 0.130233 | FBXO42 |
| ILMN_2071446 | 0.004014 | 1.49E-04 | 0.637667 | 0.160069 | -0.18883 | 0.281552 | PI15 |
| ILMN_1728535 | 0.034282 | 0.006795 | -0.50946 | -0.3984 | 0.149956 | 0.666904 | COPZ1 |
| ILMN_2105643 | 0.003641 | 1.16E-04 | 0.345436 | 0.079248 | -0.26028 | 0.052091 | GPD2 |
| ILMN_3244343 | 0.046014 | 0.010322 | -0.0218 | -0.23242 | -0.55994 | 0.148146 | SNORA21 |
| ILMN_1665435 | 0.016528 | 0.002308 | 0.362733 | -8.23E-04 | -0.18083 | 0.383275 | GPER |
| ILMN_2384056 | 6.46E-04 | 2.60E-06 | 0.290736 | 0.074875 | -0.30936 | 0.306571 | GPER |
| ILMN_1694742 | 0.033124 | 0.006467 | 0.252877 | -0.02456 | -0.09928 | 0.092573 | RPS29 |
| ILMN_1738243 | 0.021498 | 0.003411 | 0.262665 | 0.094231 | -0.16113 | -0.05062 | RPS29 |
| ILMN_1758846 | 0.009895 | 0.001015 | 0.370808 | 0.232713 | -0.15594 | 0.126142 | ANKRD40 |
| ILMN_1744381 | 1.35E-06 | 5.74E-10 | 0.724486 | -0.16009 | -0.17422 | 1.923977 | SERPINE1 |
| ILMN_1759676 | 0.00554 | 3.48E-04 | 0.304794 | 0.09884 | -0.31041 | 0.108583 | HOXC13 |
| ILMN_3249756 | 0.003835 | 1.32E-04 | 0.513967 | 0.067239 | -0.24123 | 0.261681 | DNASE1L1 |
| ILMN_1727923 | 0.013988 | 0.001788 | 0.266113 | 0.07959 | -0.10855 | -0.39929 | ZNF140 |
| ILMN_1658232 | 0.004085 | 1.63E-04 | 0.267407 | 0.019302 | -0.34897 | 0.001776 | FBXL10 |
| ILMN_2388112 | 0.010965 | 0.00121 | 0.588197 | -0.14707 | -0.36387 | 0.489341 | CR1 |
| ILMN_1666449 | 0.006447 | 4.74E-04 | 0.346472 | 0.029866 | -0.2066 | 0.027877 | FAM134C |
| ILMN_3243924 | 0.022671 | 0.003717 | 0.464374 | 0.0635 | -0.276 | -0.35429 | P2RX6 |
| ILMN_1727183 | 0.035873 | 0.007231 | 0.033636 | -0.13664 | 0.011843 | -1.17667 | ZNF763 |
| ILMN_2374191 | 0.010714 | 0.001161 | 0.362524 | 0.11048 | -0.21513 | 0.063465 | FNTA |
| ILMN_1814606 | 0.006348 | 4.58E-04 | 0.548947 | -0.03407 | -0.24366 | 0.225097 | GALNT2 |
| ILMN_2252701 | 0.047218 | 0.010741 | 0.537467 | -0.15025 | -0.24611 | -0.09698 | SLC6A9 |
| ILMN_2345824 | 0.019508 | 0.002931 | 0.285155 | 0.553327 | -0.36701 | -0.35947 | PCDHGC3 |
| ILMN_1745332 | 0.010654 | 0.00115 | 0.379901 | 0.015976 | -0.20486 | 0.120613 | PPP5C |
| ILMN_1701402 | 0.032497 | 0.006297 | 0.301403 | -0.01921 | -0.15142 | -0.33802 | IKBIP |
| ILMN_1655561 | 0.014015 | 0.001798 | 0.255672 | -0.17862 | -0.19861 | 0.150217 | ARPC3 |
| ILMN_3309164 | 0.004785 | 2.40E-04 | 0.1063 | -0.0315 | -0.33366 | 0.179848 | MIR1185-1 |
| ILMN_1757132 | 0.01805 | 0.002629 | 0.378278 | 0.030174 | -0.13167 | 0.111798 | SMC5 |
| ILMN_1703123 | 1.30E-04 | 1.83E-07 | 0.432323 | 0.038165 | -0.15816 | 0.930645 | AXUD1 |
| ILMN_1803650 | 0.004944 | 2.61E-04 | 0.294123 | 0.066382 | -0.18736 | 0.302512 | MYCL1 |
| ILMN_2175659 | 0.005837 | 3.87E-04 | 0.162869 | 0.043852 | -0.33374 | 0.171298 | NUDT8 |
| ILMN_1804676 | 0.009747 | 9.87E-04 | -0.3436 | -0.0798 | 0.471183 | -0.08845 | VAMP4 |
| ILMN_2051900 | 0.001835 | 1.70E-05 | -0.34569 | -0.0446 | 0.543743 | -0.98086 | EID2B |
| ILMN_2375879 | 0.004922 | 2.56E-04 | -0.06345 | -0.07927 | 0.638444 | -0.35811 | VEGFA |
| ILMN_1712431 | 0.002696 | 4.52E-05 | 0.176155 | 0.218383 | -0.3315 | 0.171659 | FAM113B |
| ILMN_2394250 | 0.022268 | 0.003619 | -0.28911 | -0.50391 | 0.271558 | -0.24565 | PLEKHA1 |
| ILMN_2393060 | 0.04782 | 0.010932 | 0.282598 | 0.100419 | -0.22819 | -0.12745 | PPIL3 |
| ILMN_1748661 | 0.032273 | 0.006234 | 0.185169 | 0.007591 | -0.25449 | 0.104762 | AKT1 |
| ILMN_1722680 | 0.014481 | 0.001888 | 0.304413 | 0.030635 | -0.2385 | 0.078563 | DSEL |
| ILMN_1723353 | 0.0409 | 0.008704 | -0.58896 | -0.23527 | 0.139691 | 0.090212 | FAM122A |
| ILMN_1745529 | 0.00716 | 5.87E-04 | 0.420928 | -0.01724 | -0.25347 | 0.171045 | KCNH1 |
| ILMN_1696276 | 0.048756 | 0.011239 | 0.097628 | 0.116195 | -0.23607 | 0.124933 | ZNF653 |
| ILMN_1730888 | 0.02423 | 0.004081 | -0.23958 | 0.236929 | 0.143694 | -0.80209 | ZNF680 |
| ILMN_1805192 | 0.002338 | 3.14E-05 | 0.239668 | -0.13105 | -0.05245 | 0.464297 | ITPRIP |
| ILMN_1780582 | 0.007054 | 5.71E-04 | 0.113799 | -0.41393 | -0.38596 | 0.585699 | CD83 |
| ILMN_1667381 | 0.038897 | 0.008074 | 0.253413 | -0.08713 | -0.22113 | 0.375204 | CAMKV |
| ILMN_1729775 | 0.028385 | 0.005169 | 0.006323 | 0.031624 | -0.07813 | 0.428901 | OPA1 |
| ILMN_2356786 | 0.013762 | 0.001738 | 0.321928 | 0.103109 | -0.20782 | 0.097823 | ADD1 |
| ILMN_2371280 | 0.02039 | 0.003161 | 0.001352 | 0.542773 | -0.30973 | -0.35429 | CSF3R |
| ILMN_1659659 | 0.027114 | 0.004809 | 0.234367 | 0.14755 | -0.13916 | -0.05427 | MAGEH1 |
| ILMN_2078226 | 0.00499 | 2.67E-04 | 0.257247 | 0.05624 | -0.21629 | 0.254559 | MAGEH1 |
| ILMN_3239331 | 0.013846 | 0.001757 | 0.482595 | -0.08779 | -0.18228 | 0.091459 | U58 |
| ILMN_2080751 | 0.024892 | 0.004246 | 0.20629 | -0.02292 | -0.1256 | 0.266806 | ADNP2 |
| ILMN_2255130 | 0.003408 | 9.36E-05 | 0.170762 | 0.019591 | -0.27692 | 0.198052 | CCL15 |
| ILMN_1653386 | 0.006447 | 4.74E-04 | 0.537067 | 0.120169 | -0.28569 | 0.136934 | SULT6B1 |
| ILMN_2130409 | 0.027962 | 0.005047 | 0.056053 | -0.22699 | 0.094107 | 0.467291 | PEX26 |
| ILMN_1691575 | 0.044652 | 0.009875 | -0.15139 | -0.18045 | 0.515169 | -0.66974 | SNX2 |
| ILMN_2275060 | 0.005608 | 3.57E-04 | 0.392446 | 0.029727 | -0.25536 | 0.163516 | GHRHR |
| ILMN_1685318 | 0.005956 | 4.01E-04 | 0.317911 | 0.072174 | -0.25278 | 0.148603 | EN2 |
| ILMN_1764043 | 0.008853 | 8.49E-04 | 0.33652 | 0.041269 | -0.13201 | -0.0996 | TTL |
| ILMN_1800795 | 0.043645 | 0.00957 | 0.410012 | 0.263269 | -0.16735 | -0.16989 | LOC340260 |
| ILMN_2139761 | 0.04695 | 0.010638 | -0.05121 | -0.3662 | 0.130438 | -0.47786 | LIMCH1 |
| ILMN_1756220 | 0.008482 | 7.82E-04 | -0.04002 | -0.00215 | -0.1669 | 0.234233 | DDX18 |
| ILMN_1670918 | 0.004014 | 1.49E-04 | 0.347922 | -0.06231 | -0.34317 | 0.087228 | INSR |
| ILMN_1730631 | 0.011476 | 0.001299 | 0.238418 | 0.114897 | -0.05143 | -0.88801 | C2orf44 |
| ILMN_1721495 | 0.018247 | 0.002679 | 0.27212 | 0.05184 | -0.23597 | 0.038196 | ADAMTSL2 |
| ILMN_3310509 | 8.85E-04 | 4.14E-06 | 0.052338 | -0.29357 | 0.270633 | -1.2944 | MIR30C2 |
| ILMN_1777706 | 0.00541 | 3.32E-04 | 0.25569 | 0.037306 | -0.27259 | 0.14349 | OR4C12 |
| ILMN_1699836 | 0.002034 | 2.34E-05 | 0.307739 | 0.099934 | -0.27759 | 0.203262 | LSP1 |
| ILMN_1694671 | 0.015265 | 0.002061 | 0.226108 | 0.067914 | -0.26356 | 0.103342 | ZFAND2A |
| ILMN_1736546 | 0.004377 | 2.01E-04 | 0.374276 | 0.066556 | -0.2178 | 0.032847 | SLC16A14 |
| ILMN_1721541 | 0.003028 | 6.27E-05 | 0.342024 | 0.039203 | -0.50918 | 0.208195 | WIF1 |
| ILMN_3307954 | 0.008204 | 7.37E-04 | 0.329264 | 0.037002 | -0.21366 | 0.142364 | L3MBTL4 |
| ILMN_1788759 | 0.003649 | 1.17E-04 | 0.274014 | -0.07252 | -0.28207 | 0.250982 | DSCAM |
| ILMN_1658420 | 0.011906 | 0.001387 | 0.122551 | -0.07746 | -0.26774 | 0.173838 | DSCAM |
| ILMN_3234941 | 0.003027 | 6.16E-05 | 0.491334 | -0.09036 | -0.25731 | 0.17973 | LCE4A |
| ILMN_1789642 | 0.003387 | 8.94E-05 | -0.42479 | -0.54338 | -0.41857 | 1.390227 | DNAJC5 |
| ILMN_1784948 | 0.029216 | 0.005401 | 0.493863 | 0.657368 | -0.41319 | -0.13898 | SPOCD1 |
| ILMN_2094166 | 0.032951 | 0.006418 | -0.21487 | -0.67464 | 0.169486 | -0.29068 | CHMP5 |
| ILMN_1808795 | 0.007944 | 7.01E-04 | 0.443066 | -0.12528 | -0.22701 | 0.045221 | NAALADL2 |
| ILMN_1805916 | 0.001907 | 1.97E-05 | -0.0766 | -0.2467 | 0.534331 | -0.88491 | NIPSNAP1 |
| ILMN_1663858 | 0.01259 | 0.001517 | -0.40692 | -0.02554 | 0.14231 | -1.31647 | ZNF286A |
| ILMN_1718565 | 0.003387 | 9.03E-05 | -0.3372 | -0.50892 | 0.253117 | 0.476608 | CDKN1C |
| ILMN_1688886 | 0.027893 | 0.005026 | 0.214776 | -0.46296 | 0.486469 | -0.51971 | GPC5 |
| ILMN_1761531 | 0.028863 | 0.00531 | 0.12808 | 0.158928 | -0.31255 | 0.338736 | SGPL1 |
| ILMN_1734991 | 0.006396 | 4.66E-04 | 0.116564 | -0.04656 | -0.35713 | 0.14028 | PPM1B |
| ILMN_1794560 | 0.004739 | 2.36E-04 | 0.320571 | 0.057007 | -0.2548 | 0.088169 | TMEM93 |
| ILMN_1730794 | 0.027303 | 0.004874 | 0.315748 | 0.350262 | -0.06622 | -0.88782 | SERTAD4 |
| ILMN_1790136 | 5.01E-04 | 1.54E-06 | 0.152343 | 0.092864 | -0.33172 | 0.206019 | C20orf20 |
| ILMN_2360047 | 0.007768 | 6.74E-04 | 0.182836 | 0.148565 | -0.2227 | 0.319679 | MAGEA11 |
| ILMN_1711504 | 0.006077 | 4.18E-04 | 0.40446 | -0.00252 | -0.19813 | 0.20421 | NRXN1 |
| ILMN_3307877 | 0.002811 | 5.02E-05 | 0.036731 | 0.319729 | 0.091461 | -0.8852 | C21orf58 |
| ILMN_1689655 | 0.006404 | 4.68E-04 | 0.24708 | 0.228532 | -0.34689 | -0.16664 | HLA-DRA |
| ILMN_3235964 | 0.010927 | 0.001202 | 0.123238 | 0.092212 | -0.22891 | 0.116483 | HSPA7 |
| ILMN_1708369 | 0.027354 | 0.004891 | -0.69096 | -0.49802 | 0.249537 | -0.55398 | EPS15L1 |
| ILMN_1671087 | 0.005113 | 2.86E-04 | 0.352616 | 0.069543 | -0.28258 | 0.250217 | C6orf222 |
| ILMN_1691164 | 0.003835 | 1.32E-04 | 0.367094 | 0.006099 | -0.31518 | 0.094084 | TGM7 |
| ILMN_1768391 | 0.011758 | 0.001362 | 0.375765 | -0.00169 | -0.22067 | 0.103201 | ARL4C |
| ILMN_1738263 | 0.009129 | 8.95E-04 | 0.201445 | 0.048676 | -0.16157 | 0.104984 | PIGU |
| ILMN_1779743 | 0.006616 | 5.03E-04 | 0.596287 | 0.12252 | -0.19532 | 0.370307 | SYCP2L |
| ILMN_3251501 | 0.018481 | 0.002726 | -0.10679 | -0.14594 | 0.210684 | -0.8997 | SERF2 |
| ILMN_1789136 | 0.012416 | 0.001482 | 0.309459 | -0.11289 | -0.27186 | 0.253676 | SERF2 |
| ILMN_1754207 | 0.021123 | 0.00332 | 0.359196 | 0.102135 | -0.22347 | 0.184957 | PLAC1 |
| ILMN_1760347 | 0.023831 | 0.003991 | 0.139243 | -0.1715 | -0.14571 | 0.29138 | SRGN |
| ILMN_2169152 | 0.00651 | 4.90E-04 | 0.131269 | -0.20357 | -0.31086 | 0.803085 | SRGN |
| ILMN_1815012 | 0.013651 | 0.001716 | 0.08609 | 0.333644 | -0.09424 | -0.27568 | EXOC7 |
| ILMN_1750011 | 0.002998 | 6.07E-05 | 0.388798 | -0.00448 | -0.17708 | 0.081917 | EXOC7 |
| ILMN_2169089 | 0.004305 | 1.92E-04 | 0.264697 | 0.140787 | -0.20728 | 0.276207 | C18orf54 |
| ILMN_1785285 | 0.003243 | 7.44E-05 | 0.37544 | 0.12955 | -0.31767 | 0.14664 | ABHD13 |
| ILMN_1781383 | 0.006339 | 4.56E-04 | 0.331405 | 0.060248 | -0.2888 | 0.0669 | NFKBIL2 |
| ILMN_1692368 | 0.010017 | 0.001032 | 0.528863 | 0.110196 | -0.36036 | 0.308516 | DLX2 |
| ILMN_1690922 | 0.007291 | 6.07E-04 | 0.419374 | 0.152383 | -0.17789 | 3.09E-04 | MAST2 |
| ILMN_1801616 | 0.001241 | 7.50E-06 | 0.023355 | -0.11429 | -0.17857 | 0.721106 | EMP1 |
| ILMN_1686867 | 0.011467 | 0.001297 | 0.547191 | 0.001885 | -0.15267 | 0.066769 | GJA8 |
| ILMN_1664922 | 0.04515 | 0.010056 | 0.261078 | -0.17144 | -0.14438 | 0.184354 | FLNB |
| ILMN_3240155 | 0.010404 | 0.001105 | 0.420275 | 0.003062 | -0.29428 | 0.033925 | RNU105A |
| ILMN_2397795 | 0.005131 | 2.93E-04 | 1.102301 | 0.257909 | -0.20737 | 0.546496 | SLAIN1 |
| ILMN_1811954 | 0.003817 | 1.30E-04 | 0.462898 | 0.123751 | -0.2425 | 0.298455 | NRG1 |
| ILMN_1666376 | 0.012583 | 0.001514 | 0.124247 | -0.07311 | -0.26449 | 0.106078 | TRIM56 |
| ILMN_1682339 | 0.007668 | 6.61E-04 | 0.173237 | 0.014984 | -0.21283 | 0.161308 | C19orf57 |
| ILMN_3242253 | 0.021595 | 0.003437 | 0.257444 | 0.072992 | -0.31676 | -0.04501 | LOC653501 |
| ILMN_1799871 | 0.003917 | 1.40E-04 | 0.64237 | 0.1463 | -0.20865 | 0.230979 | LTK |
| ILMN_1752006 | 0.012599 | 0.001524 | 0.290643 | 0.069659 | -0.21306 | 0.161531 | COX18 |
| ILMN_1681374 | 0.00348 | 9.81E-05 | 0.416733 | -0.00987 | -0.22802 | 0.237004 | ALDOA |
| ILMN_1725528 | 0.043487 | 0.009525 | 0.119044 | 0.217439 | 0.014744 | -0.59558 | LOC400657 |
| ILMN_1663113 | 0.019652 | 0.002963 | -0.04505 | -0.18768 | 0.421955 | -0.77687 | TTLL12 |
| ILMN_2234412 | 0.00875 | 8.26E-04 | 0.416003 | -0.15133 | -0.30759 | 0.309975 | TLE3 |
| ILMN_2408572 | 0.042875 | 0.009302 | -0.31319 | -0.02853 | -0.48849 | 0.989451 | RNASE4 |
| ILMN_2190084 | 0.023655 | 0.00395 | 0.269881 | 0.060786 | -0.20339 | -0.19286 | VAMP8 |
| ILMN_1709020 | 0.006281 | 4.48E-04 | 0.416913 | 0.033552 | -0.28376 | 0.088639 | MYBL2 |
| ILMN_2231177 | 0.043167 | 0.009398 | 0.446321 | 0.103249 | -0.24727 | 0.134523 | OR7E24 |
| ILMN_1758457 | 0.004255 | 1.86E-04 | 0.544513 | -0.028 | -0.2759 | -0.05833 | TBC1D16 |
| ILMN_1728426 | 0.046853 | 0.010589 | -0.16058 | -0.16992 | -0.02269 | -0.45009 | INPPL1 |
| ILMN_1715574 | 0.010527 | 0.001129 | 0.258614 | 0.075828 | -0.2076 | 0.035357 | ZC3H11A |
| ILMN_2118910 | 0.01816 | 0.002654 | 0.246041 | -3.61E-04 | -0.18223 | -0.01881 | TPRKB |
| ILMN_1690307 | 0.004063 | 1.57E-04 | 0.374828 | -0.00694 | -0.20797 | 0.166299 | TPRKB |
| ILMN_2235745 | 0.006484 | 4.82E-04 | 0.29035 | -0.04795 | -0.2937 | 0.066115 | GRIPAP1 |
| ILMN_1747870 | 0.004357 | 1.98E-04 | 0.438207 | 0.035143 | -0.2149 | 0.120034 | CD3EAP |
| ILMN_1717702 | 0.016726 | 0.002354 | 0.372174 | 0.224799 | -0.25317 | 0.121705 | KCNK17 |
| ILMN_1809607 | 0.030341 | 0.005708 | 0.350146 | -0.32967 | -0.21982 | 0.29479 | PPIF |
| ILMN_1687430 | 0.04338 | 0.009481 | 0.117791 | -0.18309 | 0.194443 | -1.19192 | EIF2B4 |
| ILMN_1707123 | 3.71E-04 | 9.46E-07 | 0.291634 | 0.206008 | -0.40241 | 0.449196 | DDX19B |
| ILMN_1666976 | 0.028147 | 0.005098 | 0.221893 | 0.355601 | -0.08482 | 0.241723 | PLD3 |
| ILMN_1713013 | 0.034734 | 0.006921 | 0.367836 | -0.10703 | -0.24481 | 0.063075 | PLD3 |
| ILMN_1662316 | 0.004054 | 1.54E-04 | 0.432595 | 0.018863 | -0.27017 | 0.126379 | VPS33A |
| ILMN_2324776 | 0.015499 | 0.002106 | 0.470165 | 0.361117 | -0.29174 | 0.254588 | TAS1R1 |
| ILMN_1752226 | 0.013839 | 0.001753 | 0.015473 | 0.207615 | -0.37853 | 0.129871 | P2RY11 |
| ILMN_2144088 | 0.019145 | 0.002863 | 0.2802 | -0.00256 | -0.1594 | 0.16191 | FDFT1 |
| ILMN_3242544 | 0.001973 | 2.09E-05 | 0.130974 | -0.06322 | -0.52977 | 0.055122 | BAHCC1 |
| ILMN_1794643 | 0.033438 | 0.006547 | 0.245132 | -0.27656 | -0.23111 | 0.29225 | ZGPAT |
| ILMN_2181593 | 0.011858 | 0.001379 | 0.163444 | -0.02499 | -0.25368 | 0.164799 | MAGEA1 |
| ILMN_2409596 | 0.02198 | 0.003536 | 0.340134 | 0.148314 | -0.18389 | 0.089202 | RAB11FIP1 |
| ILMN_1795018 | 0.006471 | 4.78E-04 | 0.219098 | 0.052462 | -0.16373 | 0.28091 | OR7C2 |
| ILMN_1673712 | 0.028295 | 0.005148 | 0.34706 | 0.197655 | -0.26413 | 0.119074 | GPR149 |
| ILMN_1759973 | 0.014311 | 0.001857 | -0.18211 | 0.065712 | 0.219931 | -0.52132 | NDUFA5 |
| ILMN_2352159 | 0.005751 | 3.77E-04 | 0.30273 | 0.042457 | -0.25521 | 0.156132 | BBS9 |
| ILMN_1756360 | 0.011682 | 0.001349 | 0.341065 | 0.022194 | -0.29649 | 0.099873 | RPL35A |
| ILMN_2150802 | 0.009634 | 9.69E-04 | 0.241785 | 0.230362 | -0.24763 | -0.4883 | FLJ22795 |
| ILMN_1786720 | 0.005738 | 3.74E-04 | 0.333475 | 0.270617 | -0.23878 | -0.70211 | PROM1 |
| ILMN_1677680 | 0.038897 | 0.00807 | -0.55898 | -0.02487 | 0.294232 | 0.143888 | FGF12 |
| ILMN_1809639 | 0.008801 | 8.39E-04 | -0.04543 | -0.05026 | 0.237198 | -0.71894 | TMEM26 |
| ILMN_2193892 | 0.002418 | 3.37E-05 | 0.365886 | 0.01839 | -0.33214 | 0.178863 | SELE |
| ILMN_1690343 | 0.005743 | 3.76E-04 | 0.295448 | 0.033178 | -0.28376 | 0.171349 | IMPDH1 |
| ILMN_1737847 | 0.02463 | 0.004181 | 0.245784 | 0.175782 | -0.19992 | 0.354339 | RMND5B |
| ILMN_1653466 | 0.006328 | 4.55E-04 | 0.266904 | 0.084515 | -0.21657 | 0.208841 | HES4 |
| ILMN_1727840 | 0.01007 | 0.001041 | -0.16251 | -0.75071 | 0.172316 | -1.10256 | SLC35B1 |
| ILMN_1657571 | 0.007658 | 6.60E-04 | 0.396832 | 0.0768 | -0.19797 | 0.03964 | ASNA1 |
| ILMN_3246169 | 0.003917 | 1.40E-04 | 0.529247 | 0.145781 | -0.20744 | 0.242682 | PRR20D |
| ILMN_2089073 | 0.010878 | 0.001192 | 0.174703 | -0.01757 | -0.22292 | 0.091424 | ATP9A |
| ILMN_1706406 | 0.001409 | 1.07E-05 | 0.524569 | 0.100882 | -0.30725 | 0.105645 | ATP9A |
| ILMN_1705231 | 0.034897 | 0.006968 | -0.16883 | -0.13538 | 0.219072 | -0.80785 | SLCO2A1 |
| ILMN_3241051 | 0.007806 | 6.78E-04 | 0.095109 | 0.045408 | -0.14875 | -1.14824 | LOC644907 |
| ILMN_1814313 | 0.004371 | 1.99E-04 | 0.443048 | 0.061117 | -0.27103 | 0.063435 | CREBL1 |
| ILMN_3238613 | 0.04848 | 0.011152 | 0.196469 | -0.11483 | -0.4687 | -0.04321 | SNORA26 |
| ILMN_2390974 | 0.008221 | 7.43E-04 | 0.364079 | 0.081626 | -0.25854 | -0.03527 | DNAJB2 |
| ILMN_2285213 | 0.03088 | 0.005863 | 0.340102 | 0.275335 | -0.41586 | 0.212276 | DNAJB2 |
| ILMN_2164978 | 0.014797 | 0.001952 | -0.43438 | -0.69827 | 0.410786 | -0.12298 | PDP2 |
| ILMN_1730005 | 0.019873 | 0.003028 | 0.420604 | 0.11 | -0.11553 | -0.10614 | RFP |
| ILMN_1751744 | 0.020144 | 0.003103 | 0.440207 | 0.232831 | -0.05191 | 0.234541 | ANKRD41 |
| ILMN_2041101 | 0.042906 | 0.009311 | -0.18568 | 0.165687 | -0.01843 | -0.98201 | ANXA2P1 |
| ILMN_1677432 | 0.022274 | 0.003621 | 0.259521 | -0.26884 | 0.111165 | -0.48084 | SRGAP1 |
| ILMN_2337241 | 0.005399 | 3.28E-04 | 0.325714 | 0.1056 | -0.13828 | 0.134902 | RPS15A |
| ILMN_1663592 | 0.003399 | 9.23E-05 | 0.40932 | -0.05521 | -0.30194 | 0.089585 | BBS5 |
| ILMN_1792568 | 0.033068 | 0.006452 | 0.443935 | 0.093428 | -0.22811 | 0.425826 | KRT9 |
| ILMN_1698218 | 0.002863 | 5.27E-05 | 0.300742 | -0.00734 | -0.41305 | 0.023613 | TRAF1 |
| ILMN_2171789 | 0.001853 | 1.83E-05 | 0.343763 | 0.040032 | -0.32221 | 0.12436 | TRAF1 |
| ILMN_1812473 | 0.015525 | 0.002112 | 0.310781 | 0.071791 | -0.19311 | 0.06679 | MLLT3 |
| ILMN_1754795 | 0.016511 | 0.002304 | 0.017068 | -0.06101 | 0.166965 | -0.65488 | FAT1 |
| ILMN_1733157 | 0.032681 | 0.006351 | 0.426705 | 0.001448 | -0.30115 | 0.07747 | THSD1 |
| ILMN_3308763 | 0.008831 | 8.43E-04 | 0.283831 | 0.022826 | -0.06838 | 0.305701 | MIR377 |
| ILMN_1664111 | 0.040693 | 0.008633 | -0.53932 | -0.39989 | 0.019508 | 0.483861 | RFFL |
| ILMN_1777765 | 0.003279 | 8.24E-05 | 0.287271 | -0.04705 | -0.26012 | 0.221024 | C12orf10 |
| ILMN_2366642 | 0.013358 | 0.001662 | 0.297008 | -0.02035 | -0.24519 | 0.196123 | VCX3A |
| ILMN_3246014 | 0.003751 | 1.23E-04 | 0.286891 | 0.05488 | -0.24093 | 0.131688 | C1orf130 |
| ILMN_2310001 | 0.003917 | 1.39E-04 | 0.50842 | 0.170383 | -0.23387 | 0.340831 | MOG |
| ILMN_3241626 | 6.45E-04 | 2.54E-06 | -0.36059 | 0.101358 | 0.267977 | -0.77548 | QRFPR |
| ILMN_3234837 | 0.004014 | 1.49E-04 | -0.45562 | 0.233547 | 0.071817 | -0.58021 | PKDCC |
| ILMN_1662023 | 0.019776 | 0.002998 | 0.268699 | -0.00785 | -0.20511 | 0.016293 | KLF7 |
| ILMN_1814151 | 0.022188 | 0.0036 | 0.143731 | 0.069447 | -0.25801 | -0.0137 | AGR2 |
| ILMN_1719194 | 0.005404 | 3.31E-04 | 0.462078 | 0.037662 | -0.23656 | 0.289646 | IBSP |
| ILMN_1748393 | 0.040086 | 0.008438 | 0.011539 | -0.34582 | 0.011247 | -1.09146 | mar-09 |
| ILMN_2053527 | 0.017535 | 0.002524 | 0.398076 | 0.10676 | -0.07322 | 0.069022 | PARP9 |
| ILMN_2233689 | 0.023639 | 0.003944 | 0.429543 | 0.045903 | -0.20476 | 0.111272 | MS4A13 |
| ILMN_1658678 | 0.010922 | 0.0012 | -0.1265 | -0.73074 | 0.498798 | -0.44498 | SAAL1 |
| ILMN_1720617 | 0.011496 | 0.001311 | 0.286862 | 0.057066 | -0.27246 | 0.160845 | OR2T6 |
| ILMN_3236599 | 0.00577 | 3.79E-04 | 0.287576 | 0.037698 | -0.28363 | 0.187223 | FLJ44054 |
| ILMN_1652381 | 0.003061 | 6.45E-05 | 0.207369 | 0.115263 | -0.33645 | 0.768264 | SIGLEC5 |
| ILMN_2224619 | 0.008337 | 7.59E-04 | 0.524948 | 0.099119 | -0.14061 | 0.293151 | GNRHR2 |
| ILMN_1670218 | 0.004337 | 1.95E-04 | 0.324998 | 0.063287 | -0.23119 | 0.065149 | EXOSC6 |
| ILMN_2129102 | 0.021927 | 0.003522 | -0.43297 | 0.220079 | 0.399804 | -0.60993 | FLJ40453 |
| ILMN_1731498 | 0.005117 | 2.87E-04 | 0.531281 | 0.08508 | -0.32279 | 0.120436 | GPR156 |
| ILMN_2348512 | 0.00716 | 5.87E-04 | 0.209054 | -0.07108 | -0.34753 | -0.01163 | ZXDC |
| ILMN_1744713 | 0.028107 | 0.005086 | 0.283314 | 0.012082 | -0.11765 | 0.005777 | PARK7 |
| ILMN_1743655 | 0.045988 | 0.010314 | -0.5351 | -0.70193 | 0.025646 | -0.12541 | TMED9 |
| ILMN_1664630 | 0.00577 | 3.79E-04 | 0.333055 | -0.34123 | -0.29756 | 0.029991 | CHEK1 |
| ILMN_1713636 | 0.00616 | 4.27E-04 | 0.181478 | 0.155244 | -0.07695 | -0.4712 | S100A6 |
| ILMN_2341467 | 0.029547 | 0.0055 | -0.51014 | -0.39833 | 0.130573 | -0.0195 | PARL |
| ILMN_1774330 | 0.037798 | 0.007753 | 0.427307 | 0.012721 | 0.199775 | -1.27812 | WSCD1 |
| ILMN_1744794 | 0.004639 | 2.26E-04 | 0.731189 | -0.01884 | -0.22785 | 0.136492 | USP26 |
| ILMN_2383913 | 0.004191 | 1.76E-04 | 0.168994 | 0.134053 | -0.23639 | 0.28002 | WDR33 |
| ILMN_2415235 | 0.022067 | 0.003561 | 0.237887 | 0.194059 | -0.12931 | 0.071129 | CSNK1E |
| ILMN_1724877 | 0.017954 | 0.002611 | 0.255595 | -0.52966 | -0.20708 | 0.852521 | PARD6G |
| ILMN_2048388 | 0.004671 | 2.31E-04 | 0.434487 | 0.100315 | -0.20418 | 0.184721 | ZNF643 |
| ILMN_1671557 | 9.80E-04 | 4.86E-06 | 0.449446 | 0.130916 | -0.30935 | 0.147532 | PHLDA2 |
| ILMN_1673113 | 0.033922 | 0.00669 | -0.53968 | -0.17125 | 0.348732 | -0.23367 | F2RL1 |
| ILMN_1730622 | 0.004131 | 1.67E-04 | 0.29439 | 0.212659 | -0.26319 | -0.00453 | EVL |
| ILMN_1700034 | 0.017066 | 0.00242 | 0.405784 | -0.09016 | -0.26707 | 0.039247 | C4orf6 |
| ILMN_1707448 | 0.011989 | 0.001404 | -0.25529 | -0.09926 | 0.185913 | -0.46621 | CRKRS |
| ILMN_1694007 | 0.018156 | 0.002652 | 0.400866 | 0.077801 | -0.25666 | 0.308149 | ADRB3 |
| ILMN_1756071 | 0.001403 | 1.04E-05 | 0.325917 | 0.095702 | -0.31203 | 0.074731 | MFGE8 |
| ILMN_1667893 | 0.007968 | 7.05E-04 | 0.182391 | -0.05882 | -0.09461 | 0.144833 | TNS3 |
| ILMN_1701112 | 0.013397 | 0.001671 | -0.11195 | 0.042292 | 0.293978 | -0.30375 | LOC440944 |
| ILMN_1787275 | 0.001762 | 1.58E-05 | 0.779851 | 0.042498 | -0.49474 | 1.073946 | STAG3 |
| ILMN_1719254 | 0.017293 | 0.002469 | 0.194671 | -0.28654 | -0.57337 | 0.118347 | TRIM9 |
| ILMN_1693394 | 0.012518 | 0.001501 | 0.270233 | -0.01947 | -0.29645 | 0.069669 | BCKDK |
| ILMN_1789558 | 0.0256 | 0.00444 | -0.11358 | -0.55167 | 0.176183 | -0.23393 | FAM164A |
| ILMN_2373515 | 0.014599 | 0.001911 | 0.341695 | 0.092539 | -0.20375 | -0.02107 | HSP90AA1 |
| ILMN_2411794 | 0.004474 | 2.09E-04 | 0.310418 | 0.145739 | -0.24122 | -0.08013 | PTPRA |
| ILMN_2335198 | 0.026726 | 0.004724 | 0.189213 | -0.11891 | -0.20572 | 0.024606 | NCOA1 |
| ILMN_1775739 | 0.003177 | 7.16E-05 | 0.399411 | -0.01134 | -0.31281 | -0.02423 | CDH20 |
| ILMN_1686679 | 0.016564 | 0.00232 | -0.11303 | -0.28962 | 0.05339 | -1.27757 | ZNF462 |
| ILMN_3240306 | 0.00187 | 1.91E-05 | 1.261542 | -0.38246 | -0.37254 | 0.619452 | SNORD51 |
| ILMN_2106380 | 0.001337 | 9.10E-06 | -1.20577 | -0.23666 | 0.697256 | -0.8049 | TNFSF15 |
| ILMN_1769032 | 0.010557 | 0.001134 | 0.251154 | -0.1425 | -0.38543 | 0.007829 | GLYATL1 |
| ILMN_1717029 | 0.034913 | 0.006973 | -0.16161 | 0.663736 | -0.27572 | 1.598791 | FLJ33590 |
| ILMN_3230508 | 0.003279 | 8.28E-05 | 0.031584 | 0.528813 | -0.17254 | 1.757029 | NCRNA00152 |
| ILMN_2079508 | 0.02412 | 0.004055 | -0.05586 | 0.130993 | 0.376493 | -0.69439 | ZNF204 |
| ILMN_2259633 | 6.64E-05 | 7.21E-08 | 0.392505 | -0.2033 | -0.42728 | 0.524054 | MLL5 |
| ILMN_1754811 | 0.002393 | 3.24E-05 | 0.267638 | 0.079816 | -0.3226 | 0.268216 | FBXO38 |
| ILMN_1774828 | 0.029883 | 0.00559 | 0.146997 | 0.102897 | -0.1636 | -0.17235 | VEZT |
| ILMN_2108366 | 0.007888 | 6.91E-04 | 0.312015 | 0.211743 | -0.25451 | -0.13709 | RBM41 |
| ILMN_2328952 | 0.021498 | 0.00341 | 0.120779 | 0.032714 | -0.26304 | 0.050247 | CLCC1 |
| ILMN_1697830 | 0.008801 | 8.39E-04 | 0.236655 | 0.061433 | -0.28063 | 0.31555 | MS4A1 |
| ILMN_1806023 | 0.010939 | 0.001204 | 0.155443 | -0.05389 | -0.03958 | 0.998939 | JUN |
| ILMN_1722850 | 0.008642 | 8.05E-04 | 0.049607 | 0.010798 | -0.21818 | 0.086841 | VGLL2 |
| ILMN_1671583 | 0.003888 | 1.36E-04 | 0.345428 | -0.04807 | -0.12775 | -0.0873 | MKRN1 |
| ILMN_1802615 | 0.012216 | 0.00144 | -0.00293 | -0.0821 | 0.212599 | -0.68196 | CDK6 |
| ILMN_1778788 | 0.006706 | 5.22E-04 | 0.263296 | -0.02128 | -0.23854 | 0.060897 | AMOTL2 |
| ILMN_1702764 | 0.023746 | 0.003969 | 0.135268 | 0.101771 | -0.26546 | 0.588111 | mar-05 |
| ILMN_2257432 | 0.006683 | 5.19E-04 | 0.276421 | -0.02128 | -0.30743 | 0.058512 | RAD51 |
| ILMN_2241775 | 0.004808 | 2.42E-04 | 0.398616 | 0.005693 | -0.20496 | 0.053932 | TROVE2 |
| ILMN_1798104 | 0.025793 | 0.004493 | 0.097196 | 0.055922 | -0.26913 | -0.32808 | GRIN2A |
| ILMN_1673356 | 0.001375 | 9.93E-06 | 0.520238 | 0.015133 | -0.35366 | -0.00454 | FAM83C |
| ILMN_1698020 | 0.020015 | 0.003067 | 0.184297 | 0.252014 | -0.26874 | -0.2733 | DLC1 |
| ILMN_3234879 | 0.017825 | 0.002583 | 0.191116 | -0.01943 | -0.26502 | 0.104785 | LOC653786 |
| ILMN_1740900 | 0.039624 | 0.008305 | -0.09608 | -0.06297 | -0.05862 | -0.8145 | BMP4 |
| ILMN_1723177 | 0.001859 | 1.87E-05 | -0.27165 | -0.34801 | 0.260982 | -1.083 | ANAPC5 |
| ILMN_1791376 | 0.015265 | 0.002063 | 0.288967 | 0.083099 | -0.26766 | 0.216444 | C15orf43 |
| ILMN_2228453 | 0.011974 | 0.0014 | 0.036075 | -0.77654 | 0.222796 | 0.053692 | KIAA0562 |
| ILMN_2351930 | 0.019776 | 0.002998 | 0.189646 | 0.288022 | -0.17198 | 0.342858 | TRIM33 |
| ILMN_1784447 | 0.028397 | 0.005176 | 0.363604 | -0.26961 | -0.171 | 0.295613 | PLCE1 |
| ILMN_1712707 | 0.020962 | 0.003289 | 0.308852 | -0.14676 | -0.19325 | 0.405958 | ABHD8 |
| ILMN_1773204 | 0.00308 | 6.54E-05 | 0.306187 | 0.069026 | -0.33004 | 0.170134 | CYSLTR2 |
| ILMN_1779778 | 0.010497 | 0.001121 | 0.789209 | 0.01086 | -0.23672 | 0.233971 | PRR19 |
| ILMN_1715065 | 0.041577 | 0.008918 | -0.14928 | 0.392989 | -0.32895 | -0.07813 | ZNF580 |
| ILMN_1712914 | 0.004191 | 1.76E-04 | 0.228125 | 0.382863 | -0.56254 | -0.31093 | SEMA6B |
| ILMN_1679809 | 0.006588 | 5.00E-04 | 0.313262 | -0.0011 | -0.17745 | -0.16093 | GSTP1 |
| ILMN_1696935 | 0.020595 | 0.003208 | 0.157046 | 0.021723 | 0.583176 | -0.57524 | RBM39 |
| ILMN_2258004 | 0.040472 | 0.008559 | -0.06656 | 0.159625 | -0.22957 | 0.601818 | METRNL |
| ILMN_1806533 | 0.021241 | 0.003351 | -0.14637 | -0.33099 | 0.205786 | -1.06517 | PDE7B |
| ILMN_1733757 | 0.003028 | 6.25E-05 | 0.310526 | 0.016221 | -0.28042 | 0.094077 | LOC374395 |
| ILMN_1729487 | 0.039815 | 0.008354 | 0.608388 | -0.35325 | 0.037419 | -0.31446 | GMPR |
| ILMN_1775708 | 0.024194 | 0.004073 | 0.07106 | 0.224147 | -0.14177 | 1.069978 | SLC2A3 |
| ILMN_2404065 | 0.004442 | 2.05E-04 | 0.354117 | -0.30875 | -0.01439 | -0.24176 | APP |
| ILMN_2360784 | 0.00719 | 5.92E-04 | 0.309465 | 0.076133 | -0.29559 | -0.08507 | RRBP1 |
| ILMN_2234310 | 0.022067 | 0.003559 | 0.45639 | -0.75542 | 0.151705 | -0.82841 | GLTPD1 |
| ILMN_1766204 | 0.006173 | 4.30E-04 | 0.501203 | 0.067967 | -0.32545 | -0.02929 | GPHA2 |
| ILMN_1680814 | 0.035257 | 0.007064 | 0.67759 | 0.448877 | -0.18165 | 0.169905 | EDN2 |
| ILMN_1675819 | 0.005836 | 3.87E-04 | 0.410575 | 0.276672 | -0.17127 | 0.203383 | FAM101A |
| ILMN_1672135 | 0.025118 | 0.004312 | -0.33238 | 0.145217 | 0.012136 | -0.25072 | ZNF615 |
| ILMN_1742618 | 0.047742 | 0.010901 | 0.192144 | 0.069547 | -0.23618 | -0.11199 | XAF1 |
| ILMN_1693891 | 0.008858 | 8.54E-04 | 0.215668 | 0.026633 | -0.28317 | 0.098955 | MOGAT2 |
| ILMN_1778321 | 0.030341 | 0.005708 | 0.2925 | 0.143008 | -0.46087 | -0.24385 | SLC2A6 |
| ILMN_1660544 | 0.033614 | 0.006604 | 0.065412 | -0.38601 | -0.00412 | 0.29024 | ARRDC4 |
| ILMN_1736585 | 0.01063 | 0.001146 | -0.00259 | -0.3051 | 0.037822 | 0.293692 | TMED10 |
| ILMN_1687749 | 0.014283 | 0.001844 | 0.140123 | 0.012877 | -0.18443 | 0.054142 | TFEC |
| ILMN_1689552 | 0.010118 | 0.001054 | 0.093581 | 0.158719 | -0.00911 | -0.66097 | FAM63A |
| ILMN_2408415 | 0.017467 | 0.002509 | 0.268762 | 0.042643 | -0.18096 | -0.14129 | RPL9 |
| ILMN_1752145 | 0.006471 | 4.79E-04 | 0.185962 | -0.0059 | -0.23838 | 0.245588 | ERAP1 |
| ILMN_2166275 | 0.006018 | 4.11E-04 | 0.343879 | -0.03473 | -0.3057 | 0.148987 | NOTUM |
| ILMN_1752837 | 0.006321 | 4.52E-04 | -0.2796 | -0.54623 | 0.430683 | -0.56585 | ARL8B |
| ILMN_1812570 | 0.027303 | 0.00487 | 0.164543 | -0.19939 | -0.67303 | 0.750604 | SHC1 |
| ILMN_1750250 | 0.015996 | 0.002204 | 0.446059 | 0.098434 | -0.03601 | 0.057796 | ABCA12 |
| ILMN_1742917 | 0.021421 | 0.003394 | 0.582875 | 0.11875 | -0.25663 | 0.544447 | NXNL1 |
| ILMN_1750052 | 0.008966 | 8.69E-04 | 0.120374 | -0.01376 | -0.16134 | 0.265811 | NOP14 |
| ILMN_3236358 | 0.038076 | 0.007833 | 0.288267 | -0.02874 | -0.1891 | -0.08068 | NOP14 |
| ILMN_2226324 | 0.00256 | 4.03E-05 | 0.327519 | -0.13883 | -0.23612 | -0.03756 | BRP44L |
| ILMN_1666967 | 0.044655 | 0.009878 | 0.3074 | -0.13762 | -0.17367 | -0.0138 | BRP44L |
| ILMN_1778237 | 0.00523 | 3.07E-04 | 0.307969 | 0.131482 | -0.36854 | -0.23648 | FN1 |
| ILMN_1800142 | 0.026742 | 0.00473 | 0.494823 | 0.254606 | -0.17025 | 0.272348 | LAMB3 |
| ILMN_2258749 | 0.015512 | 0.002109 | 0.067621 | -0.1797 | 0.111028 | 1.454532 | BAIAP2 |
| ILMN_2293374 | 0.044728 | 0.009908 | 0.138891 | 0.206752 | 0.043287 | -0.88717 | TOP1MT |
| ILMN_2094776 | 0.003614 | 1.12E-04 | -0.06895 | -0.1247 | -0.09995 | 0.495716 | CCNL1 |
| ILMN_1746837 | 0.01816 | 0.002655 | 0.380524 | 0.011256 | -0.25755 | 0.044643 | UPP1 |
| ILMN_1798256 | 0.030835 | 0.005848 | 0.180424 | 0.160629 | -0.23427 | 0.190676 | UPP1 |
| ILMN_2336609 | 0.007273 | 6.05E-04 | -0.01862 | 0.232558 | -0.05886 | -0.57462 | SYTL2 |
| ILMN_1659856 | 0.027191 | 0.004836 | 0.432948 | 0.00795 | -0.13661 | -0.05527 | FAM167B |
| ILMN_1672378 | 0.001984 | 2.16E-05 | 0.467285 | 0.108224 | -0.35181 | 0.205281 | ZP3 |
| ILMN_1753008 | 0.002536 | 3.87E-05 | 0.229742 | 0.102982 | -0.42006 | 0.145863 | REXO1 |
| ILMN_2344047 | 0.027766 | 0.004996 | 0.308409 | -0.03031 | -0.16646 | 0.010833 | ZNF398 |
| ILMN_1737599 | 0.002067 | 2.44E-05 | 0.470033 | 0.076692 | -0.17932 | 0.37112 | TRIM5 |
| ILMN_3239103 | 0.003175 | 7.14E-05 | 0.352862 | 0.070891 | -0.35027 | 0.048041 | LOC340357 |
| ILMN_1686862 | 0.01396 | 0.001783 | 0.636401 | -0.24387 | -0.3488 | 0.28158 | HLX |
| ILMN_3236437 | 0.006666 | 5.15E-04 | 0.37868 | -0.02184 | -0.29587 | 0.15095 | GDEP |
| ILMN_1795429 | 0.009233 | 9.09E-04 | 0.33948 | 0.121318 | -0.13531 | 0.026503 | VCL |
| ILMN_3301206 | 0.006328 | 4.54E-04 | 0.508818 | 0.630592 | -0.20448 | 0.141698 | MAGIX |
| ILMN_2059606 | 0.013166 | 0.001625 | 0.327283 | 0.059568 | -0.16096 | 0.002351 | CCDC117 |
| ILMN_1694075 | 0.00725 | 5.99E-04 | 0.400379 | -0.31683 | -0.01451 | 0.745507 | GADD45A |
| ILMN_2251225 | 0.00719 | 5.91E-04 | 0.459121 | -0.0648 | -0.17903 | -0.33119 | WDR78 |
| ILMN_1800540 | 0.004131 | 1.67E-04 | 0.195892 | 0.039987 | -0.37746 | 0.0232 | CD55 |
| ILMN_3236404 | 0.033736 | 0.006642 | 0.593705 | 0.127822 | -0.49 | 0.519596 | SNORD7 |
| ILMN_2131756 | 0.046894 | 0.010604 | 0.239991 | -0.01883 | -0.19289 | 0.024719 | LRRC40 |
| ILMN_2062001 | 0.020749 | 0.003248 | -0.34455 | -0.27296 | 0.250468 | 0.096945 | HOOK3 |
| ILMN_2123312 | 0.010085 | 0.001046 | -0.35413 | 0.121762 | 0.529156 | -0.57934 | LRAP |
| ILMN_1753950 | 0.019161 | 0.002867 | 0.369399 | -0.0132 | -0.1877 | -0.14022 | ZNF326 |
| ILMN_2048700 | 0.040552 | 0.008586 | 0.307734 | -0.38309 | -0.11983 | 0.492144 | ATAD2 |
| ILMN_1719163 | 0.018156 | 0.002651 | 0.199215 | 0.294373 | -0.32105 | -0.06971 | ZNF557 |
| ILMN_1722670 | 0.036762 | 0.007479 | 0.247034 | 0.063227 | -0.22118 | 0.11725 | LYNX1 |
| ILMN_2351269 | 0.046771 | 0.010565 | 2.203272 | 1.845907 | 0.018818 | -0.21021 | TTYH1 |
| ILMN_2227133 | 0.004063 | 1.56E-04 | 0.733506 | 0.166118 | -0.20805 | 0.19815 | KIAA0082 |
| ILMN_3244276 | 0.004145 | 1.69E-04 | 0.432817 | 0.016345 | -0.30475 | 0.176964 | LOC415056 |
| ILMN_1776666 | 0.003258 | 7.57E-05 | 0.29444 | 0.005348 | -0.33827 | -0.04481 | GRIK3 |
| ILMN_2395496 | 0.003349 | 8.71E-05 | 0.110337 | -0.02862 | 0.340093 | -1.38515 | KLK7 |
| ILMN_1738725 | 0.021809 | 0.003486 | -0.2123 | 0.462358 | -0.36932 | 1.163304 | LIF |
| ILMN_2212999 | 0.010355 | 0.001098 | 0.329516 | 0.251051 | -0.16473 | -0.26308 | KIF5C |
| ILMN_1658221 | 0.00792 | 6.96E-04 | 0.306863 | 0.054771 | -0.14009 | -0.06555 | POLH |
| ILMN_2136576 | 0.013578 | 0.001704 | 0.379769 | 0.036941 | -0.2716 | -0.04709 | POLH |
| ILMN_1796537 | 0.014793 | 0.001951 | -0.29114 | 0.772937 | -0.21947 | -0.48479 | FYB |
| ILMN_1808333 | 0.014935 | 0.001979 | 0.11977 | -0.61022 | -0.09464 | 0.506812 | PPP1R7 |
| ILMN_2399310 | 0.042576 | 0.009209 | 0.048761 | -0.00892 | 0.447349 | -0.28742 | MLLT10 |
| ILMN_2189314 | 0.039964 | 0.008391 | 0.322376 | 0.474392 | -0.20363 | 0.304425 | HDGFL1 |
| ILMN_1666845 | 0.03152 | 0.006032 | 0.413835 | 0.229755 | -0.23884 | 0.549104 | KRT17 |
| ILMN_2310896 | 0.026574 | 0.004683 | 0.720419 | 0.14513 | -0.50196 | 0.070098 | NLRP3 |
| ILMN_3307940 | 0.012217 | 0.001443 | -0.48447 | 0.229294 | -0.09815 | 0.273759 | FLJ35776 |
| ILMN_1694133 | 0.008076 | 7.19E-04 | 0.635814 | 0.311673 | -0.24688 | 0.252435 | ADD2 |
| ILMN_1760174 | 0.006725 | 5.25E-04 | 0.239836 | -0.15933 | -0.1604 | -0.01959 | MCCC1 |
| ILMN_1782095 | 0.003963 | 1.44E-04 | -0.52737 | -0.85018 | 0.321654 | -1.46504 | C20orf55 |
| ILMN_2161286 | 0.045751 | 0.010235 | 0.188462 | 0.582276 | -0.02285 | -0.5076 | FAM40B |
| ILMN_1689965 | 0.045122 | 0.010045 | -0.04941 | -0.55504 | 0.344168 | -0.66535 | KIAA1432 |
| ILMN_2369826 | 0.025673 | 0.004455 | 0.228972 | -0.02604 | -0.03262 | -0.35994 | C16orf13 |
| ILMN_1729515 | 0.041092 | 0.00877 | -0.16294 | 0.311658 | 0.191728 | -0.59069 | PIN4 |
| ILMN_1675677 | 0.003027 | 6.19E-05 | 0.328644 | -0.06602 | -0.28265 | -0.02863 | TMPRSS3 |
| ILMN_1730391 | 0.015919 | 0.002187 | 0.315521 | 0.047868 | -0.29367 | 0.017175 | MRPS18A |
| ILMN_1794095 | 0.00538 | 3.25E-04 | 0.337497 | 0.008624 | -0.29298 | 0.135845 | HTR1E |
| ILMN_1715068 | 0.013777 | 0.001741 | 0.342563 | -0.15897 | -0.39969 | 0.072235 | AQP9 |
| ILMN_1793630 | 0.015434 | 0.002094 | 0.817983 | 0.017182 | -0.23109 | -0.26037 | SLC4A3 |
| ILMN_1757736 | 0.036407 | 0.007387 | 0.126284 | -0.74804 | -0.0213 | -1.3397 | IRX5 |
| ILMN_3310805 | 0.006356 | 4.60E-04 | 0.616054 | 0.182006 | -0.19271 | 0.228556 | MIR625 |
| ILMN_1800451 | 0.002418 | 3.40E-05 | 0.189781 | -0.03188 | -0.33356 | 0.209388 | MED16 |
| ILMN_1730355 | 0.003791 | 1.27E-04 | -0.0913 | -0.81795 | 0.285175 | -0.39684 | FGFR4 |
| ILMN_2325506 | 0.043253 | 0.009423 | 0.264513 | 0.084457 | -0.27577 | 0.070334 | BCAS4 |
| ILMN_2248912 | 0.023842 | 0.003994 | -0.59205 | -0.03642 | 0.25934 | -0.7072 | BCAS4 |
| ILMN_1680726 | 0.002707 | 4.55E-05 | 0.437587 | -0.12903 | -0.25236 | -0.03641 | SYTL5 |
| ILMN_2154566 | 0.017551 | 0.002533 | 0.155893 | 0.210841 | -0.15953 | -0.02678 | RPL10A |
| ILMN_1731043 | 0.006404 | 4.68E-04 | -0.50391 | 0.200798 | 0.074397 | -0.39181 | TRA2A |
| ILMN_2251375 | 0.047525 | 0.010829 | 0.207129 | 0.090259 | -0.31098 | -0.0482 | ZFP64 |
| ILMN_2144352 | 0.006004 | 4.09E-04 | 0.333786 | -0.02744 | -0.33899 | 0.131628 | LOC441251 |
| ILMN_1712766 | 0.020466 | 0.003184 | -0.2439 | -0.41228 | 0.037122 | -0.35831 | ERGIC2 |
| ILMN_2234016 | 0.011588 | 0.00133 | 0.323808 | 0.032553 | -0.28618 | 0.085886 | FTHL7 |
| ILMN_1686557 | 0.005447 | 3.38E-04 | 0.449829 | 0.01083 | -0.28344 | 0.288944 | TIMM13 |
| ILMN_1702943 | 0.017483 | 0.002515 | 0.254467 | 0.083642 | -0.15862 | 0.142752 | DLK2 |
| ILMN_1663042 | 0.047028 | 0.010661 | -0.68696 | -0.19559 | 0.287956 | -0.15931 | SDC4 |
| ILMN_1732328 | 0.012534 | 0.001504 | 0.274176 | -0.01471 | -0.14325 | 0.150525 | LOC646200 |
| ILMN_2268156 | 0.010297 | 0.001084 | 0.349347 | -0.13923 | -0.33765 | 0.260502 | LFNG |
| ILMN_3247882 | 0.004238 | 1.84E-04 | 0.276866 | 0.047133 | -0.38319 | -0.01142 | ERI3 |
| ILMN_1688526 | 0.042875 | 0.009302 | 0.167664 | 0.049525 | -0.2833 | -0.01531 | ARL5A |
| ILMN_1712975 | 0.017148 | 0.002441 | 0.153138 | -0.11683 | -0.24956 | 0.183072 | YIF1A |
| ILMN_2404917 | 0.040796 | 0.00867 | 0.296011 | -0.31276 | 0.217948 | -0.17728 | AFAP1L2 |
| ILMN_1759732 | 0.047095 | 0.010687 | 0.21264 | -0.82609 | -0.21535 | 0.541789 | RFNG |
| ILMN_1802603 | 0.009708 | 9.80E-04 | -0.18237 | -0.17023 | 0.024078 | -0.49733 | RFNG |
| ILMN_2358277 | 0.029049 | 0.00536 | 0.126612 | 0.103384 | -0.24273 | 0.01085 | RSRC2 |
| ILMN_1694385 | 0.011494 | 0.001307 | 0.135352 | -0.04049 | -0.21797 | -0.01243 | YWHAB |
| ILMN_1678255 | 0.006011 | 4.10E-04 | 0.330898 | -0.04234 | -0.19983 | 0.101877 | ALOXE3 |
| ILMN_1703474 | 0.016511 | 0.002303 | -0.19464 | -0.1281 | 0.132994 | -0.48241 | POLK |
| ILMN_2157075 | 0.001835 | 1.74E-05 | 0.158418 | 0.186145 | -0.44146 | 0.250245 | LRCH4 |
| ILMN_3243667 | 0.048448 | 0.011143 | 0.5652 | -0.37069 | -0.41472 | 0.552548 | SNORA71B |
| ILMN_1742731 | 0.005956 | 4.02E-04 | 0.429263 | 0.054494 | -0.20081 | 0.132571 | SLC35A2 |
| ILMN_1703949 | 0.031739 | 0.006098 | 0.215025 | -0.05185 | -0.18582 | -0.01869 | KPNB1 |
| ILMN_1709815 | 0.00621 | 4.36E-04 | 0.505843 | 0.127887 | -0.26131 | 0.181017 | PURG |
| ILMN_2338116 | 0.016056 | 0.002217 | 0.385604 | -0.00939 | -0.22278 | 0.122005 | TRAF5 |
| ILMN_2162298 | 0.003417 | 9.44E-05 | 0.399579 | -0.14543 | -0.1264 | 0.361264 | DYSFIP1 |
| ILMN_1813260 | 0.003614 | 1.12E-04 | 0.228986 | 0.001747 | -0.28697 | 0.171686 | TIMM17B |
| ILMN_1706758 | 0.011494 | 0.001308 | 0.5579 | 0.106674 | -0.2254 | 0.247489 | SEC24C |
| ILMN_1672403 | 0.004543 | 2.17E-04 | 0.564137 | 0.10357 | -0.23784 | 0.134825 | ADAMTS8 |
| ILMN_1751258 | 0.035772 | 0.007197 | 0.297426 | -0.22692 | -0.11961 | -0.03298 | NDUFA4 |
| ILMN_1739734 | 0.01951 | 0.002932 | 0.035276 | -0.11396 | -0.3967 | 0.219228 | CLTCL1 |
| ILMN_2327795 | 0.02463 | 0.00418 | 0.148172 | -0.1841 | -0.14377 | 0.262465 | RERE |
| ILMN_1669409 | 0.009869 | 0.00101 | 0.250583 | -0.08228 | -0.26773 | 0.00324 | VSIG4 |
| ILMN_1692895 | 0.005087 | 2.81E-04 | 0.293473 | 0.004032 | -0.23475 | 0.227132 | PIK3R3 |
| ILMN_2305112 | 0.006471 | 4.79E-04 | -0.22687 | -0.06215 | 0.114351 | 0.669803 | CTH |
| ILMN_1803995 | 0.029035 | 0.005356 | 0.053437 | -0.07446 | -0.11503 | 0.408181 | TM7SF3 |
| ILMN_1787514 | 0.042015 | 0.00906 | 0.290832 | 0.095348 | -0.38225 | -0.12784 | CAPN12 |
| ILMN_1656399 | 0.045958 | 0.010301 | 0.141807 | -0.20218 | 0.010318 | -0.48115 | TCEAL8 |
| ILMN_1796629 | 0.01202 | 0.001408 | 0.188471 | 0.208847 | -0.15078 | -0.14509 | EDNRA |
| ILMN_1714158 | 0.014292 | 0.001851 | -0.5748 | -0.1089 | 0.155237 | -0.66808 | PON2 |
| ILMN_2189859 | 0.024496 | 0.004135 | -0.31864 | -0.22303 | 0.327808 | -0.09139 | FLJ38482 |
| ILMN_1814797 | 0.014688 | 0.001929 | 0.250094 | 0.08668 | -0.21343 | 0.075942 | SLC35F3 |
| ILMN_2284327 | 0.028248 | 0.005135 | 0.151213 | -0.12523 | -0.27853 | 0.405341 | WDR23 |
| ILMN_1815148 | 0.003317 | 8.46E-05 | -0.08081 | 0.279383 | -0.52825 | 0.032732 | MAN2A2 |
| ILMN_1761850 | 0.006485 | 4.83E-04 | 0.23853 | -0.09377 | -0.26941 | 0.116738 | ATRN |
| ILMN_1772124 | 0.024931 | 0.004265 | 0.106959 | 0.01219 | -0.31 | -0.01235 | ATRN |
| ILMN_1743621 | 0.049313 | 0.011416 | 0.334048 | 0.310326 | -0.14445 | -0.51751 | C17orf69 |
| ILMN_1784717 | 0.015011 | 0.001991 | 0.281 | 0.059341 | -0.25792 | 0.030614 | RPS19 |
| ILMN_1751984 | 0.04864 | 0.011194 | 0.058749 | -0.55003 | 0.014317 | 0.104224 | PRKAG1 |
| ILMN_1694587 | 0.001337 | 9.19E-06 | 0.324508 | -0.0818 | -0.34786 | 0.314464 | EEF1B2 |
| ILMN_2150797 | 0.047362 | 0.010783 | 0.432419 | 0.124638 | -0.20907 | 0.083927 | TNP1 |
| ILMN_2121408 | 0.018123 | 0.002642 | -0.13335 | 0.200378 | -0.52097 | 1.286115 | HBEGF |
| ILMN_1673867 | 0.028517 | 0.005207 | -0.21853 | 0.939285 | -0.59228 | -0.39684 | LIMD2 |
| ILMN_3309554 | 0.025055 | 0.004296 | 0.275673 | 0.134088 | -0.25029 | -0.04094 | MIR632 |
| ILMN_1707312 | 0.002998 | 5.98E-05 | 0.072801 | -0.04874 | -0.11331 | 0.793127 | NFIL3 |
| ILMN_1796431 | 0.002779 | 4.89E-05 | 0.284344 | 0.070348 | -0.2347 | 0.209563 | GPR101 |
| ILMN_1750563 | 0.036405 | 0.007383 | 0.32696 | 0.03535 | -0.28132 | 0.084194 | CERCAM |
| ILMN_1656300 | 0.011611 | 0.001335 | 0.161242 | 0.233846 | -0.50774 | 0.120589 | GFRA2 |
| ILMN_1700611 | 0.006207 | 4.35E-04 | 0.13001 | 0.160182 | -0.35546 | 0.183316 | CDK10 |
| ILMN_1694731 | 0.010118 | 0.001056 | 0.23889 | 0.054636 | -0.31143 | 0.032272 | CLCN7 |
| ILMN_1660806 | 0.022715 | 0.003728 | 0.337402 | -0.08693 | -0.11151 | -0.04618 | CSRP2 |
| ILMN_1804339 | 0.002067 | 2.44E-05 | 0.115062 | -0.05346 | -0.22633 | 0.137963 | CAMK1G |
| ILMN_1813796 | 0.005182 | 2.99E-04 | -0.1969 | 0.226894 | 0.42699 | -1.08329 | TMEM169 |
| ILMN_2214197 | 0.034085 | 0.006737 | -0.35478 | -0.08021 | 0.105662 | -0.0034 | TP53INP1 |
| ILMN_2147993 | 0.009758 | 9.89E-04 | -0.14999 | 0.459269 | 0.220981 | -0.31795 | ZNF23 |
| ILMN_1790461 | 0.007887 | 6.90E-04 | 0.310733 | -0.866 | -0.06357 | -0.05001 | C6orf125 |
| ILMN_2194106 | 0.041074 | 0.008762 | -0.02146 | -0.08498 | 0.360101 | -0.53245 | TSPAN12 |
| ILMN_2403542 | 0.019833 | 0.003009 | 0.072158 | 0.09276 | -0.25715 | 0.166346 | CATSPER2 |
| ILMN_1815118 | 0.023281 | 0.003866 | -0.90928 | 0.114736 | 0.261393 | -0.87443 | ZNF554 |
| ILMN_2120022 | 0.025777 | 0.004486 | 0.058396 | -0.32704 | -0.03166 | 0.425756 | ARL5B |
| ILMN_1667022 | 0.011963 | 0.001398 | 0.719142 | 0.003998 | -0.42205 | 0.123676 | PASK |
| ILMN_2388517 | 0.010153 | 0.001061 | 0.074403 | 0.20164 | 0.137309 | -0.58066 | MTERFD3 |
| ILMN_2075507 | 0.006404 | 4.70E-04 | -0.45935 | -0.14531 | 0.112576 | -0.5296 | MAN1A2 |
| ILMN_1783881 | 0.045596 | 0.010185 | 0.579333 | -0.09823 | -0.10652 | 0.279511 | ZIC5 |
| ILMN_2164482 | 0.025594 | 0.004437 | 0.257842 | -0.02452 | -0.14338 | 0.103091 | TTTY5 |
| ILMN_1743643 | 0.025924 | 0.004536 | 0.116762 | 0.024003 | -0.22658 | -0.00403 | ZXDC |
| ILMN_1753101 | 0.04229 | 0.009133 | -0.15909 | 0.025529 | 0.183213 | -0.79691 | VTCN1 |
| ILMN_1681154 | 0.025166 | 0.004331 | -0.38138 | -0.55778 | 0.282989 | -0.28785 | ZBTB39 |
| ILMN_2124064 | 0.036915 | 0.007514 | -0.06026 | -0.07167 | -0.1514 | 0.657439 | REL |
| ILMN_1766085 | 0.0051 | 2.85E-04 | -0.10635 | 0.108182 | -0.11484 | 0.845643 | REL |
| ILMN_1755502 | 0.028652 | 0.005246 | 0.568034 | -0.60472 | -0.13157 | 0.945233 | USP2 |
| ILMN_1756352 | 0.002863 | 5.38E-05 | 0.522361 | -0.09321 | -0.14971 | 0.510149 | MAPBPIP |
| ILMN_2279144 | 0.006311 | 4.51E-04 | 0.404664 | -0.05289 | -0.21806 | 0.240429 | PHF16 |
| ILMN_2133360 | 0.010631 | 0.001146 | 0.361031 | 0.088118 | -0.23577 | -0.03818 | LOC91561 |
| ILMN_1806937 | 5.01E-04 | 1.56E-06 | -0.07462 | -0.1209 | 0.513179 | -0.69163 | C19orf62 |
| ILMN_1709247 | 0.005042 | 2.75E-04 | 0.187218 | 0.334033 | -0.33742 | 1.43917 | PPP2R1B |
| ILMN_1692865 | 0.021589 | 0.003433 | 0.149692 | 0.264811 | 0.024082 | -0.4992 | VPS37D |
| ILMN_2067980 | 0.01969 | 0.002976 | 0.20298 | -0.09673 | -0.26183 | -0.02992 | NKAP |
| ILMN_1758272 | 0.049851 | 0.011574 | 0.403814 | -0.03444 | -0.22578 | 0.109418 | MYPN |
| ILMN_1706805 | 0.049851 | 0.011573 | 0.990673 | 0.404728 | -0.06027 | 0.172691 | TRPM5 |
| ILMN_1750180 | 0.004851 | 2.46E-04 | 0.705369 | -0.01901 | -0.2356 | 0.194145 | HIST1H2BB |
| ILMN_1656625 | 0.039643 | 0.008313 | 0.131257 | 0.18308 | -0.07262 | -0.29541 | RPS24 |
| ILMN_1674838 | 0.026653 | 0.004702 | 0.342021 | 0.187254 | -0.28768 | 0.257147 | ZAP70 |
| ILMN_1738742 | 0.011963 | 0.001398 | 0.346939 | -0.10588 | -0.12854 | 0.184043 | PLAT |
| ILMN_2409078 | 0.029752 | 0.005552 | 0.254675 | 0.021518 | -0.35766 | 0.060071 | SNHG10 |
| ILMN_3238832 | 0.002007 | 2.27E-05 | 0.488468 | -0.02551 | -0.2441 | 0.238658 | RBMY3AP |
| ILMN_1661170 | 0.005988 | 4.06E-04 | 0.30073 | -0.02775 | -0.13637 | 0.155697 | NDUFB8 |
| ILMN_1746924 | 0.045322 | 0.010105 | 0.421364 | 0.030609 | -0.11399 | -0.04064 | ZNF610 |
| ILMN_1701497 | 0.025706 | 0.004465 | 0.386152 | -0.03753 | -0.25568 | 0.221202 | CD5L |
| ILMN_3299047 | 0.004492 | 2.11E-04 | 0.417229 | -0.03481 | -0.33262 | 0.045072 | XAGE1C |
| ILMN_2412141 | 0.007999 | 7.08E-04 | 0.55738 | 0.041678 | -0.20571 | 0.172961 | TAAR2 |
| ILMN_1703538 | 0.049774 | 0.011546 | 0.133472 | -0.08846 | -0.40469 | 0.245477 | AIF1 |
| ILMN_1705384 | 0.018689 | 0.002766 | -0.03213 | 0.031782 | -0.04983 | -0.59196 | PPFIBP1 |
| ILMN_1662839 | 0.03621 | 0.007328 | -0.43667 | 1.31E-04 | 0.198512 | -0.40106 | PLEKHA1 |
| ILMN_1712705 | 0.008142 | 7.28E-04 | 0.221657 | 0.037019 | -0.24606 | -0.12394 | RAB40C |
| ILMN_1753805 | 0.045946 | 0.010294 | 0.241574 | -0.23431 | -0.38521 | -0.06811 | PRKD2 |
| ILMN_2330371 | 0.012526 | 0.001503 | 0.163753 | -0.06887 | -0.21729 | 0.116568 | TATDN3 |
| ILMN_2347789 | 0.011252 | 0.001261 | 0.245079 | 0.087277 | -0.21512 | 0.188412 | C4BPB |
| ILMN_2282366 | 0.020602 | 0.00321 | 0.153296 | 0.217313 | -0.24714 | 0.710525 | IQSEC3 |
| ILMN_1801043 | 0.008208 | 7.40E-04 | 0.321273 | -0.33028 | -0.14592 | 0.283955 | GSN |
| ILMN_1684158 | 0.048886 | 0.011285 | 0.337679 | -0.09278 | -0.12486 | 0.141717 | GPT2 |
| ILMN_1743583 | 0.048672 | 0.011208 | 0.174667 | 0.134693 | -0.15288 | -0.12718 | MADD |
| ILMN_1784238 | 0.004357 | 1.98E-04 | -0.10783 | -0.17434 | 0.336393 | -0.62003 | SEC22B |
| ILMN_1674250 | 0.02463 | 0.004176 | -0.77742 | 0.522073 | -0.23357 | -0.46084 | NCKAP1L |
| ILMN_1709799 | 0.003584 | 1.08E-04 | 0.287028 | 0.057407 | -0.31791 | 0.085166 | C6orf25 |
| ILMN_1749960 | 0.006193 | 4.34E-04 | 0.993525 | 0.18436 | -0.0879 | 0.205492 | FAM78B |
| ILMN_1774749 | 0.005206 | 3.04E-04 | 0.370657 | 0.058066 | -0.14422 | 0.267101 | SSTR3 |
| ILMN_1764885 | 0.041902 | 0.009034 | 0.871793 | 0.025118 | -0.61247 | 0.089347 | C20orf75 |
| ILMN_1719204 | 0.041715 | 0.008978 | 0.233616 | -0.40623 | 0.23174 | -0.42193 | PRPF31 |
| ILMN_1733559 | 0.017135 | 0.002438 | 0.375423 | 0.099283 | -0.27262 | -0.06461 | LOC100008589 |
| ILMN_3251587 | 0.004671 | 2.31E-04 | 0.446106 | -0.00551 | -0.59121 | 0.117091 | LOC100008589 |
| ILMN_2045453 | 0.014638 | 0.00192 | 0.393993 | -0.01075 | -0.33086 | 0.280728 | CFHR2 |
| ILMN_1710416 | 0.004464 | 2.07E-04 | 0.080824 | 0.096898 | -0.21158 | 0.224364 | HS3ST6 |
| ILMN_2386444 | 0.001086 | 5.79E-06 | 0.363733 | -0.09625 | -0.35208 | 0.125914 | ANGPTL4 |
| ILMN_2297315 | 0.005129 | 2.90E-04 | 0.652327 | 0.130346 | -0.24768 | 0.364755 | CDRT1 |
| ILMN_1811362 | 0.002102 | 2.54E-05 | 0.668147 | 0.087357 | -0.21856 | 0.155807 | LOC653746 |
| ILMN_2217056 | 0.008858 | 8.53E-04 | 0.258048 | 0.077937 | -0.39078 | -0.06653 | BPY2B |
| ILMN_2413898 | 0.006498 | 4.86E-04 | 0.438383 | -0.23606 | -0.30008 | -0.14368 | MCM10 |
| ILMN_1751095 | 0.005629 | 3.60E-04 | 0.713201 | 0.058212 | -0.1402 | 0.291876 | CD300E |
| ILMN_1664276 | 0.002586 | 4.14E-05 | 0.779519 | 0.047736 | -0.1821 | 0.153712 | TBX22 |
| ILMN_1707828 | 0.01952 | 0.002936 | -0.34526 | -0.08102 | 0.464215 | -0.40637 | UGT1A5 |
| ILMN_1706593 | 0.03152 | 0.006033 | -0.14212 | -0.25541 | 0.22229 | -0.34958 | PPHLN1 |
| ILMN_1685796 | 0.023422 | 0.003903 | 0.408893 | -0.01887 | -0.05507 | 0.053957 | CSDE1 |
| ILMN_1778357 | 0.042183 | 0.009104 | 0.452353 | 0.082215 | -0.19598 | 0.495845 | DNMT3L |
| ILMN_3310336 | 0.004255 | 1.87E-04 | 0.477626 | 0.003591 | -0.27363 | 0.072438 | MIR450A1 |
| ILMN_3246673 | 0.008541 | 7.91E-04 | 0.355818 | 0.027406 | -0.28512 | 0.20401 | AMAC1L3 |
| ILMN_2398432 | 0.011827 | 0.001373 | 0.330324 | 0.121749 | -0.12737 | 0.166878 | BRMS1 |
| ILMN_2132898 | 0.011239 | 0.001258 | 0.291889 | -0.01363 | -0.27138 | 0.087938 | SPRN |
| ILMN_1703573 | 0.014546 | 0.001902 | 0.233908 | 0.114705 | -0.20195 | 0.162026 | DNAJC17 |
| ILMN_1715482 | 0.047647 | 0.010868 | -0.16885 | -0.04897 | 0.101381 | -0.5176 | ULK2 |
| ILMN_1739450 | 0.04327 | 0.009429 | 0.069257 | -0.44763 | -0.02724 | -0.01968 | NFE2L1 |
| ILMN_1750469 | 0.047768 | 0.010912 | 0.742752 | 0.017154 | -0.179 | 0.541003 | UCN3 |
| ILMN_1776598 | 0.004041 | 1.53E-04 | 0.455661 | -0.11092 | -0.31567 | 0.150606 | METTL11A |
| ILMN_1779010 | 0.014137 | 0.001818 | 0.043788 | 0.007112 | -0.16845 | 0.532223 | MAP3K3 |
| ILMN_2410771 | 0.020411 | 0.00317 | 0.289411 | -0.23504 | -0.20594 | 0.144348 | KEAP1 |
| ILMN_2374159 | 0.003387 | 8.91E-05 | 0.094409 | -0.11822 | -0.12159 | 0.402773 | HERPUD1 |
| ILMN_1784515 | 0.032833 | 0.006384 | 0.418112 | 0.023687 | -0.11859 | 0.007931 | MYH6 |
| ILMN_2208802 | 0.009514 | 9.54E-04 | 0.049454 | 0.254832 | -0.15158 | -0.16553 | NPIP |
| ILMN_1750624 | 0.004944 | 2.59E-04 | 0.228439 | -0.10119 | -0.43084 | -0.02864 | RXRG |
| ILMN_2193553 | 0.04435 | 0.009794 | 0.571032 | 0.287946 | -0.12174 | 0.99327 | SLC7A5P1 |
| ILMN_1795055 | 0.00898 | 8.73E-04 | 0.368114 | 0.005098 | -0.2687 | 0.120828 | LRRC3 |
| ILMN_2275098 | 0.006011 | 4.10E-04 | 0.27419 | 0.194659 | -0.25752 | 0.042547 | DTX2 |
| ILMN_2354391 | 0.018238 | 0.002673 | 0.153494 | 0.262956 | 0.072519 | -0.36774 | EGLN2 |
| ILMN_2092441 | 0.013994 | 0.00179 | 0.219512 | -0.05191 | -0.27955 | 0.118794 | LRP12 |
| ILMN_2383150 | 0.005009 | 2.70E-04 | 0.297664 | 0.034365 | -0.21242 | 0.142181 | MCHR2 |
| ILMN_2159730 | 0.01083 | 0.001184 | 0.435388 | -0.22129 | 0.637039 | -0.01151 | GABRB1 |
| ILMN_1761206 | 0.003568 | 1.04E-04 | 0.481246 | 0.107498 | -0.15318 | 0.109973 | PALMD |
| ILMN_1681067 | 0.0471 | 0.0107 | 0.537399 | 0.269785 | -0.25129 | 0.064557 | PIK3R5 |
| ILMN_3241996 | 0.041577 | 0.008918 | 0.285453 | -0.00569 | -0.22554 | 0.132742 | C6orf59 |
| ILMN_1670748 | 0.031734 | 0.006094 | -0.1903 | 0.292399 | 0.179137 | -0.30948 | GALNT7 |
| ILMN_2405684 | 0.045519 | 0.010154 | 0.383829 | 0.437948 | -0.25283 | 0.072957 | BIRC3 |
| ILMN_1774860 | 0.001687 | 1.47E-05 | 0.261915 | 0.023331 | -0.33656 | 0.120381 | UTP23 |
| ILMN_1767171 | 0.002536 | 3.81E-05 | 0.295022 | -0.05243 | -0.35101 | 0.214617 | MOBKL2C |
| ILMN_2355225 | 0.012878 | 0.001576 | 0.275096 | 0.019027 | -0.23557 | 0.007507 | LSP1 |
| ILMN_1693560 | 0.005009 | 2.71E-04 | 0.330132 | -0.009 | -0.17778 | 0.123236 | HMGA1 |
| ILMN_1713835 | 0.005197 | 3.03E-04 | 0.516844 | -0.08933 | -0.26746 | 0.15331 | ENHO |
| ILMN_1678477 | 0.009429 | 9.39E-04 | -0.00814 | 0.047946 | 0.184887 | -0.36863 | C9orf85 |
| ILMN_1728711 | 0.028204 | 0.005118 | 0.40104 | -0.19188 | -0.40707 | 0.196941 | RGS3 |
| ILMN_1727909 | 0.016725 | 0.002351 | 0.584877 | -0.0587 | -0.15073 | 0.175357 | CNTN5 |
| ILMN_1676305 | 1.95E-06 | 9.19E-10 | 0.560452 | -0.08834 | -0.41154 | 0.131538 | PTK2 |
| ILMN_1705218 | 0.016056 | 0.002217 | 0.125643 | -1.24152 | -0.15816 | 0.535963 | TRAPPC2 |
| ILMN_3193306 | 0.040838 | 0.008687 | 0.187343 | 0.021934 | -0.26166 | -0.00517 | C14orf109 |
| ILMN_2254943 | 0.006328 | 4.53E-04 | 0.257239 | 0.044709 | -0.20098 | 0.218235 | LOC390748 |
| ILMN_1758806 | 1.30E-04 | 1.94E-07 | 0.422029 | 0.219366 | -0.42549 | 0.247387 | C21orf2 |
| ILMN_1776412 | 0.006756 | 5.31E-04 | 0.340083 | 0.119087 | -0.22569 | 0.134005 | KRTAP10-11 |
| ILMN_1669831 | 0.012752 | 0.001553 | -0.18034 | -0.07565 | -0.01545 | -0.72887 | C6orf192 |
| ILMN_1767393 | 0.047871 | 0.010951 | 0.349091 | 0.02766 | -0.16698 | 0.077848 | GRIA3 |
| ILMN_3246869 | 0.010796 | 0.001176 | 0.126576 | -0.0171 | -0.50779 | 0.19617 | SCARNA21 |
| ILMN_1736500 | 0.019863 | 0.003017 | 0.211878 | 0.067527 | -0.32066 | -0.22908 | GNB2L1 |
| ILMN_1714952 | 0.004506 | 2.11E-04 | 0.332297 | 0.106264 | -0.24056 | 0.122927 | ZNF703 |
| ILMN_2278653 | 0.005246 | 3.10E-04 | -0.03507 | 0.192166 | 0.126367 | -0.49864 | ZNF493 |
| ILMN_1777049 | 0.012923 | 0.001585 | 0.13627 | 0.056144 | -0.30433 | -0.31456 | ZNF160 |
| ILMN_1674116 | 0.004065 | 1.59E-04 | 0.338717 | 0.032808 | -0.21652 | 0.159278 | FLJ37543 |
| ILMN_1786426 | 0.001337 | 9.03E-06 | 0.500374 | 0.193603 | -0.44798 | -0.23446 | TMEM149 |
| ILMN_2259467 | 0.009663 | 9.75E-04 | 0.437056 | -0.04909 | -0.12139 | 0.114244 | N4BP2L1 |
| ILMN_1685279 | 0.007747 | 6.70E-04 | 0.185403 | 0.096319 | -0.29455 | -0.06027 | LOC375748 |
| ILMN_1789846 | 0.021887 | 0.003508 | -0.36333 | 0.233832 | 0.009879 | -1.18893 | ITGB6 |
| ILMN_3238078 | 0.001739 | 1.54E-05 | 0.448159 | 0.04614 | -0.27143 | 0.209657 | SNORA45 |
| ILMN_1671398 | 0.003387 | 9.09E-05 | 0.36881 | -0.02654 | -0.33676 | 0.173304 | NPAS3 |
| ILMN_1666372 | 0.00742 | 6.23E-04 | 0.323644 | -0.031 | -0.22416 | -4.57E-04 | ATP5H |
| ILMN_1803452 | 0.010614 | 0.001143 | 0.326213 | 0.047293 | -0.22486 | 0.139451 | CRCT1 |
| ILMN_2398077 | 0.03257 | 0.006318 | 0.06719 | 0.143598 | -0.37444 | 0.141623 | MEIS2 |
| ILMN_1676955 | 0.010194 | 0.001069 | 0.226593 | 0.051594 | -0.30238 | -0.11302 | TYK2 |
| ILMN_1652807 | 0.003087 | 6.65E-05 | 0.490905 | -0.11065 | -0.28418 | -0.5314 | HOXA3 |
| ILMN_2364376 | 0.024992 | 0.00428 | 0.278357 | 0.132958 | -0.18613 | 0.021068 | ILK |
| ILMN_1714980 | 0.021887 | 0.003509 | 0.822345 | 0.321493 | -0.31124 | 0.042871 | MRGPRD |
| ILMN_1714848 | 0.012268 | 0.001451 | 0.355012 | 0.037634 | -0.19745 | 0.050356 | ZNF354A |
| ILMN_1811050 | 0.024155 | 0.004065 | -0.02962 | 0.020829 | 0.003055 | -0.8169 | CCDC88A |
| ILMN_3246821 | 0.027303 | 0.004872 | 0.292779 | -0.02666 | -0.33352 | -0.04405 | MYO1H |
| ILMN_3247334 | 9.67E-04 | 4.70E-06 | 0.646174 | 0.025994 | -0.2931 | 0.08238 | C3orf43 |
| ILMN_1797189 | 0.008183 | 7.33E-04 | 0.442162 | 0.043139 | -0.13938 | -0.04791 | MAP3K12 |
| ILMN_2267131 | 0.003356 | 8.75E-05 | 0.419492 | -0.03465 | -0.30769 | 0.145476 | MAGEA11 |
| ILMN_1758633 | 0.004626 | 2.23E-04 | 0.271861 | 0.079618 | -0.31573 | 0.040842 | CCDC130 |
| ILMN_2359096 | 0.033592 | 0.006597 | -0.45384 | -0.21016 | 0.236307 | -0.77412 | SS18 |
| ILMN_3245912 | 0.004596 | 2.21E-04 | 0.174313 | 0.088332 | -0.40662 | -0.19984 | TMEM59L |
| ILMN_1704797 | 0.040655 | 0.008615 | 0.435517 | 0.304655 | -0.39436 | 0.391564 | CLEC10A |
| ILMN_1779171 | 0.046674 | 0.010539 | 0.343858 | 0.187829 | -0.10829 | -0.22876 | SGSM2 |
| ILMN_1730351 | 0.022373 | 0.003654 | 0.223065 | 0.12623 | -0.31846 | 0.116619 | FLJ35767 |
| ILMN_2343917 | 0.019653 | 0.002965 | 0.445351 | -0.20649 | -0.31347 | 0.101712 | ABCD4 |
| ILMN_1664439 | 0.005743 | 3.76E-04 | 0.108238 | -0.06399 | -0.36179 | 0.016927 | TAF1 |
| ILMN_1695719 | 9.69E-04 | 4.76E-06 | 0.30579 | 0.031459 | -0.30886 | 0.242882 | EIF2C2 |
| ILMN_2095133 | 0.011611 | 0.001336 | 0.347353 | -0.01415 | -0.23599 | -0.04651 | SPTAN1 |
| ILMN_1751135 | 0.003641 | 1.15E-04 | 0.377876 | 0.021181 | -0.27879 | 0.053658 | GGA3 |
| ILMN_1767020 | 0.040735 | 0.008649 | -0.38366 | 0.305894 | -0.40994 | 0.595097 | DENND1C |
| ILMN_2376205 | 0.006404 | 4.68E-04 | -0.19431 | 0.801203 | -0.49093 | -0.69746 | LTB |
| ILMN_1813100 | 0.043487 | 0.009525 | 0.252853 | -0.03 | -0.20987 | -0.07949 | KIAA1244 |
| ILMN_1674908 | 1.30E-04 | 1.92E-07 | 0.427662 | 0.110283 | -0.18744 | -0.74825 | HOXB5 |
| ILMN_1669352 | 0.037864 | 0.007779 | 0.281341 | 0.246456 | -0.24781 | 0.218741 | LY6G6C |
| ILMN_1693136 | 0.012746 | 0.00155 | 0.317601 | -0.14213 | -0.09194 | 0.023812 | VTI1B |
| ILMN_1653797 | 8.85E-04 | 4.06E-06 | -0.42299 | -0.03825 | 0.243349 | -0.548 | C6orf62 |
| ILMN_1794512 | 0.033261 | 0.006499 | 0.113251 | 0.265936 | -0.26158 | 0.170444 | ADPRH |
| ILMN_1657697 | 0.012268 | 0.001452 | 0.302617 | 0.150767 | 0.729944 | -0.83713 | SAR1A |
| ILMN_1688997 | 0.00308 | 6.54E-05 | 0.404899 | 0.043011 | -0.34335 | 0.193414 | C17orf58 |
| ILMN_2359287 | 0.009447 | 9.45E-04 | 0.432322 | 0.019836 | -0.11193 | 0.10581 | ITGA6 |
| ILMN_1798064 | 0.00333 | 8.61E-05 | 0.416182 | 0.032743 | -0.20323 | 0.396958 | ARNTL2 |
| ILMN_1702385 | 0.003641 | 1.15E-04 | 0.16877 | 0.039872 | -0.30967 | 0.142733 | LILRA4 |
| ILMN_3229324 | 0.006225 | 4.39E-04 | 0.153346 | -0.13008 | -0.04702 | 0.760567 | SGK1 |
| ILMN_1791533 | 0.003277 | 7.88E-05 | 0.524466 | 0.223294 | -0.27904 | 0.21079 | HAP1 |
| ILMN_1735014 | 0.043064 | 0.009365 | 0.044359 | -0.39262 | -0.28511 | 0.907035 | KLF6 |
| ILMN_1814631 | 0.003277 | 7.80E-05 | 0.099897 | 0.095018 | -0.28009 | 0.210831 | SMCP |
| ILMN_1749410 | 0.006328 | 4.55E-04 | 0.374747 | 0.018256 | -0.13909 | -0.04243 | PKP4 |
| ILMN_1690920 | 0.022367 | 0.003648 | -0.44095 | -0.15456 | 0.227364 | -0.24164 | SP100 |
| ILMN_2363668 | 0.004238 | 1.84E-04 | 0.146195 | 0.366929 | -0.31574 | 0.071918 | YIF1B |
| ILMN_1800889 | 0.025829 | 0.004508 | 0.153881 | 0.170519 | -0.1391 | 0.222953 | FIG4 |
| ILMN_1781752 | 0.006173 | 4.31E-04 | 0.174943 | 0.075653 | -0.23652 | 0.101726 | CLEC16A |
| ILMN_1755737 | 0.024291 | 0.004097 | 0.204129 | 0.07142 | -0.28375 | 0.023078 | TRABD |
| ILMN_1666280 | 0.027882 | 0.005021 | 0.244718 | 0.076101 | 0.040004 | -0.67282 | COX11 |
| ILMN_2344798 | 0.004976 | 2.65E-04 | 0.353942 | 0.055995 | -0.22805 | 0.161389 | APAF1 |
| ILMN_3247906 | 0.006662 | 5.13E-04 | 0.137731 | -0.0977 | -0.36068 | 0.267849 | RNF114 |
| ILMN_2145280 | 0.004563 | 2.19E-04 | 0.105786 | 0.619383 | -0.51243 | -0.01157 | DEF6 |
| ILMN_2323801 | 0.012503 | 0.001497 | 0.270455 | -0.13025 | -0.35726 | -0.03948 | MOCS1 |
| ILMN_1743142 | 0.001835 | 1.79E-05 | 0.890625 | 0.205594 | -0.23923 | -0.02271 | LASS3 |
| ILMN_3240231 | 0.033562 | 0.00658 | 0.231251 | -0.18801 | -0.39463 | 0.082115 | SNORA34 |
| ILMN_1692714 | 0.004191 | 1.74E-04 | 0.646802 | 0.167162 | -0.24906 | 0.183166 | TBX21 |
| ILMN_1803745 | 0.015236 | 0.002049 | 0.327288 | 0.010804 | -0.12753 | 0.122615 | SUOX |
| ILMN_1745811 | 0.012796 | 0.001564 | 0.330611 | -0.21354 | -0.22816 | -0.03512 | TDRD3 |
| ILMN_2366463 | 0.005956 | 4.02E-04 | 0.217135 | 0.14561 | -0.46705 | -0.45097 | FN1 |
| ILMN_2113490 | 0.020015 | 0.003063 | 0.270635 | -0.03576 | -0.18835 | -0.27487 | NTN4 |
| ILMN_2413732 | 0.006706 | 5.22E-04 | 0.356383 | 0.095189 | -0.23549 | 0.051774 | ADAMTS13 |
| ILMN_1699121 | 0.045966 | 0.010305 | 0.265371 | 0.245052 | -0.0884 | 0.115234 | TMEM16B |
| ILMN_1783276 | 0.006643 | 5.07E-04 | 0.504204 | -0.20962 | 0.077257 | -0.3742 | NEXN |
| ILMN_1743995 | 0.0082 | 7.36E-04 | 0.659675 | -0.24778 | -0.30879 | -0.20139 | ALG12 |
| ILMN_1746031 | 0.040659 | 0.00862 | 0.355385 | 0.073066 | -0.17383 | 0.04064 | RIMS4 |
| ILMN_1653480 | 0.006173 | 4.31E-04 | 0.347735 | 0.171891 | -0.31937 | -0.07269 | KENAE |
| ILMN_1716583 | 0.009271 | 9.16E-04 | 0.029336 | -0.3382 | 0.221948 | -0.36763 | NME7 |
| ILMN_1772537 | 0.04965 | 0.01151 | 0.546692 | 0.037182 | -0.17458 | -0.83226 | SCTR |
| ILMN_1812679 | 0.010308 | 0.001087 | 0.407761 | 0.042871 | -0.24889 | 0.083405 | UPK1B |
| ILMN_1762879 | 0.007063 | 5.72E-04 | 0.295329 | -0.06031 | -0.23532 | 0.130152 | DCAF4L1 |
| ILMN_2077094 | 0.004479 | 2.10E-04 | 0.170368 | 0.013981 | -0.34433 | 0.153507 | C11orf2 |
| ILMN_1700337 | 0.017825 | 0.002584 | 0.683836 | 0.012889 | -0.20517 | 0.088672 | TROAP |
| ILMN_3236135 | 0.003502 | 9.96E-05 | 0.537461 | 0.102078 | -0.21524 | 0.074448 | FAM86D |
| ILMN_1740024 | 0.035315 | 0.00709 | 0.147677 | -0.17433 | -0.0349 | -0.61377 | NAALAD2 |
| ILMN_1703411 | 0.011014 | 0.00122 | 0.386952 | -0.03629 | -0.1868 | 0.073164 | PARP3 |
| ILMN_2156172 | 0.004068 | 1.62E-04 | 0.322948 | 0.044625 | -0.22277 | 0.140326 | HK2 |
| ILMN_1781281 | 0.043795 | 0.009618 | 0.346647 | -0.41635 | -0.03923 | 0.221557 | EPPB9 |
| ILMN_1794132 | 0.013762 | 0.001737 | 0.279297 | -0.17365 | -0.2407 | 0.163087 | NDUFS8 |
| ILMN_1690217 | 0.004351 | 1.97E-04 | 0.845978 | 0.171106 | -0.24966 | 0.17917 | BFSP2 |
| ILMN_1757288 | 0.003531 | 1.02E-04 | 0.518032 | -0.02735 | -0.23259 | 0.234722 | KLHDC8A |
| ILMN_1694057 | 0.004212 | 1.82E-04 | 0.242733 | -0.04232 | -0.16441 | 0.189665 | EIF3K |
| ILMN_1763568 | 0.01371 | 0.001726 | 0.296203 | -0.04103 | -0.19246 | -0.07218 | ZDHHC16 |
| ILMN_2215211 | 0.041588 | 0.008925 | -0.06036 | 0.353552 | 0.015206 | -0.44203 | ZNF514 |
| ILMN_1674560 | 0.010587 | 0.001139 | 0.35544 | 0.046759 | -0.12973 | 0.272799 | GBA2 |
| ILMN_1762883 | 0.012923 | 0.001584 | 0.39522 | 0.003833 | -0.22187 | 0.032946 | ECE2 |
| ILMN_1699991 | 0.019653 | 0.002967 | 0.24554 | 0.186617 | -0.29567 | 0.104221 | LCK |
| ILMN_1687247 | 0.0051 | 2.84E-04 | 0.0782 | 0.075056 | -0.33026 | 0.16656 | SPATA20 |
| ILMN_1725187 | 0.034014 | 0.00672 | 0.77012 | -0.18325 | -0.09847 | 0.204239 | PITPNM2 |
| ILMN_1799467 | 0.027051 | 0.004794 | -0.19048 | -0.20948 | 0.230165 | -0.70696 | SAMD9L |
| ILMN_1807525 | 0.006498 | 4.86E-04 | 1.194545 | -0.17014 | -0.21547 | -0.04353 | CNTD2 |
| ILMN_1746029 | 0.025443 | 0.004394 | 0.398195 | 0.086759 | -0.23446 | -0.11656 | SPATS2 |
| ILMN_1729208 | 0.007922 | 6.97E-04 | 0.361746 | -0.06652 | -0.16477 | 0.067638 | NGFRAP1 |
| ILMN_2370091 | 0.039137 | 0.008155 | 0.237933 | -0.05705 | -0.21721 | -0.26613 | NGFRAP1 |
| ILMN_1666175 | 0.007197 | 5.93E-04 | 0.393693 | -0.03262 | -0.21664 | 0.192128 | IL22RA1 |
| ILMN_1687922 | 0.030907 | 0.005871 | 0.26076 | 0.119497 | -0.23258 | -0.06481 | RP9 |
| ILMN_1815568 | 0.001835 | 1.79E-05 | 0.266732 | 0.049014 | -0.35609 | 0.11933 | PLXDC1 |
| ILMN_1699249 | 0.006253 | 4.42E-04 | -0.25817 | 0.482111 | 0.098579 | -1.05813 | ZNF649 |
| ILMN_1813341 | 0.005182 | 2.99E-04 | 0.348193 | 0.02184 | -0.23571 | 0.152939 | PTGFR |
| ILMN_2135272 | 0.010049 | 0.001038 | 0.232662 | 0.037924 | -0.18784 | 0.182362 | GIMAP2 |
| ILMN_1703743 | 0.030231 | 0.005673 | -1.09839 | -0.32934 | -0.02159 | 0.008087 | AATF |
| ILMN_1764891 | 0.019459 | 0.002922 | 0.478688 | -0.04185 | -0.14557 | 0.035674 | ZNF384 |
| ILMN_1769449 | 0.048324 | 0.011098 | -0.23169 | -0.12512 | 0.057896 | 0.447846 | MBLAC2 |
| ILMN_2193752 | 0.006575 | 4.96E-04 | 0.104938 | 0.057049 | -0.29512 | 0.23669 | MBLAC2 |
| ILMN_3300353 | 0.026376 | 0.004636 | -0.52808 | -0.31103 | 0.435084 | -0.21352 | LOC729920 |
| ILMN_1696392 | 0.005956 | 4.00E-04 | 0.36209 | 0.096861 | -0.29662 | 0.386516 | SPDYA |
| ILMN_1782429 | 0.028178 | 0.00511 | -0.31392 | -0.67735 | 0.146431 | -0.08304 | TMEM56 |
| ILMN_1798467 | 5.74E-04 | 2.06E-06 | 0.317567 | -0.02127 | -0.30862 | -0.03184 | FRYL |
| ILMN_1754078 | 0.001368 | 9.69E-06 | 0.770962 | -0.15822 | -0.33934 | 0.413129 | CABP7 |
| ILMN_1754220 | 0.003003 | 6.10E-05 | 0.174729 | -0.11819 | -0.72993 | -0.13402 | SF3A2 |
| ILMN_1712751 | 0.013846 | 0.001756 | 0.203837 | -0.01188 | -0.27178 | 0.13316 | HADHA |
| ILMN_2349138 | 0.004353 | 1.97E-04 | -0.33025 | -0.11601 | 0.482967 | -0.58587 | CDC42SE1 |
| ILMN_1703374 | 0.021923 | 0.00352 | 0.235326 | -0.02617 | -0.30717 | 0.007233 | NAV1 |
| ILMN_1790534 | 0.048082 | 0.01102 | 0.145757 | -0.02033 | -0.29306 | -0.05308 | MAP2K3 |
| ILMN_1815238 | 0.017134 | 0.002434 | 0.31343 | -0.55921 | -0.2306 | 0.812276 | MAP2K3 |
| ILMN_3307767 | 0.00716 | 5.85E-04 | 0.152109 | 0.009216 | -0.21334 | 0.160985 | WNT9A |
| ILMN_2192683 | 0.001409 | 1.08E-05 | 0.261023 | 0.097002 | -0.32426 | 0.17027 | DHX37 |
| ILMN_1811104 | 0.008781 | 8.34E-04 | 0.126476 | 0.102115 | -0.27188 | -0.11414 | KTELC1 |
| ILMN_1651719 | 0.016837 | 0.002375 | 0.238741 | 0.100067 | -0.23936 | -0.00853 | MBTPS1 |
| ILMN_1795811 | 0.003795 | 1.27E-04 | 0.134703 | 0.044709 | -0.28258 | 0.193519 | ASB2 |
| ILMN_1704713 | 0.003277 | 7.87E-05 | 0.511043 | -0.02002 | -0.12063 | 0.107846 | CSNK1G1 |
| ILMN_1758055 | 0.020139 | 0.0031 | -0.57951 | -0.27554 | 0.197287 | -0.47949 | YIF1B |
| ILMN_1770260 | 0.043454 | 0.009506 | 0.558309 | -0.9173 | -0.65662 | 1.380963 | NFKBIZ |
| ILMN_1739101 | 0.009135 | 8.96E-04 | 0.671702 | 0.049172 | -0.14884 | 0.189067 | RBBP8 |
| ILMN_3251638 | 0.02999 | 0.005616 | 0.146958 | 0.043924 | -0.20061 | 0.042724 | TRIM49 |
| ILMN_1706531 | 0.021914 | 0.003516 | -0.06964 | 0.144926 | 0.039871 | -1.09824 | ABCC5 |
| ILMN_1651752 | 0.004191 | 1.76E-04 | 0.253245 | -0.03062 | -0.29001 | 0.1559 | CXorf21 |
| ILMN_1679339 | 0.002754 | 4.76E-05 | 0.360003 | 0.043843 | -0.29802 | 0.14201 | C3orf62 |
| ILMN_1733045 | 0.021866 | 0.003501 | -0.1577 | -0.01698 | 0.436696 | -0.64595 | RAB36 |
| ILMN_1724346 | 0.00523 | 3.06E-04 | 0.455747 | 0.10539 | -0.25873 | 0.079424 | IPMK |
| ILMN_2398107 | 0.005298 | 3.17E-04 | 0.265078 | 0.177192 | -0.1811 | -0.20762 | ASNS |
| ILMN_1664292 | 0.005247 | 3.11E-04 | -3.83E-04 | -0.68749 | 0.511993 | -1.02521 | ZNF415 |
| ILMN_1690342 | 0.04493 | 0.009972 | 0.103182 | -0.09717 | 0.039736 | -0.65992 | LTA4H |
| ILMN_1753468 | 0.025004 | 0.004283 | 0.104207 | 0.01903 | -0.18618 | -0.25578 | CD63 |
| ILMN_1781812 | 0.002556 | 3.91E-05 | 0.281872 | 0.677407 | -0.04668 | 2.061447 | NR4A3 |
| ILMN_2075292 | 0.008993 | 8.75E-04 | 0.419354 | 0.152261 | -0.23736 | 0.205012 | MAGEA9B |
| ILMN_1675083 | 0.047555 | 0.010843 | 0.360257 | -0.06534 | -0.21968 | 0.256598 | F8 |
| ILMN_1764034 | 0.005447 | 3.38E-04 | 0.245664 | 0.13314 | -0.39668 | 0.177532 | XCR1 |
| ILMN_1722227 | 0.02521 | 0.004341 | 0.716898 | 0.4805 | -0.04828 | -0.03795 | MRGPRX4 |
| ILMN_2376289 | 0.014003 | 0.001793 | 0.256763 | 0.226732 | -0.19137 | -0.29309 | DBNL |
| ILMN_1677404 | 0.034014 | 0.006719 | -0.3445 | 0.163823 | 0.02649 | -0.17241 | RAP2A |
| ILMN_2242635 | 0.004191 | 1.74E-04 | 0.774285 | -0.05778 | -0.27594 | 0.063736 | SSX3 |
| ILMN_1714759 | 0.049137 | 0.011369 | 0.230615 | -0.05782 | -0.17542 | -0.12571 | CNIH4 |
| ILMN_1676361 | 0.011297 | 0.001269 | 0.215416 | 0.082506 | -0.43657 | 0.146174 | ARHGAP22 |
| ILMN_1666007 | 0.030826 | 0.005845 | 0.284192 | -0.07879 | -0.0054 | -0.27946 | TRAPPC6B |
| ILMN_1757536 | 0.011797 | 0.001368 | 0.251805 | -0.06008 | -0.2758 | 0.075462 | USP40 |
| ILMN_1690921 | 0.002621 | 4.31E-05 | 0.34138 | 0.199779 | -0.16691 | -0.24825 | STAT2 |
| ILMN_1757838 | 0.008704 | 8.17E-04 | 0.36237 | -0.08668 | -0.19849 | 0.17243 | HGF |
| ILMN_1667288 | 0.006763 | 5.32E-04 | 0.628736 | -0.19862 | -0.21557 | 0.781752 | SNORD73A |
| ILMN_1708881 | 0.015671 | 0.002136 | 0.113947 | -0.18465 | -0.2242 | 0.745214 | RAB20 |
| ILMN_2244140 | 0.006165 | 4.28E-04 | 0.197133 | 0.18676 | -0.32539 | 0.117685 | UNC13A |
| ILMN_1754943 | 0.012485 | 0.001494 | 0.275715 | -0.21395 | -0.17763 | -0.15344 | MYO1E |
| ILMN_2229913 | 0.003408 | 9.34E-05 | 0.297797 | 0.032392 | -0.18067 | 0.176914 | KIR3DP1 |
| ILMN_1806030 | 0.0211 | 0.003315 | 0.259445 | -0.08379 | -0.23323 | -0.16623 | PPL |
| ILMN_2392286 | 0.00333 | 8.63E-05 | 0.177391 | 0.026944 | -0.35507 | -0.00626 | IP6K1 |
| ILMN_1698726 | 0.009777 | 9.93E-04 | 0.277185 | 0.048578 | -0.27916 | -0.03809 | SLC25A27 |
| ILMN_1763080 | 0.002424 | 3.50E-05 | 0.359229 | 0.029231 | -0.24701 | 0.111216 | QARS |
| ILMN_1790354 | 0.039974 | 0.008395 | 0.322357 | -0.15099 | -0.03738 | 0.173215 | RABGGTB |
| ILMN_1737433 | 0.016593 | 0.002327 | 0.982689 | 0.240678 | -0.45207 | 0.342655 | RARG |
| ILMN_1704985 | 0.004944 | 2.60E-04 | 0.270158 | -0.56926 | -0.07152 | 0.448051 | CYP27A1 |
| ILMN_1682054 | 0.047048 | 0.010667 | 0.245314 | 0.159708 | -0.27698 | -0.09926 | SRI |
| ILMN_2259949 | 0.008307 | 7.55E-04 | 0.437045 | 0.08777 | -0.28091 | 0.066849 | BCKDHB |
| ILMN_2067453 | 0.004611 | 2.22E-04 | 0.550691 | -0.04273 | -0.29899 | 0.238197 | LCAP |
| ILMN_1735979 | 0.012644 | 0.001533 | 0.192885 | 0.014103 | -0.35659 | -0.06878 | BCKDHA |
| ILMN_1727855 | 0.007896 | 6.92E-04 | 0.470862 | -0.62192 | -0.50083 | 0.794386 | PEMT |
| ILMN_2181469 | 0.042614 | 0.009219 | 0.121253 | -0.05682 | 0.162161 | -0.54877 | ZNF611 |
| ILMN_1726783 | 0.020389 | 0.003159 | -0.34531 | -0.98547 | 0.440518 | -0.53475 | RNASEH1 |
| ILMN_1761159 | 0.036932 | 0.007521 | 0.14438 | 0.227589 | -0.16517 | -0.05593 | ESYT1 |
| ILMN_2370414 | 0.02528 | 0.004358 | 0.436018 | 0.028641 | -0.13352 | 0.04915 | CHD3 |
| ILMN_1754163 | 0.004305 | 1.92E-04 | 0.309089 | 0.012318 | -0.19995 | 0.179665 | DDX53 |
| ILMN_1682081 | 0.014354 | 0.001866 | 0.407065 | 0.092872 | -0.36483 | -0.10929 | RNF19B |
| ILMN_3236289 | 0.005027 | 2.73E-04 | 0.416376 | -0.00493 | -0.29294 | 0.15657 | TIGIT |
| ILMN_2352131 | 0.01739 | 0.00249 | 0.084609 | -0.13833 | 0.00252 | -0.75499 | ERBB2 |
| ILMN_2259815 | 0.004034 | 1.53E-04 | 0.273226 | 0.166936 | -0.30209 | 0.179721 | TBX20 |
| ILMN_1805161 | 0.031305 | 0.005973 | 0.185331 | 0.130764 | -0.18126 | 0.085199 | LZTR1 |
| ILMN_1679979 | 0.007169 | 5.88E-04 | 0.325516 | 0.038158 | -0.21601 | 0.21546 | PLK3 |
| ILMN_1721024 | 0.010672 | 0.001154 | -0.14718 | -0.18692 | 0.536238 | -0.64446 | SRFBP1 |
| ILMN_1743619 | 0.020318 | 0.003144 | 0.464159 | -0.16763 | 0.039954 | 0.731385 | NEDD9 |
| ILMN_1724282 | 0.006098 | 4.20E-04 | 0.593985 | 0.133587 | -0.19778 | 0.158443 | LOC401296 |
| ILMN_1780357 | 0.002291 | 3.02E-05 | 0.286445 | 0.035133 | -0.2914 | 0.208846 | PRRT1 |
| ILMN_3240236 | 5.71E-04 | 1.99E-06 | -0.14314 | 0.439622 | 0.29635 | -0.99477 | SMCR5 |
| ILMN_2233050 | 0.039323 | 0.008212 | 0.044933 | 0.36173 | 0.062624 | -0.83115 | PLA2G2D |
| ILMN_1728360 | 0.017881 | 0.002596 | 0.091741 | 0.097521 | -0.05648 | -0.55501 | MED29 |
| ILMN_2180929 | 0.039513 | 0.008274 | 0.242418 | 0.129408 | -0.26944 | -0.20091 | SLC16A12 |
| ILMN_1666894 | 0.011128 | 0.001241 | 0.338886 | 0.221438 | -0.23091 | -0.07454 | CSPG4 |
| ILMN_1736256 | 0.0114 | 0.001287 | -0.20347 | 0.117409 | 0.059121 | -0.47103 | CALR |
| ILMN_1766814 | 0.034214 | 0.006774 | 0.158975 | -0.91277 | 0.175461 | -0.02907 | TK2 |
| ILMN_1664186 | 0.00716 | 5.87E-04 | -0.0763 | -0.2018 | 0.387278 | -0.82218 | MFN1 |
| ILMN_1682930 | 0.00532 | 3.20E-04 | 0.216071 | 0.21985 | -0.22049 | -0.17726 | SIPA1 |
| ILMN_3231020 | 0.004639 | 2.27E-04 | 0.417714 | 0.075265 | -0.2689 | 0.157232 | PSMG4 |
| ILMN_2384770 | 0.010428 | 0.00111 | 0.129227 | -0.14164 | -0.25423 | 2.058022 | EGFLAM |
| ILMN_2308849 | 0.046137 | 0.010365 | 0.073095 | -0.63826 | -0.14818 | 0.64628 | MYADM |
| ILMN_2357386 | 0.004508 | 2.12E-04 | 0.258706 | 0.202053 | -0.26442 | 0.160851 | FKTN |
| ILMN_2318932 | 0.004034 | 1.52E-04 | 0.357877 | 0.056892 | -0.30424 | 0.156376 | DUSP27 |
| ILMN_1678605 | 0.012863 | 0.001574 | 0.077555 | -0.31603 | -0.25076 | 0.398231 | CDC123 |
| ILMN_1709717 | 0.003753 | 1.24E-04 | 0.362878 | 0.109052 | -0.31921 | 0.108606 | LRRC15 |
| ILMN_1735004 | 0.00716 | 5.83E-04 | -0.06605 | 0.215902 | 0.269807 | -0.61315 | C4orf43 |
| ILMN_3250970 | 0.004191 | 1.73E-04 | 0.376935 | 0.00389 | -0.2464 | 0.139936 | DAZ2 |
| ILMN_1667915 | 0.001985 | 2.18E-05 | 0.453264 | 0.050267 | -0.29375 | 0.139081 | TRIM67 |
| ILMN_1754865 | 0.008614 | 8.02E-04 | 0.212374 | -0.01655 | -0.20272 | 0.078234 | LOC400455 |
| ILMN_1744508 | 0.003475 | 9.75E-05 | 0.154551 | -0.13938 | -0.35797 | 0.167231 | FAM53C |
| ILMN_1655426 | 0.004476 | 2.09E-04 | 0.253815 | 0.049538 | -0.31326 | 0.17492 | KLK3 |
| ILMN_1698307 | 0.013708 | 0.001726 | 0.208171 | 0.135391 | -0.16494 | 0.051686 | DBNL |
| ILMN_1788604 | 0.031354 | 0.005987 | 0.276629 | 0.0653 | -0.1231 | 0.102458 | WBP2 |
| ILMN_1800082 | 0.010188 | 0.001068 | 0.325274 | 0.225637 | -0.33037 | -0.0149 | B3GNT3 |
| ILMN_1780940 | 0.004447 | 2.06E-04 | 0.491783 | 0.049784 | -0.29443 | 0.201878 | PPP2R5D |
| ILMN_1805180 | 0.037214 | 0.007588 | 1.137047 | 0.359457 | -0.50464 | 0.460175 | CDAN1 |
| ILMN_1693538 | 0.007858 | 6.85E-04 | 0.245162 | 0.084547 | -0.28825 | -0.12292 | STK36 |
| ILMN_1712517 | 0.041403 | 0.008869 | -0.24077 | 0.264176 | 0.048665 | -0.44838 | ZNF696 |
| ILMN_2053650 | 0.033962 | 0.006701 | 0.290718 | -0.01667 | -0.21009 | 0.049349 | AURKAPS1 |
| ILMN_1801443 | 0.020015 | 0.003061 | 0.37067 | -0.02715 | -0.2629 | 0.136247 | TSKU |
| ILMN_1695316 | 0.018481 | 0.002726 | 0.343071 | -0.11157 | -0.2043 | -0.02918 | SLC39A8 |
| ILMN_1768049 | 0.003093 | 6.70E-05 | 0.476265 | -0.06201 | -0.29563 | 0.098934 | FOXP3 |
| ILMN_1657455 | 0.009895 | 0.001015 | 0.358349 | 0.134466 | -0.2808 | 0.13538 | CEACAM4 |
| ILMN_1706021 | 0.009248 | 9.11E-04 | 0.313498 | -0.0116 | -0.39075 | -0.10222 | TMPRSS4 |
| ILMN_1681544 | 0.013958 | 0.001781 | 0.207511 | 0.199405 | -0.25994 | 0.121809 | RASGEF1C |
| ILMN_1769934 | 0.014238 | 0.001835 | 0.353482 | -0.03817 | -0.38024 | 0.047993 | GRHL1 |
| ILMN_1731478 | 0.008557 | 7.94E-04 | 0.218292 | 0.015617 | -0.30132 | -0.01669 | RGS3 |
| ILMN_1760027 | 0.043348 | 0.00947 | -0.16465 | 0.254116 | -0.31141 | -0.06259 | WAS |
| ILMN_2330410 | 0.026726 | 0.004725 | 0.080382 | 0.487131 | -0.15144 | 0.298593 | EIF3C |
| ILMN_2324162 | 0.005471 | 3.41E-04 | 0.808773 | 0.178328 | -0.28199 | 0.283838 | SLC45A1 |
| ILMN_1674609 | 0.00499 | 2.66E-04 | 0.323332 | 0.014089 | -0.31433 | 0.07011 | CLTB |
| ILMN_2249473 | 0.038592 | 0.007975 | -0.15536 | 0.213277 | 0.019786 | -0.75848 | SPTLC1 |
| ILMN_1665094 | 0.001403 | 1.04E-05 | 0.408045 | -0.06303 | -0.2434 | 0.114211 | SPTLC1 |
| ILMN_1705904 | 0.025697 | 0.004463 | 0.591087 | 0.452321 | -0.06858 | 1.087045 | C1orf138 |
| ILMN_1702055 | 0.005073 | 2.78E-04 | 0.417385 | -0.0691 | -0.26482 | 0.084883 | ROPN1B |
| ILMN_1659936 | 3.26E-05 | 3.08E-08 | 0.379581 | 0.0671 | -0.25796 | 0.773694 | PPP1R15A |
| ILMN_2154223 | 0.020429 | 0.003175 | 0.254195 | 0.168085 | -0.27173 | 0.016802 | CEP76 |
| ILMN_1806003 | 0.021727 | 0.003463 | 0.333485 | 0.019633 | -0.22126 | -0.13607 | SPEF2 |
| ILMN_2408566 | 3.79E-06 | 2.33E-09 | 0.154324 | 0.135636 | -0.15982 | 3.509172 | NR4A1 |
| ILMN_1662932 | 0.041704 | 0.008969 | -0.19391 | 0.378896 | -0.27161 | -0.95969 | LCP1 |
| ILMN_3241140 | 0.006004 | 4.08E-04 | 0.597841 | -0.24602 | -0.37136 | 0.217906 | SNORA14B |
| ILMN_1784493 | 0.02381 | 0.003984 | 0.290673 | -0.08227 | -0.27576 | 0.426918 | LOC401286 |
| ILMN_1810100 | 0.023197 | 0.003848 | 0.149171 | 0.026628 | 0.261937 | -0.53287 | PBX3 |
| ILMN_2326071 | 0.022067 | 0.003562 | 0.192396 | 0.001051 | -0.16531 | 0.120314 | MYL6 |
| ILMN_1711311 | 0.010838 | 0.001186 | 0.284015 | -0.22498 | -0.19731 | -0.02605 | PODXL |
| ILMN_1797307 | 0.004014 | 1.48E-04 | 0.089072 | 0.028292 | -0.25379 | 0.181055 | BUB1B |
| ILMN_1755251 | 0.006731 | 5.27E-04 | 0.424635 | 0.036509 | -0.2637 | -0.03415 | ITIH1 |
| ILMN_1724234 | 0.003028 | 6.28E-05 | 0.273522 | -0.00588 | -0.36693 | 0.110216 | TRPV1 |
| ILMN_1781814 | 0.034577 | 0.006877 | 0.60558 | 0.097887 | -0.27809 | -0.04517 | SEMA4B |
| ILMN_1758672 | 0.005773 | 3.80E-04 | 0.274589 | -0.01945 | -0.23183 | -0.0914 | FAM107B |
| ILMN_2209748 | 0.013994 | 0.00179 | -0.64656 | -0.45564 | 0.177785 | 0.167331 | DERL1 |
| ILMN_2293677 | 0.011023 | 0.001223 | 0.272481 | 0.077417 | -0.26861 | 0.167812 | STAG2 |
| ILMN_1701220 | 0.035867 | 0.007223 | 0.487707 | -0.07447 | -0.35995 | -0.08697 | PIWIL1 |
| ILMN_1727205 | 0.005956 | 4.02E-04 | 0.315398 | 0.089961 | -0.21947 | -0.10127 | ARHGEF10L |
| ILMN_1689770 | 0.031321 | 0.005978 | 0.067638 | 0.162097 | -0.26557 | -0.02698 | ARHGEF10L |
| ILMN_1661000 | 0.025504 | 0.004414 | 0.295829 | 0.217314 | -0.17574 | 0.023811 | RPS6KC1 |
| ILMN_3239225 | 0.033736 | 0.00664 | 0.313197 | 0.025108 | -0.21148 | 0.060029 | RNY3 |
| ILMN_1742538 | 0.004194 | 1.81E-04 | -0.01481 | -0.03898 | -0.4426 | 0.805958 | PCDHGC4 |
| ILMN_1773200 | 0.017042 | 0.002413 | -0.14437 | -0.00169 | 0.247842 | -0.93387 | CP110 |
| ILMN_1698584 | 0.016374 | 0.002275 | 0.480831 | 0.181106 | -0.21624 | 0.446893 | KRTAP4-12 |
| ILMN_1678546 | 0.010383 | 0.001101 | -0.06002 | 0.023664 | -0.13501 | 0.258731 | PEX11B |
| ILMN_2129600 | 0.015109 | 0.002016 | 0.362583 | -0.12179 | -0.2282 | 0.194853 | OR4A15 |
| ILMN_1796663 | 0.005956 | 4.02E-04 | 0.00737 | -0.14103 | -0.2832 | 0.401076 | B4GALNT4 |
| ILMN_1683786 | 0.031353 | 0.005985 | -0.12106 | 0.149967 | -0.1621 | 0.429226 | FYTTD1 |
| ILMN_2206746 | 0.006484 | 4.82E-04 | 0.271128 | 0.153999 | -0.19356 | -0.16143 | BGN |
| ILMN_1694084 | 6.71E-04 | 2.76E-06 | 0.001443 | 0.374667 | -0.14779 | 0.468448 | PSCD1 |
| ILMN_3188342 | 0.002978 | 5.88E-05 | 0.313698 | -0.01479 | -0.28312 | 0.185742 | LOC100128675 |
| ILMN_1683867 | 0.030341 | 0.005704 | 0.246905 | 0.267543 | -0.27867 | 0.057543 | CENTD1 |
| ILMN_1694475 | 0.013915 | 0.001773 | 0.177948 | 0.082021 | -0.24558 | 0.19253 | GTPBP2 |
| ILMN_1668566 | 0.04493 | 0.009974 | -0.21142 | 0.131166 | 0.00246 | -0.55634 | ANUBL1 |
| ILMN_2114645 | 0.043487 | 0.009525 | 0.546734 | -0.06372 | -0.12898 | -0.00685 | SIAH3 |
| ILMN_1808597 | 0.020016 | 0.003068 | 0.278139 | -0.21259 | -0.31607 | -0.14433 | APLF |
| ILMN_1806407 | 0.015633 | 0.00213 | 0.341485 | -0.0266 | -0.17336 | 0.058116 | SEMA6D |
| ILMN_1712400 | 0.001409 | 1.07E-05 | 0.326923 | -0.00166 | -0.34477 | 0.133313 | SERPINB6 |
| ILMN_1666546 | 0.013892 | 0.001765 | 0.166675 | -0.00642 | -0.29242 | 0.063557 | DUSP14 |
| ILMN_1696709 | 0.003408 | 9.35E-05 | 0.395387 | 0.140543 | -0.27668 | 0.083108 | TADA2A |
| ILMN_1767032 | 0.007054 | 5.71E-04 | 0.4503 | 0.084011 | -0.21525 | 0.091835 | DDI1 |
| ILMN_1779147 | 0.01215 | 0.001428 | 0.355012 | -0.05385 | -0.1698 | -0.10301 | ENC1 |
| ILMN_1715994 | 0.006173 | 4.30E-04 | 0.210234 | 0.042231 | -0.28644 | 0.141525 | HGS |
| ILMN_1721316 | 0.004068 | 1.61E-04 | 0.318136 | 0.011603 | -0.29134 | 0.150289 | TNFRSF10A |
| ILMN_1684445 | 0.003614 | 1.11E-04 | 0.211979 | 0.128569 | -0.27803 | 0.26144 | FCRL5 |
| ILMN_1744006 | 0.009777 | 9.93E-04 | 0.28225 | 0.180296 | -0.16562 | 0.090126 | GFOD2 |
| ILMN_1683660 | 6.45E-04 | 2.45E-06 | 0.2612 | -0.07293 | -0.35313 | 0.085757 | EIF3H |
| ILMN_2155025 | 0.011187 | 0.001251 | -0.34328 | -0.10138 | 0.323666 | -0.78561 | ZNF681 |
| ILMN_1771523 | 0.001093 | 6.14E-06 | 0.394578 | -0.08469 | -0.29525 | 0.20815 | PRKACB |
| ILMN_2150654 | 0.003196 | 7.26E-05 | 0.197974 | 0.117229 | -0.30207 | 0.109215 | ZSWIM4 |
| ILMN_1685978 | 0.004543 | 2.16E-04 | 0.011777 | -0.04517 | 0.096262 | -0.62887 | ATPIF1 |
| ILMN_1652219 | 0.01083 | 0.001184 | 0.151464 | 0.020718 | -0.28428 | 0.346536 | C20orf112 |
| ILMN_1714445 | 0.017865 | 0.002591 | -0.18324 | 0.041567 | -0.44349 | -1.10586 | SLC6A9 |
| ILMN_1720755 | 0.00333 | 8.63E-05 | 0.10008 | -0.06129 | -0.35885 | -0.00773 | C10orf71 |
| ILMN_1759464 | 0.008961 | 8.68E-04 | 0.333474 | -0.0096 | -0.18557 | 0.1286 | C1orf124 |
| ILMN_1780406 | 0.006345 | 4.58E-04 | 0.464888 | 0.374459 | -0.36457 | 0.844256 | C21orf86 |
| ILMN_1689098 | 0.010049 | 0.001038 | 0.607697 | 0.110871 | -0.2182 | 0.092288 | ATP1A3 |
| ILMN_1657893 | 0.044449 | 0.009818 | 0.229562 | -0.02891 | -0.23088 | -0.0082 | TXNRD2 |
| ILMN_1792538 | 0.003387 | 9.15E-05 | -0.11066 | 0.589336 | -0.63422 | 0.324587 | CD7 |
| ILMN_1760303 | 0.044225 | 0.009757 | -0.03375 | -0.11641 | -0.06915 | -0.73999 | PIK3R1 |
| ILMN_1797384 | 0.033052 | 0.006446 | 0.339303 | 0.013525 | -0.19834 | 0.018868 | UROS |
| ILMN_1718552 | 0.025151 | 0.00432 | 0.250783 | 0.326689 | -0.24276 | 0.594411 | CXCL13 |
| ILMN_1744795 | 0.007884 | 6.89E-04 | 0.349321 | 0.027509 | -0.24276 | 0.01806 | TBL1X |
| ILMN_1689652 | 0.008787 | 8.35E-04 | 0.120903 | 0.016863 | -0.27115 | 0.153198 | RNMTL1 |
| ILMN_1652207 | 0.003917 | 1.39E-04 | 0.42696 | 0.027941 | -0.22764 | 0.112325 | COX4I1 |
| ILMN_1674394 | 0.04829 | 0.011083 | 0.310611 | 0.033405 | -0.1875 | -0.08026 | C20orf3 |
| ILMN_3235517 | 0.012552 | 0.001509 | 0.259502 | 0.075822 | -0.28154 | 0.221692 | ZNF777 |
| ILMN_1666632 | 0.024547 | 0.004146 | 0.105348 | -0.03253 | -0.1821 | 0.138133 | VPS52 |
| ILMN_1651710 | 0.010089 | 0.001048 | 0.41116 | 0.503745 | -0.46569 | 0.592158 | IQCD |
| ILMN_1693340 | 0.00616 | 4.28E-04 | 0.212353 | -0.15668 | -0.22272 | 0.146687 | RAC3 |
| ILMN_1721132 | 0.003917 | 1.39E-04 | 0.119168 | 0.029425 | -0.22579 | 0.11009 | DKKL1 |
| ILMN_2295511 | 0.005573 | 3.52E-04 | 0.607975 | 0.051135 | -0.22533 | 0.260411 | LUC7L |
| ILMN_1776334 | 0.00382 | 1.30E-04 | 0.298377 | -0.04116 | -0.26983 | 0.127686 | UXS1 |
| ILMN_2277676 | 0.010654 | 0.001151 | 0.171853 | -0.07581 | -0.22376 | 0.075773 | ERCC1 |
| ILMN_1699254 | 0.005009 | 2.70E-04 | -0.46961 | 0.239406 | 0.170197 | -0.55892 | PLEKHH1 |
| ILMN_1792860 | 0.009786 | 9.95E-04 | 0.869013 | -0.24303 | -0.27823 | 0.008276 | MED22 |
| ILMN_1697218 | 0.047095 | 0.010691 | 0.278941 | 0.148796 | -0.12211 | -0.10363 | MED22 |
| ILMN_1672128 | 0.015673 | 0.002138 | 0.235909 | 0.043338 | -0.28527 | -0.00384 | ATF4 |
| ILMN_1811313 | 0.005594 | 3.55E-04 | -0.02405 | 0.073737 | 0.03543 | -0.6431 | SLIT3 |
| ILMN_1693338 | 0.025731 | 0.004472 | -0.352 | 0.265446 | 0.004978 | -0.24935 | CYP1B1 |
| ILMN_1790230 | 0.004944 | 2.59E-04 | 0.345311 | 0.227682 | -0.33614 | 0.09222 | ZNF181 |
| ILMN_1751330 | 0.02116 | 0.003327 | 0.381569 | 0.076728 | -0.1958 | -0.11042 | RBCK1 |
| ILMN_1777214 | 0.031113 | 0.005916 | 0.276969 | -0.10256 | -0.16611 | -0.13963 | C2orf67 |
| ILMN_2388445 | 0.008849 | 8.47E-04 | 0.39981 | -0.20286 | -0.23512 | 0.171704 | DLX1 |
| ILMN_1734762 | 0.033736 | 0.006642 | -0.14007 | 0.044118 | -0.34252 | 0.906131 | OPA3 |
| ILMN_1704236 | 0.048324 | 0.011102 | 0.061112 | 0.121336 | -0.17588 | 0.074771 | MAX |
| ILMN_1719224 | 0.008704 | 8.17E-04 | 0.335714 | 0.035937 | -0.11514 | -0.00882 | C17orf45 |
| ILMN_1696933 | 0.004034 | 1.51E-04 | 0.232771 | 0.075413 | -0.20452 | 0.129296 | NLRP3 |
| ILMN_1663532 | 0.002205 | 2.88E-05 | 0.125656 | -0.16391 | 0.124355 | -1.26856 | RIC8B |
| ILMN_1769926 | 0.010072 | 0.001044 | 0.270831 | 0.103149 | -0.27712 | -0.09427 | DBN1 |
| ILMN_1805371 | 0.033006 | 0.00643 | 0.230496 | -0.14265 | -0.03685 | -0.16235 | HNRPM |
| ILMN_1783682 | 0.008197 | 7.35E-04 | 0.341692 | 0.015141 | -0.37591 | 0.371048 | C17orf82 |
| ILMN_1737406 | 2.51E-04 | 4.97E-07 | 0.194606 | 0.033045 | -0.37293 | 1.145515 | KLF6 |
| ILMN_3243185 | 0.00727 | 6.02E-04 | 0.283612 | 0.110756 | -0.19398 | -0.09963 | RERGL |
| ILMN_2061043 | 0.006253 | 4.43E-04 | -0.42442 | 0.763843 | -0.33041 | -0.46559 | CD48 |
| ILMN_2400183 | 0.004063 | 1.58E-04 | 0.408526 | -0.05333 | -0.23878 | 0.044415 | DPH5 |
| ILMN_1656487 | 0.002863 | 5.31E-05 | 0.377626 | -0.13275 | -0.37858 | 0.20976 | FGA |
| ILMN_1779492 | 0.00943 | 9.42E-04 | 0.261711 | -0.11174 | -0.35679 | 0.094015 | SMURF1 |
| ILMN_1704621 | 0.033434 | 0.006543 | 0.241859 | -0.00662 | -0.13534 | 0.048225 | WDR44 |
| ILMN_1792990 | 0.003277 | 7.84E-05 | 0.424089 | -0.53229 | 0.155185 | -0.48921 | ZNF202 |
| ILMN_1761778 | 0.005564 | 3.51E-04 | 0.375571 | -0.01429 | -0.40361 | 0.04195 | TNFSF8 |
| ILMN_2086064 | 0.005029 | 2.73E-04 | 0.230657 | -0.10685 | -0.29923 | 0.049779 | SNRPC |
| ILMN_2079488 | 0.013166 | 0.001624 | 0.213569 | 0.247838 | -0.13205 | 0.09453 | GNPDA2 |
| ILMN_1778629 | 0.020283 | 0.003136 | 0.439462 | 0.079004 | -0.32268 | 0.554213 | RTEL1 |
| ILMN_1686152 | 0.03731 | 0.007617 | 0.195273 | 0.039897 | -0.24249 | 0.047413 | GGA2 |
| ILMN_2400874 | 0.016154 | 0.002234 | 0.312987 | 0.110776 | -0.23472 | 0.136787 | SCYL1 |
| ILMN_2105919 | 0.006311 | 4.51E-04 | 0.202929 | -0.01474 | -0.22544 | 0.058314 | FGF2 |
| ILMN_1803367 | 0.025891 | 0.004527 | 0.098501 | -0.11289 | 0.03695 | -0.84762 | EVI1 |
| ILMN_1719309 | 0.00499 | 2.67E-04 | 0.381393 | -0.00753 | -0.15234 | 0.266689 | LRRC39 |
| ILMN_2211085 | 0.009786 | 9.96E-04 | 0.42962 | 0.276079 | -0.24362 | 0.11102 | EML5 |
| ILMN_1729980 | 0.012752 | 0.001552 | 0.192714 | -0.00456 | -0.18309 | 0.279296 | RNF216 |
| ILMN_1802371 | 0.009355 | 9.28E-04 | 0.114194 | 0.193282 | -0.27353 | 0.271523 | RNF216 |
| ILMN_1806249 | 0.0065 | 4.88E-04 | 0.308229 | 0.06088 | -0.30137 | 0.143745 | IL1RN |
| ILMN_1663916 | 0.027573 | 0.004938 | 0.113883 | 0.371625 | -0.22167 | -0.68373 | ARHGAP9 |
| ILMN_1676745 | 0.02691 | 0.004765 | 0.298191 | 0.163448 | -0.13548 | -0.04829 | ZNF142 |
| ILMN_1800619 | 0.018247 | 0.002677 | 0.274932 | 0.05043 | -0.21571 | 0.155447 | BRI3BP |
| ILMN_1797693 | 0.004179 | 1.72E-04 | 0.25217 | -0.04213 | -0.41514 | -0.08712 | BRI3BP |
| ILMN_2173909 | 0.039503 | 0.008263 | 0.290234 | -0.07897 | -0.15283 | 0.046833 | ZNF14 |
| ILMN_1781769 | 0.024704 | 0.004204 | -0.10953 | 0.057019 | 0.409827 | -0.1003 | CCRL1 |
| ILMN_2106331 | 0.007015 | 5.64E-04 | 0.193505 | -0.45418 | -0.03183 | 0.013206 | H3F3A |
| ILMN_1699632 | 0.022164 | 0.003592 | 0.254075 | 0.046688 | -0.28275 | 0.137167 | RANBP3 |
| ILMN_1730101 | 0.046216 | 0.010391 | -0.19467 | -0.43743 | 0.155725 | -0.51509 | GSPT2 |
| ILMN_1753795 | 0.018131 | 0.002646 | -0.29869 | -0.44263 | 0.363433 | -0.28698 | SOCS4 |
| ILMN_1685631 | 0.022533 | 0.003683 | 0.206711 | 0.045491 | -0.36042 | 0.235671 | KIAA0892 |
| ILMN_2330861 | 0.020749 | 0.003247 | 0.351664 | 0.076171 | -0.19399 | -0.02388 | SMC4 |
| ILMN_1720996 | 0.045966 | 0.010307 | -0.51524 | -0.72471 | 0.397789 | -0.52797 | SLC12A2 |
| ILMN_1711289 | 0.04561 | 0.010194 | 0.129608 | -0.14665 | -0.3028 | -0.02305 | GYS1 |
| ILMN_1768773 | 0.007273 | 6.03E-04 | -0.34432 | -0.21276 | 0.178347 | -0.66499 | EGLN2 |
| ILMN_1738124 | 0.007172 | 5.89E-04 | 0.298364 | 0.094481 | -0.22393 | -0.06999 | ZNF772 |
| ILMN_2385191 | 0.020207 | 0.003116 | 0.155215 | 0.115218 | -0.17685 | 0.209844 | QRICH1 |
| ILMN_1759154 | 0.003802 | 1.29E-04 | 0.116352 | -0.35195 | 0.370141 | -0.62612 | PABPN1 |
| ILMN_1761260 | 0.017103 | 0.002428 | 0.333478 | -0.07972 | -0.17715 | -0.11776 | COBLL1 |
| ILMN_2084391 | 0.002133 | 2.64E-05 | 0.22324 | 0.020754 | -0.30304 | 0.142304 | RAD18 |
| ILMN_2270909 | 0.013522 | 0.001689 | 0.659539 | -0.107 | -0.19115 | 0.237573 | ICA1 |
| ILMN_2365569 | 0.025531 | 0.004422 | -0.36356 | -0.63909 | -0.03535 | 0.286225 | ICA1 |
| ILMN_3251428 | 0.006447 | 4.74E-04 | 0.355748 | 0.027957 | -0.28712 | 0.152496 | MAN1C1 |
| ILMN_1657509 | 0.022111 | 0.003579 | -0.09373 | 0.21117 | 0.107965 | -1.11159 | TSEN54 |
| ILMN_1679891 | 0.002024 | 2.32E-05 | 0.496067 | 0.018502 | -0.21151 | 0.162609 | NAF1 |
| ILMN_1705261 | 0.027858 | 0.005014 | 0.253693 | 0.101616 | -0.17882 | -0.20731 | CAPN1 |
| ILMN_1789504 | 0.003279 | 8.15E-05 | 0.382352 | -0.03798 | -0.2891 | -0.11138 | PTPN9 |
| ILMN_2389501 | 0.004068 | 1.61E-04 | 0.248054 | 0.151142 | -0.18339 | 0.329698 | HSD11B1 |
| ILMN_1749466 | 0.004944 | 2.60E-04 | 0.402787 | 0.02173 | -0.23503 | 0.123497 | VAT1L |
| ILMN_1693674 | 0.007823 | 6.81E-04 | 0.318374 | 0.20098 | -0.26536 | 0.170562 | TRDN |
| ILMN_2260500 | 0.020305 | 0.00314 | 0.512626 | 0.4736 | -0.39402 | 0.302997 | KIAA0319L |
| ILMN_1654543 | 0.006723 | 5.24E-04 | 0.273496 | -0.04424 | -0.16098 | 0.273647 | MED6 |
| ILMN_1807123 | 0.004885 | 2.52E-04 | 0.114368 | -0.01395 | -0.2972 | 0.161106 | SPACA1 |
| ILMN_1667977 | 0.033591 | 0.006595 | -0.01063 | -0.16458 | 0.285297 | -0.32167 | TAF1B |
| ILMN_2362832 | 0.002298 | 3.06E-05 | -0.30967 | 0.198958 | 0.542213 | -1.17687 | STAG3L1 |
| ILMN_3247256 | 0.009835 | 0.001004 | 0.11454 | 0.494975 | 0.10032 | -0.82396 | LOC646996 |
| ILMN_1732166 | 0.042698 | 0.009244 | -0.31217 | 0.052905 | 0.214773 | -0.20155 | SDHAP3 |
| ILMN_2401258 | 0.005113 | 2.87E-04 | 0.261206 | 0.068279 | -0.28946 | -0.02137 | FAM13A |
| ILMN_1815745 | 0.033885 | 0.006681 | -0.21493 | -0.13685 | 0.002349 | -0.88003 | SOX4 |
| ILMN_1738406 | 0.004063 | 1.57E-04 | 0.293108 | -0.07358 | -0.38419 | 0.079902 | SLC2A9 |
| ILMN_2321485 | 0.019511 | 0.002934 | -0.37966 | -0.85499 | -0.1468 | 0.473823 | PPP1R8 |
| ILMN_1718949 | 0.003027 | 6.19E-05 | 0.413416 | -0.07676 | -0.27065 | 0.297291 | SLC39A12 |
| ILMN_1676215 | 0.008204 | 7.37E-04 | 0.520501 | -0.62483 | 0.196511 | -0.69792 | DLG2 |
| ILMN_2132599 | 0.019864 | 0.00302 | 0.131981 | -0.06457 | -0.26832 | -0.02022 | ANKRD22 |
| ILMN_2393456 | 0.037397 | 0.007648 | 0.112081 | -0.47537 | -0.22451 | 0.760156 | DDO |
| ILMN_1710150 | 0.024986 | 0.004278 | -0.12437 | 0.047562 | 0.112852 | -0.67073 | EED |
| ILMN_1796813 | 0.01969 | 0.002976 | -0.0152 | -0.07902 | 0.259542 | -0.59027 | EED |
| ILMN_1722732 | 0.010986 | 0.001215 | 0.349853 | -0.16937 | -0.25201 | 0.040609 | TENC1 |
| ILMN_3239440 | 0.011084 | 0.001232 | 0.025067 | 0.040122 | -0.2102 | 0.158503 | GAGE10 |
| ILMN_1727288 | 0.003871 | 1.35E-04 | 0.315858 | -0.02283 | -0.36744 | -0.14414 | EVPL |
| ILMN_1733110 | 0.002418 | 3.40E-05 | 0.328398 | 0.038042 | -0.35099 | -0.14631 | RASSF7 |
| ILMN_1806956 | 0.021006 | 0.003299 | 0.263541 | 0.041598 | -0.2007 | 0.134745 | ATAD5 |
| ILMN_2390114 | 0.034416 | 0.006835 | -0.60712 | -0.04673 | 0.072372 | -0.62196 | AP3D1 |
| ILMN_1715500 | 0.010134 | 0.001058 | 0.570641 | -0.04427 | -0.17019 | 0.053747 | SS18L1 |
| ILMN_1730601 | 0.021326 | 0.003371 | 0.66617 | -0.05969 | -0.27321 | 0.080835 | FAM194A |
| ILMN_1720440 | 0.01412 | 0.001815 | -0.10808 | -0.58802 | 0.282791 | -0.48477 | HELQ |
| ILMN_1732176 | 0.015383 | 0.002085 | 0.465469 | -0.44032 | 0.416433 | -0.75926 | AGPAT2 |
| ILMN_1773735 | 0.018578 | 0.002743 | 0.457499 | -0.01613 | -0.11551 | 0.20521 | FLJ44635 |
| ILMN_1721127 | 0.002167 | 2.76E-05 | 0.387329 | -0.00644 | -0.35837 | 0.13886 | HIST1H3D |
| ILMN_1793537 | 0.043914 | 0.009656 | 0.010612 | -0.38384 | 0.065445 | -0.96984 | MUC15 |
| ILMN_3243677 | 0.008479 | 7.81E-04 | 0.41859 | 0.015761 | -0.25661 | 0.066973 | SNORA73B |
| ILMN_2394210 | 0.010431 | 0.001111 | 0.343273 | 0.054372 | -0.2166 | 0.111311 | SLC26A8 |
| ILMN_1699735 | 0.013178 | 0.001628 | 0.467624 | 0.045072 | -0.18767 | 0.170305 | FGF1 |
| ILMN_1753322 | 0.022533 | 0.003684 | 0.071333 | -0.80537 | 0.207672 | 0.580214 | FGF1 |
| ILMN_2323007 | 0.006786 | 5.35E-04 | -0.06821 | 0.373987 | -0.29853 | -0.22527 | CDH26 |
| ILMN_2094718 | 0.015996 | 0.002204 | 0.320354 | 0.050042 | -0.18079 | 0.059617 | TPT1 |
| ILMN_1810810 | 0.043075 | 0.009372 | 0.321564 | -0.03322 | -0.22045 | -0.01409 | EEF1A1 |
| ILMN_1343291 | 0.006583 | 4.98E-04 | 0.351403 | -0.02436 | -0.12989 | 0.212374 | EEF1A1 |
| ILMN_2393775 | 0.00733 | 6.11E-04 | 0.032426 | -0.69219 | 0.205045 | -0.76237 | PCDHGA3 |
| ILMN_2232936 | 0.005433 | 3.35E-04 | 0.309644 | -0.47191 | -0.10083 | 0.10082 | UQCRH |
| ILMN_1654552 | 0.03042 | 0.005733 | -0.08018 | -0.06966 | 0.062383 | -0.69287 | MRPS31 |
| ILMN_1761061 | 0.007482 | 6.31E-04 | 0.497111 | -0.02331 | -0.34041 | 0.193421 | PAX7 |
| ILMN_3239060 | 0.009742 | 9.86E-04 | 0.27848 | 0.073558 | -0.22484 | 0.040735 | KRBA1 |
| ILMN_1678579 | 0.011914 | 0.001389 | 0.131236 | -0.364 | 0.477227 | -0.48431 | CPT2 |
| ILMN_1679949 | 0.012805 | 0.001565 | 0.217309 | -0.11069 | -0.31973 | 0.041677 | SLC25A23 |
| ILMN_1664978 | 0.040545 | 0.008582 | 0.19055 | -0.06643 | -0.28363 | 0.239733 | TJP2 |
| ILMN_1708529 | 0.005228 | 3.06E-04 | 0.214423 | 0.842079 | -0.21818 | -0.45953 | PAPPA2 |
| ILMN_1809842 | 0.011076 | 0.001231 | -0.22192 | -0.30319 | 0.408127 | -0.62973 | TNNC1 |
| ILMN_1788166 | 0.016634 | 0.002335 | 0.296002 | 3.32E-04 | -0.19541 | 0.012072 | TTK |
| ILMN_1779014 | 0.012379 | 0.001473 | 0.303626 | -0.022 | -0.23291 | -0.04283 | TSPYL1 |
| ILMN_1754912 | 0.022968 | 0.003791 | 0.270977 | 0.030261 | -0.26546 | 0.050174 | GLE1 |
| ILMN_2242533 | 0.004744 | 2.37E-04 | 0.250086 | 0.008248 | -0.31397 | 0.146839 | FAIM |
| ILMN_1753426 | 0.001813 | 1.66E-05 | 0.288029 | 0.044903 | -0.17579 | -0.01911 | KIAA0556 |
| ILMN_3248127 | 0.026489 | 0.004661 | 0.358562 | 0.091119 | -0.19524 | 0.081535 | FAM25C |
| ILMN_1669382 | 0.006666 | 5.14E-04 | 0.339873 | 0.318975 | -0.34511 | 0.754702 | CPLX2 |
| ILMN_1767337 | 0.045056 | 0.010024 | 0.154464 | -0.26359 | 0.124265 | -0.29024 | SFXN5 |
| ILMN_2219131 | 0.010995 | 0.001216 | 1.109588 | -0.50309 | -0.08407 | 0.568565 | RPS15 |
| ILMN_1717934 | 0.02039 | 0.003161 | 0.154814 | 0.118751 | -0.05863 | -0.35857 | SYT11 |
| ILMN_1678244 | 0.017699 | 0.002559 | 0.344818 | -0.08234 | -0.29708 | 0.312834 | EIF4G1 |
| ILMN_1774161 | 0.001516 | 1.27E-05 | 0.340737 | 0.156078 | -0.25911 | -0.00837 | ARL15 |
| ILMN_1812980 | 0.005251 | 3.12E-04 | 0.328863 | -0.03814 | -0.27144 | 0.185297 | OR2H2 |
| ILMN_3235185 | 0.013217 | 0.001638 | 0.266577 | 0.039686 | -0.20524 | 0.060835 | SNRNP200 |
| ILMN_1730816 | 0.003654 | 1.18E-04 | 0.24409 | 0.188994 | -0.31346 | -0.17263 | GPR162 |
| ILMN_1758784 | 0.015184 | 0.002035 | 0.312141 | -0.37468 | -0.10995 | -0.06557 | ATP2C1 |
| ILMN_1714372 | 0.019237 | 0.002881 | 0.526125 | 1.021487 | -0.56121 | 0.509185 | ITSN2 |
| ILMN_1771738 | 0.036068 | 0.007287 | 0.274812 | -0.0451 | -0.26192 | -0.07345 | ARL5A |
| ILMN_1752321 | 0.0352 | 0.007045 | 0.124259 | 0.010128 | -0.34578 | 0.205709 | SFMBT1 |
| ILMN_2358914 | 0.011585 | 0.001328 | 0.036777 | 0.201434 | -0.31429 | 0.016861 | SLC35C2 |
| ILMN_2369286 | 0.003956 | 1.43E-04 | 0.327738 | 0.110603 | -0.23587 | 0.05912 | NME7 |
| ILMN_2399489 | 0.032381 | 0.006267 | -0.04993 | 0.142162 | -0.10715 | 0.198362 | CBFB |
| ILMN_1718607 | 0.005237 | 3.08E-04 | 0.213855 | 0.033004 | -0.32854 | 0.03325 | TSPAN4 |
| ILMN_1776674 | 0.044001 | 0.009688 | -0.25867 | 0.175405 | 0.137114 | -0.24189 | SAC3D1 |
| ILMN_1659257 | 0.004191 | 1.79E-04 | 0.448546 | -0.09008 | -0.31136 | 0.032934 | TNFRSF8 |
| ILMN_1788062 | 0.011331 | 0.001277 | 0.349008 | -0.01078 | -0.15209 | 0.028415 | SH3GL1 |
| ILMN_1784036 | 0.030341 | 0.005708 | 0.270167 | 0.531569 | -0.19554 | 1.031853 | CDH15 |
| ILMN_3246678 | 0.004194 | 1.81E-04 | 0.24602 | 0.107454 | -0.44111 | -0.07821 | NPW |
| ILMN_1713832 | 0.040081 | 0.00843 | 0.359292 | -0.00378 | -0.27776 | 0.165234 | SNORD15B |
| ILMN_1766991 | 0.002521 | 3.77E-05 | 0.4597 | 0.142059 | -0.34112 | 0.311228 | ABRA |
| ILMN_2393149 | 0.005195 | 3.02E-04 | 0.458106 | 0.007306 | -0.28794 | 0.194838 | ALOX15B |
| ILMN_1665909 | 0.020429 | 0.003175 | 0.323986 | -0.01375 | -0.18059 | -0.15346 | LASP1 |
| ILMN_1652512 | 0.013256 | 0.001647 | 0.189169 | 0.28221 | -0.14504 | -0.39792 | C2CD2 |
| ILMN_1769112 | 0.025055 | 0.004296 | 0.3537 | 0.091398 | -0.26566 | 0.085687 | C2orf58 |
| ILMN_1712632 | 0.02493 | 0.004264 | 0.623205 | 0.20475 | -0.27408 | -0.01685 | XDH |
| ILMN_2292576 | 0.004927 | 2.57E-04 | 0.365613 | -0.01582 | -0.21458 | 0.223646 | TMEM151A |
| ILMN_1711585 | 0.010796 | 0.001176 | 0.105265 | 0.038373 | -0.38351 | 0.175066 | KCMF1 |
| ILMN_1720243 | 0.014797 | 0.001953 | 0.324628 | -0.04955 | -0.27112 | 0.109049 | IL25 |
| ILMN_2393765 | 0.026489 | 0.004663 | -0.50479 | 0.950177 | -0.60395 | -0.59854 | IGLL1 |
| ILMN_2041327 | 0.01259 | 0.001519 | 0.203794 | -0.03308 | -0.43192 | -0.07435 | MRPL37 |
| ILMN_1691276 | 0.005564 | 3.51E-04 | 0.29175 | 0.065024 | -0.38012 | -0.11529 | CXXC1 |
| ILMN_1700086 | 0.0051 | 2.84E-04 | 0.341501 | 0.020232 | -0.30722 | 0.151182 | DOK1 |
| ILMN_1657478 | 0.009572 | 9.60E-04 | 0.293977 | -0.041 | -0.2787 | 0.031838 | MAGEL2 |
| ILMN_2205588 | 0.031169 | 0.005935 | 0.290237 | 0.264218 | -0.20441 | -0.02758 | PCDHB6 |
| ILMN_1742073 | 0.001255 | 7.76E-06 | 0.431158 | -0.22181 | -0.31479 | 0.58161 | ADCY1 |
| ILMN_2120555 | 0.040768 | 0.008658 | 0.264144 | 0.388464 | -0.06495 | 0.849012 | ADCY1 |
| ILMN_1653174 | 0.040666 | 0.008623 | 0.079207 | -0.22692 | -0.4494 | 0.408006 | GCNT2 |
| ILMN_1739161 | 0.028474 | 0.005194 | -0.06392 | -0.2774 | 0.137126 | -0.13903 | PPAP2A |
| ILMN_3231823 | 0.007914 | 6.95E-04 | 0.216394 | -0.05427 | -0.28588 | 0.228838 | VENTXP1 |
| ILMN_1674780 | 0.015067 | 0.002008 | 0.265412 | -0.0535 | -0.03107 | -0.2133 | SF3B1 |
| ILMN_1775634 | 0.00554 | 3.48E-04 | 0.138742 | -0.05555 | -0.25369 | 0.118722 | AGTR2 |
| ILMN_1719905 | 0.026846 | 0.004752 | -0.0264 | 0.586421 | -0.27568 | 0.59206 | TLR10 |
| ILMN_1724540 | 0.007531 | 6.38E-04 | -0.0524 | 0.576108 | -0.2598 | -0.55945 | CART1 |
| ILMN_3241987 | 0.024873 | 0.004241 | 0.404311 | 0.101561 | -0.39894 | 0.024983 | DGCR9 |
| ILMN_1771223 | 0.005807 | 3.84E-04 | 0.326708 | -0.02481 | -0.28261 | 0.069945 | PADI2 |
| ILMN_3240321 | 0.016234 | 0.002249 | 0.145561 | 0.116743 | -0.33158 | 0.11808 | AEN |
| ILMN_3241985 | 0.002133 | 2.62E-05 | 0.615271 | 0.102662 | -0.40761 | 0.037902 | SNORA13 |
| ILMN_2075221 | 0.002112 | 2.57E-05 | 0.377127 | -0.01441 | -0.30723 | 0.165251 | C17orf47 |
| ILMN_2202915 | 0.006904 | 5.50E-04 | 0.371767 | -0.04645 | -0.21245 | -0.16788 | FAR2 |
| ILMN_1688614 | 0.012514 | 0.0015 | 0.431551 | 0.013808 | -0.21527 | 0.151916 | GRM5 |
| ILMN_1802053 | 0.006471 | 4.79E-04 | -0.29826 | -0.24226 | 0.23348 | -0.10104 | ZNF91 |
| ILMN_1708147 | 0.036021 | 0.007268 | -0.19604 | -0.11268 | 0.253361 | -0.95021 | TBPL1 |
| ILMN_2125010 | 0.039317 | 0.008208 | 0.058598 | 0.245284 | -0.14469 | -0.39508 | SKAP2 |
| ILMN_2224486 | 0.04338 | 0.009483 | 4.44E-04 | -0.57804 | 0.158415 | -0.01548 | C3orf14 |
| ILMN_2222065 | 0.034897 | 0.006967 | 0.231333 | 0.019089 | -0.05384 | -0.27414 | FZD1 |
| ILMN_3241524 | 0.010781 | 0.001172 | 0.309455 | 0.110351 | -0.21873 | -0.13449 | ZBTB22 |
| ILMN_1732913 | 0.00716 | 5.84E-04 | 0.276807 | -0.00269 | -0.30299 | 0.181335 | LRP6 |
| ILMN_2176467 | 0.015066 | 0.002007 | 0.115043 | 0.431125 | -0.40167 | -0.44471 | COX6B2 |
| ILMN_1695962 | 0.022221 | 0.003609 | 0.288046 | -0.06392 | -0.23935 | 0.045589 | SLC12A9 |
| ILMN_2271584 | 0.013565 | 0.001698 | 0.853136 | -0.26796 | 0.03468 | 0.357012 | FARP1 |
| ILMN_3251132 | 0.040835 | 0.008682 | 0.239075 | 0.059868 | -0.14994 | -0.03662 | TMOD2 |
| ILMN_1766269 | 0.010206 | 0.001072 | 0.040922 | 0.336986 | -0.43755 | 0.652347 | HM13 |
| ILMN_2150258 | 0.01259 | 0.001518 | -0.03114 | -0.01876 | -0.21186 | 0.163033 | ZFP36L2 |
| ILMN_3308693 | 0.016564 | 0.00232 | 0.189004 | 0.031463 | -0.22776 | 0.235774 | MIR1302-6 |
| ILMN_1811515 | 0.040364 | 0.008525 | -0.32612 | 0.146568 | -0.03264 | -0.5415 | DLG3 |
| ILMN_2336728 | 0.005877 | 3.92E-04 | 0.876824 | -0.13014 | -0.185 | 0.13393 | DLG3 |
| ILMN_1799529 | 0.02801 | 0.00506 | -0.16277 | -0.0095 | 0.350323 | 0.032789 | ZNF441 |
| ILMN_1691295 | 2.51E-04 | 4.96E-07 | 0.82315 | 0.02902 | -0.35114 | 0.132741 | PVRL1 |
| ILMN_1695549 | 0.026148 | 0.004588 | 0.46695 | 0.013473 | -0.21697 | 0.826455 | PVRL1 |
| ILMN_2255021 | 0.03734 | 0.007629 | 0.156244 | 0.017945 | -0.28309 | -0.02469 | ARPC2 |
| ILMN_1778327 | 0.006957 | 5.57E-04 | 0.280809 | 0.131905 | -0.19115 | 0.119355 | PCDHB14 |
| ILMN_1664068 | 0.019716 | 0.002983 | 0.274127 | 0.032469 | -0.1846 | -0.09173 | ERGIC1 |
| ILMN_1683998 | 0.010175 | 0.001065 | 0.777196 | -0.00532 | -0.11056 | 0.205132 | CREG2 |
| ILMN_1806349 | 0.005129 | 2.90E-04 | -0.75892 | -0.3896 | 0.484714 | -0.47248 | SLC6A8 |
| ILMN_1748889 | 0.025341 | 0.004373 | -0.1423 | -0.41954 | -0.25548 | 1.007189 | PCDHGA7 |
| ILMN_1661188 | 0.012217 | 0.001441 | 0.29678 | 0.011061 | -0.19898 | 0.296478 | HIPK1 |
| ILMN_1780924 | 0.044203 | 0.009747 | 0.681062 | 0.241561 | -0.22176 | 0.227386 | SLC43A1 |
| ILMN_2363027 | 0.007532 | 6.38E-04 | -0.08878 | 0.35181 | 0.121594 | -0.9961 | RAD51 |
| ILMN_1727738 | 0.017178 | 0.002447 | 0.010742 | 0.374945 | 0.046775 | -0.4807 | RAB33B |
| ILMN_1746206 | 0.010323 | 0.001092 | 0.212009 | 0.112015 | -0.35433 | -0.00273 | AZI1 |
| ILMN_1657884 | 0.004194 | 1.80E-04 | 0.7786 | -0.20911 | -0.29859 | -0.01956 | NME2 |
| ILMN_2175455 | 0.016204 | 0.002243 | 0.385474 | -0.1374 | -0.40402 | 0.148957 | CYP2A13 |
| ILMN_1795876 | 0.018826 | 0.002792 | 0.212967 | 0.025638 | -0.31222 | 0.072775 | GPS1 |
| ILMN_2102951 | 0.004309 | 1.93E-04 | 0.213883 | 0.060951 | -0.32041 | 0.076711 | FAHD2A |
| ILMN_1699644 | 0.011247 | 0.00126 | 0.302694 | -0.03201 | -0.24244 | 0.085722 | mar-03 |
| ILMN_1790008 | 0.021774 | 0.003472 | -0.35563 | 0.105524 | 0.0658 | -0.41174 | CYP2U1 |
| ILMN_1789645 | 0.003446 | 9.57E-05 | 0.331421 | 0.054534 | -0.34675 | 0.26659 | IL12RB1 |
| ILMN_2063114 | 0.01031 | 0.001087 | -0.18961 | -0.05454 | 0.139993 | -0.701 | TAF1D |
| ILMN_3309299 | 0.008413 | 7.69E-04 | 0.336256 | 0.009141 | -0.31318 | -0.09368 | MIR559 |
| ILMN_1782978 | 0.004068 | 1.60E-04 | 0.190784 | 0.133541 | -0.34951 | 0.173499 | GALNT8 |
| ILMN_1799067 | 0.002168 | 2.77E-05 | 0.358524 | 0.011103 | -0.31062 | 0.107752 | TRY1 |
| ILMN_1666057 | 0.002161 | 2.70E-05 | 0.71228 | 0.066052 | -0.17171 | 0.020776 | REEP2 |
| ILMN_1743131 | 0.003256 | 7.49E-05 | 0.405605 | 0.070362 | -0.21253 | 0.094619 | TOX4 |
| ILMN_2411236 | 0.023336 | 0.003881 | 0.316581 | 0.310905 | 0.002422 | -0.56151 | NRCAM |
| ILMN_1652959 | 0.017947 | 0.002609 | 0.626647 | 0.410928 | -0.30575 | 0.145242 | ABCD2 |
| ILMN_1795842 | 0.005246 | 3.09E-04 | 0.161177 | 0.112733 | -0.28794 | 0.187055 | GUCY2D |
| ILMN_2323933 | 0.003243 | 7.44E-05 | 0.634813 | 0.117714 | -0.24933 | 0.122537 | LAIR2 |
| ILMN_1762993 | 0.021727 | 0.003462 | 0.144655 | 0.083158 | -0.19179 | -0.34119 | PIGV |
| ILMN_2119486 | 0.01848 | 0.002724 | 0.104971 | -0.01971 | -0.01778 | -0.50848 | DMTF1 |
| ILMN_3251550 | 0.009127 | 8.94E-04 | 0.222895 | 0.079374 | -0.11198 | 0.404939 | PHLDA1 |
| ILMN_1687978 | 0.030049 | 0.005634 | 0.173782 | -0.46787 | -0.19183 | 0.24052 | PHLDA1 |
| ILMN_3238323 | 0.0051 | 2.84E-04 | 0.564327 | 0.109296 | -0.28055 | 0.214645 | TMEM90A |
| ILMN_2187899 | 0.029828 | 0.005576 | 0.508122 | 0.200881 | -0.44385 | 1.052563 | FLJ45422 |
| ILMN_1740180 | 0.030341 | 0.005709 | -0.01289 | -0.21307 | 0.07884 | -0.30991 | SNX3 |
| ILMN_1790951 | 0.010807 | 0.001178 | -0.39791 | 0.124142 | 0.13354 | -0.44591 | C19orf50 |
| ILMN_1799320 | 0.010315 | 0.001089 | 0.173206 | 0.098745 | -0.379 | 0.023845 | C9orf37 |
| ILMN_2076758 | 0.005564 | 3.51E-04 | 0.388246 | 0.095995 | -0.29492 | 0.184041 | MBD3L2 |
| ILMN_1768062 | 0.025789 | 0.004491 | 0.444485 | -0.37 | -0.01783 | 0.807714 | ADK |
| ILMN_1728844 | 0.038888 | 0.008064 | 0.153677 | -0.04663 | -0.34115 | 0.287383 | PTPRN2 |
| ILMN_1740415 | 0.043333 | 0.009447 | 0.457252 | 0.083924 | -0.14625 | -0.05246 | WFDC3 |
| ILMN_1714599 | 0.02423 | 0.004084 | 0.230898 | 0.039059 | -0.22237 | -0.01587 | CAMLG |
| ILMN_1798885 | 0.006892 | 5.49E-04 | 0.253973 | 0.078266 | -0.22822 | 0.539555 | SLC35A2 |
| ILMN_2042651 | 0.040177 | 0.008466 | 0.229217 | 0.233059 | -0.27115 | 0.073105 | EVI2B |
| ILMN_1795228 | 0.001086 | 5.61E-06 | 0.247902 | 0.043064 | -0.12165 | 0.462131 | ZFAND5 |
| ILMN_1658706 | 0.008557 | 7.94E-04 | 0.44471 | 0.088784 | -0.12408 | -0.31343 | ST6GALNAC2 |
| ILMN_2290808 | 0.002811 | 5.00E-05 | 0.360394 | -0.00791 | -0.2027 | 0.216751 | RPL21 |
| ILMN_1738383 | 0.012121 | 0.001423 | 0.243515 | -0.01655 | -0.20698 | -0.21832 | EEF2 |
| ILMN_1701558 | 0.0038 | 1.28E-04 | 0.652222 | -0.05068 | -0.1435 | -0.30452 | MAP1A |
| ILMN_1671568 | 0.009043 | 8.81E-04 | 0.32145 | 0.030607 | -0.17569 | 0.07587 | ECHDC2 |
| ILMN_1736670 | 4.36E-04 | 1.21E-06 | -0.28456 | 0.078411 | 0.192413 | -0.96468 | PPP1R3C |
| ILMN_1724581 | 0.022122 | 0.003583 | 0.182669 | 0.016291 | -0.35584 | -0.07857 | EDC3 |
| ILMN_1752159 | 0.004928 | 2.57E-04 | 0.664863 | 0.158061 | -0.28313 | 0.134935 | AHNAK |
| ILMN_1739726 | 0.044652 | 0.009875 | -0.11384 | 0.925905 | -0.62322 | 0.122443 | JSRP1 |
| ILMN_1684255 | 0.030278 | 0.005686 | 0.61831 | -0.01038 | -0.26393 | 0.193189 | MYL4 |
| ILMN_3308961 | 0.027937 | 0.005038 | 0.26006 | 0.117692 | -0.26374 | -0.11574 | MIR1974 |
| ILMN_1766565 | 0.01503 | 0.001997 | 0.712264 | 0.046937 | -0.09696 | 0.103292 | KRTAP5-9 |
| ILMN_1789364 | 0.010323 | 0.001092 | -0.37188 | 0.025599 | 0.247471 | -0.52246 | ZNF789 |
| ILMN_2388190 | 5.01E-04 | 1.55E-06 | 0.699558 | -0.11346 | -0.19017 | 0.225436 | SCEL |
| ILMN_1722753 | 0.043153 | 0.009393 | 0.330709 | -0.18261 | -0.15546 | -0.21973 | GJA5 |
| ILMN_1760778 | 0.006521 | 4.91E-04 | 0.20114 | 0.093594 | -0.20878 | 0.227505 | ENG |
| ILMN_1668453 | 0.005647 | 3.63E-04 | 0.312904 | 0.015779 | -0.27137 | 0.216963 | TRIM36 |
| ILMN_1728746 | 0.036476 | 0.007408 | 0.248835 | 0.383875 | -0.2138 | -0.05933 | GAB2 |
| ILMN_1755235 | 0.0065 | 4.88E-04 | 0.291585 | -0.05495 | -0.28599 | 0.322531 | XPO6 |
| ILMN_1766736 | 0.001714 | 1.51E-05 | 0.380355 | -0.10404 | -0.40124 | 0.168917 | BPI |
| ILMN_2209417 | 0.004054 | 1.54E-04 | 0.399331 | -0.14101 | -0.33957 | 0.422953 | ATP4A |
| ILMN_2404320 | 0.00628 | 4.47E-04 | -0.34595 | 0.387841 | 0.440143 | -1.07595 | SNTN |
| ILMN_1685916 | 0.007273 | 6.04E-04 | 0.554895 | 0.044983 | -0.11021 | 0.156682 | KIF2C |
| ILMN_2078466 | 0.019562 | 0.002945 | 0.288329 | 0.018558 | -0.29305 | -0.10928 | DZIP1L |
| ILMN_1779841 | 0.017043 | 0.002415 | 0.167425 | -0.07612 | -0.44118 | -0.0258 | PPP2R1B |
| ILMN_1704656 | 0.021335 | 0.003374 | -0.04558 | -0.01497 | -0.15528 | 0.464591 | PPP2R1B |
| ILMN_1671728 | 0.005128 | 2.89E-04 | 0.27144 | -0.01826 | -0.25714 | 0.103542 | CARD14 |
| ILMN_1679901 | 0.009078 | 8.87E-04 | 0.432511 | 0.163798 | -0.33143 | 0.380923 | PPARG |
| ILMN_1710752 | 0.010196 | 0.00107 | 0.202592 | -0.06055 | -0.41795 | -0.03132 | NAPRT1 |
| ILMN_1761101 | 0.034921 | 0.006976 | 0.363696 | 0.063865 | -0.2549 | 0.208678 | CCDC112 |
| ILMN_3308515 | 0.045415 | 0.010128 | 0.229811 | -0.04287 | -0.29166 | 0.061935 | SNORD116-13 |
| ILMN_2297069 | 0.002863 | 5.33E-05 | -0.48183 | -0.07014 | 0.172246 | -0.7476 | FAM115A |
| ILMN_1655405 | 0.002863 | 5.42E-05 | -0.33774 | 0.204052 | 0.170158 | -0.50857 | SCARF2 |
| ILMN_2364574 | 0.01483 | 0.001961 | 0.404472 | -0.1969 | -0.10802 | 0.239567 | MAPK8 |
| ILMN_1800461 | 0.005733 | 3.74E-04 | 0.16789 | -0.08755 | -0.1988 | 0.099603 | CSNK2B |
| ILMN_1763775 | 0.004727 | 2.35E-04 | 0.540053 | 0.034248 | -0.22627 | 0.090722 | DNAH8 |
| ILMN_1773042 | 0.010561 | 0.001135 | 0.022974 | -0.34004 | 0.552714 | -0.6021 | SGEF |
| ILMN_1792589 | 0.005743 | 3.75E-04 | 0.42677 | 0.202959 | -0.23907 | 0.156754 | PMCHL1 |
| ILMN_1653163 | 0.008719 | 8.20E-04 | -0.18573 | -0.68086 | 0.176957 | -0.04405 | ZSCAN2 |
| ILMN_2049909 | 0.004961 | 2.62E-04 | 0.361088 | -0.03222 | -0.26932 | 0.267791 | HTN3 |
| ILMN_1695041 | 0.012435 | 0.001485 | 0.235533 | -0.17446 | -0.35245 | 0.277535 | GATA6 |
| ILMN_3308828 | 0.002614 | 4.22E-05 | 0.411175 | 0.044641 | -0.28036 | 0.225046 | MIR1253 |
| ILMN_1735908 | 0.016511 | 0.002301 | 0.332639 | 0.144029 | -0.16184 | -0.09668 | UTP15 |
| ILMN_1712959 | 0.001086 | 5.74E-06 | 0.321209 | 0.152898 | -0.42852 | 0.314372 | DUSP2 |
| ILMN_1669177 | 0.010428 | 0.00111 | 0.016032 | 0.213869 | -0.40067 | 0.563007 | DHRS12 |
| ILMN_3240241 | 0.008858 | 8.54E-04 | 0.381055 | -0.01006 | -0.19501 | 0.082147 | TMEM132B |
| ILMN_1706302 | 0.003531 | 1.02E-04 | 0.419652 | 0.085156 | -0.23604 | 0.29299 | ADAM22 |
| ILMN_2224765 | 0.022373 | 0.003655 | 0.62649 | -0.07572 | -0.37267 | 0.059365 | TMEM75 |
| ILMN_1660723 | 0.03081 | 0.005835 | -0.12455 | -0.50574 | 0.128683 | -1.47193 | RDH13 |
| ILMN_1729514 | 0.036355 | 0.007369 | 0.162872 | -0.40438 | -0.2487 | 0.089482 | CMTM4 |
| ILMN_1675770 | 0.004744 | 2.37E-04 | 0.262754 | -0.21305 | -0.32672 | 0.091547 | SEC14L3 |
| ILMN_1713613 | 0.026735 | 0.004727 | 0.211511 | -0.03335 | -0.31497 | -0.09139 | PIAS2 |
| ILMN_1653412 | 0.006506 | 4.89E-04 | 0.070169 | 0.683772 | 0.475096 | -1.23146 | RAXL1 |
| ILMN_1754570 | 0.021185 | 0.003335 | -0.94476 | -0.12197 | 0.341921 | 0.247507 | KCTD8 |
| ILMN_1761875 | 0.005649 | 3.64E-04 | 0.331235 | 0.030534 | -0.25382 | 0.138061 | SH3BGR |
| ILMN_1812312 | 0.01503 | 0.001997 | 0.368 | -0.20969 | -0.04191 | 0.019905 | NDUFS4 |
| ILMN_2066849 | 0.007871 | 6.87E-04 | 0.15676 | 0.06968 | -0.28255 | 0.275551 | FAM26F |
| ILMN_1753182 | 0.00541 | 3.32E-04 | 0.702781 | 0.10881 | -0.24455 | 0.121285 | COL20A1 |
| ILMN_3236816 | 0.007038 | 5.68E-04 | 0.325148 | 0.395199 | -0.29512 | 0.244385 | FLJ16779 |
| ILMN_1677452 | 0.015262 | 0.002058 | -0.07615 | 0.348161 | -0.10005 | -0.32202 | REXO4 |
| ILMN_1697989 | 0.021798 | 0.003479 | 0.577551 | -0.2064 | -0.5471 | 0.297491 | SNORD101 |
| ILMN_1806779 | 0.025924 | 0.004535 | -0.00183 | 0.513185 | -0.09044 | -0.41658 | ZNF155 |
| ILMN_1767360 | 0.045946 | 0.010295 | 0.237083 | -0.47978 | -0.01779 | -0.03036 | IL10RB |
| ILMN_1716424 | 0.02058 | 0.003203 | 0.518234 | -0.11909 | -0.29177 | 0.446317 | PKP1 |
| ILMN_2073520 | 0.003641 | 1.16E-04 | 0.38313 | -0.0194 | -0.28716 | 0.148447 | C2orf21 |
| ILMN_1721011 | 0.002908 | 5.57E-05 | 0.247682 | -6.25E-04 | -0.3359 | 0.158888 | LCN1 |
| ILMN_1815023 | 0.011796 | 0.001367 | -0.27194 | -1.10186 | -0.32066 | 0.778711 | PIM1 |
| ILMN_2145116 | 0.02511 | 0.00431 | 0.131938 | 0.166906 | -0.26491 | -0.04462 | TMEM173 |
| ILMN_1800152 | 0.022062 | 0.003555 | -0.13529 | 0.195441 | -0.31053 | 0.089825 | KIAA1271 |
| ILMN_2373266 | 0.04695 | 0.010638 | 0.096247 | 0.032432 | 0.067702 | -0.44046 | SFRS12 |
| ILMN_2405592 | 0.019626 | 0.002958 | 0.492149 | -0.17807 | -0.50264 | 0.151788 | TMEM93 |
| ILMN_1737163 | 0.047659 | 0.010873 | -0.1696 | 0.142442 | -0.1476 | 0.251331 | SH3BGRL3 |
| ILMN_1671154 | 0.002851 | 5.20E-05 | 0.413679 | 0.009537 | -0.30436 | 0.079959 | TMPRSS13 |
| ILMN_1709937 | 0.001835 | 1.75E-05 | 0.306875 | 0.043165 | -0.29365 | 0.20625 | KCNN4 |
| ILMN_3236169 | 0.025547 | 0.004426 | -0.55037 | 0.498558 | 0.242759 | 0.617804 | MACC1 |
| ILMN_2335319 | 0.00187 | 1.90E-05 | 0.099021 | -0.10441 | -0.27129 | 0.439764 | KCNG3 |
| ILMN_1686954 | 0.01818 | 0.002659 | 0.320769 | 0.074829 | -0.22262 | 0.050701 | RPS10 |
| ILMN_1705843 | 0.011588 | 0.001329 | 0.442942 | -0.01326 | -0.22264 | 0.110909 | CCDC122 |
| ILMN_1812226 | 0.004034 | 1.52E-04 | 0.339319 | -0.01318 | -0.1877 | 0.185942 | ICAM1 |
| ILMN_2322375 | 2.20E-04 | 4.06E-07 | 0.025708 | -0.10297 | -0.21509 | 1.045768 | MAFF |
| ILMN_1700413 | 4.48E-05 | 4.65E-08 | 0.066187 | -0.09258 | -0.36229 | 1.23616 | MAFF |
| ILMN_1680139 | 3.24E-05 | 2.90E-08 | 0.305134 | 0.201329 | -0.25658 | 1.95224 | MAFF |
| ILMN_1672486 | 0.025138 | 0.004317 | -0.1146 | 0.169091 | -0.28589 | 0.285941 | TCF7L2 |
| ILMN_1688475 | 0.039845 | 0.008363 | 0.099725 | 6.31E-04 | -0.27403 | -0.01914 | DGKB |
| ILMN_1711124 | 0.006583 | 4.98E-04 | -0.3278 | -0.47951 | 0.150841 | -1.09834 | MARVELD2 |
| ILMN_1718297 | 0.033677 | 0.006621 | -0.15462 | 0.233374 | 0.003205 | -0.20464 | EML4 |
| ILMN_1776515 | 0.00272 | 4.66E-05 | -0.43623 | -0.25509 | 0.142467 | -1.35803 | MPPE1 |
| ILMN_2384152 | 0.003122 | 6.91E-05 | 0.468499 | 0.071944 | -0.24795 | 0.171226 | EPS8L3 |
| ILMN_1736015 | 0.04493 | 0.009979 | 0.162437 | 0.108213 | -0.23164 | -0.11287 | PHF17 |
| ILMN_1796962 | 0.013846 | 0.001756 | -0.1726 | -0.33614 | 0.206226 | -0.49287 | PPP3R1 |
| ILMN_1732426 | 0.008379 | 7.64E-04 | 0.335228 | 0.04884 | -0.20426 | 0.112892 | CRHR1 |
| ILMN_2396444 | 0.003408 | 9.36E-05 | 0.283985 | 0.035425 | -0.35454 | 0.069862 | CD14 |
| ILMN_1725787 | 6.45E-04 | 2.56E-06 | -0.09137 | -0.16384 | 0.230122 | -0.8323 | RFX1 |
| ILMN_3308916 | 0.005131 | 2.92E-04 | 0.422502 | 0.122611 | -0.29528 | 0.36132 | MIR1267 |
| ILMN_1690259 | 0.020005 | 0.003058 | 0.755471 | 0.393079 | -0.26227 | 1.318697 | RAE1 |
| ILMN_1782412 | 0.003614 | 1.12E-04 | 0.041167 | -0.61374 | -0.00678 | -2.18658 | IRX2 |
| ILMN_1790710 | 0.040886 | 0.008699 | 0.124571 | 0.359001 | -0.36341 | 0.204246 | SKP1A |
| ILMN_1771060 | 0.014283 | 0.001843 | 0.483113 | -0.00839 | -0.30052 | 0.278572 | SNRPD2 |
| ILMN_2143314 | 0.027573 | 0.004938 | 0.271312 | 0.237432 | -0.31736 | 0.321196 | SPIB |
| ILMN_1671911 | 0.045019 | 0.010008 | -0.18581 | -0.13022 | 0.00513 | -1.16381 | MTA1 |
| ILMN_2076014 | 0.014011 | 0.001797 | 0.440907 | 0.177839 | -0.25347 | 0.177836 | C9orf117 |
| ILMN_2412244 | 0.028248 | 0.005135 | 0.637266 | 0.247115 | -0.12132 | 0.358524 | NF2 |
| ILMN_1725169 | 0.008564 | 7.95E-04 | 0.154854 | 0.023683 | 0.024498 | -1.02001 | INTS12 |
| ILMN_1761797 | 0.034219 | 0.006778 | 0.159327 | -0.31848 | 0.033992 | -0.04599 | CSTB |
| ILMN_1798108 | 0.049726 | 0.011533 | 0.182843 | -0.05318 | -0.2763 | 0.012809 | C6orf211 |
| ILMN_1746277 | 0.006583 | 4.99E-04 | 0.4197 | 0.054484 | -0.22996 | 0.154643 | MLLT4 |
| ILMN_1747696 | 0.030716 | 0.005814 | 0.323744 | -0.0919 | -0.15532 | 0.061264 | TPM3 |
| ILMN_3187852 | 0.016234 | 0.002249 | 0.279818 | 0.108481 | -0.1729 | -0.03466 | KIAA1310 |
| ILMN_3234936 | 0.005112 | 2.86E-04 | 0.312073 | 0.002159 | -0.30338 | 0.111374 | EYS |
| ILMN_2277492 | 0.012795 | 0.001563 | 0.545023 | -0.05343 | 0.111099 | -0.33133 | AGL |
| ILMN_2391891 | 0.025807 | 0.004496 | 0.217525 | 0.123783 | -0.22992 | 0.09127 | MAP2K5 |
| ILMN_1654691 | 0.041693 | 0.008965 | 0.038542 | -0.20023 | 0.059991 | -0.43853 | FKBP3 |
| ILMN_1667371 | 0.004255 | 1.88E-04 | 0.054574 | 0.05023 | -0.26722 | 0.202517 | SLA |
| ILMN_1681857 | 0.009799 | 9.98E-04 | 0.126564 | -0.05019 | -0.29045 | 0.207455 | MYL7 |
| ILMN_1678695 | 0.015569 | 0.00212 | 0.276639 | -0.0318 | -0.33714 | 0.409313 | RBBP8 |
| ILMN_1655068 | 0.01339 | 0.001668 | -0.08333 | -0.06957 | -0.34006 | 0.337538 | TOM1L2 |
| ILMN_2072091 | 0.024674 | 0.004191 | 0.129907 | 0.001872 | -0.27231 | -0.13954 | HNRNPUL2 |
| ILMN_1694147 | 0.003279 | 8.31E-05 | 0.439416 | 0.062924 | -0.15369 | 0.029292 | PUS3 |
| ILMN_1769104 | 0.005404 | 3.30E-04 | 0.431244 | 0.05377 | -0.2581 | 0.211897 | ZCCHC10 |
| ILMN_2396272 | 0.028986 | 0.005339 | 0.180146 | 0.15571 | -0.20416 | -0.03485 | PDCD4 |
| ILMN_2140559 | 0.028147 | 0.005098 | 0.41895 | -0.04846 | -0.16235 | -1.2481 | IRX5 |
| ILMN_1663417 | 0.004744 | 2.37E-04 | 0.145102 | 0.069755 | -0.26662 | 0.18832 | C22orf33 |
| ILMN_1725121 | 0.023177 | 0.003842 | -0.32093 | -0.45789 | 0.194325 | -0.50314 | XPO1 |
| ILMN_2288232 | 0.006077 | 4.19E-04 | -1.09954 | -0.63801 | 0.189821 | 0.083149 | PHKB |
| ILMN_1806330 | 0.005712 | 3.71E-04 | 0.370455 | -0.00132 | -0.25424 | 0.044414 | C17orf78 |
| ILMN_2242984 | 0.004191 | 1.76E-04 | 0.249831 | 0.005437 | -0.21518 | 0.119288 | FAM98B |
| ILMN_1700074 | 0.008524 | 7.88E-04 | 0.636052 | -0.27803 | -0.48261 | 0.563864 | SNORA62 |
| ILMN_1767643 | 0.015267 | 0.002064 | 0.600467 | 0.008281 | -0.25917 | 1.036767 | CTRB2 |
| ILMN_1651430 | 0.004626 | 2.25E-04 | 0.35895 | -0.09999 | -0.18826 | 0.1979 | CTRB2 |
| ILMN_1685445 | 0.002998 | 6.02E-05 | 0.125499 | 0.246149 | -0.24717 | -0.37745 | NOTCH2NL |
| ILMN_1720048 | 0.003279 | 7.99E-05 | -0.06534 | 0.267165 | -0.27822 | 0.785588 | CCL2 |
| ILMN_1789349 | 0.018018 | 0.002623 | 0.32466 | -0.00816 | -0.22151 | 0.058205 | UBQLN4 |
| ILMN_1786072 | 0.038997 | 0.008118 | 0.772373 | 0.340068 | -0.23261 | 0.286512 | MEPE |
| ILMN_2259818 | 0.005129 | 2.91E-04 | 0.406732 | -0.02139 | -0.17757 | 0.244487 | MFI2 |
| ILMN_1688113 | 0.006682 | 5.18E-04 | 0.284932 | 0.01958 | -0.29034 | 0.024889 | LOC645974 |
| ILMN_1797155 | 0.010688 | 0.001157 | -0.20051 | -0.1824 | 0.251337 | -1.08832 | C17orf75 |
| ILMN_1750596 | 0.033477 | 0.006558 | -0.27251 | 0.147059 | -0.00666 | -0.59634 | CLUAP1 |
| ILMN_1738938 | 0.048006 | 0.010991 | 0.242291 | 0.001154 | -0.12262 | -0.33196 | TIMM8B |
| ILMN_1763409 | 0.006493 | 4.84E-04 | -0.31024 | -0.87222 | 0.222497 | 0.108823 | LRRC8D |
| ILMN_2053178 | 0.006484 | 4.83E-04 | 0.161641 | -0.02164 | -0.29415 | -0.00969 | ACTG1 |
| ILMN_1742548 | 7.15E-04 | 3.04E-06 | 0.701747 | -0.06006 | -0.25179 | 0.457746 | GFI1B |
| ILMN_2358628 | 0.01009 | 0.001049 | 0.256532 | -0.04965 | -0.33129 | -0.00295 | ADK |
| ILMN_2047480 | 0.014283 | 0.001843 | 0.268275 | -0.01692 | -0.27037 | 0.03441 | CXorf36 |
| ILMN_1789994 | 0.038045 | 0.007823 | 0.56935 | -0.1287 | -0.29826 | -0.00385 | BMP1 |
| ILMN_2349600 | 0.032392 | 0.006271 | 0.316096 | -0.32911 | -0.24666 | 0.084995 | BRD8 |
| ILMN_1757956 | 0.023008 | 0.003804 | 0.484168 | -0.08185 | 0.253163 | -0.51297 | PCGF1 |
| ILMN_1693221 | 0.005381 | 3.26E-04 | 0.319779 | -0.05017 | -0.28412 | 0.175557 | CENPH |
| ILMN_2147440 | 0.010637 | 0.001148 | 0.326728 | 0.095332 | -0.23335 | 0.087364 | MAN2A1 |
| ILMN_3250893 | 0.009137 | 8.96E-04 | 0.249157 | 0.080802 | -0.19697 | 0.006566 | CNNM3 |
| ILMN_1653461 | 0.005713 | 3.71E-04 | 0.389724 | -0.0911 | -0.29659 | 0.184203 | MGC48628 |
| ILMN_2331525 | 0.005365 | 3.24E-04 | 0.336233 | -0.75164 | 0.100436 | -1.28988 | PRKD2 |
| ILMN_1801349 | 0.012923 | 0.001583 | -0.2692 | 0.116341 | 0.327013 | -0.38564 | TAS2R14 |
| ILMN_1803162 | 0.024704 | 0.004204 | 0.375257 | 0.091314 | -0.07804 | -0.1172 | RTEL1 |
| ILMN_1778941 | 0.025055 | 0.004294 | -0.03691 | -0.07277 | 0.116512 | -0.7454 | TCF2 |
| ILMN_1685115 | 0.006193 | 4.34E-04 | 0.304722 | 0.003353 | -0.20453 | 0.075086 | HEXIM1 |
| ILMN_3272378 | 0.009869 | 0.00101 | 0.266435 | 0.042755 | -0.24284 | 0.081722 | EZR |
| ILMN_1735143 | 0.003903 | 1.37E-04 | 0.42021 | 0.095924 | -0.19291 | 0.218243 | C1orf38 |
| ILMN_1710064 | 0.004903 | 2.53E-04 | 0.225443 | 0.063961 | -0.29273 | 0.263094 | SDR16C5 |
| ILMN_2092041 | 0.00308 | 6.52E-05 | 0.425953 | -0.02761 | -0.25931 | 0.219685 | PSCDBP |
| ILMN_2405642 | 0.028226 | 0.005125 | 0.038055 | 0.066184 | -0.21218 | 0.351584 | DHDDS |
| ILMN_1728572 | 0.029238 | 0.00541 | 0.622086 | -0.49613 | 0.206615 | -0.00171 | DOK5 |
| ILMN_1661488 | 0.036711 | 0.007465 | 0.336965 | -0.03961 | -0.20966 | 0.451815 | LOC729905 |
| ILMN_1735743 | 0.015842 | 0.002171 | 0.585168 | 0.259741 | -0.25794 | 0.145915 | FBLN7 |
| ILMN_1752452 | 0.00358 | 1.07E-04 | 0.470378 | 0.007825 | -0.23498 | 0.244356 | CST4 |
| ILMN_1730809 | 0.017498 | 0.002518 | -0.37327 | 0.158628 | -0.0318 | -1.11632 | SLC29A2 |
| ILMN_1798808 | 0.007273 | 6.04E-04 | 0.224501 | -0.00552 | -0.24804 | 0.16332 | FOXH1 |
| ILMN_2390472 | 0.034568 | 0.006873 | -0.14389 | 0.086059 | 0.145747 | -0.57398 | TTC14 |
| ILMN_2168903 | 0.028836 | 0.005298 | 0.500796 | 0.136223 | -0.20011 | -0.08665 | FAM47B |
| ILMN_1805750 | 0.012901 | 0.00158 | 0.28239 | 0.057618 | -0.11859 | -0.07748 | IFITM3 |
| ILMN_3249244 | 3.86E-04 | 1.04E-06 | -0.13918 | 0.284071 | 0.285866 | -1.14829 | TMEM106A |
| ILMN_1791925 | 0.010426 | 0.001109 | 0.375958 | 0.055638 | -0.27286 | 0.177871 | NFAM1 |
| ILMN_1664283 | 0.006786 | 5.34E-04 | 0.048091 | -0.37117 | 0.12332 | -0.85252 | C9orf75 |
| ILMN_2364700 | 0.031637 | 0.006068 | 0.156419 | -0.0436 | -0.16786 | 0.122556 | ENSA |
| ILMN_1747934 | 0.003614 | 1.11E-04 | 0.469094 | 0.174591 | -0.35713 | -0.15178 | ISYNA1 |
| ILMN_2295957 | 0.007029 | 5.66E-04 | 0.251608 | 0.084276 | -0.23538 | 0.231042 | HHLA3 |
| ILMN_2410347 | 0.041617 | 0.008945 | -0.50496 | -0.01791 | -0.0997 | -0.81749 | HHLA3 |
| ILMN_2343361 | 0.004646 | 2.28E-04 | 0.611448 | -0.05486 | -0.16102 | 0.116543 | CMYA3 |
| ILMN_1738631 | 0.02412 | 0.004054 | 0.453659 | 0.255571 | -0.23973 | 0.164782 | LOC338797 |
| ILMN_1719547 | 0.031142 | 0.005926 | 0.457191 | 0.398795 | -0.22911 | -0.14966 | INHBA |
| ILMN_2291407 | 0.008689 | 8.13E-04 | 0.140096 | 0.33082 | -0.35629 | 0.235206 | PDE4DIP |
| ILMN_2097546 | 0.003151 | 7.07E-05 | 0.331424 | 0.031771 | -0.28248 | 0.102103 | NUDC |
| ILMN_3242307 | 0.007254 | 6.00E-04 | 0.573159 | 0.177141 | -0.30367 | 0.291645 | DNM1P35 |
| ILMN_3242038 | 0.012583 | 0.001514 | 0.141336 | 0.099926 | -0.26078 | -0.10699 | GPX8 |
| ILMN_2334242 | 0.019079 | 0.002848 | -0.42223 | -0.13905 | 0.444446 | -0.54216 | CREB1 |
| ILMN_1808272 | 0.016184 | 0.00224 | 0.315443 | 0.027689 | -0.11435 | -0.36141 | KCNJ10 |
| ILMN_2412172 | 0.008197 | 7.35E-04 | 0.355931 | 0.127767 | -0.22292 | 0.050592 | APOBEC3F |
| ILMN_2296950 | 0.005365 | 3.24E-04 | 0.309184 | -0.00909 | -0.36719 | 0.181537 | APOBEC3F |
| ILMN_1720578 | 0.04132 | 0.008838 | -0.23241 | -0.10212 | 0.139617 | -0.45725 | PRAF2 |
| ILMN_2391219 | 0.003917 | 1.39E-04 | 0.375688 | 0.105441 | -0.23987 | 0.176019 | RGR |
| ILMN_1786347 | 0.002067 | 2.41E-05 | -0.09491 | 0.012322 | 0.304798 | -0.65927 | TNPO1 |
| ILMN_3236653 | 0.014868 | 0.001966 | 0.411058 | 0.104623 | -0.28567 | -0.10703 | RNU1-5 |
| ILMN_1655537 | 0.017135 | 0.002436 | -0.01679 | -0.04177 | -0.37323 | 0.167787 | ING1 |
| ILMN_2347999 | 0.015864 | 0.002175 | 0.255972 | 0.163414 | -0.23283 | -0.11223 | IFNAR2 |
| ILMN_1746158 | 0.006221 | 4.37E-04 | 0.358555 | 0.01246 | -0.2631 | -0.00747 | HOXD11 |
| ILMN_3226505 | 0.004529 | 2.14E-04 | 0.124571 | 0.251334 | -0.20638 | -0.01542 | MSL3 |
| ILMN_1744709 | 0.014042 | 0.001803 | 0.024031 | 0.03267 | -0.17934 | 0.060299 | DLG5 |
| ILMN_1718805 | 0.025154 | 0.004323 | 0.172778 | 0.211283 | -0.28894 | -0.04461 | PPP2R3A |
| ILMN_2359601 | 0.049164 | 0.011379 | 0.070402 | 0.260919 | -0.41604 | 0.777785 | CAMK2G |
| ILMN_1742775 | 0.007396 | 6.18E-04 | 0.179515 | 0.121432 | -0.34376 | 0.299316 | ZNRF1 |
| ILMN_1682996 | 0.024057 | 0.004038 | -0.30777 | -0.08228 | 0.242712 | -0.2886 | VWA5A |
| ILMN_1718069 | 0.045531 | 0.010162 | 0.189955 | 0.025962 | -0.15969 | 0.004117 | MIS12 |
| ILMN_1811443 | 0.005182 | 2.98E-04 | 0.450335 | 0.085295 | -0.2124 | 0.196354 | AVP |
| ILMN_2174804 | 0.005608 | 3.57E-04 | 0.343297 | 0.048374 | -0.20303 | 0.217012 | CD300LG |
| ILMN_1702700 | 0.010523 | 0.001128 | 0.509042 | 0.067395 | -0.28282 | 0.058717 | PCBP4 |
| ILMN_1787591 | 0.006289 | 4.48E-04 | 0.257283 | -0.01307 | -0.24683 | 0.051386 | XPA |
| ILMN_2387385 | 0.002205 | 2.87E-05 | 0.352311 | -0.23633 | -0.27591 | 1.104705 | IGFBP1 |
| ILMN_2208435 | 0.001745 | 1.56E-05 | -0.38502 | -0.10349 | 0.461789 | -1.21204 | CLDN16 |
| ILMN_1740976 | 0.01276 | 0.001557 | 0.238807 | -0.10452 | -0.26196 | 0.143983 | NONO |
| ILMN_2158594 | 0.042844 | 0.009291 | 0.05173 | 0.07871 | -0.24653 | -0.01277 | C10orf28 |
| ILMN_1754421 | 0.014218 | 0.001831 | 0.379936 | 0.001576 | -0.18032 | 0.076998 | NDUFAF1 |
| ILMN_2412873 | 0.011611 | 0.001336 | 0.122361 | -0.3156 | 0.080914 | -0.31672 | ZNF37A |
| ILMN_1730986 | 0.017539 | 0.002526 | -0.27332 | -0.04233 | 0.054052 | -0.87413 | MALT1 |
| ILMN_1733931 | 0.004325 | 1.94E-04 | 0.158379 | 0.193522 | -0.2314 | 0.107584 | PDCD6 |
| ILMN_1655974 | 0.047455 | 0.010809 | -0.29063 | -0.57543 | 0.282981 | -0.57792 | PDE6B |
| ILMN_1776852 | 0.006461 | 4.76E-04 | 0.379941 | 0.049928 | -0.19702 | 0.226999 | TUSC5 |
| ILMN_1662192 | 0.00639 | 4.64E-04 | 0.129823 | 0.217573 | -0.00586 | -0.4365 | ZNF248 |
| ILMN_1776038 | 0.013211 | 0.001634 | 0.063593 | 0.054628 | -0.28279 | 0.027341 | UBXN2A |
| ILMN_1782050 | 0.004848 | 2.45E-04 | 0.315604 | -0.15982 | -0.08396 | 0.563103 | CEBPD |
| ILMN_1685433 | 0.018903 | 0.002807 | -0.15803 | 0.120239 | -0.10798 | -1.02636 | COL8A1 |
| ILMN_2330310 | 0.005476 | 3.43E-04 | 0.229777 | 0.015225 | -0.24411 | 0.153139 | OR4L1 |
| ILMN_2350574 | 0.001375 | 9.92E-06 | 0.26419 | 0.067092 | -0.22007 | 0.608338 | MYADM |
| ILMN_2124221 | 0.037822 | 0.007763 | 0.194669 | -0.10002 | -0.24364 | 0.283883 | CLLU1OS |
| ILMN_1652749 | 0.011466 | 0.001296 | -0.38235 | -0.15474 | -0.00993 | 0.546368 | ERF |
| ILMN_1813434 | 0.008237 | 7.46E-04 | 0.48437 | 0.067988 | -0.27145 | 0.074179 | ATP10A |
| ILMN_1730082 | 0.004163 | 1.71E-04 | 0.205577 | 0.018991 | -0.28911 | 0.060337 | RPUSD4 |
| ILMN_2220845 | 0.004376 | 2.00E-04 | 0.144743 | 0.012829 | -0.31771 | 0.178269 | PRSS7 |
| ILMN_2161556 | 0.029763 | 0.005555 | -1.00771 | -0.80077 | 0.03284 | -0.63825 | C12orf49 |
| ILMN_1691299 | 0.003531 | 1.03E-04 | 0.445302 | 0.027405 | -0.18174 | 0.02844 | C1orf192 |
| ILMN_2395913 | 0.033803 | 0.006662 | 0.275689 | 0.055197 | -0.23184 | 0.071907 | ARHGAP11A |
| ILMN_2294751 | 0.042814 | 0.009283 | 0.391786 | 0.069368 | -0.10104 | -0.01437 | ASCC3 |
| ILMN_2333687 | 0.006763 | 5.32E-04 | 0.338696 | -0.11205 | -0.17237 | 0.363625 | CD59 |
| ILMN_1673953 | 0.016056 | 0.002218 | 0.171809 | -0.07536 | -0.23646 | 0.087777 | HRH1 |
| ILMN_1725597 | 0.028418 | 0.005182 | 0.232086 | 0.312535 | -0.249 | -0.74522 | FXYD4 |
| ILMN_1811579 | 0.029035 | 0.005355 | -0.25687 | -0.31833 | 0.145151 | -0.83221 | HOMER3 |
| ILMN_3265365 | 0.026479 | 0.004658 | 0.385806 | -0.53259 | 0.027117 | -0.42698 | CEP78 |
| ILMN_2392674 | 0.047168 | 0.010721 | 0.146732 | 0.088442 | -0.21206 | -0.13992 | PRR3 |
| ILMN_1670379 | 0.013547 | 0.001694 | 0.094229 | 0.285855 | -0.1071 | -0.07562 | ANTXR1 |
| ILMN_2075578 | 0.008726 | 8.22E-04 | 0.327967 | 0.089135 | -0.17453 | 0.148171 | FAM126A |
| ILMN_1708164 | 0.005447 | 3.39E-04 | 0.323601 | 0.067486 | -0.23546 | 0.032031 | EIF3A |
| ILMN_1718275 | 0.00577 | 3.78E-04 | 0.417792 | 0.044917 | -0.2623 | 0.113999 | IL1F7 |
| ILMN_1764694 | 0.032843 | 0.006388 | -0.01826 | 0.060167 | -0.05977 | -0.44976 | ZFP14 |
| ILMN_2335718 | 0.001091 | 6.01E-06 | 0.224525 | -0.05511 | -0.22522 | 0.339346 | HNRNPAB |
| ILMN_1686846 | 0.007054 | 5.71E-04 | -0.1414 | 0.070626 | -0.29445 | 0.692925 | AKAP12 |
| ILMN_3241848 | 0.020798 | 0.00326 | 0.468999 | 0.092206 | -0.19038 | 0.187155 | SNORD111B |
| ILMN_2193325 | 0.004148 | 1.70E-04 | 0.140725 | 0.039471 | -0.25547 | 0.238563 | MMP23B |
| ILMN_3244841 | 0.03357 | 0.006589 | 0.435467 | -0.30978 | -0.16294 | 0.916926 | LOC100132111 |
| ILMN_2370208 | 0.001403 | 1.05E-05 | 0.669812 | -0.01188 | -0.29657 | 0.030344 | CMTM3 |
| ILMN_2397230 | 0.03893 | 0.008096 | -0.02794 | -0.20107 | 0.026992 | -0.40557 | USP16 |
| ILMN_2038777 | 0.04504 | 0.010019 | 0.166372 | 0.01803 | -0.10144 | -0.0632 | ACTB |
| ILMN_1705144 | 0.014041 | 0.001802 | 0.163709 | 0.02608 | -0.18445 | 0.16724 | ULK1 |
| ILMN_1735052 | 0.009165 | 9.00E-04 | 0.271582 | 0.058851 | -0.13647 | 0.17314 | ULK1 |
| ILMN_1752283 | 0.004131 | 1.68E-04 | 0.223566 | 0.045262 | -0.22329 | 0.31542 | ITCH |
| ILMN_2108339 | 0.022164 | 0.003593 | -0.36793 | -0.18671 | 0.271366 | -0.8042 | THUMPD1 |
| ILMN_1676222 | 0.005246 | 3.10E-04 | 0.306009 | -0.0028 | -0.36838 | -0.0813 | C16orf89 |
| ILMN_2397521 | 0.02007 | 0.003081 | 0.006929 | 0.272907 | 0.080906 | 0.794401 | KDM6B |
| ILMN_1698258 | 0.011023 | 0.001223 | 0.158831 | 0.005346 | -0.23929 | 0.190927 | DNAJC8 |
| ILMN_1669692 | 0.003387 | 8.94E-05 | -0.00106 | 0.766565 | -0.47898 | -0.13594 | IKZF3 |
| ILMN_2320250 | 0.021776 | 0.003473 | 0.273225 | 0.058626 | -0.26403 | -0.02198 | NOL6 |
| ILMN_1678437 | 0.005191 | 3.01E-04 | 0.455814 | -0.07512 | -0.18314 | -0.10235 | FRY |
| ILMN_2086105 | 0.02463 | 0.004178 | -0.29942 | -0.07232 | -0.02689 | 0.52744 | SPRY4 |
| ILMN_1722781 | 2.20E-07 | 4.48E-11 | 0.1921 | 0.042687 | -0.51257 | 2.594524 | EGR3 |
| ILMN_2174127 | 0.032666 | 0.006347 | -0.19941 | -0.18214 | 0.219955 | -0.72517 | DCBLD2 |
| ILMN_2117223 | 0.013193 | 0.00163 | 0.164767 | 0.055838 | -0.18237 | 0.01146 | ROD1 |
| ILMN_1712560 | 0.046486 | 0.010479 | -0.22227 | 0.164395 | -0.48434 | 0.828056 | SF1 |
| ILMN_1790781 | 0.023126 | 0.003831 | 0.397271 | 0.106704 | -0.31455 | -0.07974 | DHRS13 |
| ILMN_2364414 | 0.027723 | 0.004987 | 0.756241 | 0.186739 | -0.24415 | 0.098482 | PCDHA6 |
| ILMN_2414014 | 0.008362 | 7.61E-04 | 0.315091 | 0.077288 | -0.24272 | 0.013624 | RBM10 |
| ILMN_1786065 | 0.027303 | 0.00487 | -0.14698 | 0.337442 | 0.392464 | -0.62701 | UHRF1 |
| ILMN_2198185 | 0.022057 | 0.003552 | 0.33111 | 0.026971 | -0.08411 | -0.37976 | CXorf12 |
| ILMN_1801572 | 0.025694 | 0.00446 | 0.104194 | -0.00934 | -0.39415 | -0.19603 | C19orf44 |
| ILMN_1726752 | 0.004123 | 1.66E-04 | 0.493142 | 0.081271 | -0.20325 | 0.164421 | APTX |
| ILMN_1801124 | 0.027273 | 0.004856 | 0.01486 | 0.090314 | 0.403016 | -0.74851 | KIAA1826 |
| ILMN_1702430 | 0.005476 | 3.43E-04 | 0.444162 | -0.02883 | -0.25584 | 0.201404 | SOX30 |
| ILMN_1685722 | 0.043333 | 0.009454 | 0.28184 | -0.02267 | -0.13347 | -0.07727 | EIF4A2 |
| ILMN_1700248 | 0.00727 | 6.02E-04 | 0.242003 | 0.138042 | -0.35836 | -0.12802 | WDR86 |
| ILMN_1743130 | 0.035952 | 0.00725 | 0.858411 | -0.32883 | -0.05087 | 0.46141 | PTGFRN |
| ILMN_1708414 | 0.016869 | 0.002381 | 0.233125 | 0.064505 | -0.24461 | -0.08868 | GNL3L |
| ILMN_1745737 | 0.004148 | 1.69E-04 | 0.096354 | 0.38687 | -0.35054 | 0.451634 | DIS3L2 |
| ILMN_2351029 | 0.001486 | 1.20E-05 | 0.375849 | 0.034057 | -0.31943 | 0.078065 | MTMR2 |
| ILMN_1661861 | 0.002005 | 2.24E-05 | 0.687301 | 0.072085 | -0.18544 | 0.240636 | CSF2 |
| ILMN_2055165 | 0.021306 | 0.003365 | 0.12592 | 0.027827 | -0.19354 | 0.046164 | MRFAP1 |
| ILMN_1800731 | 0.038872 | 0.008059 | 0.294001 | -0.21877 | -0.09481 | 0.077558 | MBD5 |
| ILMN_3245458 | 0.0491 | 0.011354 | 0.271258 | 0.058257 | -0.27515 | -0.04178 | SNORA61 |
| ILMN_2076640 | 0.003568 | 1.05E-04 | 0.154064 | 0.281444 | -0.22032 | 0.214531 | KHDRBS1 |
| ILMN_1671633 | 0.003279 | 8.04E-05 | 0.471974 | 0.139351 | -0.19765 | 0.15582 | EDA |
| ILMN_2367141 | 0.003831 | 1.31E-04 | 0.365888 | 0.13896 | -0.401 | 0.389624 | TCF7 |
| ILMN_2100258 | 0.023425 | 0.003904 | 0.331038 | 0.178203 | -0.11185 | 0.050373 | PCDHB16 |
| ILMN_1790625 | 0.034819 | 0.006943 | -0.29096 | -0.25548 | 0.376935 | -0.40263 | CBX3 |
| ILMN_1789666 | 0.026092 | 0.004574 | 0.319721 | 0.193772 | -0.26435 | 0.07226 | OR7G1 |
| ILMN_1761968 | 0.00577 | 3.79E-04 | 0.12865 | 0.056043 | -0.27767 | 0.128025 | PPP1R14A |
| ILMN_2394264 | 0.004063 | 1.57E-04 | 0.331592 | 0.163068 | -0.17234 | -0.10772 | UBTF |
| ILMN_1676709 | 0.012485 | 0.001494 | 0.053213 | 0.152033 | -0.27827 | 0.308541 | SDK2 |
| ILMN_1774974 | 0.03081 | 0.005834 | -0.1523 | -0.61338 | 0.120471 | -0.20601 | CLUAP1 |
| ILMN_2100834 | 0.004063 | 1.58E-04 | -0.19093 | 0.598096 | 0.195373 | -1.76583 | ZNF528 |
| ILMN_1762134 | 0.02968 | 0.005533 | 0.340965 | 0.085623 | -0.22601 | -0.07125 | ECHDC1 |
| ILMN_2167011 | 0.042935 | 0.009325 | -0.05387 | -0.29208 | 0.239364 | -0.3732 | ECHDC1 |
| ILMN_1779064 | 0.03505 | 0.007012 | -0.83822 | -0.00146 | 0.117709 | -0.14812 | KLC2 |
| ILMN_1674405 | 0.005956 | 4.00E-04 | 0.322636 | 0.138612 | -0.22993 | 0.107798 | CEPT1 |
| ILMN_1805228 | 0.003387 | 9.00E-05 | 0.488555 | 0.076515 | -0.18056 | 0.075122 | LRG1 |
| ILMN_1657095 | 0.011717 | 0.001357 | 0.136085 | -0.0471 | -0.05475 | -0.41497 | STARD3 |
| ILMN_1751161 | 1.23E-04 | 1.51E-07 | -0.8674 | -0.05243 | 0.32076 | -1.45928 | COL7A1 |
| ILMN_1670215 | 0.016412 | 0.002281 | 0.195172 | 0.030172 | -0.20822 | 0.046318 | FLJ46836 |
| ILMN_1735108 | 0.022125 | 0.003584 | 0.223544 | 0.325043 | -0.23319 | -0.08609 | ANKS6 |
| ILMN_2154157 | 0.003743 | 1.22E-04 | 0.392706 | -0.08107 | -0.09438 | 0.167241 | CHRNA3 |
| ILMN_1680673 | 0.010873 | 0.001191 | -0.21501 | -0.79422 | 0.10077 | -0.21387 | NT5DC1 |
| ILMN_3250902 | 0.01898 | 0.002827 | 0.569775 | 0.009906 | -0.1064 | 0.129463 | CLEC2D |
| ILMN_1729288 | 0.011525 | 0.001316 | 0.402492 | 0.766214 | -0.34971 | -0.55699 | C1QTNF6 |
| ILMN_1684368 | 0.020063 | 0.003079 | 0.239959 | 0.060212 | -0.16479 | 0.384743 | C5orf62 |
| ILMN_1786197 | 0.019908 | 0.003038 | 0.226027 | 0.060524 | -0.13854 | 0.085624 | NR2F1 |
| ILMN_2273224 | 0.013646 | 0.001714 | 0.043149 | 0.540273 | 0.200194 | -0.92134 | SLC4A5 |
| ILMN_1667750 | 0.017582 | 0.002539 | 0.479496 | -0.03027 | -0.31207 | 0.38924 | SLC4A5 |
| ILMN_1779333 | 0.004344 | 1.96E-04 | 0.711531 | 0.075082 | -0.18303 | 0.137249 | MSRB3 |
| ILMN_1754913 | 0.010938 | 0.001203 | 0.222401 | -0.11681 | -0.26028 | 0.10477 | WDR88 |
| ILMN_1789266 | 0.025875 | 0.004523 | 0.233827 | 0.053177 | -0.10538 | -0.41713 | CCDC25 |
| ILMN_1793146 | 0.005743 | 3.75E-04 | 0.32575 | -0.08793 | -0.26531 | 0.088 | CCDC25 |
| ILMN_1703718 | 0.024147 | 0.004062 | 0.200141 | -0.04468 | -0.33911 | -0.03418 | CCT7 |
| ILMN_1651346 | 0.004068 | 1.61E-04 | 0.130379 | 0.204776 | -0.02329 | -1.05291 | TICAM2 |
| ILMN_1737394 | 0.005128 | 2.88E-04 | 0.284477 | -0.13985 | -0.34573 | 0.560999 | LMNA |
| ILMN_2041368 | 0.039598 | 0.008298 | 0.329612 | 0.050681 | -0.21435 | 0.012988 | RN7SL1 |
| ILMN_1665923 | 0.005447 | 3.37E-04 | 0.418823 | 0.033451 | -0.24189 | 0.257207 | THEM5 |
| ILMN_1658152 | 0.004255 | 1.86E-04 | 0.197165 | 0.001074 | -0.37985 | -0.17665 | MAP3K10 |
| ILMN_3245707 | 0.03883 | 0.008041 | -0.19606 | -0.1903 | 0.24364 | -0.1948 | RIMKLB |
| ILMN_2101810 | 0.008853 | 8.49E-04 | 0.557322 | -0.05447 | -0.24424 | 0.176639 | ARHGAP12 |
| ILMN_2376859 | 0.02928 | 0.005429 | 0.191522 | -0.26431 | 0.42144 | -0.22821 | PDGFD |
| ILMN_1690352 | 0.007829 | 6.82E-04 | -0.11566 | -0.19155 | 0.162991 | -0.82272 | ADO |
| ILMN_1726421 | 0.02767 | 0.004971 | -0.50576 | -0.1639 | 0.260867 | -0.28627 | METTL9 |
| ILMN_1711318 | 0.005129 | 2.91E-04 | 0.48011 | -0.00468 | -0.24711 | 0.068045 | PHF8 |
| ILMN_1678754 | 0.017718 | 0.002564 | 0.276244 | 0.048733 | -0.24313 | 0.009783 | PFDN2 |
| ILMN_1709399 | 0.033028 | 0.006436 | 0.305345 | -0.02418 | -0.1796 | 0.44161 | SLC25A34 |
| ILMN_2377746 | 0.007646 | 6.54E-04 | 0.268809 | 0.014603 | -0.28363 | -0.0434 | RUNX2 |
| ILMN_1709877 | 0.004651 | 2.29E-04 | 0.716996 | 0.269098 | -0.18671 | 0.425677 | TRIML1 |
| ILMN_1671621 | 0.003853 | 1.34E-04 | 0.364036 | 0.038933 | -0.24467 | 0.140841 | PCMT1 |
| ILMN_1699978 | 0.001853 | 1.85E-05 | 0.309233 | -0.05563 | -0.36384 | 0.158456 | FAM70A |
| ILMN_1725130 | 0.00763 | 6.51E-04 | 0.26769 | 0.099624 | -0.31573 | 0.029147 | FAM50A |
| ILMN_1792256 | 0.033961 | 0.0067 | -0.10001 | -0.14981 | 0.253049 | -0.56479 | TBX2 |
| ILMN_2128967 | 0.037864 | 0.007777 | 0.244336 | -0.01716 | -0.11664 | 0.265853 | C11orf1 |
| ILMN_1691290 | 0.01901 | 0.002835 | 0.064719 | 0.705442 | -0.32114 | -0.02854 | CELSR3 |
| ILMN_1654630 | 0.003851 | 1.33E-04 | 0.32922 | 0.121245 | -0.27802 | 0.160523 | FAM193B |
| ILMN_3237452 | 0.008714 | 8.19E-04 | -0.1912 | -0.28769 | 0.119405 | -1.75829 | C17orf100 |
| ILMN_1652170 | 0.004148 | 1.69E-04 | 0.4117 | 0.029662 | -0.27663 | 0.111278 | LOC649159 |
| ILMN_2197164 | 0.004014 | 1.48E-04 | 0.375014 | -0.0029 | -0.24816 | 0.11399 | TAAR1 |
| ILMN_1675085 | 0.032572 | 0.006326 | -0.00797 | 0.253461 | 0.275651 | -0.54979 | UBA6 |
| ILMN_3241250 | 0.004068 | 1.60E-04 | 0.267121 | 0.239371 | -0.34468 | 0.18947 | FAM18A |
| ILMN_1724349 | 0.001337 | 9.21E-06 | 0.046389 | -0.18447 | -0.31608 | 0.574685 | RGS3 |
| ILMN_3244903 | 0.007532 | 6.38E-04 | 0.457471 | 0.098572 | -0.18684 | 0.172975 | C7orf65 |
| ILMN_3243593 | 0.022715 | 0.003728 | 0.423397 | 0.125487 | -0.31727 | 0.011162 | LOC100008588 |
| ILMN_1759910 | 0.030396 | 0.005722 | 0.057815 | -0.02612 | -0.08644 | -0.57537 | SERPINA5 |
| ILMN_2139061 | 0.004639 | 2.27E-04 | 0.440963 | -0.01189 | -0.28497 | 0.060056 | COPS2 |
| ILMN_2077008 | 0.009259 | 9.13E-04 | 0.46416 | 0.435341 | -0.1892 | 0.136528 | WDR38 |
| ILMN_1776493 | 0.021185 | 0.003338 | 0.416039 | -0.01667 | -0.10239 | -0.0033 | MTUS1 |
| ILMN_1762529 | 0.016511 | 0.002302 | 0.270704 | -0.18405 | -0.36342 | 0.399744 | SLC12A8 |
| ILMN_1805643 | 0.023336 | 0.003883 | 0.225382 | 0.029754 | -0.16463 | -0.00788 | RILPL1 |
| ILMN_1655935 | 0.006493 | 4.85E-04 | 0.532034 | 0.24474 | -0.34675 | 0.136038 | ADCY7 |
| ILMN_1731851 | 0.02786 | 0.005016 | 0.096184 | -0.08362 | -0.23292 | 0.187829 | OXA1L |
| ILMN_1704537 | 0.043488 | 0.009528 | 0.277548 | -0.08357 | -0.10934 | 0.223244 | PHGDH |
| ILMN_2148668 | 0.01768 | 0.002554 | -0.13315 | -0.08847 | 0.20001 | -0.68315 | RCBTB2 |
| ILMN_1784105 | 0.031672 | 0.006076 | -0.22843 | -0.36112 | 0.141244 | -1.27321 | SLC38A7 |
| ILMN_2206224 | 0.04493 | 0.009979 | 0.359086 | 0.03615 | -0.16084 | 0.262836 | OR4M2 |
| ILMN_3247671 | 0.006268 | 4.46E-04 | 0.470631 | -0.03283 | -0.31028 | 0.182657 | SNORD66 |
| ILMN_2343775 | 0.020624 | 0.003221 | 0.204631 | 0.011161 | -0.29049 | -0.02695 | RPL38 |
| ILMN_1724699 | 0.018815 | 0.002789 | -0.41339 | -0.0553 | 0.558015 | -0.36948 | ACAD8 |
| ILMN_2119793 | 0.00589 | 3.95E-04 | 0.265282 | 0.007704 | -0.24183 | 0.183357 | ANKRD34A |
| ILMN_2329625 | 0.023791 | 0.00398 | 0.166598 | -0.43982 | -0.12282 | 0.476148 | PCDH11X |
| ILMN_2176502 | 0.046286 | 0.010412 | -0.36243 | -0.14001 | 0.402034 | -0.96647 | UNC5B |
| ILMN_1692665 | 0.01676 | 0.00236 | -0.31394 | 0.993008 | -0.01731 | 0.941604 | UNC5B |
| ILMN_1708451 | 0.030586 | 0.005777 | 0.425452 | 0.27078 | -0.15779 | 0.978068 | DSCR5 |
| ILMN_1688959 | 0.02209 | 0.00357 | 0.70768 | 0.43916 | -0.43134 | -0.08306 | CD27 |
| ILMN_1799708 | 0.010017 | 0.001033 | 0.380659 | 0.194003 | -0.2222 | 0.064913 | GYPA |
| ILMN_1657639 | 0.00949 | 9.51E-04 | 0.364536 | 0.051448 | -0.2131 | -0.01308 | EDEM3 |
| ILMN_2065299 | 0.037454 | 0.00767 | 0.129067 | 0.103017 | -0.1901 | -0.16753 | EDEM3 |
| ILMN_3243385 | 0.020608 | 0.003212 | 0.439748 | 0.071536 | -0.10563 | -0.10564 | SDR42E1 |
| ILMN_1706644 | 0.004879 | 2.49E-04 | 0.291481 | 0.135032 | -0.2932 | 0.137615 | BDNF |
| ILMN_1657680 | 0.038202 | 0.007868 | 0.365621 | 0.098477 | -0.14478 | 0.097645 | CCDC69 |
| ILMN_2084489 | 0.038919 | 0.008086 | -0.55338 | 0.041326 | 0.224557 | -0.50853 | ZNF595 |
| ILMN_1715181 | 0.011708 | 0.001354 | 0.294913 | 0.014303 | -0.19695 | -0.01792 | ARRDC2 |
| ILMN_1680850 | 0.03137 | 0.005996 | 0.317521 | 0.115029 | -0.18815 | 0.036277 | RECQL |
| ILMN_2367204 | 0.015111 | 0.002018 | 0.124495 | 0.007964 | -0.19142 | 0.227047 | DSCAM |
| ILMN_2349124 | 0.014311 | 0.001856 | 0.039097 | 0.14716 | -0.09494 | 0.620702 | OSBPL2 |
| ILMN_1656482 | 0.016749 | 0.002358 | -0.24726 | -0.07221 | 0.199893 | -0.82484 | OSBPL2 |
| ILMN_1686623 | 0.038091 | 0.007838 | 0.069035 | 0.231983 | -0.21853 | -0.02673 | CSF1R |
| ILMN_1738322 | 0.004191 | 1.76E-04 | 0.480987 | 0.048756 | -0.2165 | -0.02616 | SHROOM1 |
| ILMN_1798129 | 0.011956 | 0.001395 | 0.308596 | 0.113819 | -0.26736 | -0.0668 | C4orf23 |
| ILMN_1710428 | 0.002167 | 2.74E-05 | 0.533641 | 0.055632 | -0.30708 | 0.163982 | CDC2 |
| ILMN_1657283 | 0.025531 | 0.004421 | 0.239887 | -0.05237 | -0.16591 | 0.035602 | ALKBH5 |
| ILMN_1670895 | 0.047956 | 0.010975 | 0.281739 | 0.062048 | -0.09343 | 0.069136 | ZNF207 |
| ILMN_1684923 | 0.008801 | 8.39E-04 | 0.284163 | -0.149 | -0.253 | 0.149358 | ANP32E |
| ILMN_1727753 | 0.010965 | 0.00121 | 1.060895 | 0.049233 | -0.10717 | 0.477983 | CR2 |
| ILMN_2357155 | 0.007764 | 6.73E-04 | 0.591893 | 0.020312 | -0.17733 | 0.494915 | TPO |
| ILMN_1794726 | 0.048662 | 0.011203 | 0.273532 | -0.0755 | -0.01873 | -0.19762 | RNF167 |
| ILMN_2385097 | 0.015531 | 0.002113 | 0.251305 | 0.180539 | -0.22905 | -0.06281 | NDRG3 |
| ILMN_3246830 | 0.008379 | 7.64E-04 | 0.375037 | 0.062871 | -0.23121 | 0.081374 | NCRNA00087 |
| ILMN_1729611 | 0.039506 | 0.008269 | 0.077987 | 0.463007 | 0.232879 | -0.73542 | REEP4 |
| ILMN_1702635 | 0.015023 | 0.001994 | 0.468366 | -0.04009 | -0.02507 | 0.184624 | KLHDC10 |
| ILMN_1693981 | 0.003387 | 9.14E-05 | 0.454589 | -0.06336 | -0.27561 | 0.092098 | SH3TC2 |
| ILMN_1697472 | 0.046116 | 0.010354 | -0.09436 | -0.18392 | -0.47148 | 0.598121 | TM2D3 |
| ILMN_1733874 | 0.002614 | 4.23E-05 | 0.241972 | -0.04931 | -0.42724 | 0.314461 | SH2D2A |
| ILMN_3251643 | 0.001091 | 5.89E-06 | 0.552018 | 0.134675 | -0.14617 | 0.253498 | C1orf194 |
| ILMN_2196078 | 0.006032 | 4.13E-04 | -0.27874 | 0.856926 | -0.46396 | -0.48518 | SLAMF6 |
| ILMN_1711062 | 0.002998 | 6.07E-05 | 0.690096 | -0.4172 | -0.11941 | 1.760072 | MMP19 |
| ILMN_2202930 | 0.026178 | 0.004594 | 0.208255 | 0.118074 | -0.25412 | 0.092194 | PCYT2 |
| ILMN_1769540 | 0.013571 | 0.0017 | -0.04767 | 0.232789 | 0.112388 | -0.67885 | FLJ43276 |
| ILMN_1779648 | 0.015318 | 0.002074 | 0.396012 | -0.1843 | -0.32583 | 0.129999 | HIST3H2A |
| ILMN_2054607 | 0.010739 | 0.001165 | 0.290857 | -0.06673 | -0.23153 | -0.04049 | CYP4V2 |
| ILMN_2250853 | 0.017293 | 0.00247 | -0.75194 | -0.06933 | 0.2028 | -0.99936 | C1orf84 |
| ILMN_1794612 | 0.002418 | 3.41E-05 | 0.244966 | 0.092137 | -0.40477 | -0.12389 | UBA7 |
| ILMN_1661596 | 0.010911 | 0.001198 | 0.249054 | 0.082805 | -0.32568 | -0.0476 | FLVCR1 |
| ILMN_1775542 | 0.018587 | 0.002749 | 0.311564 | 0.642717 | -0.33584 | 0.341626 | FAIM3 |
| ILMN_1794666 | 0.021306 | 0.003364 | 0.248947 | 0.044121 | -0.26895 | 0.204787 | TSSK1B |
| ILMN_1771987 | 0.002745 | 4.72E-05 | 0.208366 | 0.047591 | -0.36095 | -0.07701 | SLC44A2 |
| ILMN_3240160 | 0.013761 | 0.001736 | 0.320861 | 0.080536 | -0.19604 | 0.201441 | PTENP1 |
| ILMN_1755588 | 0.011906 | 0.001387 | 0.799618 | -1.15844 | 0.357494 | 0.776764 | ANKRD47 |
| ILMN_1716869 | 0.00998 | 0.001026 | 0.390738 | 0.018053 | -0.15227 | -0.06747 | GPM6A |
| ILMN_2171588 | 0.027354 | 0.00489 | 0.28896 | 0.124082 | -0.20285 | 0.081485 | SLC6A1 |
| ILMN_1800262 | 0.003502 | 9.99E-05 | 0.355094 | -0.03463 | -0.36096 | 0.125202 | PPFIA3 |
| ILMN_1665257 | 0.001283 | 8.15E-06 | 0.468386 | 0.009896 | -0.34949 | 0.063603 | RIMS2 |
| ILMN_1724915 | 0.005312 | 3.19E-04 | 0.437592 | 0.001254 | -0.33027 | 0.324853 | C6orf191 |
| ILMN_3234775 | 0.008221 | 7.43E-04 | 0.304298 | 0.034464 | 0.040827 | -0.34727 | SLAIN2 |
| ILMN_1758102 | 0.046141 | 0.01037 | -0.14431 | -0.35777 | -0.22262 | 0.618176 | LOC643853 |
| ILMN_2388254 | 0.014504 | 0.001891 | 0.105328 | 0.038157 | -0.38503 | 0.079623 | GCET2 |
| ILMN_1713505 | 0.003888 | 1.36E-04 | 0.030697 | -0.05466 | -0.18014 | 0.715561 | NPC1 |
| ILMN_1720531 | 0.004851 | 2.47E-04 | -0.71617 | -0.65854 | 0.209984 | -1.00713 | JMJD2B |
| ILMN_2129859 | 0.003277 | 7.89E-05 | -0.35187 | -0.79168 | 0.304184 | 0.332415 | FASTKD3 |
| ILMN_3239244 | 0.005247 | 3.11E-04 | 0.43172 | 0.005922 | -0.24894 | 0.217523 | PRAMEF14 |
| ILMN_1806408 | 0.019436 | 0.002917 | 0.277902 | 0.030347 | -0.12206 | 0.083907 | ACADVL |
| ILMN_1736176 | 0.01503 | 0.001996 | 0.294969 | -0.08581 | -0.23677 | -0.05371 | PLK1 |
| ILMN_1756696 | 0.004068 | 1.62E-04 | 0.069514 | -0.01271 | -0.09763 | -0.54815 | USF2 |
| ILMN_3310030 | 0.022172 | 0.003595 | 0.42631 | 0.066805 | -0.20922 | 0.17601 | MIR1265 |
| ILMN_2105960 | 0.013217 | 0.001639 | -0.02709 | 0.041473 | 0.017863 | -0.49639 | KIF12 |
| ILMN_3308410 | 0.009843 | 0.001006 | 0.379392 | 0.073392 | -0.26731 | 0.073749 | MIR451 |
| ILMN_1794163 | 0.038611 | 0.007983 | 0.705634 | -0.17766 | -0.38599 | 0.171102 | TMOD4 |
| ILMN_1778337 | 0.02192 | 0.003518 | 0.457057 | -0.02617 | -0.15587 | -0.45851 | TCF2 |
| ILMN_1763487 | 0.02445 | 0.004126 | 0.150416 | 0.139469 | -0.34226 | 0.684324 | CTLA4 |
| ILMN_2078389 | 0.006811 | 5.39E-04 | -0.11419 | -0.2896 | -0.35456 | 0.88288 | SLC4A2 |
| ILMN_1725297 | 0.006461 | 4.76E-04 | 0.426298 | 0.016797 | -0.25917 | 0.075412 | AMPH |
| ILMN_1658710 | 0.003917 | 1.40E-04 | 0.388683 | -8.93E-04 | -0.31167 | 0.239577 | FAM19A4 |
| ILMN_1754788 | 0.003387 | 9.14E-05 | 0.828792 | 0.085834 | -0.28365 | 0.014387 | GMPR2 |
| ILMN_1777727 | 0.029984 | 0.005613 | 0.333247 | 0.075659 | -0.28555 | 0.114345 | KANK1 |
| ILMN_3249240 | 0.024267 | 0.004092 | -0.51955 | -0.12704 | 0.214832 | -0.31473 | C4orf46 |
| ILMN_1665235 | 0.047215 | 0.010734 | 0.110101 | -0.10884 | -0.18633 | 0.16567 | CRTAP |
| ILMN_2361575 | 0.008417 | 7.71E-04 | -0.09058 | -0.07716 | 0.307134 | -0.80618 | SNX14 |
| ILMN_1704091 | 0.001358 | 9.55E-06 | -0.10021 | -0.17675 | 0.228023 | -1.60078 | DGAT1 |
| ILMN_3247170 | 0.002586 | 4.13E-05 | 0.664525 | 0.352385 | -0.45573 | 0.153914 | SNORD32B |
| ILMN_1769684 | 0.004131 | 1.67E-04 | 0.273438 | 0.150951 | -0.31104 | 0.102522 | LCE1E |
| ILMN_3309799 | 0.00365 | 1.17E-04 | 0.205036 | -0.06683 | -0.2919 | 0.119059 | MIR569 |
| ILMN_1782618 | 0.008833 | 8.44E-04 | -0.69829 | -1.17825 | 0.173442 | -0.31006 | C9orf16 |
| ILMN_1810937 | 0.006808 | 5.38E-04 | 0.326861 | -0.02851 | -0.27033 | 0.04357 | MPZ |
| ILMN_2207533 | 0.015919 | 0.002187 | 0.261742 | 0.103474 | -0.25676 | -0.25591 | RPS17 |
| ILMN_1760647 | 0.022573 | 0.003693 | 0.233971 | 0.087661 | -0.22159 | -0.46137 | HOXA3 |
| ILMN_1743646 | 0.02039 | 0.003162 | 0.097588 | 0.054486 | -0.24777 | 0.087875 | VASP |
| ILMN_1740231 | 0.036093 | 0.007294 | 0.505868 | 0.253369 | -0.41572 | 0.023472 | ELMO1 |
| ILMN_1811277 | 0.03257 | 0.006324 | 0.19523 | 0.340868 | -0.22903 | 0.343815 | TRIM13 |
| ILMN_2245523 | 0.003977 | 1.46E-04 | 0.254495 | -0.01541 | -0.37406 | 0.03984 | TMEM202 |
| ILMN_1742586 | 0.035293 | 0.007079 | 0.279106 | 0.038872 | -0.19327 | 0.234768 | TMPRSS11F |
| ILMN_1658743 | 0.040801 | 0.008673 | 0.342426 | -0.11788 | -0.12017 | 0.364225 | CCNDBP1 |
| ILMN_1654210 | 0.00765 | 6.58E-04 | -0.0714 | 0.868688 | -0.61875 | -1.10035 | CD1C |
| ILMN_1677385 | 0.040556 | 0.008588 | 0.076161 | -0.10216 | 0.470445 | -0.62358 | C8orf40 |
| ILMN_1808650 | 0.005803 | 3.84E-04 | 0.198586 | 0.087075 | -0.21695 | 0.167156 | CYP39A1 |
| ILMN_2395373 | 0.003387 | 8.96E-05 | 0.277432 | 0.496656 | -0.21627 | -0.50844 | GABBR1 |
| ILMN_2181089 | 0.032948 | 0.006416 | 0.233946 | -0.4247 | -0.15162 | -0.00162 | VPRBP |
| ILMN_2319424 | 0.008245 | 7.47E-04 | 0.382058 | 0.034664 | -0.27945 | 0.196148 | GYG2 |
| ILMN_1810045 | 0.043886 | 0.009648 | 0.109568 | 0.25641 | -0.49297 | 0.1717 | NLRP1 |
| ILMN_1673384 | 0.018297 | 0.002687 | 0.245445 | 0.325898 | -0.49949 | 0.513465 | GEMIN7 |
| ILMN_1756676 | 0.030043 | 0.005632 | 0.420322 | 0.11867 | -0.20754 | 0.056611 | PHF19 |
| ILMN_1738983 | 0.002479 | 3.64E-05 | -0.05535 | 0.120496 | 0.312589 | -1.09592 | SIRT5 |
| ILMN_1768567 | 0.025162 | 0.004329 | 0.336369 | 0.075805 | -0.30105 | 0.006354 | OR10S1 |
| ILMN_1803846 | 0.029467 | 0.005475 | 0.393825 | -0.04763 | -0.21711 | 0.188558 | EIF1 |
| ILMN_2271304 | 0.024563 | 0.004156 | 0.591248 | 0.185433 | -0.27623 | 0.728675 | CACNB1 |
| ILMN_1660963 | 0.003999 | 1.47E-04 | 0.470378 | 0.059549 | -0.21239 | 0.247555 | CCIN |
| ILMN_2369785 | 0.003358 | 8.77E-05 | 0.34818 | -0.01029 | -0.25075 | 0.137366 | SNRPD2 |
| ILMN_1794492 | 0.019919 | 0.003041 | 0.142895 | 0.171727 | -0.00176 | -0.62422 | HOXC6 |
| ILMN_1685774 | 0.009652 | 9.72E-04 | 0.155812 | -0.13264 | -0.2516 | 0.018476 | LOC647340 |
| ILMN_1779234 | 0.041292 | 0.00883 | 0.308125 | 0.130309 | -0.28136 | -0.09535 | CXCL6 |
| ILMN_2161577 | 0.004646 | 2.28E-04 | -0.05105 | 0.700606 | -0.35407 | -0.37875 | CXCL6 |
| ILMN_2409298 | 0.014003 | 0.001794 | 0.353089 | -0.10663 | -0.19143 | 0.079962 | NUSAP1 |
| ILMN_1670122 | 0.004255 | 1.89E-04 | 0.258581 | 0.065468 | -0.30894 | 0.151621 | ZNF624 |
| ILMN_1791773 | 0.038219 | 0.007875 | 0.275437 | 0.115442 | -0.20544 | 0.186498 | OR1A2 |
| ILMN_1659823 | 0.030396 | 0.005723 | 0.377844 | 0.063768 | -0.29067 | 0.475388 | PCDH1 |
| ILMN_1728255 | 0.023354 | 0.003888 | 0.619905 | 0.267481 | -0.17047 | 0.117935 | LALBA |
| ILMN_1681223 | 0.0065 | 4.88E-04 | 0.278639 | 0.015846 | -0.26764 | 0.050759 | CACNA1D |
| ILMN_1814022 | 0.006972 | 5.59E-04 | 0.257127 | 0.048374 | -0.29168 | -0.08432 | NR1H3 |
| ILMN_1773228 | 0.006343 | 4.57E-04 | 0.233961 | -0.13643 | -0.13168 | 0.231031 | DLST |
| ILMN_1760542 | 0.007543 | 6.41E-04 | 0.170787 | 0.003232 | -0.19703 | 0.153256 | PSMA1 |
| ILMN_1682567 | 0.004639 | 2.27E-04 | 0.324532 | 0.023472 | -0.23619 | 0.17989 | CCDC106 |
| ILMN_3249598 | 0.006484 | 4.82E-04 | 0.313126 | 0.039541 | -0.29364 | 0.143226 | RGS21 |
| ILMN_1666819 | 0.041402 | 0.008865 | 0.09732 | 0.139404 | -0.19128 | -0.71254 | PHLDB1 |
| ILMN_1701244 | 0.026148 | 0.004587 | 0.253427 | 0.339152 | -0.2766 | -0.08415 | ITFG2 |
| ILMN_2231569 | 0.040622 | 0.008606 | -0.4547 | 0.162055 | 0.196196 | -0.84222 | ATAD3C |
| ILMN_1791114 | 0.034718 | 0.006916 | 0.460709 | 0.018791 | -0.1646 | 0.149625 | CDR1 |
| ILMN_2211065 | 0.009049 | 8.82E-04 | 0.030836 | 0.369944 | -0.39117 | 0.241598 | TMEM91 |
| ILMN_1752340 | 0.0498 | 0.011557 | -0.21086 | -0.29643 | 0.231373 | -0.42962 | ARF5 |
| ILMN_1763382 | 0.003893 | 1.36E-04 | 0.498202 | -0.0129 | -0.28179 | 0.224468 | NPPB |
| ILMN_1796855 | 0.006645 | 5.09E-04 | -0.00698 | 0.222931 | 0.028651 | -0.49809 | TIAL1 |
| ILMN_1702691 | 0.004237 | 1.83E-04 | 0.345123 | 0.067288 | -0.25479 | 0.206965 | TNFAIP3 |
| ILMN_1739374 | 0.01083 | 0.001184 | 0.546363 | 0.001639 | -0.15752 | 0.084565 | NKAIN1 |
| ILMN_3249167 | 0.016444 | 0.002287 | 0.333316 | -0.16873 | -0.38468 | 0.308573 | SNORA63 |
| ILMN_1718621 | 0.00943 | 9.42E-04 | 0.037604 | 1.074069 | -0.10632 | -0.01778 | TSPAN32 |
| ILMN_1668194 | 0.016903 | 0.002388 | 0.496247 | 0.163932 | -0.3647 | -0.12 | LMTK3 |
| ILMN_2387599 | 0.013661 | 0.001719 | 0.278147 | -0.05262 | -0.17693 | 0.178415 | C20orf24 |
| ILMN_1652787 | 0.006122 | 4.23E-04 | 0.343184 | -0.05767 | -0.2343 | 0.091403 | PIK3AP1 |
| ILMN_1790160 | 0.006484 | 4.81E-04 | -0.31614 | -1.07364 | 0.111052 | 0.385052 | KIT |
| ILMN_2048793 | 0.022781 | 0.003743 | 0.200996 | 0.013621 | -0.31364 | -0.19708 | CIAO1 |
| ILMN_1756411 | 0.021977 | 0.003534 | 0.22837 | -0.02031 | -0.33895 | 0.11336 | SHOX2 |
| ILMN_2246894 | 0.011629 | 0.001341 | 0.246916 | 0.066121 | 0.007352 | -0.32283 | EDF1 |
| ILMN_1653861 | 0.016056 | 0.002218 | 0.302417 | 0.061151 | -0.1704 | 0.126563 | SCMH1 |
| ILMN_2398408 | 0.011284 | 0.001265 | 0.042651 | 0.150977 | -0.40579 | 1.026671 | TCEAL1 |
| ILMN_1716678 | 0.006178 | 4.32E-04 | 0.363177 | 0.111445 | -0.1663 | 0.09196 | NPC2 |
| ILMN_1781027 | 0.015146 | 0.00203 | 0.768395 | -0.06878 | -0.41162 | 0.753316 | TSNARE1 |
| ILMN_1788099 | 0.04468 | 0.009888 | 0.330361 | -0.03732 | -0.07067 | 0.322727 | LSM4 |
| ILMN_3308500 | 0.011161 | 0.001245 | 0.726975 | 0.012246 | -0.15355 | 0.228186 | MIR611 |
| ILMN_2345015 | 0.003329 | 8.51E-05 | 0.294873 | -0.05011 | -0.27826 | 0.089037 | PTGES2 |
| ILMN_2064237 | 0.021377 | 0.003382 | 0.343619 | 0.100799 | -0.17881 | 0.02961 | C1orf88 |
| ILMN_1698605 | 0.003279 | 8.11E-05 | 0.358169 | 0.009181 | -0.2958 | 0.084145 | TMEM43 |
| ILMN_1657288 | 2.85E-06 | 1.62E-09 | -1.12132 | -0.64812 | 1.095508 | -1.58346 | GUSBL2 |
| ILMN_1772644 | 0.002133 | 2.63E-05 | 0.232028 | 0.068372 | -0.41768 | -0.00851 | EML3 |
| ILMN_1670746 | 0.006506 | 4.89E-04 | 0.465931 | 0.076643 | -0.21574 | 0.181287 | BOLL |
| ILMN_1798187 | 0.040463 | 0.008554 | 0.283611 | -0.02614 | -0.14404 | -0.08078 | MYST2 |
| ILMN_2085446 | 0.004921 | 2.55E-04 | 0.247782 | 0.131579 | -0.26763 | 0.081638 | ZNF670 |
| ILMN_1740494 | 0.011584 | 0.001327 | 0.192517 | -0.09197 | -0.3284 | 0.093266 | PCDHA11 |
| ILMN_1760714 | 0.003329 | 8.53E-05 | 0.383813 | -0.05287 | -0.43206 | 0.156612 | RPS3 |
| ILMN_1804568 | 0.015673 | 0.002139 | 0.386497 | -0.4214 | 0.060089 | -0.09186 | HOMER1 |
| ILMN_1792432 | 0.004191 | 1.75E-04 | 0.383316 | 0.20235 | -0.13904 | 0.121305 | KIAA1920 |
| ILMN_1794740 | 2.45E-04 | 4.62E-07 | -0.01042 | -0.09769 | 0.219538 | -0.73041 | CD151 |
| ILMN_1783728 | 0.012354 | 0.001467 | -0.11995 | -0.56286 | 0.545654 | 0.166621 | TBRG4 |
| ILMN_1773126 | 0.01007 | 0.001042 | 0.304967 | 0.092561 | -0.19349 | -0.18752 | FANK1 |
| ILMN_1687036 | 0.004072 | 1.62E-04 | 0.31315 | -0.14758 | -0.2205 | 0.059475 | MRPL47 |
| ILMN_1815083 | 0.012795 | 0.001562 | 0.373463 | -0.0159 | -0.20653 | -0.01502 | WHSC2 |
| ILMN_1808634 | 0.028601 | 0.005228 | -0.20141 | 0.137509 | 0.26767 | -0.50769 | TMEM77 |
| ILMN_1709471 | 0.048714 | 0.01122 | 0.221555 | -0.07733 | 0.116866 | -0.12494 | CNTN4 |
| ILMN_2083789 | 3.70E-04 | 9.09E-07 | -0.11253 | -0.31611 | 0.253752 | -1.05693 | C14orf105 |
| ILMN_3236316 | 0.017737 | 0.002568 | 0.377281 | 0.121823 | -0.25255 | 0.159166 | LOC729121 |
| ILMN_2145153 | 0.008747 | 8.25E-04 | 0.432968 | 0.110509 | -0.21978 | 0.139789 | ANKRD35 |
| ILMN_1682197 | 0.037214 | 0.007591 | 0.137541 | -0.01691 | 0.236657 | -0.76854 | NFXL1 |
| ILMN_1669363 | 0.02604 | 0.004564 | 0.397414 | 0.043147 | -0.22014 | 0.15823 | TGM4 |
| ILMN_1798308 | 0.01119 | 0.001252 | -0.2598 | -0.15734 | 0.192142 | -1.01263 | AHSA2 |
| ILMN_1702140 | 0.005113 | 2.86E-04 | 0.397081 | 0.041562 | -0.30582 | 0.02204 | RACGAP1 |
| ILMN_1733462 | 0.003576 | 1.06E-04 | 0.782768 | 0.509101 | -0.19733 | 1.094714 | HSD3B2 |
| ILMN_1716224 | 0.009663 | 9.75E-04 | 0.280428 | -0.02503 | -0.2444 | 0.034019 | STARD4 |
| ILMN_2215025 | 0.003387 | 9.12E-05 | 0.258548 | 0.004846 | -0.27341 | 0.261756 | HORMAD1 |
| ILMN_1729409 | 0.010494 | 0.00112 | 0.428951 | -0.09492 | -0.13481 | 0.144103 | DLC1 |
| ILMN_1651817 | 0.039326 | 0.008215 | -0.38842 | -0.11921 | 0.266191 | -0.45299 | GRIK1 |
| ILMN_1663213 | 0.023281 | 0.003867 | 1.039382 | 0.613609 | -0.18416 | 0.572776 | ITIH5 |
| ILMN_2043007 | 0.041151 | 0.008786 | -0.36476 | 0.095686 | -0.37947 | 0.987257 | DDI2 |
| ILMN_1784774 | 0.046904 | 0.01061 | -0.27715 | 0.579592 | -0.29574 | -0.22875 | P2RY10 |
| ILMN_1702636 | 0.018247 | 0.002675 | 0.940718 | -0.31534 | -0.08917 | 1.110374 | TUBB6 |
| ILMN_1804522 | 0.007922 | 6.97E-04 | 0.36991 | 0.104255 | -0.20073 | 0.049009 | CCDC47 |
| ILMN_2082699 | 0.034305 | 0.006803 | 0.502633 | 0.272028 | -0.21098 | 0.057552 | C8ORFK36 |
| ILMN_3311080 | 0.006396 | 4.66E-04 | 0.88123 | 0.06032 | -0.15652 | 0.212915 | MIR490 |
| ILMN_1657158 | 0.022312 | 0.003633 | 0.220371 | 0.054923 | -0.22014 | 0.326985 | RNF168 |
| ILMN_2152387 | 0.027051 | 0.004795 | 0.261552 | 0.006935 | -0.23458 | 0.00338 | DOCK7 |
| ILMN_3245151 | 0.014292 | 0.001851 | 0.367042 | 0.086852 | -0.16235 | 0.053347 | URB1 |
| ILMN_1747730 | 0.031916 | 0.006152 | 0.194903 | 0.226114 | -0.22072 | -0.28062 | NOTCH2NL |
| ILMN_1774739 | 0.003614 | 1.12E-04 | 0.263108 | 0.08977 | -0.22607 | 0.236257 | MMP14 |
| ILMN_1711174 | 0.029246 | 0.005415 | 0.141355 | 0.145462 | -0.50524 | 0.034102 | SPRR1B |
| ILMN_1705828 | 0.036198 | 0.007322 | 0.109649 | 0.195101 | -0.21648 | -0.17111 | RICTOR |
| ILMN_1784706 | 0.005252 | 3.12E-04 | 0.358359 | 0.193129 | -0.17799 | -0.04251 | GABRE |
| ILMN_2400644 | 0.006056 | 4.15E-04 | 0.288163 | 0.179047 | -0.19705 | 0.232807 | SRGAP3 |
| ILMN_2373982 | 0.003091 | 6.69E-05 | 0.232181 | -0.01153 | -0.25938 | 0.092039 | PICK1 |
| ILMN_1659990 | 0.004563 | 2.19E-04 | 0.273241 | 0.073246 | -0.2604 | 0.206441 | C7orf68 |
| ILMN_1805952 | 0.030429 | 0.005737 | 0.224611 | -0.02631 | -0.23554 | 0.178068 | SYT15 |
| ILMN_1767303 | 0.025425 | 0.004389 | 0.334106 | 0.387007 | -0.26885 | 0.180247 | LOC400965 |
| ILMN_1653771 | 0.025823 | 0.004504 | 0.282684 | 0.047391 | -0.23372 | 0.055345 | WDR63 |
| ILMN_2352303 | 0.00756 | 6.43E-04 | 0.43798 | 0.062664 | -0.18443 | 0.146806 | RASSF2 |
| ILMN_1723277 | 0.023757 | 0.003973 | 0.466883 | -0.09201 | -0.12781 | -0.17847 | MFAP3L |
| ILMN_1779353 | 0.025961 | 0.004544 | -0.66978 | -0.55299 | 0.510982 | -0.6446 | PUS7 |
| ILMN_1667771 | 0.003372 | 8.82E-05 | 0.563385 | -0.4894 | -0.1898 | 1.00049 | GRB10 |
| ILMN_2090567 | 0.026376 | 0.004637 | 0.266666 | 0.021496 | -0.25617 | 0.066347 | THSD1P |
| ILMN_3308966 | 0.038611 | 0.007982 | 0.834955 | 0.288916 | -0.24626 | 0.338914 | MIR1910 |
| ILMN_1756312 | 0.001952 | 2.06E-05 | 0.507307 | -0.10512 | -0.36282 | 0.308322 | SEMA7A |
| ILMN_1652379 | 0.030903 | 0.005869 | -0.22791 | -0.09908 | 0.16335 | -0.58541 | SUCLG2 |
| ILMN_1805665 | 0.006883 | 5.47E-04 | 0.019802 | -0.38364 | 0.020551 | -1.00384 | FLRT3 |
| ILMN_1718877 | 0.006577 | 4.97E-04 | 0.270037 | 0.202575 | -0.22839 | 0.253061 | FSHB |
| ILMN_1786125 | 0.044869 | 0.009953 | 0.182054 | 0.043873 | -0.06451 | -0.73882 | CCNA2 |
| ILMN_2214678 | 0.021664 | 0.003449 | -0.24013 | -0.26995 | 0.227984 | 0.393114 | MXD1 |
| ILMN_1691843 | 0.014157 | 0.001821 | 0.564751 | -0.50418 | -0.34446 | 0.51946 | RNPS1 |
| ILMN_2375386 | 0.017715 | 0.002562 | 0.060278 | 0.059775 | -0.37462 | 0.282921 | RNPS1 |
| ILMN_1705032 | 0.021414 | 0.003392 | 0.297632 | -0.11061 | -0.1884 | 0.404813 | SEH1L |
| ILMN_1802557 | 0.034691 | 0.006907 | 0.220238 | -0.02872 | -0.10779 | 0.049269 | HEBP1 |
| ILMN_2403006 | 0.014757 | 0.001944 | 0.182786 | -0.10411 | -0.20287 | 0.197625 | TJP1 |
| ILMN_2260313 | 0.00474 | 2.36E-04 | 0.309715 | 0.00857 | -0.24532 | 0.162188 | CRYBA2 |
| ILMN_1729314 | 0.012217 | 0.001443 | 0.339815 | 0.189485 | -0.27712 | 0.106618 | PRG2 |
| ILMN_1733675 | 0.00256 | 4.02E-05 | 0.35895 | -0.15943 | -0.11291 | 0.168357 | MPP1 |
| ILMN_1669273 | 0.04885 | 0.011274 | -0.60643 | -0.35791 | 0.315593 | -0.87703 | PPT1 |
| ILMN_2368434 | 0.028659 | 0.00525 | -0.12984 | -0.25591 | -0.23856 | 0.524519 | CUX1 |
| ILMN_1673743 | 0.005129 | 2.90E-04 | 0.365052 | 0.042807 | -0.27909 | 0.156827 | C22orf26 |
| ILMN_3235928 | 0.011616 | 0.001337 | 0.635719 | 0.067341 | -0.20431 | 0.591535 | CYTIP |
| ILMN_2268618 | 0.029777 | 0.00556 | -0.02829 | 0.126978 | -0.26305 | -0.06545 | PHF20L1 |
| ILMN_2361185 | 0.023197 | 0.003849 | 0.084068 | -0.38637 | 0.017907 | -0.24308 | PHF20L1 |
| ILMN_1666933 | 0.034282 | 0.006795 | 0.399572 | -0.12268 | -0.12337 | 0.079212 | ASH2L |
| ILMN_1717639 | 6.21E-10 | 2.93E-14 | 0.172673 | 0.216694 | -0.38767 | 2.242803 | SIK1 |
| ILMN_3240883 | 0.013217 | 0.001639 | 0.248567 | -0.01508 | -0.33833 | 0.047491 | SNORA11C |
| ILMN_2262543 | 0.039297 | 0.008201 | 0.339827 | 0.075285 | -0.15438 | -0.02267 | C20orf7 |
| ILMN_1765109 | 0.017077 | 0.002424 | 0.023466 | 0.823682 | -0.35293 | -0.68734 | TNFRSF25 |
| ILMN_2247226 | 0.002761 | 4.85E-05 | 0.471675 | 0.153327 | 0.038386 | 2.691963 | BAIAP2 |
| ILMN_1652865 | 0.004034 | 1.51E-04 | 0.343316 | 0.017935 | -0.31599 | 0.119837 | BAIAP2 |
| ILMN_1768161 | 0.003028 | 6.29E-05 | 0.401266 | -0.04653 | -0.20626 | 0.262805 | OR8H3 |
| ILMN_1720482 | 0.025859 | 0.004519 | 0.411053 | -0.02571 | -0.14458 | -0.17553 | CEND1 |
| ILMN_1773168 | 0.002614 | 4.24E-05 | 1.107771 | 0.225696 | -0.22499 | -0.04484 | SYCE1 |
| ILMN_1749882 | 0.018682 | 0.002764 | 0.280882 | 0.105143 | -0.17186 | -0.0158 | STRN |
| ILMN_1773148 | 0.004194 | 1.81E-04 | -0.26928 | -0.04624 | 0.188291 | -0.82854 | C11orf61 |
| ILMN_1769412 | 0.045799 | 0.010252 | 0.232905 | -0.04651 | -0.16007 | 0.078993 | RAPGEF1 |
| ILMN_1700915 | 0.023313 | 0.003874 | 0.037334 | 0.124189 | 0.027841 | -0.67958 | BMI1 |
| ILMN_3310965 | 0.004086 | 1.64E-04 | 0.465045 | 0.001657 | -0.18934 | 0.142553 | MIR1274A |
| ILMN_1669599 | 0.006575 | 4.96E-04 | 0.297321 | 0.11651 | -0.23689 | 0.092016 | DENND4C |
| ILMN_1710710 | 0.001381 | 1.01E-05 | 0.409826 | 0.061862 | -0.38806 | -0.12772 | DEDD |
| ILMN_1740749 | 0.029572 | 0.005508 | 0.287874 | 0.076924 | -0.14535 | 0.01833 | RPL7A |
| ILMN_1703412 | 0.031516 | 0.00603 | 0.016875 | -0.12539 | -0.16102 | 0.499628 | LATS2 |
| ILMN_3300358 | 0.006356 | 4.60E-04 | 0.192941 | 0.207464 | 0.128782 | -0.89941 | ZNF84 |
| ILMN_1814333 | 0.046345 | 0.010431 | -0.43488 | -0.55221 | 0.177751 | -0.08933 | SERPINI1 |
| ILMN_1696657 | 0.01262 | 0.001527 | 0.366849 | 0.005392 | -0.24137 | -0.26163 | LRRN2 |
| ILMN_1691364 | 0.020163 | 0.003106 | 0.128317 | 0.074546 | -0.31541 | -0.18032 | STAT1 |
| ILMN_1731991 | 0.017135 | 0.002438 | -0.14672 | -0.90839 | 0.086099 | -0.56363 | SCYL1 |
| ILMN_2369682 | 0.004063 | 1.56E-04 | 0.267534 | -0.02378 | -0.25531 | 0.010717 | HNRPA2B1 |
| ILMN_1682812 | 0.039232 | 0.008184 | -1.1734 | 0.183471 | 0.050005 | 0.221386 | C21orf33 |
| ILMN_2389013 | 0.042906 | 0.009313 | 0.058693 | 0.199047 | 0.306181 | -1.19888 | ADRM1 |
| ILMN_1757106 | 0.006328 | 4.54E-04 | 0.299965 | 0.105071 | -0.26033 | 0.164389 | mar-06 |
| ILMN_1736650 | 0.044869 | 0.009955 | 0.219286 | 0.139167 | -0.19908 | -0.27048 | JMJD2A |
| ILMN_1693290 | 0.003637 | 1.14E-04 | 0.476266 | -0.20847 | -0.22901 | -0.02595 | METT10D |
| ILMN_1722554 | 0.010796 | 0.001176 | 0.486792 | 0.033382 | -0.19322 | 0.228685 | DCD |
| ILMN_1682336 | 0.028666 | 0.005257 | 0.769884 | -0.37168 | -0.12359 | 0.407061 | MASTL |
| ILMN_1658717 | 0.032417 | 0.006279 | 0.355776 | -0.08026 | -0.17267 | -0.15218 | DEPDC5 |
| ILMN_3247737 | 0.003917 | 1.39E-04 | 0.246409 | -0.02777 | -0.31823 | 0.211775 | RFPL1 |
| ILMN_1768101 | 0.017294 | 0.002471 | 0.193739 | 0.189492 | -0.18419 | -0.39879 | HOXB6 |
| ILMN_1667831 | 0.003571 | 1.06E-04 | 0.41632 | -0.0517 | -0.28801 | 0.114667 | HOXB6 |
| ILMN_2365549 | 0.004489 | 2.10E-04 | 0.182114 | 0.106818 | -0.29643 | 0.112825 | BRPF1 |
| ILMN_3244650 | 0.003631 | 1.14E-04 | 0.208686 | -0.11419 | -0.35926 | 0.126549 | FLJ41941 |
| ILMN_2360054 | 0.008876 | 8.57E-04 | 0.301898 | -0.06421 | -0.15997 | 0.024016 | DDR1 |
| ILMN_1803906 | 0.004034 | 1.53E-04 | 0.233057 | 0.038102 | -0.33529 | -0.05184 | ETV2 |
| ILMN_1657009 | 0.043598 | 0.009558 | 0.292236 | -0.4177 | -0.27152 | 0.070624 | NFASC |
| ILMN_1722151 | 0.00358 | 1.07E-04 | 0.132861 | 0.034908 | -0.46831 | 0.245531 | EIF6 |
| ILMN_1661717 | 0.027635 | 0.004959 | 0.116242 | -0.07698 | -0.17229 | 0.210026 | TFDP1 |
| ILMN_1728305 | 0.035315 | 0.00709 | -0.20987 | -0.45736 | 0.023212 | -0.11923 | PUM2 |
| ILMN_2350122 | 5.17E-04 | 1.73E-06 | 0.143561 | -0.07628 | -0.0339 | -0.74355 | TRIM13 |
| ILMN_2262275 | 0.015067 | 0.002008 | -0.38691 | -0.0621 | 0.400995 | -0.6671 | TRIM13 |
| ILMN_1719093 | 0.01336 | 0.001664 | 0.139559 | 0.049708 | -0.23014 | 0.278123 | KLRC4 |
| ILMN_1800952 | 0.003399 | 9.23E-05 | 0.380208 | 0.017551 | -0.28793 | 0.116867 | PSMD11 |
| ILMN_1656557 | 0.006132 | 4.24E-04 | 0.41273 | 0.097089 | -0.26172 | 0.080817 | ARMC8 |
| ILMN_3248094 | 0.019138 | 0.002861 | 0.703133 | -0.05278 | -0.07671 | 0.078381 | ANO2 |
| ILMN_1681304 | 0.011833 | 0.001374 | 0.320964 | 0.121167 | -0.1265 | -0.06628 | PAN3 |
| ILMN_1749078 | 0.00198 | 2.13E-05 | 0.271153 | 0.041886 | -0.26944 | -0.0251 | TIMP2 |
| ILMN_1684553 | 0.003919 | 1.41E-04 | 0.382321 | 0.054068 | -0.29301 | 0.187935 | RHOH |
| ILMN_2374352 | 0.011963 | 0.001398 | 0.178863 | 0.27283 | -0.28485 | -0.22866 | DBNDD1 |
| ILMN_1774066 | 0.049005 | 0.011322 | 0.251623 | -0.02664 | -0.18669 | 0.011916 | TMEM141 |
| ILMN_1736567 | 0.009588 | 9.64E-04 | 0.198918 | -0.04505 | -0.29846 | -0.13538 | CD74 |
| ILMN_2222991 | 0.017718 | 0.002564 | 0.440317 | -0.04489 | -0.2267 | -0.1745 | ETF1 |
| ILMN_1665547 | 0.002417 | 3.33E-05 | 0.34612 | 0.079588 | -0.26904 | 0.157471 | CADPS |
| ILMN_3306993 | 0.004063 | 1.56E-04 | 0.392683 | 0.057855 | -0.30882 | 0.116485 | FZR1 |
| ILMN_1700719 | 0.019864 | 0.003019 | 0.443167 | 0.218585 | -0.38849 | 0.624862 | ST7OT1 |
| ILMN_1666761 | 0.013358 | 0.001663 | -0.08087 | -0.4008 | 0.170755 | -0.45207 | PPP2R5E |
| ILMN_1778709 | 0.012978 | 0.001595 | -0.38357 | 1.06E-04 | 0.268916 | 0.057092 | PICALM |
| ILMN_1669607 | 0.025749 | 0.004478 | 0.171255 | -0.37201 | -0.13394 | -0.69968 | PHKG2 |
| ILMN_3309284 | 0.026632 | 0.004697 | 0.356985 | -0.01895 | -0.25147 | 0.015078 | MIR137 |
| ILMN_1790741 | 0.001872 | 1.92E-05 | 0.372648 | 0.088473 | -0.24651 | 0.127593 | RNF126 |
| ILMN_1681721 | 0.014572 | 0.001906 | 0.46844 | -0.13016 | -0.2577 | 0.677181 | OASL |
| ILMN_2092850 | 0.011494 | 0.001303 | 0.258146 | 0.074354 | -0.21021 | 0.211176 | HPSE |
| ILMN_2405521 | 0.008689 | 8.14E-04 | 0.384745 | -0.12508 | -0.31586 | 0.845594 | MTHFD2 |
| ILMN_1651604 | 0.007944 | 7.00E-04 | 0.325043 | -0.03022 | -0.24535 | 0.019164 | MARK4 |
| ILMN_2389429 | 0.007482 | 6.31E-04 | 0.225709 | -0.08899 | -0.14886 | 0.127816 | DCUN1D4 |
| ILMN_2201580 | 0.00358 | 1.08E-04 | 0.159429 | 0.076177 | -0.51442 | -0.00608 | GSTM2 |
| ILMN_1699362 | 0.021962 | 0.00353 | 0.292496 | 0.077952 | -0.19609 | -0.07185 | IK |
| ILMN_1675462 | 0.04006 | 0.008421 | -0.23209 | -0.14712 | 0.250827 | -0.21069 | LSM6 |
| ILMN_3310291 | 0.002005 | 2.23E-05 | 0.386162 | 0.207853 | -0.41985 | 0.970296 | MIR645 |
| ILMN_1705813 | 0.005 | 2.68E-04 | 0.326734 | 0.068668 | -0.22398 | 0.090877 | CFTR |
| ILMN_1795340 | 0.007647 | 6.56E-04 | 0.185934 | -0.1118 | -0.25301 | 0.143095 | TMPO |
| ILMN_1663502 | 3.71E-04 | 9.37E-07 | 0.69698 | -0.02169 | -0.23399 | 0.204361 | WHSC1 |
| ILMN_1656807 | 0.015041 | 0.002 | 0.251755 | -0.00909 | -0.30899 | 0.116578 | RPL27 |
| ILMN_1742288 | 0.010101 | 0.00105 | 0.168276 | -0.08682 | -0.16598 | 0.027738 | ZNF620 |
| ILMN_1782691 | 0.002586 | 4.13E-05 | 0.504843 | 0.08644 | -0.28453 | 0.227742 | LOC149620 |
| ILMN_1702389 | 0.015673 | 0.002138 | 0.12173 | 0.083958 | -0.3515 | 0.048265 | ZC3H3 |
| ILMN_2412564 | 0.024113 | 0.004052 | 0.101399 | -0.39234 | -0.17168 | 0.367898 | NCBP2 |
| ILMN_1737912 | 0.007848 | 6.84E-04 | 0.543441 | 0.24459 | -0.31885 | 0.255595 | GLRX2 |
| ILMN_1681340 | 0.015236 | 0.002051 | 0.570661 | 0.035568 | -0.15863 | 0.023565 | C1orf41 |
| ILMN_1779857 | 0.005191 | 3.01E-04 | 0.36048 | 0.224216 | -0.22971 | 0.558278 | KLF4 |
| ILMN_2137789 | 0.007649 | 6.58E-04 | 0.377882 | 0.10658 | -0.32526 | 0.473689 | KLF4 |
| ILMN_1668369 | 0.005629 | 3.60E-04 | 0.372266 | 0.062833 | -0.2317 | 0.123357 | CDC37 |
| ILMN_1808777 | 0.030716 | 0.005814 | 0.37499 | -0.02376 | -0.16799 | -0.41926 | EHD2 |
| ILMN_1750029 | 0.00324 | 7.40E-05 | 0.426955 | -0.00389 | -0.20049 | 0.148267 | GABPA |
| ILMN_1706101 | 0.031585 | 0.006053 | 0.247602 | -0.00441 | -0.19781 | 0.208745 | C7orf31 |
| ILMN_1664646 | 0.019716 | 0.002984 | 0.237952 | 0.360447 | -0.16127 | -0.12653 | NSUN6 |
| ILMN_2153547 | 0.020776 | 0.003256 | 0.312002 | 0.021117 | -0.24043 | 0.095909 | OR4C15 |
| ILMN_1675612 | 0.03157 | 0.006046 | -0.08564 | -0.49999 | 0.233915 | -0.82151 | BLCAP |
| ILMN_1802252 | 0.004191 | 1.79E-04 | 0.377444 | -0.06077 | -0.2707 | -0.09024 | GAPDH |
| ILMN_2038778 | 0.03784 | 0.007768 | 0.29547 | -0.07558 | -0.26132 | 0.023753 | GAPDH |
| ILMN_1663106 | 0.029231 | 0.005406 | 0.885805 | 0.081042 | -0.12897 | 0.288259 | SPTB |
| ILMN_2347314 | 0.006 | 4.07E-04 | -0.00862 | -0.35278 | 0.096533 | -0.37224 | ARNT |
| ILMN_1677226 | 0.014283 | 0.001845 | -0.66284 | -0.26149 | -0.02044 | -0.50373 | ARVCF |
| ILMN_2348268 | 0.013379 | 0.001666 | 0.118329 | 0.244022 | -0.1558 | -0.40964 | IFFO1 |
| ILMN_1813120 | 0.013117 | 0.001616 | 0.409577 | -0.1698 | -0.24536 | 0.056406 | MAP4K3 |
| ILMN_1743199 | 0.004034 | 1.51E-04 | 0.355349 | 0.195222 | -0.25156 | 0.21328 | EGR2 |
| ILMN_2195319 | 0.001984 | 2.16E-05 | 0.329273 | 0.120169 | -0.23237 | 0.220543 | PDE6A |
| ILMN_1664833 | 0.011708 | 0.001354 | -0.32334 | 0.342701 | 0.554072 | -1.03376 | MRPL50 |
| ILMN_1673716 | 0.003956 | 1.43E-04 | 0.351701 | -0.06129 | -0.27315 | 0.181796 | KIF6 |
| ILMN_3242856 | 0.006662 | 5.13E-04 | 0.149484 | -0.11811 | -0.32767 | 0.185284 | BEND3 |
| ILMN_1754062 | 0.002851 | 5.20E-05 | 0.161578 | 0.104226 | -0.30246 | 0.218488 | BEND3 |
| ILMN_2334112 | 0.005158 | 2.96E-04 | 0.339889 | -0.17997 | -0.25168 | 0.147493 | WHSC1 |
| ILMN_1681678 | 0.041207 | 0.008808 | 0.200031 | -0.59521 | -0.1058 | 0.246742 | CTDSP1 |
| ILMN_3236491 | 0.011981 | 0.001401 | 0.339766 | 0.155836 | -0.25773 | 0.132395 | FAM57B |
| ILMN_1814688 | 0.045284 | 0.010091 | 0.514957 | -0.17517 | -0.56335 | 0.408966 | ATP8B2 |
| ILMN_2367428 | 0.021003 | 0.003297 | 0.076312 | -0.40289 | -0.11884 | -0.29722 | FAM96A |
| ILMN_1666966 | 0.002536 | 3.86E-05 | 0.685157 | 0.150056 | -0.2501 | 0.176469 | INS |
| ILMN_1706478 | 0.038204 | 0.00787 | -0.36652 | -0.23498 | 0.147147 | -0.70207 | HOXA7 |
| ILMN_1809947 | 0.004446 | 2.06E-04 | 0.189825 | 0.028851 | -0.23203 | 0.332809 | TMEM155 |
| ILMN_2044832 | 0.023818 | 0.003987 | 0.305717 | 9.10E-04 | -0.22116 | 0.020121 | NOP56 |
| ILMN_1772929 | 0.025553 | 0.004428 | 0.283222 | -0.08699 | -0.17174 | 0.222035 | ATP5J |
| ILMN_1653220 | 0.041467 | 0.008889 | -0.12752 | 0.023987 | 0.169955 | -0.87645 | PITPNM1 |
| ILMN_2100437 | 0.027706 | 0.004983 | -0.27391 | -0.13887 | 0.146499 | -1.20831 | HBB |
| ILMN_1746376 | 5.08E-04 | 1.65E-06 | 0.750785 | 0.068256 | -0.45271 | -0.03817 | SCARA3 |
| ILMN_1754660 | 0.041538 | 0.008906 | 0.300697 | -0.22666 | 0.076387 | -0.49113 | ZCCHC24 |
| ILMN_2393046 | 0.022223 | 0.003611 | -0.41082 | -0.22996 | 0.215542 | -0.43048 | NHLRC3 |
| ILMN_1794967 | 0.011297 | 0.001267 | 0.323617 | 0.071777 | -0.24244 | -0.04892 | EIF4ENIF1 |
| ILMN_1654458 | 0.011306 | 0.001272 | 0.365124 | 0.125031 | -0.22753 | 0.11362 | FLJ37587 |
| ILMN_1759195 | 0.018587 | 0.002746 | 0.5823 | 0.029015 | -0.13689 | -0.04361 | C22orf9 |
| ILMN_1806052 | 0.001907 | 1.99E-05 | 0.792481 | 0.106944 | -0.0651 | -0.03222 | UNC119 |
| ILMN_1664698 | 0.038098 | 0.007841 | 0.116925 | 0.274911 | -0.13998 | -0.35653 | UNC119 |
| ILMN_1748852 | 0.021197 | 0.003341 | 0.265591 | -0.05883 | -0.25815 | 0.24831 | KRTAP19-7 |
| ILMN_2226753 | 0.03042 | 0.005733 | -0.49092 | 0.039955 | 0.276246 | -0.26935 | ZC3HAV1L |
| ILMN_2410038 | 0.00532 | 3.19E-04 | 0.324878 | 0.198789 | -0.08761 | -0.45803 | FAM111A |
| ILMN_3251455 | 0.010322 | 0.00109 | 0.273942 | 0.001037 | -0.26035 | 0.13005 | RHOQ |
| ILMN_1661173 | 0.046505 | 0.010488 | -0.54011 | 0.170717 | 0.085442 | -0.03718 | TRIP4 |
| ILMN_1653319 | 0.0038 | 1.28E-04 | 0.275397 | 0.304225 | -0.33702 | 0.118112 | MC1R |
| ILMN_1726591 | 0.004191 | 1.77E-04 | 0.300328 | 0.143173 | -0.33328 | 0.109616 | CARD16 |
| ILMN_1724474 | 0.0038 | 1.28E-04 | 0.352593 | 0.046777 | -0.2664 | 0.168075 | CARD16 |
| ILMN_2112755 | 0.034974 | 0.006989 | -0.4098 | -0.10436 | 0.167726 | -0.34742 | HSDL1 |
| ILMN_2292863 | 0.003329 | 8.55E-05 | 0.348056 | 0.10023 | -0.26873 | -0.19685 | RASA1 |
| ILMN_1761927 | 0.042913 | 0.009319 | 0.418778 | 0.26365 | -0.19141 | 0.142768 | IL1F8 |
| ILMN_3229770 | 0.01083 | 0.001184 | 0.413695 | 0.040203 | -0.30401 | 0.058534 | SKP1 |
| ILMN_2133784 | 0.004344 | 1.96E-04 | 0.227927 | -0.04438 | -0.30767 | -0.17338 | PATE2 |
| ILMN_3310055 | 0.022855 | 0.003764 | 0.305761 | 0.039608 | -0.36518 | 0.193241 | MIR760 |
| ILMN_2415786 | 0.019703 | 0.002979 | -0.37439 | 0.44337 | -0.46456 | -0.52083 | CD96 |
| ILMN_1808325 | 0.004063 | 1.56E-04 | 0.747469 | -0.05493 | -0.28998 | 0.16017 | TM4SF19 |
| ILMN_1653666 | 0.029216 | 0.005402 | 0.081742 | -0.12789 | -0.31604 | -0.01173 | KLHL7 |
| ILMN_2383107 | 0.007752 | 6.72E-04 | 0.332445 | 0.056561 | -0.26603 | 0.0058 | VPS41 |
| ILMN_2149952 | 0.006266 | 4.44E-04 | 0.022377 | 0.464067 | 0.194171 | -1.11619 | FLJ45256 |
| ILMN_1794386 | 0.014694 | 0.001931 | 0.056258 | 0.982939 | -0.22805 | 0.312029 | IL2RG |
| ILMN_2375003 | 0.019873 | 0.003027 | 0.258853 | -0.01999 | -0.4136 | 0.142819 | MAP4K4 |
| ILMN_1784141 | 0.003853 | 1.34E-04 | 0.396468 | -0.00997 | -0.24363 | 0.368239 | JAKMIP1 |
| ILMN_1813741 | 0.011121 | 0.001239 | -0.00554 | -0.18275 | 0.108005 | -0.97319 | KCNJ16 |
| ILMN_2213558 | 0.015747 | 0.002153 | -0.4531 | -0.06682 | 0.35705 | -0.4793 | TMED10P |
| ILMN_2334587 | 0.045827 | 0.010261 | 0.025913 | -0.20314 | -0.24256 | 0.294263 | HNRNPC |
| ILMN_2046611 | 0.022111 | 0.003577 | 0.285197 | -0.01232 | 0.32958 | -0.38097 | MCOLN3 |
| ILMN_2129649 | 0.005009 | 2.71E-04 | 0.611292 | -0.04818 | -0.2838 | 0.226369 | RAB27B |
| ILMN_2129650 | 0.004131 | 1.68E-04 | 0.299013 | 0.025302 | -0.33727 | 0.023833 | RAB27B |
| ILMN_1785731 | 0.004344 | 1.96E-04 | 0.61727 | 0.239537 | -0.30454 | 0.113042 | MAPK8IP2 |
| ILMN_1719010 | 0.02493 | 0.004263 | -0.2629 | 0.037472 | 0.245948 | -0.85858 | ARL4D |
| ILMN_1722718 | 0.032298 | 0.00624 | -0.10656 | -0.34647 | 0.197601 | 0.487389 | BMP2 |
| ILMN_2225104 | 0.003763 | 1.25E-04 | 0.489463 | -0.05023 | -0.26229 | 0.311581 | ZNF569 |
| ILMN_3243324 | 0.01646 | 0.00229 | 0.168569 | 0.2574 | -0.28914 | 0.16779 | FLJ35024 |
| ILMN_1699091 | 0.012886 | 0.001578 | 0.016729 | -0.27557 | 0.24295 | -0.38755 | C14orf118 |
| ILMN_2333594 | 0.004718 | 2.34E-04 | 0.258476 | -0.08073 | -0.17466 | 0.17377 | SUMO2 |
| ILMN_1760352 | 0.004851 | 2.47E-04 | 0.307237 | 0.027918 | -0.28376 | 0.102136 | C2orf52 |
| ILMN_2117736 | 0.01215 | 0.001429 | 0.638948 | -0.01038 | -0.19244 | 0.246928 | MGAT4C |
| ILMN_1807211 | 0.009786 | 9.95E-04 | 0.328842 | 0.124961 | -0.19673 | -0.05032 | NICN1 |
| ILMN_1804104 | 0.017212 | 0.002454 | 0.40625 | -0.07139 | -0.38294 | 0.232691 | ZAP70 |
| ILMN_1778764 | 0.027273 | 0.004857 | -0.16571 | -0.22308 | 0.201516 | -0.27656 | BUB3 |
| ILMN_1776000 | 0.002842 | 5.13E-05 | 0.32561 | 0.051894 | -0.32068 | 0.071204 | EID3 |
| ILMN_1742968 | 0.019873 | 0.003024 | 0.62699 | 0.001019 | -0.25266 | 0.384312 | NMNAT2 |
| ILMN_1803818 | 0.00616 | 4.28E-04 | 0.273434 | -0.04852 | -0.18102 | 0.211613 | NMNAT2 |
| ILMN_1784286 | 0.038592 | 0.007976 | 0.308885 | 0.016693 | -0.19853 | -0.04029 | NDUFA1 |
| ILMN_2356068 | 0.00716 | 5.87E-04 | 0.085758 | -0.09887 | 0.033397 | -0.5282 | CDC2L5 |
| ILMN_1697701 | 0.003834 | 1.32E-04 | 0.390076 | 0.179213 | -0.2469 | 0.146969 | PLEKHJ1 |
| ILMN_1673222 | 0.009388 | 9.33E-04 | 0.426358 | -0.03148 | -0.17399 | 0.536103 | ZBTB10 |
| ILMN_3245559 | 0.006266 | 4.45E-04 | -0.10196 | -0.08078 | 0.055863 | -0.40258 | CDK2AP1 |
| ILMN_2058782 | 0.031672 | 0.006077 | -0.36191 | -0.2996 | 0.24138 | -0.62591 | IFI27 |
| ILMN_1695604 | 0.007709 | 6.65E-04 | -0.03815 | 0.127018 | -0.0168 | -0.73833 | DCBLD1 |
| ILMN_1678968 | 0.020368 | 0.003153 | 0.418731 | 0.036233 | -0.31302 | 0.893963 | GEM |
| ILMN_1784871 | 0.038919 | 0.008089 | 0.188696 | -0.01373 | -0.28885 | -0.04996 | FASN |
| ILMN_3308555 | 0.007622 | 6.50E-04 | 0.403981 | -0.08314 | -0.3864 | 0.087442 | SNORD113-7 |
| ILMN_1673639 | 0.043223 | 0.009412 | 0.029307 | 0.041361 | 0.081741 | -0.89308 | ABI3BP |
| ILMN_2369179 | 0.022671 | 0.003716 | 0.039315 | -0.10137 | -0.24841 | 0.296966 | PREX2 |
| ILMN_2261379 | 0.001764 | 1.60E-05 | 0.586656 | -0.33025 | -0.17084 | 1.064136 | SRGAP2 |
| ILMN_1759549 | 0.010896 | 0.001194 | 0.115319 | -0.6536 | -0.22449 | 0.89743 | SRGAP2 |
| ILMN_1796847 | 0.009668 | 9.76E-04 | 0.631816 | -0.06501 | -0.15907 | 0.059972 | PITX2 |
| ILMN_1727850 | 0.03623 | 0.007337 | 0.423554 | 0.090943 | -0.34683 | 0.126951 | KCNC4 |
| ILMN_1707304 | 0.011297 | 0.001269 | 0.368276 | -0.0114 | -0.20444 | 0.057087 | KLF1 |
| ILMN_1684850 | 0.028576 | 0.00522 | 0.318876 | 0.004301 | -0.27577 | -0.07762 | PRKAR2B |
| ILMN_1708304 | 0.002424 | 3.50E-05 | 0.578838 | -0.01122 | -0.21659 | 0.212647 | C11orf16 |
| ILMN_1669753 | 0.010563 | 0.001136 | 0.318641 | -0.06172 | -0.15855 | -0.04261 | PDIA3 |
| ILMN_1685259 | 0.009655 | 9.73E-04 | 0.658277 | -0.0725 | -0.2454 | 0.142628 | SLC45A2 |
| ILMN_1727670 | 0.006056 | 4.15E-04 | 0.508941 | 0.136907 | -0.25634 | 0.20348 | FLJ44674 |
| ILMN_1786310 | 0.040732 | 0.008646 | 0.061418 | -1.07377 | 0.281296 | -0.83202 | MVK |
| ILMN_2347949 | 0.019318 | 0.002896 | 0.192441 | -0.04468 | -0.26459 | 0.134311 | G6PD |
| ILMN_2356890 | 0.013203 | 0.001632 | -0.81009 | -0.33126 | 0.052386 | 0.199609 | MRPL42 |
| ILMN_1754708 | 0.009753 | 9.88E-04 | 0.438225 | 0.108929 | -0.2855 | 0.099043 | TNK2 |
| ILMN_2382083 | 0.005573 | 3.52E-04 | 0.005696 | -0.12077 | 0.120457 | -0.38582 | CUGBP1 |
| ILMN_1763187 | 0.00725 | 5.99E-04 | 0.310513 | 0.058284 | -0.25368 | -0.0202 | PAK4 |
| ILMN_1788729 | 0.041025 | 0.008746 | 0.481533 | 0.237917 | -0.29457 | 0.024516 | TCF23 |
| ILMN_1676880 | 0.031585 | 0.006054 | 0.518187 | -0.39255 | 0.185557 | -1.23E-04 | CORO2B |
| ILMN_2399588 | 0.047742 | 0.010901 | 0.610556 | -0.27946 | -0.27533 | 0.75009 | THSD1 |
| ILMN_2054442 | 0.009259 | 9.13E-04 | -0.01692 | 0.099264 | 0.219038 | -0.72894 | ZNF146 |
| ILMN_2175712 | 0.020144 | 0.003103 | 0.277146 | -0.76821 | -0.10578 | -0.02093 | NDUFA11 |
| ILMN_1775298 | 0.008427 | 7.73E-04 | 0.379186 | -0.03778 | -0.20834 | 0.22636 | NOX5 |
| ILMN_1765674 | 0.010458 | 0.001115 | 0.322107 | -0.01238 | -0.16383 | -0.03139 | ALS2CR12 |
| ILMN_1671509 | 6.49E-07 | 2.45E-10 | 0.678353 | -0.2231 | -0.5228 | 2.369555 | CCL3 |
| ILMN_1738075 | 0.015768 | 0.002157 | 0.182089 | -0.00597 | -0.25179 | -0.00514 | CMIP |
| ILMN_1757646 | 0.037932 | 0.007798 | -0.53353 | -0.14585 | 0.176819 | -0.08131 | UFM1 |
| ILMN_1743638 | 0.006404 | 4.69E-04 | 0.391359 | 0.129208 | -0.29717 | 0.148522 | ABCG1 |
| ILMN_2328813 | 0.024563 | 0.004157 | 0.192699 | 0.206873 | -0.2727 | -0.12458 | DMAP1 |
| ILMN_1693939 | 0.003834 | 1.32E-04 | 0.331042 | 0.124681 | -0.29107 | 0.169782 | OIT3 |
| ILMN_1729529 | 0.008883 | 8.59E-04 | 0.702688 | 0.099117 | -0.21555 | 0.112561 | BAI1 |
| ILMN_1672711 | 0.014817 | 0.001957 | 0.508666 | 0.107029 | -0.17263 | 0.254717 | C10orf4 |
| ILMN_3310231 | 0.028153 | 0.005103 | 0.483735 | 0.019409 | -0.20181 | 0.188856 | MIR555 |
| ILMN_1671005 | 0.041387 | 0.00886 | -0.08224 | -0.18008 | -0.26617 | 0.423863 | IRF2BP2 |
| ILMN_1691111 | 0.003753 | 1.24E-04 | 0.16225 | 0.150912 | -0.28009 | 0.454866 | SPATA2L |
| ILMN_1701413 | 0.011556 | 0.001322 | 0.12501 | 0.034404 | -0.36747 | -0.07337 | PIGQ |
| ILMN_2293744 | 0.006493 | 4.84E-04 | 0.042422 | -0.50401 | 0.199611 | -0.54857 | MARVELD2 |
| ILMN_2082314 | 0.030586 | 0.005776 | 0.16839 | -0.04759 | -0.20306 | 0.027893 | TOM1 |
| ILMN_2203588 | 0.009993 | 0.001029 | -0.10939 | 0.09551 | 0.392314 | -0.43669 | MYL5 |
| ILMN_2047511 | 0.008858 | 8.51E-04 | 0.198048 | -0.03997 | -0.39741 | -0.13836 | CENTA1 |
| ILMN_1806601 | 0.01703 | 0.00241 | -0.09289 | -0.1372 | 0.170377 | -0.52203 | GRSF1 |
| ILMN_1791754 | 0.016564 | 0.002319 | -0.41324 | -0.19055 | 0.391067 | -0.49463 | CPT1B |
| ILMN_1704571 | 0.009117 | 8.92E-04 | 0.390069 | -0.117 | -0.26089 | 0.310518 | FAM53B |
| ILMN_1657547 | 0.020112 | 0.003092 | 0.238626 | 0.028744 | -0.21559 | -0.15577 | CCDC34 |
| ILMN_2394305 | 0.003122 | 6.88E-05 | 0.649107 | -0.10221 | -0.47185 | 0.933813 | PDGFB |
| ILMN_1677200 | 0.007899 | 6.93E-04 | 0.492962 | -0.12568 | -0.22617 | 0.008734 | CYFIP2 |
| ILMN_1651799 | 0.007468 | 6.29E-04 | 0.315531 | 0.088585 | -0.1591 | 0.044262 | SLC38A2 |
| ILMN_2408987 | 5.08E-04 | 1.61E-06 | -0.19257 | -0.01621 | 0.568823 | -1.41141 | SMARCD3 |
| ILMN_2336037 | 0.008237 | 7.46E-04 | 0.432283 | 0.243996 | -0.58962 | 1.100974 | EPN2 |
| ILMN_1707727 | 0.003144 | 7.03E-05 | 0.522933 | -0.46199 | 0.009873 | 0.85067 | ANGPTL4 |
| ILMN_1674376 | 0.002975 | 5.86E-05 | 0.325992 | -0.04205 | -0.21559 | 0.227349 | ANGPTL4 |
| ILMN_1735779 | 0.015944 | 0.002192 | 0.221429 | 0.112608 | 0.239247 | -1.61165 | KCNJ8 |
| ILMN_1730182 | 0.030591 | 0.005779 | 0.303814 | 0.051603 | -0.31456 | 0.20652 | ZFAT |
| ILMN_1711994 | 0.041594 | 0.008938 | -0.27858 | -0.16676 | -0.0672 | -1.49775 | TCIRG1 |
| ILMN_1702171 | 0.008076 | 7.18E-04 | 0.360839 | 0.019901 | -0.21284 | -0.04997 | LPCAT1 |
| ILMN_3249739 | 0.020776 | 0.003255 | 0.305889 | 0.004729 | -0.21908 | 0.079227 | FAM86A |
| ILMN_1730906 | 0.030826 | 0.005844 | -0.42275 | -0.53698 | -0.20453 | 0.675525 | FILIP1L |
| ILMN_2415157 | 0.00256 | 4.00E-05 | -0.11522 | 0.57451 | -0.29258 | 1.265501 | ARID5A |
| ILMN_1734694 | 0.0088 | 8.37E-04 | 0.261085 | 0.249605 | -0.32251 | 0.081275 | MEP1B |
| ILMN_2342068 | 0.02076 | 0.00325 | -0.0479 | -0.02586 | 0.178682 | -0.48746 | ERC1 |
| ILMN_2367530 | 0.024919 | 0.004259 | -0.09403 | -0.17708 | 0.078936 | -0.58168 | ZNF280D |
| ILMN_1800634 | 0.003279 | 8.19E-05 | 0.124892 | 0.069133 | -0.28553 | 0.05568 | NME4 |
| ILMN_2411745 | 0.030599 | 0.005782 | -0.83182 | -0.06076 | -0.31983 | 0.563486 | EML1 |
| ILMN_1719975 | 0.006645 | 5.10E-04 | 0.123532 | 0.13774 | -0.27574 | -0.3143 | HOXC4 |
| ILMN_2326713 | 0.008208 | 7.40E-04 | 0.264683 | 0.024555 | -0.28157 | -0.15695 | CD151 |
| ILMN_1714990 | 0.040224 | 0.008483 | 0.247127 | 0.021434 | -0.19279 | -0.07374 | DBT |
| ILMN_1760982 | 0.012294 | 0.001456 | 0.196302 | -0.17072 | 0.002787 | -0.62737 | ZNF187 |
| ILMN_1651905 | 0.037885 | 0.007785 | 0.538115 | 0.143809 | 0.169312 | -0.4465 | ZNF187 |
| ILMN_1789999 | 0.03376 | 0.006649 | 0.234178 | 4.27E-04 | -0.17065 | 0.049281 | SLC30A7 |
| ILMN_1790197 | 0.003956 | 1.43E-04 | 0.369273 | 0.076601 | 0.166312 | -1.40065 | FLJ20674 |
| ILMN_2362341 | 0.002919 | 5.63E-05 | 0.293732 | 0.107513 | -0.38335 | 0.126399 | L1CAM |
| ILMN_1712639 | 0.013397 | 0.001671 | 0.411998 | -0.06235 | -0.17458 | 0.066665 | AIFM2 |
| ILMN_2321301 | 0.004596 | 2.21E-04 | 0.42025 | 0.055904 | -0.25687 | 0.172345 | GRIN1 |
| ILMN_2288402 | 0.039241 | 0.008188 | -3.22E-04 | 0.027745 | -0.36929 | 0.413385 | HIP1R |
| ILMN_1741356 | 0.010399 | 0.001104 | 0.234447 | 0.04446 | -0.18268 | -0.02293 | PRICKLE1 |
| ILMN_1683666 | 0.045946 | 0.010296 | 0.027661 | 0.242244 | -0.02975 | -0.35176 | ING3 |
| ILMN_1754507 | 0.014716 | 0.001936 | 0.227449 | 0.373412 | -0.47424 | 0.256135 | IRF4 |
| ILMN_2410145 | 0.001154 | 6.65E-06 | 0.265505 | -0.14262 | -0.1401 | 2.796366 | NR4A1 |
| ILMN_1770085 | 2.20E-07 | 4.85E-11 | 0.046908 | 0.050868 | -0.2641 | 1.160137 | BTG2 |
| ILMN_3249366 | 0.029238 | 0.00541 | 0.10738 | -0.22871 | -0.30504 | 0.302111 | JMJD8 |
| ILMN_1687921 | 0.002024 | 2.31E-05 | 0.314872 | -0.0247 | -0.38505 | -0.04171 | JMJD8 |
| ILMN_2098446 | 6.46E-04 | 2.62E-06 | 0.49744 | 0.107989 | -0.29609 | 0.581123 | PMAIP1 |
| ILMN_1681916 | 0.005195 | 3.02E-04 | 0.416332 | 0.109483 | -0.1753 | 0.169512 | KCNA6 |
| ILMN_1732641 | 0.014757 | 0.001945 | 0.312981 | -0.07519 | -0.20438 | 0.10774 | ARHGAP6 |
| ILMN_1770030 | 0.012403 | 0.001479 | -0.15575 | -0.04666 | 0.250276 | -0.42081 | C7orf43 |
| ILMN_3237329 | 0.027675 | 0.004973 | 0.552079 | -0.0376 | -0.28085 | 0.166984 | FNIP2 |
| ILMN_1776631 | 0.011322 | 0.001275 | 0.21787 | -0.34873 | -0.40709 | 0.223292 | KIAA1862 |
| ILMN_1755920 | 0.017483 | 0.002514 | 0.305669 | 0.015548 | -0.23668 | 0.227818 | LOC158381 |
| ILMN_1686992 | 0.010523 | 0.001128 | 0.45316 | -0.06681 | -0.1937 | 0.10738 | TEDDM1 |
| ILMN_1805807 | 0.00314 | 6.99E-05 | 0.458017 | 0.012352 | -0.25255 | 0.164733 | SLC30A3 |
| ILMN_1713491 | 0.042576 | 0.009205 | 0.166225 | -0.04943 | -0.11789 | 0.235035 | VAMP2 |
| ILMN_1694943 | 0.00639 | 4.65E-04 | 0.439082 | 0.056929 | -0.18034 | 0.455169 | CAST |
| ILMN_3236182 | 0.005621 | 3.59E-04 | 0.189004 | 0.045325 | -0.29717 | 0.00728 | GCNT4 |
| ILMN_1805992 | 0.004024 | 1.50E-04 | -0.83629 | -0.03561 | 0.190413 | -0.441 | KIAA1598 |
| ILMN_1736888 | 0.021575 | 0.003428 | 0.216656 | -0.05772 | -0.12771 | 0.345766 | SAR1B |
| ILMN_2106818 | 0.028958 | 0.005331 | -0.14764 | -0.28977 | 0.258625 | 0.072845 | MBIP |
| ILMN_2102330 | 0.002519 | 3.76E-05 | 0.235443 | -0.12462 | -0.34508 | 0.246031 | COL8A2 |
| ILMN_1779399 | 0.004131 | 1.66E-04 | -0.33415 | 0.178565 | -0.02958 | -0.83764 | SNRPA |
| ILMN_2103774 | 0.012119 | 0.001423 | 0.521992 | 0.289189 | -0.11781 | 0.2785 | PIP5KL1 |
| ILMN_1747281 | 0.010428 | 0.00111 | 0.172945 | -0.01905 | -0.33062 | 0.186014 | EVI5L |
| ILMN_3237507 | 0.011621 | 0.001338 | -1.03131 | -0.27446 | 0.227205 | -0.29204 | LOC552889 |
| ILMN_1791400 | 0.030019 | 0.005626 | 0.340837 | -0.34737 | -0.11914 | 0.325491 | ATE1 |
| ILMN_2224907 | 0.012149 | 0.001427 | 0.223395 | 0.029134 | -0.33644 | -0.00815 | C4orf34 |
| ILMN_1713892 | 0.035707 | 0.007181 | 0.14757 | -0.12726 | -0.09125 | 0.038051 | C4orf34 |
| ILMN_2294411 | 0.0143 | 0.001854 | 0.474084 | -0.55571 | -0.21601 | 1.139555 | CTDP1 |
| ILMN_1685858 | 0.011708 | 0.001355 | 0.401507 | 0.054606 | -0.30325 | 0.176152 | MLN |
| ILMN_3243135 | 0.023231 | 0.003856 | 0.30869 | -0.21386 | -0.356 | 0.099839 | SNORA37 |
| ILMN_3243253 | 0.004789 | 2.41E-04 | 0.368363 | 0.068586 | -0.26376 | 0.141793 | LPAR3 |
| ILMN_1771203 | 0.048006 | 0.010993 | -0.43214 | -0.47684 | 0.212485 | -0.38341 | SMAD2 |
| ILMN_2127605 | 0.002898 | 5.52E-05 | 0.117837 | 0.047471 | -0.38719 | 0.096736 | LRP3 |
| ILMN_3307921 | 0.045019 | 0.010006 | 0.109989 | 0.50963 | -0.26297 | 0.87863 | PGLYRP2 |
| ILMN_1802942 | 0.007417 | 6.23E-04 | 0.369624 | 0.046256 | -0.10068 | 0.212222 | C6orf25 |
| ILMN_1690708 | 0.015768 | 0.002158 | 0.120556 | -0.1441 | -0.18499 | 0.243356 | SPTBN1 |
| ILMN_1661335 | 0.027937 | 0.005039 | 0.342196 | 0.059899 | -0.16338 | 0.033622 | SPTBN1 |
| ILMN_1651567 | 0.006583 | 4.99E-04 | 0.233829 | 0.021339 | -0.23169 | 0.21646 | WHSC1 |
| ILMN_1758356 | 0.004131 | 1.68E-04 | 0.383134 | -0.02791 | -0.14425 | 0.660575 | OR2W5 |
| ILMN_2358457 | 0.008858 | 8.51E-04 | 0.279297 | 0.069954 | -0.20725 | 0.035934 | ATF4 |
| ILMN_3235825 | 0.003408 | 9.28E-05 | -0.30857 | -0.49096 | 0.215723 | -0.73396 | UBE2QP2 |
| ILMN_1675797 | 0.023168 | 0.00384 | -0.11376 | -0.36034 | 0.186269 | -0.49298 | EPDR1 |
| ILMN_1742827 | 0.022285 | 0.003624 | 0.278659 | 1.99E-04 | -0.04537 | -0.4559 | EXOC4 |
| ILMN_2238928 | 0.008853 | 8.49E-04 | 0.565673 | 0.048429 | -0.16088 | 0.127334 | RAD51C |
| ILMN_1686434 | 0.042657 | 0.009231 | 0.229993 | 0.245892 | -0.23891 | 0.059348 | TXNDC6 |
| ILMN_1685854 | 0.011107 | 0.001236 | 0.049892 | -0.23568 | 0.286142 | -0.67059 | C5orf53 |
| ILMN_1812638 | 0.00365 | 1.17E-04 | 0.311173 | -0.14945 | -0.22362 | 0.113837 | ATP5L |
| ILMN_2195721 | 0.020611 | 0.003217 | 0.559296 | -0.04986 | -0.25304 | 0.130599 | KRTAP21-1 |
| ILMN_3224962 | 0.047719 | 0.010889 | 0.620575 | 0.038168 | -0.0351 | 0.231927 | TP63 |
| ILMN_1669966 | 0.033138 | 0.006473 | 0.280724 | 0.050928 | -0.20297 | 0.154867 | NDUFS7 |
| ILMN_1687865 | 0.03937 | 0.008229 | 0.44236 | -0.04653 | -0.24047 | 0.217925 | TMOD3 |
| ILMN_1786308 | 0.038115 | 0.007848 | 0.240622 | 0.105614 | -0.29595 | -0.11684 | NIPSNAP3B |
| ILMN_1663158 | 0.008417 | 7.71E-04 | 0.365871 | -0.05085 | -0.18999 | 0.051513 | ZNF174 |
| ILMN_1804652 | 0.025467 | 0.004402 | 0.162309 | 0.143731 | -0.00155 | -0.57335 | PLEKHH3 |
| ILMN_1707741 | 0.041235 | 0.008816 | 0.179562 | -0.02773 | -0.22592 | 0.395554 | TBC1D26 |
| ILMN_1781045 | 0.019138 | 0.00286 | 0.365222 | -0.03518 | -0.35999 | -0.25038 | FXYD2 |
| ILMN_1701681 | 0.007152 | 5.81E-04 | -0.37553 | 0.142981 | 0.392002 | -0.5668 | SEC11C |
| ILMN_1752343 | 0.037434 | 0.00766 | -0.19001 | 0.238802 | 0.177694 | -0.66298 | FAM13C1 |
| ILMN_1770772 | 0.005389 | 3.27E-04 | 0.237929 | 0.147611 | -0.15111 | 0.033692 | CMA1 |
| ILMN_1652753 | 0.001255 | 7.82E-06 | 0.595372 | -0.82971 | -0.07936 | 0.305781 | PAAF1 |
| ILMN_1715896 | 0.006682 | 5.18E-04 | -0.01899 | -0.40282 | 0.176663 | -0.60787 | PMVK |
| ILMN_1779015 | 0.00256 | 4.01E-05 | 0.204239 | 0.045918 | -0.22836 | 0.202419 | ZNF467 |
| ILMN_1813517 | 0.003724 | 1.22E-04 | 0.472944 | 0.071867 | -0.15445 | 0.090715 | DISP2 |
| ILMN_1757060 | 0.003329 | 8.57E-05 | 0.408328 | 0.10752 | -0.1936 | 0.068918 | CAMK2D |
| ILMN_3238259 | 0.035315 | 0.007088 | 0.265416 | -0.16449 | -0.24342 | -0.03004 | SNORA30 |
| ILMN_1811102 | 0.011494 | 0.001309 | 0.237339 | 0.051032 | -0.24863 | 0.026832 | LRSAM1 |
| ILMN_1716359 | 0.040081 | 0.008432 | 0.105008 | -0.02926 | -0.26696 | 0.35939 | SLC19A3 |
| ILMN_1784818 | 0.001835 | 1.69E-05 | 0.552852 | 0.008922 | -0.27775 | 0.225425 | OPN5 |
| ILMN_1768845 | 0.004543 | 2.16E-04 | 0.449224 | -0.00189 | -0.22863 | 0.214778 | DNAJB8 |
| ILMN_2078430 | 0.004918 | 2.55E-04 | 0.473236 | 0.022175 | -0.31447 | 0.177215 | MAGEB2 |
| ILMN_3244402 | 0.002417 | 3.31E-05 | 0.316684 | -0.00526 | -0.40598 | 0.307005 | RIMBP3C |
| ILMN_3241869 | 0.006461 | 4.76E-04 | 0.337298 | -0.27203 | -0.51612 | 0.162407 | SCARNA6 |
| ILMN_1718132 | 0.015046 | 0.002003 | -0.52945 | -0.26634 | 0.070364 | -0.45698 | ECHS1 |
| ILMN_1755792 | 0.029544 | 0.005497 | -0.45284 | -0.02337 | 0.263771 | -0.47123 | STK38L |
| ILMN_1783204 | 0.003531 | 1.02E-04 | 0.325659 | 0.099854 | -0.20641 | 0.131138 | CNTN4 |
| ILMN_1711422 | 0.018961 | 0.002823 | 0.38269 | 0.261867 | -0.40063 | 0.127513 | PLEKHN1 |
| ILMN_1747052 | 0.004101 | 1.64E-04 | 0.392851 | 0.033734 | -0.22942 | 0.150192 | ITGA4 |
| ILMN_1800530 | 0.035873 | 0.007227 | 0.272039 | -0.04413 | -0.22683 | 0.019797 | CENTG2 |
| ILMN_2219437 | 0.005346 | 3.22E-04 | -0.17204 | -0.16592 | 0.259934 | -0.40457 | PRRG4 |
| ILMN_1802799 | 0.014354 | 0.001866 | 0.197855 | -0.01133 | -0.20058 | 0.839694 | AKIRIN1 |
| ILMN_1658337 | 0.040329 | 0.008516 | 0.299933 | -0.11387 | -0.17848 | 0.200823 | AKIRIN1 |
| ILMN_2407799 | 0.028418 | 0.005181 | 0.217052 | 0.05016 | -0.27459 | 0.095979 | IL24 |
| ILMN_2043569 | 0.022111 | 0.003578 | 0.353246 | 0.140451 | -0.23619 | 0.130634 | MOSPD2 |
| ILMN_2373689 | 0.011858 | 0.00138 | 0.229813 | -0.07229 | -0.24111 | 0.109406 | DIAPH3 |
| ILMN_3247732 | 0.015476 | 0.0021 | 0.256888 | 0.190263 | -0.15167 | 0.205614 | ZRSR2 |
| ILMN_1773576 | 0.004373 | 1.99E-04 | -0.49606 | -0.13682 | 0.142147 | -0.15393 | CPNE3 |
| ILMN_2403566 | 0.003122 | 6.87E-05 | 0.322478 | 0.114381 | -0.27738 | 0.216067 | CSHL1 |
| ILMN_2114747 | 0.004191 | 1.75E-04 | 0.502612 | 0.032068 | -0.30674 | 0.229499 | NEIL3 |
| ILMN_1773079 | 0.008247 | 7.48E-04 | -0.15043 | 0.513144 | -0.05179 | -0.887 | COL3A1 |
| ILMN_1691223 | 0.027303 | 0.004868 | 0.326799 | -0.03685 | -0.15566 | -0.01986 | ZNF566 |
| ILMN_2382687 | 1.69E-04 | 2.95E-07 | 0.763731 | 0.613184 | -0.22143 | 3.241903 | NR4A3 |
| ILMN_1657077 | 0.004626 | 2.25E-04 | 0.217323 | 0.019072 | -0.29347 | -0.0258 | SOCS6 |
| ILMN_1778587 | 0.027665 | 0.004969 | 0.118622 | -0.0224 | -0.20683 | 0.226604 | PPP2R2D |
| ILMN_1683589 | 0.02945 | 0.00547 | 0.305296 | 0.191464 | -0.24345 | -0.30771 | PPP2R2D |
| ILMN_3258875 | 0.012754 | 0.001556 | 0.774438 | -0.01778 | -0.29726 | 0.302489 | FAM115C |
| ILMN_1814652 | 0.00499 | 2.67E-04 | 0.641412 | -0.02743 | -0.28844 | 0.120604 | NALP5 |
| ILMN_1769997 | 0.041705 | 0.008972 | 0.30956 | 0.219744 | -0.28039 | 0.752838 | NFATC1 |
| ILMN_1722538 | 0.002082 | 2.49E-05 | 0.539424 | 0.103188 | -0.23585 | 0.161988 | DEFB123 |
| ILMN_1800101 | 0.016314 | 0.002262 | 0.410555 | 0.014876 | -0.31488 | 0.144791 | HHIPL2 |
| ILMN_1717803 | 0.008183 | 7.33E-04 | 0.756047 | -0.02718 | -0.24227 | 0.063228 | CDK5R2 |
| ILMN_2179083 | 0.002421 | 3.44E-05 | 0.105345 | 0.245691 | -0.28878 | -0.55659 | LOXL4 |
| ILMN_2348093 | 0.020642 | 0.003227 | 0.248707 | -0.09691 | -0.15955 | 0.260302 | ATP5J |
| ILMN_2279873 | 0.03232 | 0.006249 | 0.171339 | 0.253611 | -0.06554 | -0.17711 | WHAMM |
| ILMN_2386732 | 0.01969 | 0.002975 | -0.44374 | 0.202016 | 0.321767 | -0.33972 | RCHY1 |
| ILMN_2255256 | 0.021185 | 0.003335 | -0.00157 | -0.0736 | 0.532976 | 0.037454 | MARVELD3 |
| ILMN_1652866 | 0.013217 | 0.00164 | 1.033504 | -0.51593 | 0.37321 | 2.106548 | LMX1B |
| ILMN_1741566 | 0.016837 | 0.002374 | 0.324396 | -0.02189 | -0.18744 | 0.217786 | BMP7 |
| ILMN_1690780 | 0.011494 | 0.001309 | 0.231733 | 0.085666 | -0.19406 | -5.64E-04 | RFK |
| ILMN_2413779 | 0.010504 | 0.001125 | -0.3178 | 0.522433 | -0.16424 | -0.7049 | SEZ6L2 |
| ILMN_1711712 | 0.003903 | 1.37E-04 | 0.294824 | -0.09313 | -0.269 | 0.281751 | GAS2L2 |
| ILMN_1721713 | 0.010504 | 0.001124 | 0.266899 | 0.047861 | -0.23391 | 0.163083 | EXOSC9 |
| ILMN_2415189 | 0.01491 | 0.001974 | 0.39874 | -0.11803 | -0.21778 | -0.17378 | ATP1A1 |
| ILMN_3240177 | 0.016059 | 0.002219 | 0.148437 | 0.078164 | -0.24769 | 0.192709 | LLPH |
| ILMN_1746712 | 0.010238 | 0.001077 | 0.5393 | -0.19378 | -0.37652 | -0.07758 | TSHR |
| ILMN_1791306 | 0.018584 | 0.002745 | 0.307643 | -0.16776 | -0.25529 | 0.414229 | C9orf103 |
| ILMN_1682828 | 0.045037 | 0.010016 | 0.58181 | 0.14611 | -0.16171 | 0.089033 | FGL1 |
| ILMN_1763560 | 0.012485 | 0.001494 | 0.356386 | -0.0435 | -0.35441 | 0.123589 | NFKBID |
| ILMN_2114422 | 0.014216 | 0.00183 | 0.110775 | 0.339441 | -0.34048 | -0.84562 | NOD1 |
| ILMN_1692962 | 0.025257 | 0.004352 | 0.28337 | 0.039862 | -0.1894 | 0.318026 | CTDSP2 |
| ILMN_1732318 | 0.027553 | 0.004931 | 0.210317 | -0.05382 | -0.29668 | 0.369578 | PTK2B |
| ILMN_2288915 | 0.016013 | 0.002207 | 0.080701 | 0.243551 | -0.11255 | -0.24597 | DNAJB14 |
| ILMN_1685415 | 0.015146 | 0.00203 | 0.28299 | 0.284887 | -0.12622 | 0.373931 | HBP1 |
| ILMN_1790918 | 0.00716 | 5.86E-04 | -0.15148 | -0.20651 | -0.17486 | 0.931434 | ZNF236 |
| ILMN_1708950 | 0.004422 | 2.04E-04 | 0.25566 | 0.075849 | -0.2199 | 0.03382 | ASPHD1 |
| ILMN_1763878 | 0.046354 | 0.010436 | 0.011588 | 0.110864 | -0.36416 | 0.135546 | CDADC1 |
| ILMN_3240730 | 0.012698 | 0.001541 | 0.18458 | 0.028909 | -0.32579 | 0.09498 | C5orf43 |
| ILMN_1660176 | 0.030396 | 0.005724 | 0.239707 | 0.067062 | -0.25567 | -0.12329 | ZNF582 |
| ILMN_1776582 | 0.026654 | 0.004706 | 0.279698 | -0.18591 | -0.11845 | 0.236889 | PDK3 |
| ILMN_2350266 | 0.018123 | 0.002643 | -0.43778 | 0.323829 | -0.20949 | 0.606976 | FBXO46 |
| ILMN_1689976 | 0.023091 | 0.003823 | 0.383485 | 0.149587 | -0.22081 | -0.15893 | EDIL3 |
| ILMN_1782032 | 0.008208 | 7.39E-04 | 0.439148 | -0.11113 | -0.01642 | -0.09032 | ASH1L |
| ILMN_1767651 | 0.038616 | 0.007988 | -0.18013 | -0.26972 | -0.18311 | 0.393458 | TECPR1 |
| ILMN_3246465 | 0.03641 | 0.007391 | 0.230963 | -0.08295 | -0.40049 | 0.074397 | SNORA16A |
| ILMN_1814113 | 0.0165 | 0.002297 | 0.240704 | -0.03343 | -0.104 | 0.175339 | ZFR |
| ILMN_2415357 | 0.012192 | 0.001436 | 0.341235 | 0.122739 | -0.18429 | 0.096707 | ARID1B |
| ILMN_1696118 | 0.003838 | 1.32E-04 | 0.386905 | 0.068277 | -0.3026 | -0.02431 | GTPBP10 |
| ILMN_2356574 | 0.019744 | 0.002989 | 0.265735 | 0.066506 | -0.24033 | -0.23868 | GTF3C2 |
| ILMN_2219681 | 0.011006 | 0.001219 | 0.364559 | 0.089402 | -0.25936 | 0.248135 | RBP2 |
| ILMN_1659029 | 8.43E-04 | 3.74E-06 | 0.198984 | 0.041163 | -0.43679 | 0.088558 | FAM116B |
| ILMN_1687275 | 0.006791 | 5.36E-04 | 0.434739 | 0.061005 | -0.15163 | 0.127613 | JUB |
| ILMN_1663489 | 0.045573 | 0.010178 | 0.113398 | -0.01354 | -0.13281 | 0.199066 | UBR2 |
| ILMN_1719616 | 0.018345 | 0.002698 | 0.873162 | -0.57081 | 0.215938 | -0.24194 | DNASE1 |
| ILMN_1779374 | 0.041795 | 0.009001 | 0.115621 | -0.2423 | 0.087663 | -0.59318 | AMMECR1 |
| ILMN_1725183 | 0.041594 | 0.008933 | 0.365051 | 0.039683 | -0.28319 | -0.1212 | TBCE |
| ILMN_1707051 | 0.004563 | 2.19E-04 | 0.322813 | 0.108566 | -0.2901 | 0.224976 | NFATC1 |
| ILMN_1688138 | 0.010157 | 0.001063 | 0.311528 | -0.0121 | -0.30439 | 0.030654 | ZNF713 |
| ILMN_1652445 | 0.005404 | 3.30E-04 | 0.088463 | 0.01546 | -0.28965 | 0.084629 | RAC1 |
| ILMN_3243441 | 0.006839 | 5.43E-04 | -0.07115 | -0.26572 | 0.403478 | -1.08981 | EEF1AL7 |
| ILMN_1669046 | 0.004882 | 2.50E-04 | -0.33892 | 0.022828 | 0.12656 | -1.00278 | FOXQ1 |
| ILMN_1702899 | 0.027962 | 0.005045 | -0.3028 | -0.0385 | 0.15352 | -0.33626 | PLEKHA7 |
| ILMN_1802246 | 0.008689 | 8.13E-04 | 0.244415 | 0.007185 | -0.25282 | 0.05324 | KLK10 |
| ILMN_1719158 | 0.016854 | 0.002378 | -0.02825 | 0.353487 | 0.414584 | -0.80846 | CTBP1 |
| ILMN_1743055 | 0.015499 | 0.002105 | 0.359269 | 0.035637 | -0.17126 | 0.03441 | NAT1 |
| ILMN_1690907 | 0.038396 | 0.007927 | -0.87751 | 0.596219 | -0.27943 | -0.49918 | CCR6 |
| ILMN_1744914 | 0.005404 | 3.29E-04 | 0.270567 | -0.15305 | -0.27688 | 0.164117 | FUCA2 |
| ILMN_1772055 | 0.004255 | 1.86E-04 | 0.501664 | -0.91433 | -0.95875 | 0.746872 | VARS |
| ILMN_1718977 | 0.01063 | 0.001146 | 0.215815 | -0.20504 | -0.06877 | 0.659379 | GADD45B |
| ILMN_1805823 | 0.006011 | 4.10E-04 | 0.147247 | 0.013602 | -0.29656 | 0.070586 | BANF2 |
| ILMN_1660636 | 0.028153 | 0.005102 | 0.031113 | -0.1418 | -0.4897 | -0.23543 | WWOX |
| ILMN_1677756 | 0.003198 | 7.28E-05 | 0.185927 | 0.069348 | -0.25193 | 0.217883 | UBE2D2 |
| ILMN_1776181 | 0.044786 | 0.009924 | -0.12355 | 0.497534 | -0.33014 | 0.108155 | BIRC3 |
| ILMN_1653750 | 0.02722 | 0.004843 | 0.904128 | 0.529602 | -0.1661 | 0.327745 | SOX10 |
| ILMN_3240168 | 0.012309 | 0.00146 | 0.488523 | -0.1076 | -0.0355 | 0.325506 | C3orf74 |
| ILMN_1790249 | 0.037486 | 0.00768 | 0.179814 | -0.01464 | -0.17955 | -0.0033 | F8A1 |
| ILMN_2295330 | 0.003622 | 1.13E-04 | 0.432808 | 0.032849 | -0.23907 | 0.230219 | NEK10 |
| ILMN_1724837 | 0.006006 | 4.09E-04 | 0.087228 | 0.15551 | -0.25219 | 0.366926 | ZC3HAV1 |
| ILMN_2338565 | 0.027559 | 0.004933 | 0.551811 | -0.34766 | -0.27704 | 0.171293 | DBNDD2 |
| ILMN_1756631 | 0.026304 | 0.00462 | -0.01728 | 0.470941 | -0.51567 | -0.29106 | ZNF526 |
| ILMN_1679973 | 0.003584 | 1.08E-04 | 0.417185 | -0.0134 | -0.19453 | 0.229321 | OR1J2 |
| ILMN_3310750 | 0.016593 | 0.002328 | 0.282458 | -0.1793 | -0.25794 | 0.337289 | MIR657 |
| ILMN_1675671 | 0.004944 | 2.60E-04 | 0.097617 | -0.13784 | -0.48695 | 0.52098 | ICOSLG |
| ILMN_1810719 | 0.043037 | 0.009354 | 0.43785 | -0.19982 | 0.05349 | -0.55508 | DCUN1D1 |
| ILMN_1684083 | 0.008975 | 8.71E-04 | 0.277558 | -0.06185 | -0.22796 | 0.073072 | RNASE10 |
| ILMN_1707350 | 0.008781 | 8.33E-04 | 0.45244 | -0.07303 | -0.36315 | 0.029856 | TUSC1 |
| ILMN_2331010 | 0.030291 | 0.00569 | -0.01099 | 0.169678 | -0.28558 | 0.678088 | TNFRSF10B |
| ILMN_1815292 | 0.032306 | 0.006243 | -0.0104 | 0.091712 | -0.11088 | -0.41823 | RPL7 |
| ILMN_1730828 | 0.03257 | 0.006322 | 0.22447 | 0.022786 | -0.25138 | 0.430009 | LOC257358 |
| ILMN_1708337 | 0.001508 | 1.24E-05 | 0.260551 | -0.0581 | -0.52174 | 4.86E-04 | NOXO1 |
| ILMN_1682747 | 0.035005 | 0.007 | 0.275427 | -0.05174 | -0.32984 | 0.071751 | FANCA |
| ILMN_2049293 | 0.024075 | 0.004043 | 0.312202 | 0.174533 | -0.1509 | 0.174531 | CD84 |
| ILMN_1691899 | 0.0123 | 0.001458 | 0.227191 | 0.144963 | -0.28673 | 0.052017 | C6orf1 |
| ILMN_1763162 | 0.020633 | 0.003225 | 0.733854 | -0.16949 | -0.25733 | 0.332619 | DPH2 |
| ILMN_2387078 | 0.031242 | 0.005959 | -0.11228 | 0.160302 | -0.09932 | -0.63375 | MPZL2 |
| ILMN_1752435 | 0.011858 | 0.001377 | 0.413592 | -0.38412 | -0.20983 | -0.00607 | SEC22C |
| ILMN_1763479 | 0.009372 | 9.30E-04 | 0.430165 | 0.008571 | -0.21619 | 0.146473 | LOC652870 |
| ILMN_2411963 | 0.018864 | 0.0028 | 0.151211 | 0.084723 | -0.16252 | 0.05808 | RBM39 |
| ILMN_1787378 | 0.017303 | 0.002475 | 0.12151 | -0.09854 | -0.16289 | 0.372994 | ADD3 |
| ILMN_1726391 | 0.035841 | 0.007214 | 0.147313 | 0.176663 | -0.17723 | 0.103526 | MRPL39 |
| ILMN_3241750 | 0.015692 | 0.002143 | 0.284587 | -0.04849 | -0.29403 | -0.04157 | CCNI2 |
| ILMN_1726704 | 0.047382 | 0.01079 | -0.48522 | -0.20345 | 0.263636 | -0.01818 | RSC1A1 |
| ILMN_2138856 | 0.046104 | 0.010348 | -0.14115 | 0.265348 | -0.08648 | 0.380446 | YOD1 |
| ILMN_1810992 | 0.001612 | 1.39E-05 | 0.873608 | -0.01534 | -0.32804 | 0.155295 | CAD |
| ILMN_1750549 | 0.027656 | 0.004966 | 0.407852 | 0.124011 | -0.18034 | 0.131645 | PI4K2A |
| ILMN_1770653 | 0.014757 | 0.001945 | 0.164481 | -0.08897 | 0.341412 | -0.40496 | MAL2 |
| ILMN_2104409 | 0.013558 | 0.001696 | 0.282806 | -0.01831 | -0.26471 | 0.110124 | RPL29P2 |
| ILMN_1695058 | 0.006493 | 4.85E-04 | 0.628643 | 0.074607 | -0.20474 | 0.008486 | SLC38A5 |
| ILMN_1798534 | 0.002236 | 2.94E-05 | 0.354369 | -0.03261 | -0.28222 | 0.197308 | OR3A1 |
| ILMN_1737585 | 0.049358 | 0.011431 | 0.255116 | 0.095282 | -0.15301 | -0.0551 | VARS2 |
| ILMN_2188966 | 0.006274 | 4.46E-04 | 0.472332 | 0.086074 | -0.28172 | 0.190114 | IGFALS |
| ILMN_2307032 | 0.006173 | 4.31E-04 | 0.535816 | 0.103757 | -0.5556 | -0.03079 | OSBPL5 |
| ILMN_1745256 | 0.002513 | 3.74E-05 | -0.25295 | 0.043047 | 0.231583 | -0.6984 | CXXC5 |
| ILMN_1662184 | 0.027303 | 0.004867 | -0.0079 | -1.16996 | 0.100457 | -0.89651 | C5orf34 |
| ILMN_1810120 | 0.004916 | 2.54E-04 | 0.500271 | 0.017726 | -0.15594 | 0.172111 | SYT7 |
| ILMN_1802205 | 4.24E-04 | 1.16E-06 | 0.145023 | -0.02041 | -0.11038 | 0.514017 | RHOB |
| ILMN_1758412 | 0.007649 | 6.58E-04 | 0.193901 | 0.080783 | -0.10762 | -0.34958 | COPS7A |
| ILMN_1683211 | 0.005404 | 3.29E-04 | 0.39361 | 0.056017 | -0.21721 | 0.199527 | NCAN |
| ILMN_3235514 | 0.004163 | 1.71E-04 | 0.390303 | 0.195775 | -0.26052 | 0.172021 | GPR183 |
| ILMN_1721978 | 0.010341 | 0.001095 | 0.161284 | 0.337175 | -0.46626 | -0.16119 | CARD11 |
| ILMN_2156936 | 0.008524 | 7.89E-04 | 0.215024 | -0.0756 | -0.30402 | 0.137242 | SCCPDH |
| ILMN_2258363 | 0.013761 | 0.001736 | 0.249458 | 0.054944 | -0.29325 | 0.062321 | KLC4 |
| ILMN_1811718 | 0.00358 | 1.07E-04 | 0.48175 | 0.062717 | -0.21682 | 0.245743 | INPP5F |
| ILMN_3239445 | 0.024224 | 0.004079 | 0.466724 | 0.123373 | -0.1254 | 0.036652 | ZBTB42 |
| ILMN_3236741 | 0.006623 | 5.04E-04 | 0.528639 | 0.291231 | -0.15012 | 0.098554 | HCG26 |
| ILMN_3310366 | 0.008458 | 7.78E-04 | 0.596844 | 0.349622 | -0.3081 | 0.270746 | MIR365-2 |
| ILMN_1738530 | 0.002863 | 5.33E-05 | -0.37448 | -0.52922 | 0.069929 | -1.11796 | ATAD3A |
| ILMN_1659773 | 0.0065 | 4.87E-04 | 0.359483 | 0.134461 | -0.21063 | 0.263966 | PCDHAC2 |
| ILMN_1758816 | 0.001508 | 1.24E-05 | -0.40153 | -0.1336 | 0.176656 | -1.33125 | UGT8 |
| ILMN_1674703 | 0.016629 | 0.002333 | -0.17256 | 0.035649 | 0.178499 | -0.82835 | UBE1DC1 |
| ILMN_1725175 | 4.64E-06 | 3.07E-09 | 0.319775 | 0.07372 | -0.38861 | 0.594527 | FOSL2 |
| ILMN_2292517 | 0.003399 | 9.20E-05 | 0.389271 | -0.03876 | -0.20618 | 0.11343 | TXNDC5 |
| ILMN_2177732 | 0.026027 | 0.004559 | 0.179052 | -0.25354 | -0.05155 | -0.26626 | RANBP6 |
| ILMN_1786707 | 0.003614 | 1.12E-04 | -0.54688 | 0.152958 | 0.162572 | -0.32042 | C19orf63 |
| ILMN_2060578 | 0.004063 | 1.58E-04 | 0.314619 | 0.083272 | -0.30791 | 0.020834 | INSL5 |
| ILMN_2377829 | 0.004194 | 1.81E-04 | -0.3554 | -0.28557 | 0.617188 | -0.5399 | NANOS1 |
| ILMN_1684492 | 0.007747 | 6.71E-04 | 0.364975 | 0.029252 | -0.21689 | 0.266733 | LEKR1 |
| ILMN_1701461 | 0.005131 | 2.92E-04 | 0.358983 | -0.13464 | -0.17225 | 0.226236 | TIMP3 |
| ILMN_1757785 | 0.006222 | 4.38E-04 | 0.815658 | -0.15439 | -0.17557 | 0.385264 | GIMAP6 |
| ILMN_2159339 | 0.005573 | 3.53E-04 | 0.608965 | 0.162994 | -0.15139 | 0.27133 | NKX6-3 |
| ILMN_1688452 | 0.003277 | 7.83E-05 | 0.403798 | -0.04405 | -0.23449 | 0.318578 | LCMT1 |
| ILMN_3243695 | 0.004255 | 1.88E-04 | 0.180256 | 0.01787 | -0.31156 | 0.101251 | APLF |
| ILMN_3224934 | 0.003329 | 8.55E-05 | 0.213616 | 0.034076 | 0.043003 | -0.36479 | SFRS18 |
| ILMN_1694389 | 0.030193 | 0.005664 | 0.388405 | 0.425576 | -0.05635 | 0.077003 | MYLC2PL |
| ILMN_1752285 | 0.003032 | 6.32E-05 | 0.308138 | -0.01697 | -0.24264 | 0.160317 | RPL4 |
| ILMN_1751396 | 0.003707 | 1.21E-04 | 0.532265 | 0.250096 | -0.37816 | 0.201021 | BAG5 |
| ILMN_1763695 | 0.049696 | 0.011524 | 0.13826 | -0.06379 | -0.36325 | 0.061343 | C9orf24 |
| ILMN_1706649 | 0.008524 | 7.89E-04 | 0.046343 | -0.09474 | -0.18629 | 0.184607 | OR4N2 |
| ILMN_2327203 | 0.025531 | 0.004421 | 0.085639 | -0.35017 | -0.01847 | 0.22321 | POT1 |
| ILMN_1670245 | 0.001711 | 1.50E-05 | 0.3744 | 0.062107 | -0.33547 | 0.010083 | KLF3 |
| ILMN_3247552 | 0.00616 | 4.27E-04 | 0.311881 | 0.067472 | -0.26108 | 0.228576 | KNCN |
| ILMN_1694730 | 0.004651 | 2.29E-04 | 0.394929 | -0.0933 | -0.26959 | 0.052303 | RNPEPL1 |
| ILMN_1697409 | 0.016705 | 0.002347 | 0.254683 | 0.035434 | -0.26323 | 0.020699 | TNFRSF14 |
| ILMN_2278265 | 0.022968 | 0.003791 | 0.527346 | 0.015905 | -0.21935 | -0.00558 | PAOX |
| ILMN_3310151 | 0.008781 | 8.33E-04 | 0.328708 | -0.1482 | -0.21962 | 0.20043 | MIR1909 |
| ILMN_2141444 | 0.005956 | 4.01E-04 | 0.331709 | 0.069582 | -0.32678 | -0.04459 | RPL18A |
| ILMN_1683277 | 0.04006 | 0.008421 | -0.08071 | 0.060231 | -0.20727 | 0.203217 | KIAA0319L |
| ILMN_1702592 | 0.005157 | 2.95E-04 | 0.11447 | 0.026275 | 0.072635 | -0.87595 | WDR73 |
| ILMN_1761598 | 0.005476 | 3.43E-04 | 0.373387 | 0.078523 | -0.26145 | 0.24768 | PRDM9 |
| ILMN_1781803 | 0.008747 | 8.25E-04 | 0.108775 | 0.064181 | -0.46819 | 0.035124 | FIZ1 |
| ILMN_1764098 | 0.041594 | 0.008936 | 0.05338 | -0.23378 | 0.262946 | -0.75826 | TRNT1 |
| ILMN_1810441 | 0.013899 | 0.001767 | 0.128103 | 0.191098 | -0.17692 | 0.327565 | TRIM3 |
| ILMN_1654570 | 0.010824 | 0.001181 | 0.33496 | 0.091943 | -0.2192 | 0.009516 | TEKT1 |
| ILMN_2342121 | 0.002418 | 3.41E-05 | 0.273995 | 0.052915 | -0.33721 | 0.119776 | PSMD13 |
| ILMN_1655930 | 0.001665 | 1.44E-05 | -0.12496 | -0.05967 | 0.039688 | 0.72616 | ELL2 |
| ILMN_1666409 | 0.006838 | 5.42E-04 | 0.283858 | -0.01504 | -0.18937 | 0.129467 | PSMB6 |
| ILMN_1671871 | 0.004255 | 1.87E-04 | 0.334052 | 0.013118 | -0.30038 | 0.136329 | ITGB1BP2 |
| ILMN_1803261 | 0.033277 | 0.006503 | 0.578768 | 0.113269 | -0.12316 | 0.154851 | OPRM1 |
| ILMN_1718060 | 0.013117 | 0.001616 | 0.379692 | 0.002861 | -0.12319 | 0.657036 | OTUD7A |
| ILMN_1761941 | 0.043333 | 0.00946 | 0.072727 | 0.315915 | -0.06806 | -0.21157 | C4orf18 |
| ILMN_2166686 | 0.0081 | 7.24E-04 | 0.412373 | 0.064099 | -0.21078 | 0.079704 | NUFIP1 |
| ILMN_1655469 | 0.022977 | 0.003795 | 0.186051 | -0.04264 | -0.21241 | 0.089639 | TSPAN3 |
| ILMN_1814619 | 0.001091 | 6.06E-06 | -0.91311 | 0.017373 | 0.241976 | -0.22603 | ZNF33A |
| ILMN_2344204 | 0.02197 | 0.003532 | 0.268127 | -0.02502 | -0.2447 | -0.0416 | PRR13 |
| ILMN_1765419 | 0.004191 | 1.79E-04 | 0.276065 | -0.00364 | -0.23625 | 0.051515 | GPLD1 |
| ILMN_2187487 | 0.023655 | 0.00395 | -0.56077 | -0.54342 | 0.127832 | -0.3207 | HEATR5B |
| ILMN_1804415 | 0.039955 | 0.008387 | 0.498379 | 0.049362 | 0.225525 | -0.63087 | SMAGP |
| ILMN_1684446 | 0.030706 | 0.00581 | -0.80962 | -0.02578 | -0.00277 | 0.047087 | SPAG7 |
| ILMN_1809894 | 0.004063 | 1.57E-04 | 0.389444 | 0.061649 | -0.21806 | -0.07447 | TMEM117 |
| ILMN_1711102 | 0.004671 | 2.31E-04 | 0.426699 | -0.42144 | -0.24149 | 0.6573 | B3GNT2 |
| ILMN_1776522 | 0.010089 | 0.001048 | 0.042949 | -0.10776 | -0.30181 | 0.085849 | RAG1AP1 |
| ILMN_1693367 | 0.009758 | 9.90E-04 | 0.34577 | -0.15481 | -0.26437 | -0.03523 | TPD52 |
| ILMN_1815063 | 0.007484 | 6.32E-04 | 0.284273 | -0.01282 | -0.33174 | -0.03012 | PXK |
| ILMN_1712456 | 0.037798 | 0.007752 | 0.787688 | -0.0353 | 0.231534 | 1.217235 | DKFZp434I1020 |
| ILMN_1695946 | 0.002851 | 5.18E-05 | 0.480452 | -0.00509 | -0.32083 | 0.150355 | TRNP1 |
| ILMN_1753279 | 0.03734 | 0.007631 | -0.35127 | -0.07773 | -0.12179 | 0.417512 | HNRNPA0 |
| ILMN_1720799 | 0.006404 | 4.70E-04 | -0.39612 | -1.14262 | 0.355662 | -0.07362 | TECR |
| ILMN_1697548 | 0.013358 | 0.001662 | -0.10735 | -0.03797 | 0.150613 | -0.47001 | LPHN2 |
| ILMN_1687332 | 0.015135 | 0.002023 | 0.361187 | -0.0205 | -0.2569 | -0.05053 | SCFD1 |
| ILMN_2178509 | 0.026594 | 0.004689 | 0.308774 | -0.08792 | -0.19727 | 0.147721 | OR52A5 |
| ILMN_1708081 | 0.029296 | 0.005439 | -0.05992 | -0.47684 | 0.350774 | -0.36576 | LCLAT1 |
| ILMN_1759341 | 0.035216 | 0.007052 | -0.60304 | 0.238464 | -0.10691 | 0.235069 | MAN2B1 |
| ILMN_2141157 | 0.027677 | 0.004975 | 0.07943 | 0.011926 | -0.27492 | 0.081487 | RANBP9 |
| ILMN_1667101 | 0.009527 | 9.55E-04 | 0.375699 | 0.089971 | -0.19395 | 0.109758 | PRM3 |
| ILMN_1796240 | 0.039344 | 0.00822 | -0.1048 | -0.08966 | 0.148168 | -0.83745 | KIAA1012 |
| ILMN_3236942 | 0.040215 | 0.008478 | 0.390684 | -0.60356 | -0.55933 | 0.379305 | SNORA5A |
| ILMN_1721563 | 0.018386 | 0.002709 | 0.275901 | 0.054283 | -0.13129 | -0.07872 | TMEM127 |
| ILMN_1757877 | 0.017551 | 0.002532 | 0.208571 | -0.00372 | -0.04826 | -0.39291 | HCFC1R1 |
| ILMN_2318568 | 0.02468 | 0.004193 | 0.251004 | 0.323435 | -0.04012 | -0.39225 | HCFC1R1 |
| ILMN_2358783 | 0.017043 | 0.002416 | 0.11686 | 0.214591 | -0.24639 | -0.3169 | ASB3 |
| ILMN_2358784 | 0.005764 | 3.78E-04 | 0.210383 | -0.10787 | 0.33446 | -0.81659 | ASB3 |
| ILMN_1676002 | 0.001154 | 6.61E-06 | 0.355856 | 0.093897 | -0.31243 | 0.175439 | QRICH1 |
| ILMN_3248304 | 0.005608 | 3.57E-04 | 0.152828 | -0.05515 | -0.25008 | 0.237359 | LIPM |
| ILMN_1670306 | 0.002919 | 5.63E-05 | 0.428764 | -0.05665 | -0.33857 | 0.154631 | SCGB3A2 |
| ILMN_1796458 | 0.021962 | 0.003529 | 0.341679 | -0.15471 | -0.09495 | 0.155829 | GABARAPL2 |
| ILMN_1760530 | 0.02398 | 0.004022 | -0.03403 | -0.69463 | 0.292437 | 0.485569 | BLOC1S2 |
| ILMN_1728496 | 0.029777 | 0.005561 | 0.456501 | 0.11777 | -0.06557 | 1.338144 | SYT9 |
| ILMN_1815392 | 0.005009 | 2.70E-04 | 0.31647 | -0.0384 | -0.29649 | 0.375474 | ACTRT1 |
| ILMN_2354855 | 0.004876 | 2.49E-04 | 0.241631 | 0.007246 | -0.35675 | -0.06175 | OTUB1 |
| ILMN_2163819 | 0.02764 | 0.004962 | 0.643452 | 0.156716 | -0.16688 | 0.201233 | KIF21B |
| ILMN_3310563 | 0.043555 | 0.009544 | 0.219535 | 0.067496 | -0.18971 | 0.240989 | MIR606 |
| ILMN_1741134 | 0.004163 | 1.71E-04 | 0.341739 | 0.019542 | -0.30876 | 0.091312 | WDR89 |
| ILMN_1754055 | 0.006623 | 5.04E-04 | 0.461605 | 0.157823 | -0.24172 | 0.117556 | APOA5 |
| ILMN_1794011 | 0.003753 | 1.24E-04 | 0.308404 | 0.096142 | -0.28458 | 0.09811 | CHST2 |
| ILMN_2362232 | 0.048996 | 0.011317 | 0.386028 | -0.11512 | -0.19453 | 0.404954 | ZNF331 |
| ILMN_2269193 | 0.009127 | 8.94E-04 | 0.312332 | -0.08276 | -0.30655 | 0.043876 | ZNF331 |
| ILMN_1675866 | 0.005237 | 3.08E-04 | 0.294328 | 0.038421 | -0.184 | 0.107994 | ARRB2 |
| ILMN_1806804 | 0.037304 | 0.007614 | -0.1689 | -0.13618 | 0.177575 | 0.678223 | USP14 |
| ILMN_1655052 | 0.015247 | 0.002054 | 0.183587 | 0.222877 | -0.16097 | -0.2148 | TRNT1 |
| ILMN_2340565 | 0.003917 | 1.38E-04 | -0.12549 | -0.05377 | -0.28472 | 0.258894 | ATP2C1 |
| ILMN_1673234 | 0.01412 | 0.001815 | 0.1935 | -0.00678 | -0.35264 | 0.080161 | PKDREJ |
| ILMN_2055036 | 0.044032 | 0.009697 | 0.283842 | 0.205999 | -0.22516 | 0.379827 | KLHDC1 |
| ILMN_3240150 | 0.024696 | 0.004198 | 0.310195 | -0.05503 | -0.35561 | -0.01034 | SNORA75 |
| ILMN_1686043 | 0.019618 | 0.002956 | 0.23408 | -0.26546 | 0.104368 | -0.51786 | FAM164C |
| ILMN_1674661 | 0.016334 | 0.002267 | 0.263137 | 0.03153 | -0.17381 | 0.192101 | CIRBP |
| ILMN_1707257 | 0.004191 | 1.77E-04 | 0.347726 | 4.74E-04 | -0.26214 | 0.135466 | HIST1H3J |
| ILMN_1673682 | 0.015813 | 0.002166 | 0.194011 | 0.03844 | -0.15151 | 0.179513 | GATAD2A |
| ILMN_1699496 | 0.005182 | 2.99E-04 | 0.339622 | 0.085728 | -0.11002 | -0.00485 | PHF21A |
| ILMN_2105033 | 0.00716 | 5.86E-04 | -0.38012 | -0.49781 | 0.456213 | -0.13024 | PLDN |
| ILMN_1803454 | 0.003122 | 6.89E-05 | 0.281763 | 0.05713 | -0.35499 | 0.252291 | PCGF3 |
| ILMN_2397954 | 0.039216 | 0.008177 | 0.245753 | 0.035778 | -0.1597 | -0.01047 | PARP3 |
| ILMN_1722872 | 0.008325 | 7.57E-04 | 0.290046 | 0.002849 | -0.14022 | 0.060015 | MYH9 |
| ILMN_1769011 | 5.71E-04 | 2.00E-06 | 0.42028 | -0.13694 | -0.25316 | -0.36453 | C8orf79 |
| ILMN_1741648 | 0.012418 | 0.001483 | 0.366788 | 1.14901 | -0.29458 | 0.829568 | HLA-DQB2 |
| ILMN_1753139 | 0.021799 | 0.00348 | 0.479404 | -0.18468 | -0.07447 | 0.530622 | LOC124220 |
| ILMN_3251477 | 0.005608 | 3.57E-04 | 0.180112 | 0.006984 | 0.206783 | -1.01982 | RG9MTD3 |
| ILMN_2372974 | 0.007623 | 6.50E-04 | 0.212871 | -0.12401 | -0.13913 | 0.93008 | SIRPA |
| ILMN_2340721 | 0.028051 | 0.005069 | 0.347163 | -0.05991 | -0.1796 | 0.211742 | TMEM134 |
| ILMN_1813296 | 0.004408 | 2.03E-04 | 0.382133 | 0.173432 | -0.31635 | 0.324733 | RDH8 |
| ILMN_1756992 | 0.030341 | 0.005708 | 0.172329 | 0.130326 | -0.24782 | -0.58415 | MUC1 |
| ILMN_1750664 | 0.038897 | 0.008076 | 0.185067 | -0.03358 | -0.30198 | 0.236349 | PPT2 |
| ILMN_1701248 | 0.022373 | 0.003652 | 0.357653 | 0.087706 | -0.1836 | 0.14683 | TREM2 |
| ILMN_3240559 | 0.003028 | 6.25E-05 | 0.349295 | -0.04473 | -0.30445 | 0.160955 | FAM189A1 |
| ILMN_1763260 | 0.028516 | 0.005204 | 0.099652 | -0.07815 | -0.1021 | 0.217052 | HIF1A |
| ILMN_2266334 | 0.028093 | 0.005079 | 0.288544 | 0.082492 | -0.17673 | -0.09373 | BCAP29 |
| ILMN_1782922 | 0.003571 | 1.06E-04 | 0.224305 | -0.04459 | -0.19658 | 1.499433 | PDE4B |
| ILMN_2347693 | 0.022825 | 0.003754 | 0.326558 | 0.054027 | -0.08672 | 0.149056 | NCOA3 |
| ILMN_2404850 | 0.044652 | 0.009874 | 0.220247 | -0.11409 | -0.23984 | 0.049927 | RPL14 |
| ILMN_1726460 | 0.009447 | 9.45E-04 | 0.257159 | -0.01366 | -0.19646 | -0.02961 | RPL14 |
| ILMN_1707783 | 0.010899 | 0.001195 | 0.245012 | -0.09561 | -0.13772 | 0.063948 | CCDC72 |
| ILMN_1677158 | 0.003654 | 1.18E-04 | 0.385378 | 0.04838 | -0.27436 | 0.196982 | IGFBPL1 |
| ILMN_1661178 | 1.45E-04 | 2.25E-07 | 0.373741 | 0.372701 | -0.38446 | 3.590157 | NR4A1 |
| ILMN_1776102 | 0.008747 | 8.25E-04 | -0.41208 | -0.25162 | 0.279978 | -0.15027 | PSMD10 |
| ILMN_1796773 | 0.042913 | 0.009317 | -0.12763 | -0.67107 | 0.124708 | -0.42367 | BTBD8 |
| ILMN_3248294 | 0.005728 | 3.73E-04 | 0.336069 | 0.124869 | -0.32308 | 0.216321 | SNORD77 |
| ILMN_1806733 | 0.004646 | 2.28E-04 | 0.111423 | 0.043752 | -0.02729 | -0.48338 | COL18A1 |
| ILMN_2113535 | 0.020241 | 0.003128 | -0.37862 | -0.42732 | 0.094024 | -0.48042 | PCYOX1 |
| ILMN_3243603 | 0.010426 | 0.001108 | 0.393827 | 0.06285 | -0.19461 | 0.087222 | LOC100129354 |
| ILMN_1742332 | 0.013892 | 0.001765 | -0.12793 | -0.07518 | 0.35017 | -0.26114 | KCTD12 |
| ILMN_1803742 | 0.003969 | 1.45E-04 | -0.11109 | 0.265086 | -0.39104 | 0.135267 | CAPZA1 |
| ILMN_1714449 | 0.031887 | 0.006141 | 0.350453 | 0.003023 | -0.17468 | 0.121806 | OR5D18 |
| ILMN_1767666 | 0.006077 | 4.18E-04 | 0.208417 | -0.16991 | -0.51238 | 0.501576 | SCN1B |
| ILMN_2155719 | 0.003751 | 1.23E-04 | 0.343221 | 0.105769 | -0.20274 | -0.10694 | NBPF10 |
| ILMN_2231906 | 0.003485 | 9.84E-05 | 0.599531 | -0.04034 | -0.27597 | 0.216209 | SPESP1 |
| ILMN_1697629 | 0.003277 | 7.84E-05 | 0.330879 | 0.108924 | -0.3202 | 0.091265 | PLA2G4B |
| ILMN_2239754 | 0.031424 | 0.006008 | 0.222549 | -0.54195 | -0.29922 | 0.481005 | IFIT3 |
| ILMN_3308068 | 0.005075 | 2.79E-04 | 0.353007 | 0.005503 | -0.3664 | 0.150345 | MIR1289-1 |
| ILMN_2098126 | 0.042574 | 0.009203 | -0.11979 | 0.304202 | -0.42099 | -0.28944 | CCL5 |
| ILMN_1742840 | 0.009429 | 9.39E-04 | 0.308193 | 0.052682 | -0.23138 | 0.200795 | COL2A1 |
| ILMN_2264634 | 0.01254 | 0.001506 | 0.485474 | 0.097864 | -0.23726 | -0.11739 | UTY |
| ILMN_2214790 | 2.92E-05 | 2.35E-08 | 0.050348 | 0.021473 | 0.420796 | -0.98319 | LAMB1 |
| ILMN_1658709 | 0.034536 | 0.006865 | 0.173152 | 0.063637 | -0.1094 | -0.32524 | LAMB1 |
| ILMN_1729509 | 0.018863 | 0.002798 | 0.288503 | -0.37445 | -0.06823 | -0.30578 | C1orf43 |
| ILMN_2382126 | 0.040463 | 0.008552 | 0.218679 | -0.66019 | -0.05879 | 0.247816 | PPFIA1 |
| ILMN_1760441 | 0.012599 | 0.001524 | 0.260503 | -0.27779 | 0.208999 | -0.6688 | MRPS5 |
| ILMN_2117526 | 0.007385 | 6.17E-04 | 0.564875 | 0.049195 | -0.26404 | 0.129331 | DMGDH |
| ILMN_1811148 | 0.0182 | 0.002665 | 0.218785 | 0.121026 | -0.1617 | 0.119454 | BMP2K |
| ILMN_3251751 | 0.011699 | 0.001352 | -0.10234 | 0.354713 | 0.039352 | -0.25606 | MFSD8 |
| ILMN_1680973 | 0.018297 | 0.002688 | 0.201649 | -0.17021 | -0.23426 | 0.288938 | FOXF1 |
| ILMN_1739659 | 0.021185 | 0.003336 | -0.18236 | -0.02343 | 0.140452 | -0.33598 | ZDHHC6 |
| ILMN_1813230 | 0.003641 | 1.16E-04 | 0.353169 | -0.0985 | -0.38966 | 0.028484 | WSCD2 |
[truncated: 185,145 more chars]
